# Influence of rapamycin on safety and healthspan metrics after one year: PEARL trial results

**DOI:** 10.18632/aging.206235

**Published:** 2025-04-04

**Authors:** Mauricio Moel, Girish Harinath, Virginia Lee, Andy Nyquist, Stefanie L. Morgan, Anar Isman, Sajad Zalzala

**Affiliations:** 1 AgelessRx, Ann Arbor, MI 48104, USA; 2 Division of Research and Applied Sciences, AgelessRx, Ann Arbor, MI 48104, USA

**Keywords:** rapamycin, aging, healthspan, longevity, geroscience

## Abstract

Design: This 48-week decentralized, double-blinded, randomized, placebo-controlled trial (NCT04488601) evaluated the long-term safety of intermittent low-dose rapamycin in a healthy, normative-aging human cohort. Participants received placebo, 5 mg or 10 mg compounded rapamycin weekly. The primary outcome measure was visceral adiposity (by DXA scan), secondary outcomes were blood biomarkers, and lean tissue and bone mineral content (by DXA scan). Established surveys were utilized to evaluate health and well-being. Safety was assessed through adverse events and blood biomarker monitoring.

Results: Adverse and serious adverse events were similar across all groups. Visceral adiposity did not change significantly (η_p_^2^ = 0.001, *p* = 0.942), and changes in blood biomarkers remained within normal ranges. Lean tissue mass (η_p_^2^ = 0.202, *p* = 0.013) and self-reported pain (η_p_^2^ = 0.168, *p* = 0.015) improved significantly for women using 10 mg rapamycin. Self-reported emotional well-being (η_p_^2^ = 0.108, *p* = 0.023) and general health (η_p_^2^ = 0.166, *p* = 0.004) also improved for those using 5 mg rapamycin. No other significant effects were observed.

Conclusions: Low-dose, intermittent rapamycin administration over 48 weeks is relatively safe in healthy, normative-aging adults, and was associated with significant improvements in lean tissue mass and pain in women. Future work will evaluate benefits of a broader range of rapamycin doses on healthspan metrics for longevity, and will aim to more comprehensively establish efficacy.

## INTRODUCTION

Aging is the greatest risk factor for all major chronic diseases, accounting for nearly 70% of human mortality [1–3]. While advancements in medical technologies and public health practices over the past 150 years have led to longer lifespans less shaped by natural selection, the period of disease and disability-free life often referred to as “healthspan” has not kept pace [4]. In conjunction with an epidemic of poor lifestyle habits, this has collectively led to a growing chasm between lifespan and healthspan known as the healthspan gap, which in the United States lasts several decades and is characterized by a high burden of functional disability and age-related diseases (such as type 2 diabetes, osteoarthritis, and Alzheimer’s) that often coexist as multi-morbidities [5]. While significant research has historically focused on treating these diseases individually, a growing body of work within translational geroscience explores developing gerotherapeutics that slow the aging process and delay the onset of or prevent age-related disease altogether [6].

The field of translational geroscience has made rapid advancements in recent years, due in large part to the strategic utilization of interventions already approved for other conditions by the US Food and Drug Administration (FDA) [7]. By repurposing such drugs for their potential to target the biology of aging and extend healthy longevity, clinical validation is fast-tracked to permit a more immediate collection of application-specific efficacy data. Notable among these is rapamycin, which is widely used for its purported longevity and healthspan benefits within the pro-longevity community [8]. While evidence supports a role for rapamycin in improving life- and health-spans in preclinical studies [9], little data exists on its clinical efficacy in normative aging humans.

As an FDA-approved small molecule drug, rapamycin is an evolutionary conserved inhibitor of the mammalian target of rapamycin serine/threonine kinase complex 1 (mTORC1), though it is known to also impact mTORC2 in certain contexts. mTORC1 is a known regulator of aging processes, and its hyperactivity has been linked to multiple chronic disease processes [10, 11]. Conversely, partial inhibition of mTORC1 induced by caloric restriction and rapamycin is hypothesized to be a major mediator of their lifespan and healthspan-enhancing effects across organisms from yeast to non-human primates [12–21]. Rapamycin has demonstrated particular efficacy as a geroprotective intervention in mice, extending lifespan in heterogeneous genetic backgrounds in both males and females across multiple studies from independent labs at multiple dosages, dosing periods, and regimens, even in elderly animals [14, 16, 21–25]. Similar effects have been reported to be conserved in companion dogs and marmosets, however, clinical data on rapamycin’s gerotherapeutic effects in humans remains limited [9, 12, 17, 26].

Given the substantial promise of preclinical data, it is essential to obtain a deeper understanding of the clinical benefits of rapamycin use for improving aging in healthy human adults. While biomarkers for evaluating rapamycin’s longevity effects are not yet well defined, body composition metrics provide a more tangible measure of factors known to be associated with age-related disease and mortality risk. Specifically, salient measures such as visceral adipose tissue (VAT) accumulation, a loss of lean muscle tissue, and loss of bone mass are all associated with reductions in quality of life (QoL), increased pain, and limited mobility, particularly for post-menopausal women [27–34]. While available evidence suggests use of low-dose rapamycin may mitigate these features of the aging process to enhance healthspan, many open questions remain [25, 35, 36].

The widespread adoption of rapamycin as a gerotherapeutic has historically been limited by concerns regarding its known impact on immunosuppression, hyperlipidemia, and hyperglycemia [37]. However, the vast majority of these effects stem from chronic daily dosing regimens utilized in severely ill organ transplants or cancer patients, where the clinical aim is inhibition of the immune system or anti-tumorigenic effects. In contrast, as a gerotherapeutic for normative aging populations, low-dose, intermittent rapamycin (commonly administered at 3–10 mg per week of standard commercial formulations or the equivalent) is revealing promise for minimizing side effects while still mitigating aspects of age-related decline [9, 38, 39]. For example, Mannick et al. demonstrated that healthy elderly individuals taking 0.5 mg of a rapalog daily or 5 mg/week for 6 weeks mitigated age-related immune decline by enhancing the adaptive immune system’s response to vaccination [39]. This supports our recent findings from a study of 333 low-dose rapamycin users indicating a high perceived QoL and improved health outcomes compared to non-users [8]. While such promising findings have encouraged some physicians to prescribe off-label rapamycin as a therapy to maintain healthspan, there are many open questions that require further study, particularly in a clinical setting.

An important gap in the clinical understanding of rapamycin for longevity is that to date, no long-term randomized controlled trials (RCT) have been conducted to explore the safety and effectiveness of low-dose, intermittent rapamycin regimens for improving multiple healthspan metrics in normative aging cohorts. The current study, the Participatory Evaluation of Aging with Rapamycin for Longevity (PEARL) trial, aimed to address this gap, and represents the longest clinical study of rapamycin use for healthy aging performed to date.

## RESULTS

A total of 114 participants completed the study and were included in data analysis. An additional 11 discontinued participation prior to study completion, and were not included in these analyses. Of the 114 who completed the study, 40 received 5 mg/week of rapamycin, 35 received 10 mg/week of rapamycin, and 39 received placebo (Supplementary Figure 1). Importantly, in the midst of this trial, we learned that compounded rapamycin, which was used for this work due to placebo generation considerations, could have reduced bioavailability relative to commercial formulations. This trial was temporarily paused while we explored this possibility in an independent cohort. It was subsequently discovered that compounded rapamycin did indeed have approximately ⅓ the concentration in blood after 24 hrs relative to commercial [40]. As such, while rapamycin doses are listed at the advertised compounded dose, it should be noted that equivalent effective doses for compounded forms are approximately 66% less.

Participant dosing groups were not significantly different at baseline on the vast majority of measures, including age, gender, weight, and BMI, however, we observed a relatively low enrollment of women across all groups (35.1% of participants overall (*n* = 40), with 20% in the 10 mg group (*n* = 8), 42.5% in the 5 mg group (*n* = 17), and 38.5% in the placebo group (*n* = 15; Supplementary Tables 1, 2 and Supplementary File 1). For those who discontinued participation, 6 were in the placebo group, 3 in the 10 mg group, and 2 in the 5 mg group. Comprehensive details regarding participants who optionally withdrew are included in Supplementary File 2 and Supplementary Table 3.

Participants who experienced serious adverse events (SAEs) are included in Supplementary File 2 and Supplementary Table 3, and included 1 event in the 10 mg group, 2 in the 5 mg group, and 3 in the placebo. With the exception of one placebo user who was withdrawn, all other participants experiencing SAEs completed the study (Supplementary Figure 2A). For non-severe adverse events (AEs), similar total numbers were reported in all groups (10 mg = 117, 5 mg = 116, placebo = 122), with no clear differences by gender (10 mg: Female = 48, Male = 69, 5 mg: Female = 57, Male = 59, placebo: Female = 76, Male = 46; χ^2^ of all comparisons non-significant). As some participants reported multiple AEs, we compared the number of participants reporting AEs (Supplementary Figure 2B). This was also found to be relatively consistent across all groups and genders (10 mg: Group = 29 (80.6%), Female = 8 (88.9%), Male = 21 (77.8%); 5 mg: Group = 31 (77.5%), Female = 13 (76.5%), Male = 18 (78.3%); placebo: Group = 34 (87.2%), Female = 13 (86.7%), Male = 21 (87.5%); Supplementary Figure 2C), though GI symptoms were reported more often for rapamycin users than placebo (10 mg = 8, 5 mg = 7, placebo = 4).

Phenotypic hallmarks of biological aging were evaluated using DXA scans of body composition after 24 and 48 weeks of treatment, specifically for measures of visceral adipose tissue (VAT), bone mineral content (BMC), bone mineral density (BMD), and lean tissue mass (LTM). Given expected differences in participant body composition and size at baseline (i.e., participants spanned a 43.18 cm range in height, 74.2 kg in weight, and BMI from 18.5–36.5; Supplementary Table 2), all DXA-based body composition measures were normalized to individual baseline as a percent change over the described time before further analysis. Following this normalization, odds ratios were calculated for all body composition measures (Table 1). While the small sample sizes in this study produced predictably large 95% confidence intervals, significant *p*-values were nonetheless observed for measures of decreased bone mineral density (OR = 0.24, 95% CI = 0.06–0.93, *p* = 0.04) and for increased lean tissue mass in females (OR = 28, 95% CI = 2.42–323.7, *p* = 0.008).

**Table 1:** Odds ratios of improvement on body composition metrics.

**Odds Ratios**									
	**All**			**Female**			**Male**		
**VAT**	**OR**	**95% CI**	*p*-value^*^	**OR**	**95% CI**	***p*-value**	**OR**	**95% CI**	***p*-value**
10 mg	1.68	0.66–4.32	0.28	4	0.61–26.12	0.15	1.12	0.37–3.40	0.84
5 mg	1.81	0.73–4.50	0.20	2.8	0.57–13.75	0.21	1.53	0.48–4.86	0.47
**BMD**									
10 mg	1.00	0.35–2.85	0.99	0.67	0.10–4.58	0.68	1.33	0.36–4.92	0.67
5 mg	0.24	0.06–0.93	0.04^*^	0.13	0.13–1.23	0.07	0.36	0.06–2.09	0.26
**BMC**									
10 mg	1.33	0.49–3.56	0.57	0.39	0.04–4.28	0.44	1.67	0.52–5.39	0.39
5 mg	0.74	0.27–2.05	0.56	0.85	0.17–4.19	0.84	0.68	0.18–2.54	0.56
**LTM**									
10 mg	2.29	0.81–4.49	0.12	28	2.42–323.7	0.008^*^	1.09	0.28–4.14	0.90
5 mg	1.66	0.59–4.63	0.34	1.67	0.32–2.42	0.54	1.66	0.44–6.26	0.45

^*^p ≤ 0.05.

**Figure 1:**
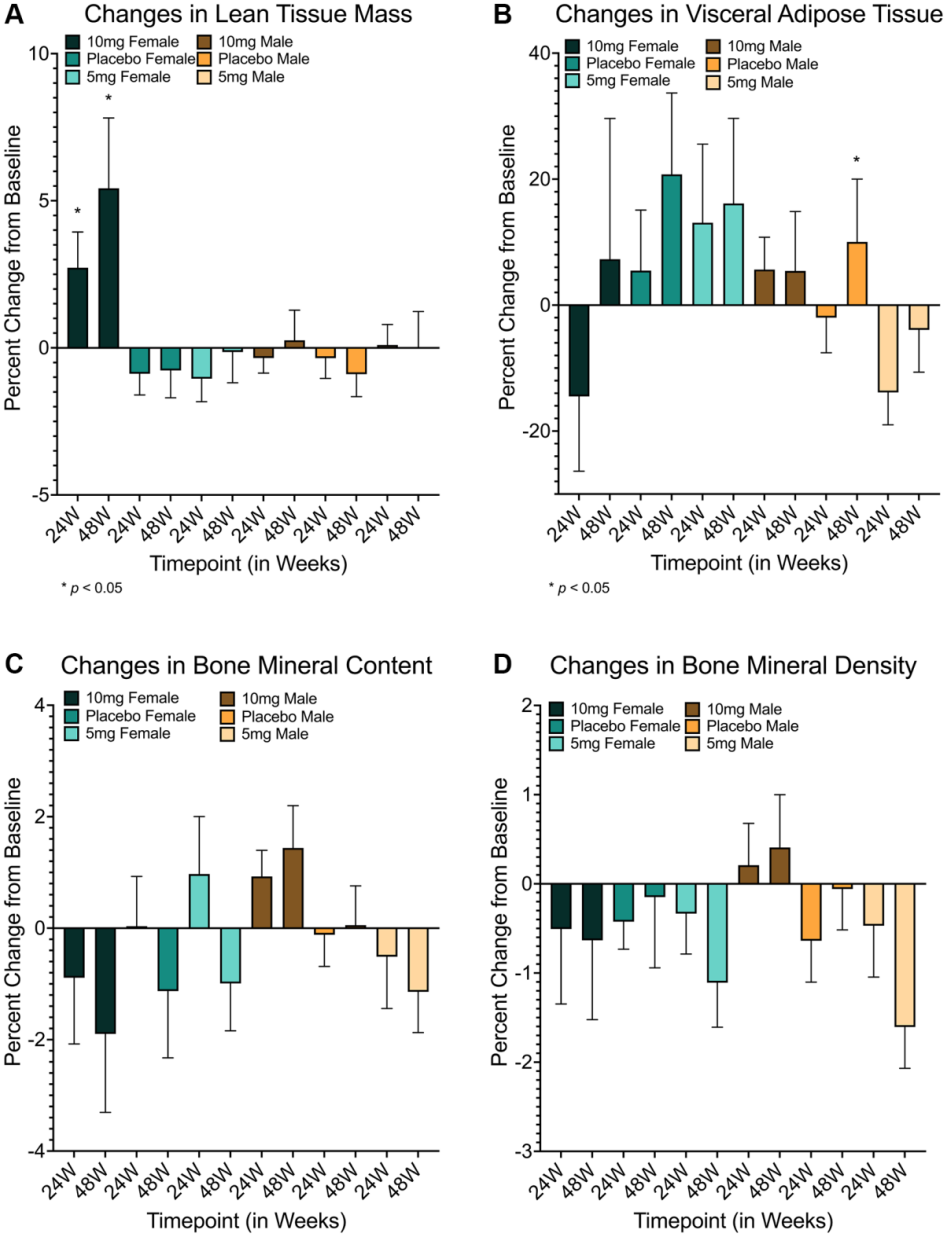
**Changes in body composition measures in response to rapamycin use.** Females using 10 mg of rapamycin had significant improvements in lean tissue mass at 24 and 48 weeks relative to both placebo (24 week: *md* = 3.60472 (95% CI = 0.0913–7.1182), *p* = 0.043; 48 week: *md* = 6.194 (95% CI = 0.8773–11.5105), *p* = 0.018) and 5mg groups (24 week: *md* = 3.774 (95% CI = 0.3271–7.2212), *p* = 0.028; 48 week: *md* = 5.565 (95% CI = 0.5311–10.5979), *p* = 0.026) (**A**). Improvements in visceral adiposity (measured by VAT) were clear for males in the 5 mg cohort relative to the 10 mg cohort (*md* = -19.520 (95% CI = −37.6513–−1.3893), *p* = 0.031) but not placebo (*md* = −11.866 (95% CI = −30.4152–6.6813), *p* = 0.362) at 24 weeks, but reverted to non-significance after 48 weeks (**B**). While no other measures showed significant differences (**C**, **D**), trending differences were observed in BMC for males at 48 weeks in 10 mg versus 5 mg groups (*md* = 2.580 (95% CI = −0.0600–5.2198), *p* = 0.057) but not placebo (*md* = 1.383 (95% CI = −1.1092–3.8757), *p* = 0.527) (**C**). *md* = mean difference, ^*^=*p* ≤ 0.05. Error bars represent standard error of the mean.

Subsequent simplified analysis (following multiple statistical approaches detailed in Supplementary Table 4) of DXA-based body composition changes at 24 and 48 weeks by dosing group suggested significant differences only in the secondary endpoint of LTM for females across dosing groups after both 24 and 48 weeks (24w: *F(2, 36)* = 4.208, *p* = 0.023, *ε^2^* = 0.144; 48w: *F(2, 30)* = 5.052, *p* = 0.013, *ε^2^* = 0.202), with Bonferroni-corrected post-hoc analyses suggesting the 10 mg group had significant increases in at both timepoints relative to both placebo and 5 mg groups (placebo −24w: *md* = 3.60472 (95% CI = 0.0913–7.1182), *p* = 0.043; 48w: *md* = 6.194 (95% CI = 0.8773–11.5105), *p* = 0.018; 5 mg–24 w: *md* = 3.774 (95% CI = 0.3271–7.2212), *p* = 0.028; 48w: *md* = 5.565 (95% CI = 0.5311–10.5979), *p* = 0.026; Table 2, Figure 1A, Supplementary Figures 3A–H and 4A). Interestingly, no significant differences were found for the primary end point of VAT after 48 weeks for either gender (Table 2, Supplementary Table 4, Figure 1B and Supplementary Figure 4B), or for the secondary end point of BMC after 48 weeks (Table 2, Figure 1C, 1D and Supplementary Figure 4C, 4D). While surprising, limited sample sizes and variability in individual response (Supplementary Figure 3E–3H) likely restricted the statistical interpretation of results for this trial cohort.

**Table 2:** Changes in body composition by DXA scan after 24 and 48 weeks.

**ANOVA of body composition changes by gender**											
**Females after 24 weeks**										**95% Confidence interval**	
	**df**	**F**	***p*-value**	Effect size^**^	**Group 1**	**Group 2**	**Mean difference**	**Std error**	***p*-value**	**Lower bound**	**Upper bound**
VAT	2, 36	1.135	0.333	0.007	10 mg	Placebo	−19.95911	18.55764	0.868	−66.5581	26.6399
						5 mg	–27.57582	18.35482	0.425	–73.6655	18.5139
					Placebo	10 mg	19.95911	18.55764	0.868	–26.6399	66.5581
						5 mg	–7.6167	15.23438	1	–45.8708	30.6374
					5 mg	10 mg	27.57582	18.35482	0.425	–18.5139	73.6655
						Placebo	7.6167	15.23438	1	–30.6374	45.8708
BMD	2, 35	0.029	0.972	–0.055	10 mg	Placebo	–0.08426	0.75078	1	–1.9721	1.8036
						5 mg	–0.17587	0.75078	1	–2.0637	1.712
					Placebo	10 mg	0.08426	0.75078	1	–1.8036	1.9721
						5 mg	–0.09161	0.6262	1	–1.6662	1.483
					5 mg	10 mg	0.17587	0.75078	1	–1.712	2.0637
						Placebo	0.09161	0.6262	1	–1.483	1.6662
BMC	2, 35	0.699	0.504	–0.017	10 mg	Placebo	–0.92574	1.60565	1	–4.9632	3.1117
						5 mg	–1.86067	1.60565	0.763	–5.8981	2.1768
					Placebo	10 mg	0.92574	1.60565	1	–3.1117	4.9632
						5 mg	–0.93493	1.3392	1	–4.3024	2.4325
					5 mg	10 mg	1.86067	1.60565	0.763	–2.1768	5.8981
						Placebo	0.93493	1.3392	1	–2.4325	4.3024
LTM	2, 36	4.208	0.023	0.144	10 mg	Placebo	3.60472^*^	1.39919	0.043	0.0913	7.1182
						5 mg	3.77419^*^	1.37276	0.028	0.3271	7.2212
					Placebo	10 mg	–3.60472^*^	1.39919	0.043	–7.1182	–0.0913
						5 mg	0.16947	1.08284	1	–2.5496	2.8885
					5 mg	10 mg	–3.77419^*^	1.37276	0.028	–7.2212	–0.3271
	Placebo	–0.16947	1.08284	1	–2.8885	2.5496
**Females after 48 weeks**											
VAT	2, 29	0.115	0.892	–0.061	10 mg	Placebo	–11.39146	24.13444	1	–72.7149	49.9319
						5 mg	–8.8483	22.97064	1	–67.2146	49.518
					Placebo	10 mg	11.39146	24.13444	1	–49.9319	72.7149
						5 mg	2.54316	18.87685	1	–45.4212	50.5075
					5 mg	10 mg	8.8483	22.97064	1	–49.518	67.2146
						Placebo	–2.54316	18.87685	1	–50.5075	45.4212
BMD	2, 35	0.575	0.568	–0.23	10 mg	Placebo	12.83206	12.06834	0.885	–17.5143	43.1784
						5 mg	7.06968	12.06834	1	–23.2767	37.416
					Placebo	10 mg	–12.83206	12.06834	0.885	–43.1784	17.5143
						5 mg	–5.76237	10.06569	1	–31.073	19.5482
					5 mg	10 mg	–7.06968	12.06834	1	–37.416	23.2767
						Placebo	5.76237	10.06569	1	–19.5482	31.073
BMC	2, 28	0.157	0.856	–0.06	10 mg	Placebo	–0.76923	1.71819	1	–5.1445	3.6061
						5 mg	–0.90536	1.666	1	–5.1478	3.3371
					Placebo	10 mg	0.76923	1.71819	1	–3.6061	5.1445
						5 mg	–0.13613	1.45586	1	–3.8434	3.5712
					5 mg	10 mg	0.90536	1.666	1	–3.3371	5.1478
						Placebo	0.13613	1.45586	1	–3.5712	3.8434
LTM	2, 30	5.052	0.013	0.202	10 mg	Placebo	6.19390^*^	2.09667	0.018	0.8773	11.5105
						5 mg	5.56454^*^	1.98498	0.026	0.5311	10.5979
					Placebo	10 mg	–6.19390^*^	2.09667	0.018	–11.5105	–0.8773
						5 mg	–0.62936	1.72141	1	–4.9944	3.7357
					5 mg	10 mg	–5.56454^*^	1.98498	0.026	–10.5979	–0.5311
						Placebo	0.62936	1.72141	1	–3.7357	4.9944
**Males after 24 weeks**											
VAT	2, 63	3.548	0.035	0.073	10 mg	Placebo	7.65337	7.46072	0.927	–10.6969	26.0036
						5 mg	19.52029^*^	7.37156	0.031	1.3893	37.6513
					Placebo	10 mg	–7.65337	7.46072	0.927	–26.0036	10.6969
						5 mg	11.86692	7.54122	0.362	–6.6813	30.4152
					5 mg	10 mg	–19.52029^*^	7.37156	0.031	–37.6513	–1.3893
						Placebo	–11.86692	7.54122	0.362	–30.4152	6.6813
BMD	2, 69	0.401	0.671	–0.017	10 mg	Placebo	–1.57949	9.98452	1	–26.079	22.92
						5 mg	–8.8775	10.3552	1	–34.2865	16.5315
					Placebo	10 mg	1.57949	9.98452	1	–22.92	26.079
						5 mg	–7.29801	10.63458	1	–33.3926	18.7965
					5 mg	10 mg	8.8775	10.3552	1	–16.5315	34.2865
						Placebo	7.29801	10.63458	1	–18.7965	33.3926
BMC	2, 60	1.311	0.277	0.01	10 mg	Placebo	1.04581	0.90798	0.762	–1.1905	3.2821
						5 mg	1.43883	0.93263	0.384	–0.8582	3.7358
					Placebo	10 mg	–1.04581	0.90798	0.762	–3.2821	1.1905
						5 mg	0.39302	0.95251	1	–1.953	2.739
					5 mg	10 mg	–1.43883	0.93263	0.384	–3.7358	0.8582
						Placebo	–0.39302	0.95251	1	–2.739	1.953
LTM	2, 62	0.162	0.851	–0.027	10 mg	Placebo	0.00392	0.88827	1	–2.1818	2.1897
						5 mg	–0.44255	0.88827	1	–2.6283	1.7432
					Placebo	10 mg	–0.00392	0.88827	1	–2.1897	2.1818
						5 mg	–0.44648	0.90823	1	–2.6813	1.7884
					5 mg	10 mg	0.44255	0.88827	1	–1.7432	2.6283
						Placebo	0.44648	0.90823	1	–1.7884	2.6813
**Males after 48 weeks**											
VAT	2, 54	0.625	0.539	0.088	10 mg	Placebo	–4.61129	12.4849	1	–35.4596	26.237
						5 mg	9.35657	12.81836	1	–22.3157	41.0288
					Placebo	10 mg	4.61129	12.4849	1	–26.237	35.4596
						5 mg	13.96786	12.6615	0.825	–17.3168	45.2525
					5 mg	10 mg	–9.35657	12.81836	1	–41.0288	22.3157
						Placebo	–13.96786	12.6615	0.825	–45.2525	17.3168
BMD	2, 69	0.219	0.804	0.033	10 mg	Placebo	2.24729	11.0736	1	–24.9245	29.4191
						5 mg	–5.39434	11.48471	1	–33.5749	22.7862
					Placebo	10 mg	–2.24729	11.0736	1	–29.4191	24.9245
						5 mg	–7.64163	11.79457	1	–36.5825	21.2992
					5 mg	10 mg	5.39434	11.48471	1	–22.7862	33.5749
						Placebo	7.64163	11.79457	1	–21.2992	36.5825
BMC	2, 52	2.949	0.061	0.221	10 mg	Placebo	1.38327	1.00751	0.527	–1.1092	3.8757
						5 mg	2.57988	1.0671	0.057	–0.06	5.2198
					Placebo	10 mg	–1.38327	1.00751	0.527	–3.8757	1.1092
						5 mg	1.19661	1.05483	0.785	–1.4129	3.8062
					5 mg	10 mg	–2.57988	1.0671	0.057	–5.2198	0.06
						Placebo	–1.19661	1.05483	0.785	–3.8062	1.4129
LTM	2, 54	0.379	0.686	0.063	10 mg	Placebo	1.15136	1.40058	1	–2.3093	4.612
						5 mg	0.23314	1.43799	1	–3.3199	3.7862
					Placebo	10 mg	–1.15136	1.40058	1	–4.612	2.3093
						5 mg	–0.91822	1.4204	1	–4.4278	2.5914
					5 mg	10 mg	–0.23314	1.43799	1	–3.7862	3.3199
						Placebo	0.91822	1.4204	1	–2.5914	4.4278

Abbreviation: df: degrees of freedom. Provided as: between groups, within groups. ^*^p ≤ 0.05. ^**^effect size provided as epsilon squared for ANOVA or omega squared for Welch's ANOVA. post hoc tests were performed using the Bonferroni correction.

As established blood biomarkers for evaluating rapamycin’s longevity impacts have not yet been well established, we examined comprehensive blood work panels for overall health and longevity signals at 0 weeks, 24 weeks, and 48 weeks of the study. These same tests were utilized for safety monitoring in this trial cohort. Multiple analyses suggested no significant changes for most values over time, and any observed changes remained within normal result windows (a complete table of results is available in Supplementary Tables 5 and 6). However, given concerns regarding rapamycin use and impacts on blood cells, insulin, and kidney health, it is worth noting that some changes were observed over the course of the study for RBCs, BUN, Hemoglobin A1C, carbon dioxide, and calcium levels. Specifically, RBCs increased for the 5 mg group but no others (*F(4, 198)* = 2.677, *p* = 0.033, η_p_^2^ = 0.051, *md* = 0.109 (95% CI = −0.189–0.003), *p* = 0.042), and BUN levels increased only for males in the 10 mg treatment group (*F(4, 102)* = 2.805, *p* = 0.030, η_p_^2^ = 0.099; *md* = 2.222 (95% CI = 0.161–4.238), *p* = 0.031). Similarly, males in the 5 mg cohort demonstrated small Hemoglobin A1C increases at 48 weeks (*F(2, 114)* = 4.821, *p* = 0.010, η_p_^2^ = 0.078; *md* = 0.059 (95% CI = 0.006–0.112), *p* = 0.024), though no significant changes were observed in glucose or insulin levels (Supplementary Tables 5 and 6). In contrast, carbon dioxide levels decreased overall in the 10 mg cohort over the course of the study (*F(2, 52)* = 7.492, *p* = 0.001, η_p_^2^ = 0.224, *md* = −1.308 (95% CI = −2.301–−0.315), *p* = 0.006), while calcium significantly decreased only for males in the 10 mg cohort (*F(2, 40)* = 3.827, *p* = 0.030, η_p_^2^ = 0.161; *md* = −0.167 (95% CI = −0.317–−0.017), *p* = 0.027).

In the interest of comprehensively evaluating rapamycin responses in our participants, we submitted a subset of samples for epigenetic aging analysis (TruAge from TruDiagnostic, *n* = 24, 9 female and 15 male) and gut microbiome analysis (Gut Health Test from Thorne, *n* = 81, 31 female and 50 male). Within the epigenetic testing results, we saw no meaningful significant changes between groups. In the gut microbiome testing, simplified analysis suggested small but significant increases after 48 weeks in gut dysbiosis in males in the 10 mg treatment group (*F(1, 18)* = 4.729, *p* = 0.045, η_p_^2^ = 0.228; *md* = 2.235 (95% CI = 0.056–4.414), *p* = 0.045), and trends of increased intestinal permeability in females in the 10 mg group (*F(1,4)* = 6.641, *p* = 0.062, η_p_^2^ = 0.624; *md* = 3.020 (95% CI = −0.234–6.274), *p* = 0.062, Supplementary Table 7).

**Figure 2:**
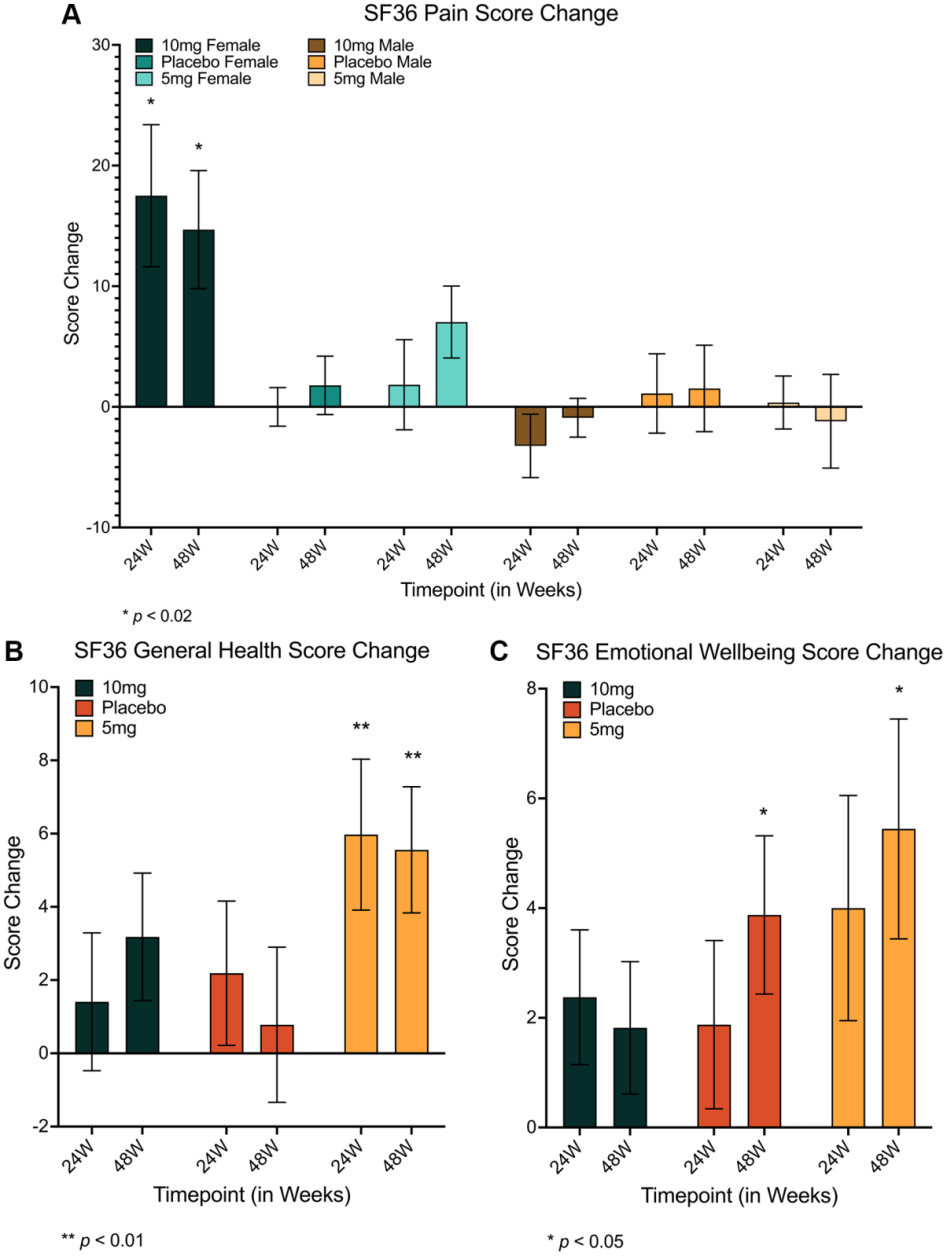
**Changes in self-reported survey scores of quality of life and health.** Females using 10 mg of rapamycin again had significant improvements in self-reported measures of pain at both 24 and 48 weeks (24 weeks: *md* = 6.765 (95% CI = 1.315–12.215), *p* = 0.011; 48 weeks: *md* = 8.071 (95% CI = 3.044–13.098), *p* < 0.001) (**A**). Additionally, improvements in measures of General Health reports were specific to the 5mg rapamycin group, increasing at 24 weeks and remaining relatively constant thereafter (24 weeks: *md* = 5.882 (95% CI = 0.388–11.376), *p* = 0.033; 48 weeks: *md* = 5.882 (95% CI = 1.350–10.415), *p* = 0.007) (**B**), however, improvements in Emotional Well-being were only seen for 5mg rapamycin users and placebo groups after 48 weeks (5mg: *md* = 5.176 (95% CI = 0.056–10.297), *p* = 0.047; placebo: *md* = 4.267 (95% CI = 0.432–8.102), *p* = 0.025) (**C**). *md* = mean difference, ^*^=*p* ≤ 0.05, ^**^p ≤ 0.01. Error bars represent standard error of the mean.

In addition to biological measures of health, the impacts of low-dose rapamycin on quality of life (QoL) measures were evaluated using validated surveys of self-reported well-being and health (the SF-36 and WOMAC scales, specifically). These were administered to study participants electronically at 0 weeks, 24 weeks, and 48 weeks. Changes in WOMAC scores over time were non-significant for all analyses across all treatment groups (Supplementary Table 8). However, multi-faceted analysis of SF-36 scores (detailed in Tables 3, 4 and Supplementary Table 9) suggested robustly significant improvements in measures of pain for females over time at both 24 and 48 weeks (*F(4, 66)* = 3.331, *p* = 0.015, η_p_^2^ = 0.168; 24w: *md* = 6.765 (95% CI = 1.315–12.215), *p* = 0.011; 48w: *md* = 8.071 (95% CI = 3.044–13.098), *p* < 0.001 Table 3 and Figure 2A), and in measures of General Health for all genders in only the 5 mg group (*F(1.757, 57.994)* = 6.582, *p* = 0.004, η_p_^2^ = 0.166; 24w: *md* = 5.882 (95% CI = 0.388–11.376), *p* = 0.033; 48w: *md* = 5.882 (95% CI = 1.350–10.415), *p* = 0.007; Figure 2B). Similarly, SF-36 measures of Emotional Well-being improved for all genders after 48 weeks for the 5 mg and placebo groups only (5 mg: *F(2, 66)* = 3.987, *p* = 0.023, η_p_^2^ = 0.108; *md* = 5.176 (95% CI = 0.056–10.297), *p* = 0.047; placebo: *F(2, 58)* = 4.265, *p* = 0.019, η_p_^2^ = 0.128; *md* = 4.267 (95% CI = 0.432–8.102), *p* = 0.025; Figure 2C, Table 4 and Supplementary Table 9). No other significant changes in SF-36 measures were observed.

**Table 3:** Changes in SF-36 self-reported measures of well-being over 48 weeks.

**Repeated mixed measure ANOVA of SF36 measures**												
**Change in scores for females**											**95% Confidence interval**	
	**df 1**	**df 2**	**F**	***p*-value**	**Partial Eta squared**	**Time 1**	**Time 2**	**Mean difference**	**Std. Error**	***p*-value**	**Lower bound**	**Upper bound**
Physical Function	4	66	0.423	0.792	0.025	Baseline	24 weeks	0.291	0.83	1	–1.804	2.385
							48 weeks	−0.462	0.842	1	–2.584	1.661
						24 weeks	Baseline	–0.291	0.83	1	–2.385	1.804
							48 weeks	–0.752	0.951	1	–3.151	1.647
						48 weeks	Baseline	0.462	0.842	1	–1.661	2.584
							24 weeks	0.752	0.951	1	–1.647	3.151
Role limitations due to physical health^^^	3.324	54.841	0.831	0.493	0.048	Baseline	24 weeks	–6.571	3.423	0.191	–15.204	2.063
							48 weeks	–4.076	4.313	1	–14.955	6.803
						24 weeks	Baseline	6.571	3.423	0.191	–2.063	15.204
							48 weeks	2.495	2.948	1	–4.941	9.931
						48 weeks	Baseline	4.076	4.313	1	–6.803	14.955
							24 weeks	–2.495	2.948	1	–9.931	4.941
Role limitations due to emotional problems	4	66	0.691	0.601	0.04	Baseline	24 weeks	–1.484	2.788	1	–8.516	5.547
							48 weeks	–0.949	2.358	1	–6.895	4.998
						24 weeks	Baseline	1.484	2.788	1	–5.547	8.516
							48 weeks	0.536	1.953	1	–4.39	5.462
						48 weeks	Baseline	0.949	2.358	1	–4.998	6.895
							24 weeks	–0.536	1.953	1	–5.462	4.39
Energy/Fatigue	4	66	0.632	0.642	0.037	Baseline	24 weeks	–5.960^*^	2.285	0.041	–11.723	–0.198
							48 weeks	–7.518^*^	2.668	0.024	–14.248	–0.788
						24 weeks	Baseline	5.960^*^	2.285	0.041	0.198	11.723
							48 weeks	–1.558	1.908	1	–6.369	3.254
						48 weeks	Baseline	7.518^*^	2.668	0.024	0.788	14.248
							24 weeks	1.558	1.908	1	–3.254	6.369
Emotional Wellbeing	4	66	0.575	0.682	0.034	Baseline	24 weeks	–4.17	1.697	0.058	–8.451	0.111
							48 weeks	–4.921	1.969	0.053	–9.888	0.045
						24 weeks	Baseline	4.17	1.697	0.058	–0.111	8.451
							48 weeks	–0.751	1.467	1	–4.451	2.948
						48 weeks	Baseline	4.921	1.969	0.053	–0.045	9.888
							24 weeks	0.751	1.467	1	–2.948	4.451
Social Functioning^^^	2.871	47.365	0.901	0.444	0.052	Baseline	24 weeks	–6.151	2.513	0.06	–12.489	0.187
							48 weeks	–6.197	2.685	0.082	–12.969	0.576
						24 weeks	Baseline	6.151	2.513	0.06	–0.187	12.489
							48 weeks	–0.045	1.402	1	–3.582	3.491
						48 weeks	Baseline	6.197	2.685	0.082	–0.576	12.969
							24 weeks	0.045	1.402	1	–3.491	3.582
Pain	4	66	3.331	0.015	0.168	Baseline	24 weeks	–6.765^*^	2.161	0.011	–12.215	–1.315
							48 weeks	–8.071^*^	1.993	<.001	–13.098	–3.044
						24 weeks	Baseline	6.765^*^	2.161	0.011	1.315	12.215
							48 weeks	–1.306	1.794	1	–5.832	3.22
						48 weeks	Baseline	8.071^*^	1.993	<.001	3.044	13.098
							24 weeks	1.306	1.794	1	–3.22	5.832
General Health	4	66	0.21	0.932	0.013	Baseline	24 weeks	–6.000^*^	2.07	0.02	–11.222	–0.778
							48 weeks	–5.063	2.048	0.056	–10.228	0.102
						24 weeks	Baseline	6.000^*^	2.07	0.02	0.778	11.222
							48 weeks	0.937	1.536	1	–2.936	4.81
						48 weeks	Baseline	5.063	2.048	0.056	–0.102	10.228
							24 weeks	–0.937	1.536	1	–4.81	2.936
**Change in scores for males**												
Physical Function^^^	3.099	86.762	0.28	0.846	0.01	Baseline	24 weeks	0.616	0.83	1	–1.432	2.663
							48 weeks	0.874	0.983	1	–1.552	3.299
						24 weeks	Baseline	–0.616	0.83	1	–2.663	1.432
							48 weeks	0.258	0.587	1	–1.191	1.707
						48 weeks	Baseline	–0.874	0.983	1	–3.299	1.552
							24 weeks	–0.258	0.587	1	–1.707	1.191
Role limitations due to physical health^^^	3.437	96.234	0.595	0.642	0.021	Baseline	24 weeks	0.858	2.592	1	–5.538	7.255
							48 weeks	2.326	3.312	1	–5.848	10.501
						24 weeks	Baseline	–0.858	2.592	1	–7.255	5.538
							48 weeks	1.468	3.758	1	–7.807	10.743
						48 weeks	Baseline	–2.326	3.312	1	–10.501	5.848
							24 weeks	–1.468	3.758	1	–10.743	7.807
Role limitations due to emotional problems^^^	3.279	91.81	0.708	0.562	0.025	Baseline	24 weeks	–2.75	3.547	1	–11.504	6.003
							48 weeks	–6.191	3.049	0.141	–13.717	1.334
						24 weeks	Baseline	2.75	3.547	1	–6.003	11.504
							48 weeks	–3.441	2.291	0.416	–9.095	2.213
						48 weeks	Baseline	6.191	3.049	0.141	–1.334	13.717
							24 weeks	3.441	2.291	0.416	–2.213	9.095
Energy/Fatigue	4	112	0.125	0.973	0.004	Baseline	24 weeks	0.118	1.597	1	–3.824	4.059
							48 weeks	–1.938	1.337	0.459	–5.239	1.363
						24 weeks	Baseline	–0.118	1.597	1	–4.059	3.824
							48 weeks	–2.055	1.441	0.478	–5.611	1.5
						48 weeks	Baseline	1.938	1.337	0.459	–1.363	5.239
							24 weeks	2.055	1.441	0.478	–1.5	5.611
Emotional Wellbeing	4	112	0.957	0.434	0.033	Baseline	24 weeks	–2.128	1.239	0.275	–5.187	0.931
							48 weeks	–3.361^*^	1.064	0.008	–5.988	–0.734
						24 weeks	Baseline	2.128	1.239	0.275	–0.931	5.187
							48 weeks	–1.233	1.061	0.75	–3.852	1.386
						48 weeks	Baseline	3.361^*^	1.064	0.008	0.734	5.988
							24 weeks	1.233	1.061	0.75	–1.386	3.852
Social Functioning	4	112	0.067	0.992	0.002	Baseline	24 weeks	–0.102	2.005	1	–5.051	4.847
							48 weeks	–1.975	1.661	0.719	–6.075	2.125
						24 weeks	Baseline	0.102	2.005	1	–4.847	5.051
							48 weeks	–1.873	1.748	0.866	–6.186	2.441
						48 weeks	Baseline	1.975	1.661	0.719	–2.125	6.075
							24 weeks	1.873	1.748	0.866	–2.441	6.186
Pain	4	112	0.313	0.869	0.011	Baseline	24 weeks	0.819	1.662	1	–3.282	4.92
							48 weeks	–0.564	1.736	1	–4.848	3.72
						24 weeks	Baseline	–0.819	1.662	1	–4.92	3.282
							48 weeks	–1.383	1.855	1	–5.96	3.194
						48 weeks	Baseline	0.564	1.736	1	–3.72	4.848
							24 weeks	1.383	1.855	1	–3.194	5.96
General Health^^^	3.265	91.413	1.875	0.134	0.063	Baseline	24 weeks	–1.966	1.495	0.582	–5.657	1.725
							48 weeks	–2.242	1.367	0.32	–5.616	1.133
						24 weeks	Baseline	1.966	1.495	0.582	–1.725	5.657
							48 weeks	–0.275	0.952	1	–2.626	2.075
						48 weeks	Baseline	2.242	1.367	0.32	–1.133	5.616
							24 weeks	0.275	0.952	1	–2.075	2.626

Abbreviation: df: degrees of freedom. provided as: between groups, within groups. ^^^denotes use of Welch’s ANOVA in instances that lack homogeneity of variances. ^*^p ≤ 0.05.

**Table 4:** Changes in SF-36 self-reported measures of Emotional Well-being and General Health over 48 weeks.

**Repeated measures ANOVA**												**95% confidence interval**	
		**df 1**	**df 2**	**F**	***p*-value**	**Partial Eta squared**	**Time 1**	**Time 2**	**Mean difference**	**Std. Error**	***p*-value**	**Lower bound**	**Upper bound**
Emotional wellbeing	10 mg	2	60	1.789	0.176	0.056	Baseline	24 weeks	−1.935	1.186	0.339	−4.943	1.072
								48 weeks	−1.935	1.27	0.414	−5.156	1.285
							24 weeks	Baseline	1.935	1.186	0.339	−1.072	4.943
								48 weeks	0	1.082	1	−2.743	2.743
							48 weeks	Baseline	1.935	1.27	0.414	−1.285	5.156
								24 weeks	0	1.082	1	−2.743	2.743
	Placebo	2	58	4.265	0.019	0.128	Baseline	24 weeks	−2.533	1.516	0.316	−6.385	1.319
								48 weeks	−4.267^*^	1.509	0.025	−8.102	−0.432
							24 weeks	Baseline	2.533	1.516	0.316	−1.319	6.385
								48 weeks	−1.733	1.379	0.656	−5.237	1.77
							48 weeks	Baseline	4.267^*^	1.509	0.025	0.432	8.102
								24 weeks	1.733	1.379	0.656	−1.77	5.237
	5 mg	2	66	3.987	0.023	0.108	Baseline	24 weeks	−4.471	2.121	0.128	−9.821	0.88
								48 weeks	−5.176^*^	2.03	0.047	−10.297	−0.056
							24 weeks	Baseline	4.471	2.121	0.128	−0.88	9.821
								48 weeks	−0.706	1.799	1	−5.243	3.831
							48 weeks	Baseline	5.176^*^	2.03	0.047	0.056	10.297
								24 weeks	0.706	1.799	1	−3.831	5.243
General health	10 mg	1.35^^^	40.509	1.805	0.186	0.057	Baseline	24 weeks	−1.452	1.943	1	−6.378	3.474
								48 weeks	−3.065	1.791	0.292	−7.607	1.478
							24 weeks	Baseline	1.452	1.943	1	−3.474	6.378
								48 weeks	−1.613	0.91	0.259	−3.919	0.693
							48 weeks	Baseline	3.065	1.791	0.292	−1.478	7.607
								24 weeks	1.613	0.91	0.259	−0.693	3.919
	Placebo	1.737^^^	50.381	1.231	0.296	0.041	Baseline	24 weeks	−3	2.014	0.442	−8.118	2.118
								48 weeks	−0.833	2.263	1	−6.583	4.916
							24 weeks	Baseline	3	2.014	0.442	−2.118	8.118
								48 weeks	2.167	1.584	0.546	−1.859	6.193
							48 weeks	Baseline	0.833	2.263	1	−4.916	6.583
								24 weeks	−2.167	1.584	0.546	−6.193	1.859
	5 mg	1.757^^^	57.994	6.582	0.004	0.166	Baseline	24 weeks	−5.882^*^	2.178	0.033	−11.376	−0.388
								48 weeks	−5.882^*^	1.797	0.007	−10.415	−1.35
							24 weeks	Baseline	5.882^*^	2.178	0.033	0.388	11.376
								48 weeks	0	1.594	1	−4.02	4.02
							48 weeks	Baseline	5.882^*^	1.797	0.007	1.35	10.415
								24 weeks	0	1.594	1	−4.02	4.02

Abbreviation: df: degrees of freedom. Provided as: between groups, within groups. ^^^denotes use of Welch's ANOVA in instances that lack homogeneity of variances. ^*^p ≤ 0.05.

## DISCUSSION

Few clinical trials to date have evaluated the effects of rapamycin and its derivatives in generally healthy individuals, and those that have been conducted are often challenged by small cohort size, short-term follow-up, or both. While the most robust of these studies have suggested improvements in age-related immune decline in healthy elderly individuals administered low-dose everolimus for 6 to 16 weeks [39], many questions regarding low-dose rapamycin for supporting healthy aging in normative aging individuals remain, particularly regarding the safety of long-term low-dose use. The PEARL trial represents one of the largest efforts to date for evaluating the long-term safety of low-dose rapamycin for longevity in a normative aging cohort, and provides preliminary support for the suggestion that low-dose rapamycin may be useful in combating age-related decline by improving healthspan measures.

The primary goal of the current study was to evaluate the relative safety of low-dose rapamycin use over 48 weeks, and to evaluate whether any clear patterns of concerning side effects emerged in a preliminary cohort. Overall, reports of adverse events (AEs) were relatively consistent across all groups. While rapamycin users appeared to have more GI symptoms than placebo users, no other clear patterns of AEs for rapamycin users emerged. AEs resulting in participant study withdrawal or serious AEs (SAEs) were also similar across groups, with many of the most severe outcomes in the placebo groups. Particular attention was given to immune challenge symptoms for rapamycin users; however, overall reports of cold/flu-like illness and slowed recovery were similar across all groups. There was a single report of anemia in the entire study, in a participant in the 5 mg treatment group. While it resolved with treatment (blood transfusion), did not recur, and the participant completed the entire trial, we highlighted the incidence given interest in this specific outcome for low-dose rapamycin users.

Preliminary investigations of possible efficacy were also explored in this study, however, findings are limited by the small relative cohort size and require both replication and extension before definitive guiding conclusions can be drawn. Specifically, a few key limitations to the study should be noted. First, adherence to the once-weekly dosing schedule was based largely on self-report; missed doses or irregular dosing could have impacted treatment effect. Second, our cohort demographics showed relatively few women and predominantly health-conscious participants, which could mask larger effects in populations with higher baseline adiposity or different lifestyle patterns. Third, only broad measures of diet and activity were captured in self-reports, leaving these factors as a plausible source of unexplained variance in outcomes. Indeed, we hypothesize that the lack of significant differences between dose groups for our primary outcome measure of visceral adiposity can largely be explained by a combination of these factors. For example, in addition to the cohort size limitations, the participants in this trial were notably health-conscious at baseline (e.g., lower BMI range, healthier diet/exercise habits), which likely limited the potential to detect meaningful VAT changes.

Nonetheless, we saw strong improvements in the secondary outcome measure of lean tissue mass, and in self-reported pain symptoms for women taking 10 mg of compounded rapamycin (equivalent to ~3.33 mgs of generic Sirolimus). We further observed modest improvements in other measures of self-reported well-being for some groups in both genders (general health and emotional well-being). These effects are largely in keeping with the suggested benefits of low-dose rapamycin use in the longevity community, and provide some measure of clinically validated support for rapamycin’s reputed effects on this front despite the small sample numbers. Indeed, this may lend even greater support to the likelihood that rapamycin has meaningful longevity benefits, as any evidence of efficacy with such small numbers is decidedly unexpected. While future studies will be required to more fully understand these effects, and should include a broader dosing range as well as a larger cohort, these findings provide a foundation upon which to build further investigation into the health and longevity effects of low-dose rapamycin.

Taken together, findings from the PEARL trial are the largest and longest to date for evaluating the safety and efficacy of low, intermittent “longevity doses” of rapamycin on healthy aging through the measurement of clinically relevant healthspan metrics. Our findings provide evidence that these rapamycin regimens are well tolerated with minimal adverse effects when administered for at least one year within normative aging individuals. Although the lack of significant VAT change (our primary endpoint) indicates that rapamycin may not strongly influence visceral adiposity in this population, we nonetheless observed some benefits for rapamycin users, particularly women, who had significant improvements in lean muscle mass and self-reported pain. While further investigation into low-dose, intermittent rapamycin’s longevity effects is undoubtedly required and indeed is ongoing, this study provides evidence that rapamycin taken in this manner is relatively safe, and lays the foundation upon which larger and more detailed studies may be developed in the future. Collectively, this and future work aims to build evidence that beyond merely clinical measures of health improvements, rapamycin may promote essential, comprehensive well-being associated with “adding life to years, not just years to life”.

## METHODS

### Study design

The PEARL study was a decentralized, single-center, prospective, double-blind, placebo-controlled trial assessing rapamycin in healthy individuals aged 50–85 years, to determine the safety and efficacy in mitigating aging-related decline (Supplementary Figure 1). It was registered as a clinical trial on 2020-07-28, NCT04488601, and was conducted in accordance with the standards of Good Clinical Practice, as defined by the International Conference on Harmonisation and all applicable federal and local regulations. The study protocol was approved by the institutional review board of the Institute of Regenerative and Cellular Medicine in May 2020 (IRCM; approval number IRCM-2020-252). Discussions with the FDA determined that this study was exempt from IND requirements. To ensure fidelity of the double-blinded design, randomization and dispensing of medications was managed by the distributing pharmacy partner, and kept confidential from the participants and AgelessRx study staff until the trial was concluded.

### Study endpoints

The primary endpoint of this study was changes in visceral fat as measured by dual-energy x-ray absorptiometry (DXA) scan. Secondary endpoints included changes in lean tissue mass and bone density as determined by DXA scan, as well as changes in blood biomarkers from complete blood count (CBC), blood electrolytes, liver function, renal function, serum glucose, insulin, and hemoglobin A1C. Standardized self-reported surveys of quality of life (SF36, [41]) and frailty (WOMAC, [42]) were also completed by study participants, but were not included as specific study endpoints.

### Study population

Participants were recruited and screened for eligibility via the AgelessRx online medical platform. If deemed eligible, informed consent was obtained for participation in the study. Participants were eligible for the study if they were aged between 50 and 85 years at the start of the study, were interested in taking rapamycin off-label, were willing to undergo minimally invasive tests, and were in good health or had well-managed clinically-stable chronic diseases. Participants were excluded from the study if they had anemia, neutropenia, or thrombocytopenia, were premenopausal, were scheduled to undergo major surgery in next 12 months, were undergoing or were scheduled to undergo chemotherapy, were scheduled for immunosuppressant therapy for an organ transplant, had impaired wound healing or history of chronic open wound, untreated dyslipidemia, impaired hepatic function, chronic infections requiring ongoing treatment or monitoring (e.g., human immunodeficiency virus/acquired immunodeficiency syndrome, chronic Lyme disease), allergy to rapamycin, clinically-relevant primary or secondary immune dysfunction or deficiency, chronic oral corticosteroid or immunosuppressive medication use, fibromyalgia, chronic fatigue syndrome/myalgic encephalomyelitis, breast implant illness, congestive heart failure, impaired renal function, poorly controlled diabetes, type I or insulin-dependent type II diabetes, untreated or treated within the last five years for substance abuse disorder, and untreated or poorly controlled mental health disorder. Further, those who had recently taken or were taking metformin, rapamycin, or rapalogs were excluded unless the participant agreed to a 6-month washout period prior to the start of the trial.

### Treatments

Rapamycin used in this study was a compounded formulation of 5 mg or 10 mg, received from Belmar Pharma Solutions (Golden, CO, USA). Placebo capsules were also formulated by Belmar, and were designed to have a similar appearance to rapamycin capsules. Both were taken orally. Study participants were randomized into three groups by the Belmar Pharmacy Solutions staff who dispensed medications accordingly: receiving 5 mg of compounded rapamycin, 10 mg of compounded rapamycin, or placebo, once per week by mouth. Participants were instructed to take the medication with or without food. Study participants were prescribed the drug for 48 weeks upon enrollment in the study and were dispensed supplies for 12 weeks at a time. Both participants and AgelessRx research staff were blinded to the randomization assignments until the trial was completed. After completion and unblinding of the trial, participants receiving the placebo were given 1 year of no-cost compounded rapamycin if desired, and were monitored for any adverse side effects.

### Assessments

All assessments were performed for all randomization groups at baseline, after 24 weeks, and after 48 weeks of rapamycin treatment, and included comprehensive blood testing, DXA body composition scans, and established self-report surveys (SF36 and WOMAC scales). Testing was overseen by AgelessRx staff blinded to participant randomization group until the trial was completed. Safety-based blood testing (including Triglycerides, Total Cholesterol, LDL-Cholesterol, Glucose, Creatinine, ALT, WBC, RBC, Hemoglobin, Hemoglobin A1C, and ApoB) was performed two additional times (at 2 weeks and 4 weeks of treatment for all markers except Hemoglobin A1C and ApoB, which were limited to baseline, 24 weeks, and 48 weeks) to evaluate safety. All blood testing was performed by local Quest Diagnostics or LabCorp laboratories, and included complete blood count (CBC), comprehensive metabolic panel, liver function tests, renal function tests, lipid panels, and insulin/glucose monitoring panels. Participants were asked to fast the night before blood draws.

DXA scans were performed by designated partner facilities DexaFit and Fitnescity at locations convenient for the participants, and were used to measure visceral adiposity, bone density (from both bone mineral content and bone mineral density), and lean tissue mass. AgelessRx staff assisted participants in finding and scheduling appointments at these facilities as needed. For participants for whom neither partner facility had a convenient nearby location, alternative facilities were identified in conjunction with the AgelessRx staff. For all DXA scan facilities, scans were completed by trained technicians familiar with equipment calibration and function, appropriate patient positioning, and necessary safety protocols. For all participants, the following procedures were used:

Pre-Scan, participants were advised to avoid calcium supplements and certain medications for 24–48 hours before the scan to prevent interference with results. They were asked to avoid exercise prior to the scan and come in well-hydrated. Additionally, they need to fast 2–3 hours prior to their scan, and inform the technician of any recent surgeries, fractures, or medical conditions that may impact the scan. During the scan, they were asked to wear loose, metal-free clothing, and to remove metal objects such as jewelry or belts to avoid artifacts.

All DXA measures are obtained by comparing the X-ray attenuation in the designated region or tissue type. Measurements are reported in grams or grams per cm^2^. Results are compared to those of national averages for a participant’s given age, gender, and race.

Measures of gut microbiome health were evaluated using the at-home Thorne Gut Health test (Thorne), and measures of epigenetic age were evaluated using the at-home TruDiagnostic TruAge kit (TruDiagnostic). Results were provided with the kit, and interpreted versions were returned to AgelessRx researchers for correlation and comparison with dosing groups and other measures reported in this manuscript.

Health-related QoL was assessed using electronic versions of standardized, validated surveys. The Short-Form 36 (SF-36) survey, which consists of 36 questions covering eight health domains, was used to assess physical functioning, role limitations due to physical health, bodily pain, general health perceptions, vitality (energy levels), social functioning, role limitations due to emotional problems, and emotional health [41]. The responses were scored and summarized to provide a profile of an individual’s perceived health status. Pain, fitness, and functional limitations were further assessed using the Western Ontario and McMaster Universities Osteoarthritis (WOMAC) index, which is a questionnaire that is commonly used to assess the health status of individuals with osteoarthritis of the hip and knee [42]. It consists of 24 items divided into three subscales: pain, stiffness, and physical function. The responses are scored to provide quantitative assessments of the severity of symptoms and functional limitations associated with osteoarthritis.

### Participant protocol adherence, data quality control and adverse event monitoring

At the onset of the trial participants receive an emailed copy of a participant guide that included general instructions on getting started and important timepoints. Participants were monitored routinely throughout the study through weekly email surveys and 4 virtual meetings at regular timepoints throughout the trial. They were asked to schedule all 4× virtual check-in meetings (via Calendly) with clinical trial staff at the time of onboarding. These meetings were conducted in 2 weeks (after 1st dose date), 4 weeks, 6 months, and the final 12-months. During these meetings, any outstanding items that participants had not yet completed would be reviewed and documented. Items included verifying weekly administration of their medication, completing weekly check-in surveys (WOMAC, SF36, adverse events, etc.), completing at-home kits (as relevant), scheduling and completing Quest blood draws as required, and completing DXA scans as required. All participant tasks, check-ins, adverse event reporting, and ongoing participant support during the study were tracked and conducted by a contract research organization and an internal project manager, all of whom remained blinded to the treatment condition participants had been assigned to by the pharmacy until the trial was completed. Clinical trial staff had internal project management sheets to monitor participants and their scheduled tasks, as they progressed through the trial. Participants were emailed in a timely manner to ensure participant compliance with intervention regimen and tasks were completed, inform of upcoming tasks and provide support as needed. Any missed meetings due to participant schedule conflicts/no-shows/cancellations would be re-scheduled or followed up in a timely manner with participants to ensure trial compliance and support as needed.

Adverse events (AEs) were obtained through weekly monitoring forms sent out to participants. Clinical trial staff reviewed and documented all AEs, and conferred with medical staff as necessary to determine if individuals should be removed from the study for any specific AEs or serious AEs. A full list of AEs, withdrawn patients, and SAEs is presented in Supplementary Table 3 and Supplementary File 2.

### Statistical analysis

Data were analyzed using tests as described in the text, with relevant corrections for sphericity (Greenhouse-Geisser), homogeneity of variance (Welch’s test), and multiple comparisons (Bonferroni or Games-Howell correction), unless otherwise noted. All analyses were conducted using SPSS 29.0.2.0 (IBM, Armonk, NY, USA). Not all participants completed all datapoints, thus in some tests, the number of cases will differ from the total number of study participants. For each test, the maximum number of data points was utilized for comparisons, with pairwise removal for missing values.

## Supplementary Materials

Supplementary Files

Supplementary Figures

Supplementary Table 1 and 8

Supplementary Table 2-7 and 9
