# Supplementary material for: Influence of rapamycin on safety and healthspan metrics after one year: PEARL trial results
Source: Aging (Albany NY). 2025 Apr 4;17(4):908–36. doi: 10.18632/aging.206235 (PMC12074816; doi:10.18632/aging.206235)
Supplement: Supplementary Figures [file aging-17-206235-s002.pdf]

SUPPLEMENTARY FIGURES

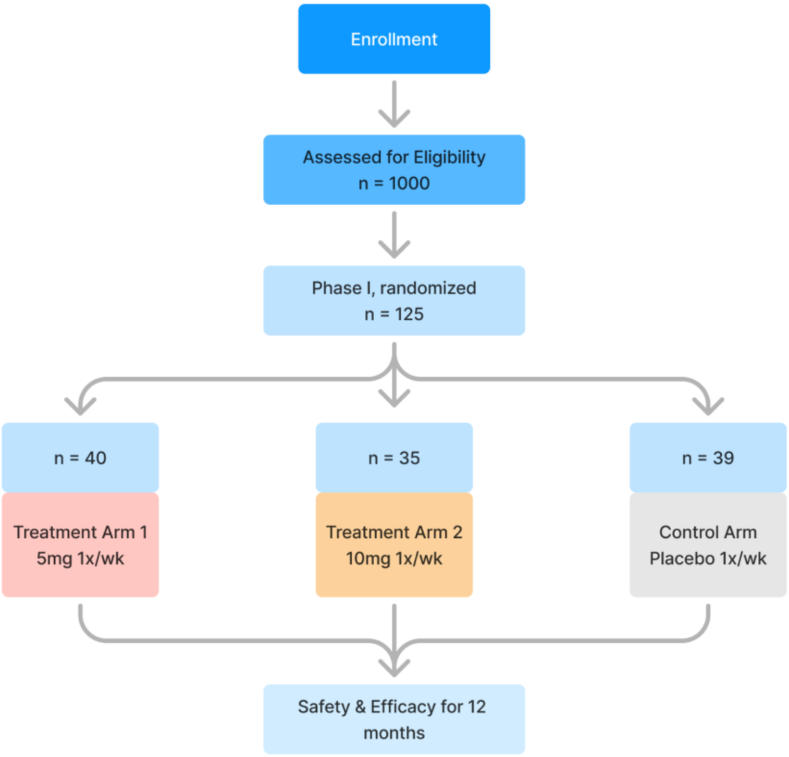

Supplementary Figure 1. PEARL trial design. Schematic of trial enrollment, participant screening, randomization, and follow-up.

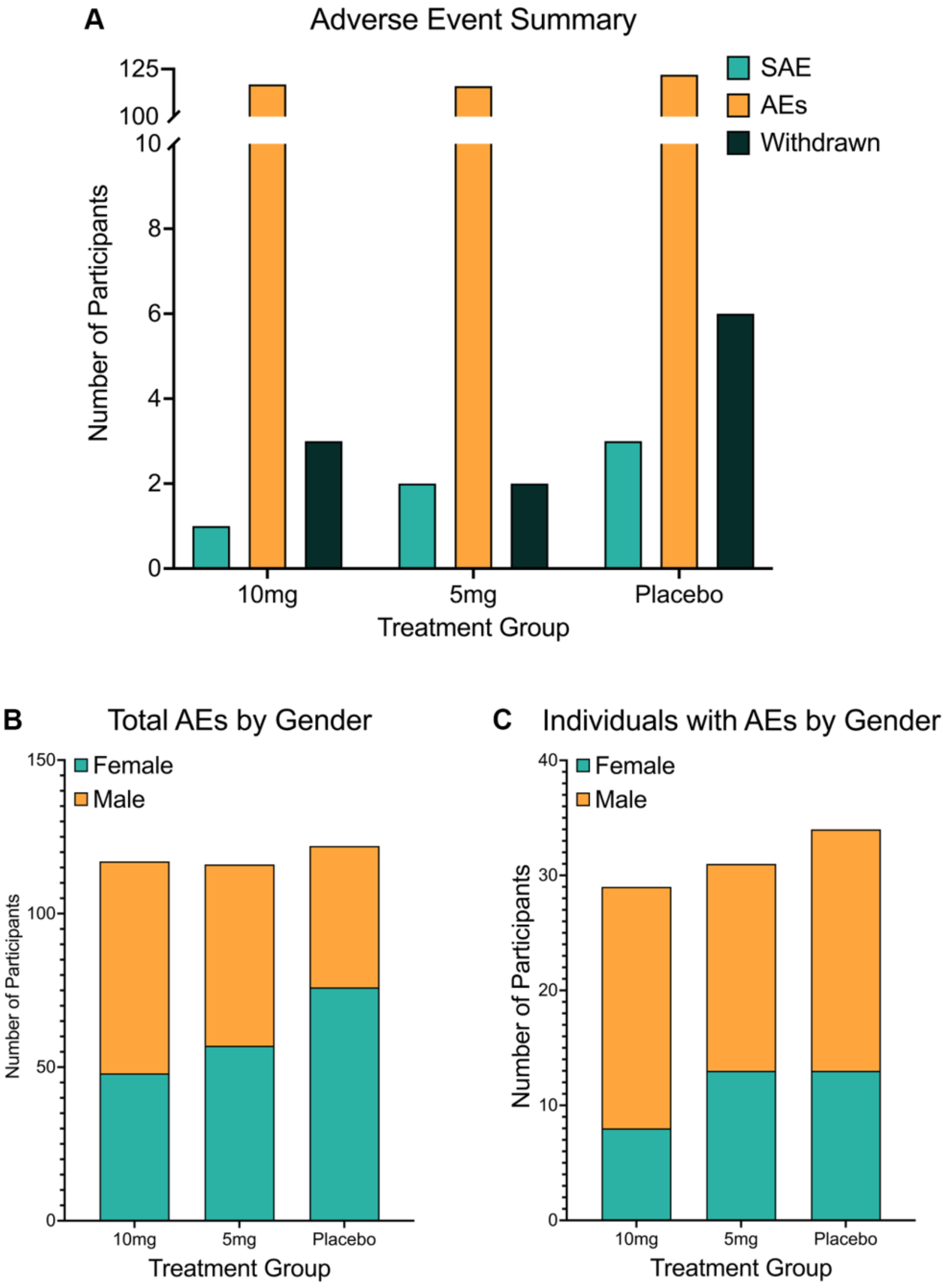

Supplementary Figure 2. Summary of adverse events and types for PEARL participants. Participants across all groups reported a similar number of incidences of adverse events, with the highest rates of serious adverse events in placebo users (**A**). Adverse event numbers were similar by gender for all groups (**B**), and in total number of participants experiencing adverse events in each group (**C**).

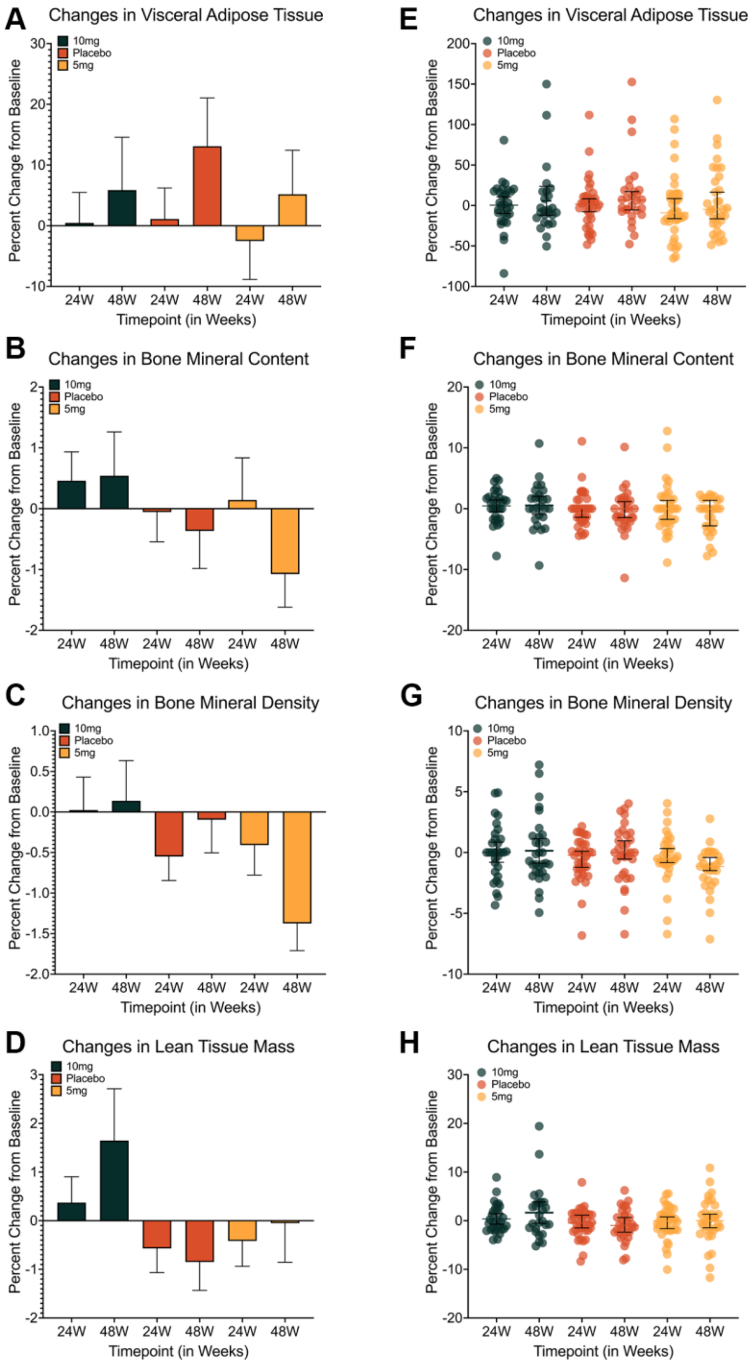

Supplementary Figure 3. Changes in body composition during the PEARL trial. Body composition measures did not change significantly over the course of the trial for groups as a whole, despite trending differences of improvement for means in some measures (**A**–**D**). However, individual changes during the study period were widely varied across all doses and groups (**E**–**H**). Error bars represent standard error of the mean.

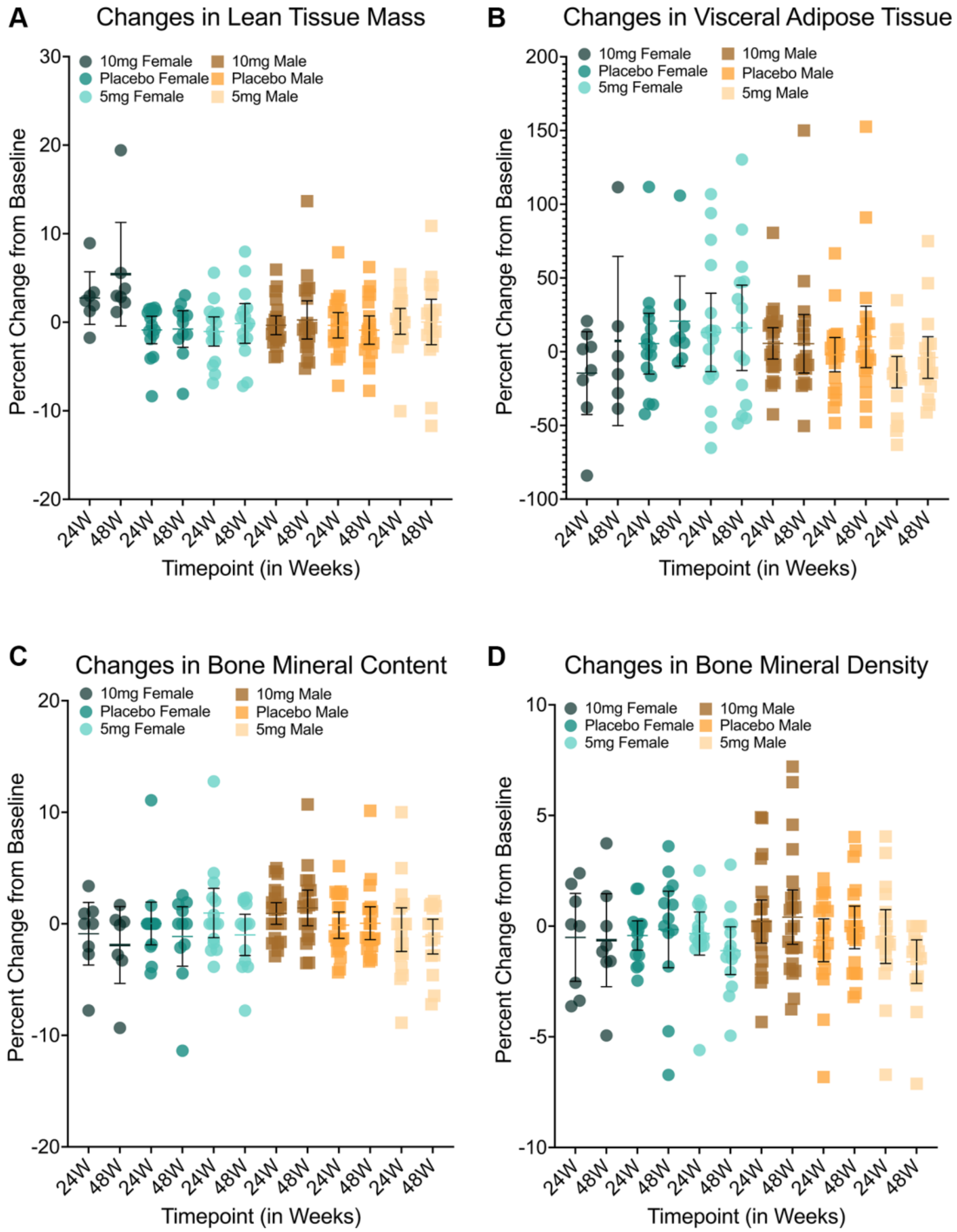

Supplementary Figure 4. Heterogeneity of individual response in body composition for each gender during the PEARL trial. Individual responses for measures of body composition change span a range of values for each dose and gender (**A**–**D**). Bars represent the 95% CI, dots represent individuals.
