# Supplementary material for: Influence of rapamycin on safety and healthspan metrics after one year: PEARL trial results
Source: Aging (Albany NY). 2025 Apr 4;17(4):908–36. doi: 10.18632/aging.206235 (PMC12074816; doi:10.18632/aging.206235)
Supplement: Supplementary Table 1 and 8 [file aging-17-206235-s003.pdf]

SUPPLEMENTARY TABLES

Supplementary Table 1. Demographic information for all study participants.

Treatment group	Gender	*N*	Percent *N* in group	Percent *N* overall	Category	Minimum	Maximum	Mean	Std. deviation
10 mg	F	8	22.86%	20.00%	Age (years)	51	76	58.75	8.17
					BMI	19.5	29.3	23.88	3.97
					Weight (kg)	49.9	85.59	65.81	10.42
					Height (in)	60	71	65.5	3.89
	M	27	77.14%	36.49%	Age	51	81	63.78	9.27
					BMI	22.1	34.6	26.13	2.93
					Weight	66.22	115.67	84.06	11.62
					Height	67	77	70.62	2.39
	Total	35	100.00%	30.70%	Age	51	81	62.63	9.17
					BMI	19.5	34.6	25.58	3.29
					Weight	49.9	115.67	79.77	13.68
					Height	60	77	69.38	3.55
Placebo	F	15	38.46%	37.50%	Age	55	71	62.33	4.89
					BMI	18.9	27.1	22.39	2.63
					Weight	49.9	73.94	62.32	7.78
					Height	62	69	65.31	2.15
	M	24	61.54%	32.43%	Age	52	78	62.83	7.57
					BMI	19.4	31.4	25.38	2.73
					Weight	61.23	105.64	82.67	12.01
					Height	67	77	70.93	2.49
	Total	39	100.00%	34.21%	Age	52	78	62.64	6.6
					BMI	18.9	31.4	24.3	3.03
					Weight	49.9	105.64	75.17	14.49
					Height	62	77	68.9	3.6
5 mg	F	17	42.50%	42.50%	Age	50	77	61.12	7.92
					BMI	18.5	30.7	23.53	3.4
					Weight	44.45	91.49	62.65	11.78
					Height	61	68	63.93	1.87
	M	23	57.50%	31.08%	Age	50	74	61.17	7.52
					BMI	21.5	36.5	26.33	3.11
					Weight	68.04	118.66	84.17	10.55
					Height	65	75	70.49	2.39
	Total	40	100.00%	35.09%	Age	50	77	61.15	7.59
					BMI	18.5	36.5	25.18	3.47
					Weight	44.45	118.66	75.56	15.26
					Height	61	75	67.68	3.93

Supplementary Table 8. Analysis of WOMAC self-reported measures of well-being.

Means and standard deviations

	Randomization group, source: demographics	All genders			Females			Males		
		Mean	Std. deviation	*N*	Mean	Std. deviation	*N*	Mean	Std. deviation	*N*
Total 0 weeks	10 mg	27.387	5.0045	31	29	6.6548	8	26.826	4.3343	23
	Placebo	29.7	8.8362	30	30.308	11.9957	13	29.235	5.7503	17
	5 mg	28.971	6.2352	34	29.133	5.6172	15	28.842	6.8334	19
Total 24 weeks	10 mg	26.871	6.4122	31	26.75	4.0267	8	26.913	7.1345	23
	Placebo	29.567	9.3244	30	31.846	12.3817	13	27.824	5.9291	17
	5 mg	29.529	12.3147	34	31.2	16.7298	15	28.211	7.495	19
Total 48 weeks	10 mg	28.161	6.3198	31	26	1.4142	8	28.913	7.179	23
	Placebo	28.567	7.3938	30	29.538	9.3239	13	27.824	5.7035	17
	5 mg	27.353	4.7026	34	26.8	2.8082	15	27.789	5.8269	19
Pain 0 weeks	10 mg	6.032	1.8526	31	6.625	2.9246	8	5.826	1.3366	23
	Placebo	6.1	1.7489	30	6.462	2.4364	13	5.824	0.951	17
	5 mg	6.529	2.0778	34	6.533	1.9223	15	6.526	2.2452	19
Pain 24 weeks	10 mg	5.839	1.8991	31	6.125	1.8851	8	5.739	1.9357	23
	Placebo	6.3	2.152	30	7	2.7988	13	5.765	1.3477	17
	5 mg	6.353	2.4727	34	6.933	3.1045	15	5.895	1.7918	19
Pain 48 weeks	10 mg	6.355	2.1377	31	5.875	1.126	8	6.522	2.3907	23
	Placebo	6.2	2.4551	30	6.923	3.4269	13	5.647	1.1695	17
	5 mg	5.912	1.4221	34	5.6	0.9103	15	6.158	1.7083	19
Stiffness 0 weeks	10 mg	2.774	0.956	31	3.25	1.165	8	2.609	0.8388	23
	Placebo	3.267	1.2847	30	3.231	1.5359	13	3.294	1.1048	17
	5 mg	3.118	1.2251	34	3.533	1.302	15	2.789	1.0842	19
Stiffness 24 weeks	10 mg	2.645	0.8774	31	2.875	0.991	8	2.565	0.8435	23
	Placebo	2.9	1.0939	30	2.923	1.1875	13	2.882	1.0537	17
	5 mg	2.912	1.4846	34	2.933	1.6676	15	2.895	1.3701	19
Stiffness 48 weeks	10 mg	2.742	0.8152	31	2.625	0.9161	8	2.783	0.7952	23
	Placebo	3	1.0828	30	2.846	1.2142	13	3.118	0.9926	17
	5 mg	2.706	1.0009	34	2.8	0.9411	15	2.632	1.0651	19
Physical Function 0 weeks	10 mg	18.581	3.5002	31	19.125	4.8532	8	18.391	3.0112	23
	Placebo	20.333	6.3209	30	20.615	8.3918	13	20.118	4.4142	17
	5 mg	19.324	3.7717	34	19.067	3.4115	15	19.526	4.1146	19
Physical Function 24 weeks	10 mg	18.387	4.0717	31	17.75	1.7525	8	18.609	4.6293	23
	Placebo	20.367	6.7389	30	21.923	9.0964	13	19.176	4.0963	17
	5 mg	20.265	8.8431	34	21.333	12.3269	15	19.421	4.8456	19
Physical Function 48 weeks	10 mg	19.065	4.082	31	17.5	1.069	8	19.609	4.5998	23
	Placebo	19.367	4.7233	30	19.769	5.3875	13	19.059	4.2935	17
	5 mg	18.735	2.8102	34	18.4	1.7238	15	19	3.4641	19

Repeated Measures Mixed ANOVA

	All genders					Females					Males				
**Measure**	**df 1**	**df 2**	**F**	***p*-value**	**Partial eta squared**	**df 1**	**df 2**	**F**	***P*-value**	**Partial Eta squared**	**df 1**	**df 2**	**F**	***p*-value**	**Partial Eta squared**
Total^	3.548	163.227	0.84	0.49	0.018	2.649	43.709	0.324	0.783	0.019	4	112	1.092	0.364	0.038
Pain	4	184	1.215	0.306	0.026	4	66	0.871	0.486	0.05	4	112	1.211	0.31	0.041
Stiffness	4	184	0.642	0.633	0.014	4	66	0.15	0.962	0.009	4	112	1.113	0.354	0.038
Physical Function^	3.422	157.426	0.714	0.563	0.015	2.527	41.695	0.308	0.786	0.018	3.617	101.264	0.911	0.453	0.031

Abbreviation: df: degrees of freedom. Provided as: between groups, within groups. ^denotes use of Welch's ANOVA in instances that lack homogeneity of variances. $^{*}p \leq 0.05$.
