# Supplementary material for: Influence of rapamycin on safety and healthspan metrics after one year: PEARL trial results
Source: Aging (Albany NY). 2025 Apr 4;17(4):908–36. doi: 10.18632/aging.206235 (PMC12074816; doi:10.18632/aging.206235)
Supplement: Supplementary Table 2-7 and 9 [file aging-17-206235-s004.docx]

| **Supplementary Table 2. Baseline Measurements for body composition, self-report surveys, and bloodwork** | | | | | | | | | | | | | | | | |
| --- | --- | --- | --- | --- | --- | --- | --- | --- | --- | --- | --- | --- | --- | --- | --- | --- |
|  |  |  |  |  |  |  |  |  |  |  |  |  |  |  |  |  |
| **ANOVA of Basline measurement differences** | | | | | | | | | | |  |  |  |  |  |  |
|  | Group | N | Mean | Std. Deviation | Std. Error | df | F | p-value | Effect Size**** | Group 1 | Group 2 | Mean Difference | Std Error | p-value | 95% Confidence Interval | |
|  |  |  |  |  |  |  |  |  |  |  |  |  |  |  | Lower Bound | Upper Bound |
| **SF-36** |  |  |  |  |  |  |  |  |  |  |  |  |  |  |  |  |
| Physical Functioning | 10mg | 36 | 95.694 | 8.7139 | 1.4523 | 2, 110 | 0.197 | 0.821 | -0.015 | 10mg | Placebo | 1.235 | 1.9991 | 1 | -3.625 | 6.095 |
|  |  |  |  |  |  |  |  |  |  |  | 5mg | 0.8194 | 1.9618 | 1 | -3.95 | 5.589 |
|  | Placebo | 37 | 94.459 | 9.4856 | 1.5594 |  |  |  |  | Placebo | 10mg | -1.235 | 1.9991 | 1 | -6.095 | 3.625 |
|  |  |  |  |  |  |  |  |  |  |  | 5mg | -0.4155 | 1.9478 | 1 | -5.151 | 4.32 |
|  | 5mg | 40 | 94.875 | 7.3805 | 1.167 |  |  |  |  | 5mg | 10mg | -0.8194 | 1.9618 | 1 | -5.589 | 3.95 |
|  |  |  |  |  |  |  |  |  |  |  | Placebo | 0.4155 | 1.9478 | 1 | -4.32 | 5.151 |
| Role limitations due to physical health* | 10mg | 36 | 95.139 | 11.6794 | 1.9466 | 2, 67.248 | 1.098 | 0.339 | 0.003 | 10mg | Placebo | 7.3011 | 4.9071 | 0.306 | -4.559 | 19.162 |
|  |  |  |  |  |  |  |  |  |  |  | 5mg | 1.3889 | 3.5218 | 0.918 | -7.054 | 9.832 |
|  | Placebo | 37 | 87.838 | 27.3998 | 4.5045 |  |  |  |  | Placebo | 10mg | -7.3011 | 4.9071 | 0.306 | -19.162 | 4.559 |
|  |  |  |  |  |  |  |  |  |  |  | 5mg | -5.9122 | 5.3763 | 0.518 | -18.819 | 6.994 |
|  | 5mg | 40 | 93.75 | 18.5621 | 2.9349 |  |  |  |  | 5mg | 10mg | -1.3889 | 3.5218 | 0.918 | -9.832 | 7.054 |
|  |  |  |  |  |  |  |  |  |  |  | Placebo | 5.9122 | 5.3763 | 0.518 | -6.994 | 18.819 |
| Role limitations due to emotional problems* | 10mg | 36 | 98.15 | 7.7359 | 1.2893 | 2, 60.793 | 4.103 | 0.021 | 0.017 | 10mg | Placebo | 5.3527 | 3.4484 | 0.276 | -2.991 | 13.696 |
|  |  |  |  |  |  |  |  |  |  |  | 5mg | 10.6525 | 4.1107 | 0.033 | 0.706 | 20.599 |
|  | Placebo | 37 | 92.797 | 19.4543 | 3.1983 |  |  |  |  | Placebo | 10mg | -5.3527 | 3.4484 | 0.276 | -13.696 | 2.991 |
|  |  |  |  |  |  |  |  |  |  |  | 5mg | 5.2998 | 5.0462 | 0.548 | -6.772 | 17.372 |
|  | 5mg | 40 | 87.498 | 24.6865 | 3.9033 |  |  |  |  | 5mg | 10mg | -10.6525 | 4.1107 | 0.033 | -20.599 | -0.706 |
|  |  |  |  |  |  |  |  |  |  |  | Placebo | -5.2998 | 5.0462 | 0.548 | -17.372 | 6.772 |
| Energy/Fatigue | 10mg | 36 | 71.528 | 13.4068 | 2.2345 | 2, 110 | 1.587 | 0.209 | 0.01 | 10mg | Placebo | 2.744 | 3.9147 | 1 | -6.773 | 12.261 |
|  |  |  |  |  |  |  |  |  |  |  | 5mg | 6.7778 | 3.8416 | 0.241 | -2.562 | 16.117 |
|  | Placebo | 37 | 68.784 | 17.2967 | 2.8436 |  |  |  |  | Placebo | 10mg | -2.744 | 3.9147 | 1 | -12.261 | 6.773 |
|  |  |  |  |  |  |  |  |  |  |  | 5mg | 4.0338 | 3.8142 | 0.878 | -5.239 | 13.307 |
|  | 5mg | 40 | 64.75 | 18.7408 | 2.9632 |  |  |  |  | 5mg | 10mg | -6.7778 | 3.8416 | 0.241 | -16.117 | 2.562 |
|  |  |  |  |  |  |  |  |  |  |  | Placebo | -4.0338 | 3.8142 | 0.878 | -13.307 | 5.239 |
| Emotional Wellbeing | 10mg | 36 | 83.667 | 10.226 | 1.7043 | 2, 110 | 4.083 | 0.019 | 0.052 | 10mg | Placebo | 2.4775 | 2.8071 | 1 | -4.347 | 9.302 |
|  |  |  |  |  |  |  |  |  |  |  | 5mg | 7.6667 | 2.7547 | 0.019 | 0.97 | 14.364 |
|  | Placebo | 37 | 81.189 | 10.8675 | 1.7866 |  |  |  |  | Placebo | 10mg | -2.4775 | 2.8071 | 1 | -9.302 | 4.347 |
|  |  |  |  |  |  |  |  |  |  |  | 5mg | 5.1892 | 2.735 | 0.181 | -1.46 | 11.838 |
|  | 5mg | 40 | 76 | 14.2361 | 2.2509 |  |  |  |  | 5mg | 10mg | -7.6667 | 2.7547 | 0.019 | -14.364 | -0.97 |
|  |  |  |  |  |  |  |  |  |  |  | Placebo | -5.1892 | 2.735 | 0.181 | -11.838 | 1.46 |
| Social Functioning | 10mg | 36 | 93.403 | 15.6656 | 2.6109 | 2, 110 | 0.11 | 0.896 | -0.016 | 10mg | Placebo | -0.5161 | 3.1544 | 1 | -8.185 | 7.153 |
|  |  |  |  |  |  |  |  |  |  |  | 5mg | 0.9028 | 3.0955 | 1 | -6.623 | 8.428 |
|  | Placebo | 37 | 93.919 | 10.874 | 1.7877 |  |  |  |  | Placebo | 10mg | 0.5161 | 3.1544 | 1 | -7.153 | 8.185 |
|  |  |  |  |  |  |  |  |  |  |  | 5mg | 1.4189 | 3.0734 | 1 | -6.053 | 8.891 |
|  | 5mg | 40 | 92.5 | 13.5164 | 2.1371 |  |  |  |  | 5mg | 10mg | -0.9028 | 3.0955 | 1 | -8.428 | 6.623 |
|  |  |  |  |  |  |  |  |  |  |  | Placebo | -1.4189 | 3.0734 | 1 | -8.891 | 6.053 |
| Pain | 10mg | 36 | 84.375 | 15.9841 | 2.664 | 2, 110 | 0.643 | 0.528 | -0.006 | 10mg | Placebo | 3.7669 | 3.6623 | 0.918 | -5.137 | 12.671 |
|  |  |  |  |  |  |  |  |  |  |  | 5mg | 0.4375 | 3.594 | 1 | -8.3 | 9.175 |
|  | Placebo | 37 | 80.608 | 17.9738 | 2.9549 |  |  |  |  | Placebo | 10mg | -3.7669 | 3.6623 | 0.918 | -12.671 | 5.137 |
|  |  |  |  |  |  |  |  |  |  |  | 5mg | -3.3294 | 3.5683 | 1 | -12.004 | 5.346 |
|  | 5mg | 40 | 83.938 | 12.7587 | 2.0173 |  |  |  |  | 5mg | 10mg | -0.4375 | 3.594 | 1 | -9.175 | 8.3 |
|  |  |  |  |  |  |  |  |  |  |  | Placebo | 3.3294 | 3.5683 | 1 | -5.346 | 12.004 |
| General Health | 10mg | 36 | 82.639 | 16.2343 | 2.7057 | 2, 110 | 1.618 | 0.203 | 0.011 | 10mg | Placebo | 6.4227 | 4.0881 | 0.357 | -3.516 | 16.361 |
|  |  |  |  |  |  |  |  |  |  |  | 5mg | 6.2639 | 4.0118 | 0.364 | -3.489 | 16.017 |
|  | Placebo | 37 | 76.216 | 19.1994 | 3.1564 |  |  |  |  | Placebo | 10mg | -6.4227 | 4.0881 | 0.357 | -16.361 | 3.516 |
|  |  |  |  |  |  |  |  |  |  |  | 5mg | -0.1588 | 3.9831 | 1 | -9.842 | 9.525 |
|  | 5mg | 40 | 76.375 | 16.832 | 2.6614 |  |  |  |  | 5mg | 10mg | -6.2639 | 4.0118 | 0.364 | -16.017 | 3.489 |
|  |  |  |  |  |  |  |  |  |  |  | Placebo | 0.1588 | 3.9831 | 1 | -9.525 | 9.842 |
|  |  |  |  |  |  |  |  |  |  |  |  |  |  |  |  |  |
|  | Group | N | Mean | Std. Deviation | Std. Error | df | F | p-value | Effect Size**** | Group 1 | Group 2 | Mean Difference | Std Error | p-value | 95% Confidence Interval | |
| **WOMAC** |  |  |  |  |  |  |  |  |  |  |  |  |  |  | Lower Bound | Upper Bound |
| Total Score | 10mg | 36 | 28.333 | 7.7754 | 1.2959 | 2, 110 | 1.135 | 0.325 | 0.002 | 10mg | Placebo | -2.6667 | 1.8955 | 0.487 | -7.275 | 1.942 |
|  |  |  |  |  |  |  |  |  |  |  | 5mg | -0.4917 | 1.8602 | 1 | -5.014 | 4.031 |
|  | Placebo | 37 | 31 | 10.0194 | 1.6472 |  |  |  |  | Placebo | 10mg | 2.6667 | 1.8955 | 0.487 | -1.942 | 7.275 |
|  |  |  |  |  |  |  |  |  |  |  | 5mg | 2.175 | 1.8469 | 0.724 | -2.315 | 6.665 |
|  | 5mg | 40 | 28.825 | 6.1639 | 0.9746 |  |  |  |  | 5mg | 10mg | 0.4917 | 1.8602 | 1 | -4.031 | 5.014 |
|  |  |  |  |  |  |  |  |  |  |  | Placebo | -2.175 | 1.8469 | 0.724 | -6.665 | 2.315 |
| Pain | 10mg | 36 | 6.25 | 2.4187 | 0.4031 | 2, 110 | 0.106 | 0.9 | -0.016 | 10mg | Placebo | -0.1014 | 0.5007 | 1 | -1.319 | 1.116 |
|  |  |  |  |  |  |  |  |  |  |  | 5mg | -0.225 | 0.4913 | 1 | -1.42 | 0.97 |
|  | Placebo | 37 | 6.351 | 1.9891 | 0.327 |  |  |  |  | Placebo | 10mg | 0.1014 | 0.5007 | 1 | -1.116 | 1.319 |
|  |  |  |  |  |  |  |  |  |  |  | 5mg | -0.1236 | 0.4878 | 1 | -1.31 | 1.062 |
|  | 5mg | 40 | 6.475 | 1.9998 | 0.3162 |  |  |  |  | 5mg | 10mg | 0.225 | 0.4913 | 1 | -0.97 | 1.42 |
|  |  |  |  |  |  |  |  |  |  |  | Placebo | 0.1236 | 0.4878 | 1 | -1.062 | 1.31 |
| Stiffness | 10mg | 36 | 2.861 | 1.0731 | 0.1789 | 2, 110 | 2.341 | 0.101 | 0.023 | 10mg | Placebo | -0.5983 | 0.2836 | 0.111 | -1.288 | 0.091 |
|  |  |  |  |  |  |  |  |  |  |  | 5mg | -0.1889 | 0.2783 | 1 | -0.865 | 0.488 |
|  | Placebo | 37 | 3.459 | 1.3457 | 0.2212 |  |  |  |  | Placebo | 10mg | 0.5983 | 0.2836 | 0.111 | -0.091 | 1.288 |
|  |  |  |  |  |  |  |  |  |  |  | 5mg | 0.4095 | 0.2763 | 0.424 | -0.262 | 1.081 |
|  | 5mg | 40 | 3.05 | 1.1972 | 0.1893 |  |  |  |  | 5mg | 10mg | 0.1889 | 0.2783 | 1 | -0.488 | 0.865 |
|  |  |  |  |  |  |  |  |  |  |  | Placebo | -0.4095 | 0.2763 | 0.424 | -1.081 | 0.262 |
| Physical Function | 10mg | 36 | 19.222 | 5.122 | 0.8537 | 2, 110 | 1.535 | 0.22 | 0.009 | 10mg | Placebo | -1.967 | 1.2846 | 0.386 | -5.09 | 1.156 |
|  |  |  |  |  |  |  |  |  |  |  | 5mg | -0.0778 | 1.2606 | 1 | -3.143 | 2.987 |
|  | Placebo | 37 | 21.189 | 7.1796 | 1.1803 |  |  |  |  | Placebo | 10mg | 1.967 | 1.2846 | 0.386 | -1.156 | 5.09 |
|  |  |  |  |  |  |  |  |  |  |  | 5mg | 1.8892 | 1.2516 | 0.402 | -1.154 | 4.932 |
|  | 5mg | 40 | 19.3 | 3.7155 | 0.5875 |  |  |  |  | 5mg | 10mg | 0.0778 | 1.2606 | 1 | -2.987 | 3.143 |
|  |  |  |  |  |  |  |  |  |  |  | Placebo | -1.8892 | 1.2516 | 0.402 | -4.932 | 1.154 |
|  |  |  |  |  |  |  |  |  |  |  |  |  |  |  |  |  |
|  | Group | N | Mean | Std. Deviation | Std. Error | df | F | p-value | Effect Size**** | Group 1 | Group 2 | Mean Difference | Std Error | p-value | 95% Confidence Interval | |
| **Bloodwork** |  |  |  |  |  |  |  |  |  |  |  |  |  |  | Lower Bound | Upper Bound |
| Total Cholesterol | 10mg | 36 | 187.75 | 44.0346 | 7.3391 | 2, 112 | 0.697 | 0.5 | -0.005 | 10mg | Placebo | -10.1987 | 9.5717 | 0.867 | -33.462 | 13.065 |
|  |  |  |  |  |  |  |  |  |  |  | 5mg | -1.175 | 9.5141 | 1 | -24.299 | 21.949 |
|  | Placebo | 39 | 197.949 | 40.3126 | 6.4552 |  |  |  |  | Placebo | 10mg | 10.1987 | 9.5717 | 0.867 | -13.065 | 33.462 |
|  |  |  |  |  |  |  |  |  |  |  | 5mg | 9.0237 | 9.3195 | 1 | -13.627 | 31.674 |
|  | 5mg | 40 | 188.925 | 40.0214 | 6.3279 |  |  |  |  | 5mg | 10mg | 1.175 | 9.5141 | 1 | -21.949 | 24.299 |
|  |  |  |  |  |  |  |  |  |  |  | Placebo | -9.0237 | 9.3195 | 1 | -31.674 | 13.627 |
| HDL Cholesterol | 10mg | 36 | 64.944 | 18.6593 | 3.1099 | 2, 112 | 0.105 | 0.9 | -0.016 | 10mg | Placebo | 1.2009 | 4.0353 | 1 | -8.607 | 11.009 |
|  |  |  |  |  |  |  |  |  |  |  | 5mg | 1.8194 | 4.0111 | 1 | -7.929 | 11.568 |
|  | Placebo | 39 | 63.744 | 17.4849 | 2.7998 |  |  |  |  | Placebo | 10mg | -1.2009 | 4.0353 | 1 | -11.009 | 8.607 |
|  |  |  |  |  |  |  |  |  |  |  | 5mg | 0.6186 | 3.929 | 1 | -8.931 | 10.168 |
|  | 5mg | 40 | 63.125 | 16.2815 | 2.5743 |  |  |  |  | 5mg | 10mg | -1.8194 | 4.0111 | 1 | -11.568 | 7.929 |
|  |  |  |  |  |  |  |  |  |  |  | Placebo | -0.6186 | 3.929 | 1 | -10.168 | 8.931 |
| Triglycerides | 10mg | 36 | 90.028 | 39.8264 | 6.6377 | 2, 112 | 0.28 | 0.756 | -0.013 | 10mg | Placebo | -2.7671 | 9.1658 | 1 | -25.044 | 19.51 |
|  |  |  |  |  |  |  |  |  |  |  | 5mg | 3.8778 | 9.1107 | 1 | -18.265 | 26.021 |
|  | Placebo | 39 | 92.795 | 45.7971 | 7.3334 |  |  |  |  | Placebo | 10mg | 2.7671 | 9.1658 | 1 | -19.51 | 25.044 |
|  |  |  |  |  |  |  |  |  |  |  | 5mg | 6.6449 | 8.9244 | 1 | -15.045 | 28.335 |
|  | 5mg | 40 | 86.15 | 32.3954 | 5.1222 |  |  |  |  | 5mg | 10mg | -3.8778 | 9.1107 | 1 | -26.021 | 18.265 |
|  |  |  |  |  |  |  |  |  |  |  | Placebo | -6.6449 | 8.9244 | 1 | -28.335 | 15.045 |
| LDL Cholesterol | 10mg | 36 | 104.261 | 39.5337 | 6.5889 | 2, 112 | 0.921 | 0.401 | -0.001 | 10mg | Placebo | -10.8415 | 8.1878 | 0.564 | -30.742 | 9.059 |
|  |  |  |  |  |  |  |  |  |  |  | 5mg | -3.5639 | 8.1385 | 1 | -23.344 | 16.216 |
|  | Placebo | 39 | 115.103 | 32.345 | 5.1793 |  |  |  |  | Placebo | 10mg | 10.8415 | 8.1878 | 0.564 | -9.059 | 30.742 |
|  |  |  |  |  |  |  |  |  |  |  | 5mg | 7.2776 | 7.972 | 1 | -12.098 | 26.653 |
|  | 5mg | 40 | 107.825 | 34.3809 | 5.4361 |  |  |  |  | 5mg | 10mg | 3.5639 | 8.1385 | 1 | -16.216 | 23.344 |
|  |  |  |  |  |  |  |  |  |  |  | Placebo | -7.2776 | 7.972 | 1 | -26.653 | 12.098 |
| CHOL/HDLC Ratio | 10mg | 36 | 3.092 | 0.9892 | 0.1649 | 2, 112 | 0.394 | 0.675 | -0.011 | 10mg | Placebo | -0.1545 | 0.1955 | 1 | -0.63 | 0.321 |
|  |  |  |  |  |  |  |  |  |  |  | 5mg | -0.0133 | 0.1943 | 1 | -0.486 | 0.459 |
|  | Placebo | 39 | 3.246 | 0.7843 | 0.1256 |  |  |  |  | Placebo | 10mg | 0.1545 | 0.1955 | 1 | -0.321 | 0.63 |
|  |  |  |  |  |  |  |  |  |  |  | 5mg | 0.1412 | 0.1903 | 1 | -0.321 | 0.604 |
|  | 5mg | 40 | 3.105 | 0.7595 | 0.1201 |  |  |  |  | 5mg | 10mg | 0.0133 | 0.1943 | 1 | -0.459 | 0.486 |
|  |  |  |  |  |  |  |  |  |  |  | Placebo | -0.1412 | 0.1903 | 1 | -0.604 | 0.321 |
| Non HDL Cholesterol | 10mg | 35 | 121.571 | 43.4513 | 7.3446 | 2, 111 | 1.683 | 0.191 | 0.012 | 10mg | Placebo | -12.6337 | 8.9454 | 0.482 | -34.378 | 9.111 |
|  |  |  |  |  |  |  |  |  |  |  | 5mg | 2.1714 | 8.8924 | 1 | -19.444 | 23.787 |
|  | Placebo | 39 | 134.205 | 34.4561 | 5.5174 |  |  |  |  | Placebo | 10mg | 12.6337 | 8.9454 | 0.482 | -9.111 | 34.378 |
|  |  |  |  |  |  |  |  |  |  |  | 5mg | 14.8051 | 8.6458 | 0.269 | -6.211 | 35.821 |
|  | 5mg | 40 | 119.4 | 37.3945 | 5.9126 |  |  |  |  | 5mg | 10mg | -2.1714 | 8.8924 | 1 | -23.787 | 19.444 |
|  |  |  |  |  |  |  |  |  |  |  | Placebo | -14.8051 | 8.6458 | 0.269 | -35.821 | 6.211 |
| VLDL Cholesterol | 10mg | 34 | 20.147 | 15.3645 | 2.635 | 2, 109 | 0.009 | 0.991 | -0.018 | 10mg | Placebo | 0.3576 | 3.5672 | 1 | -8.316 | 9.031 |
|  |  |  |  |  |  |  |  |  |  |  | 5mg | 0.4471 | 3.5248 | 1 | -8.123 | 9.018 |
|  | Placebo | 38 | 19.789 | 10.614 | 1.7218 |  |  |  |  | Placebo | 10mg | -0.3576 | 3.5672 | 1 | -9.031 | 8.316 |
|  |  |  |  |  |  |  |  |  |  |  | 5mg | 0.0895 | 3.4231 | 1 | -8.234 | 8.413 |
|  | 5mg | 40 | 19.7 | 18.2085 | 2.879 |  |  |  |  | 5mg | 10mg | -0.4471 | 3.5248 | 1 | -9.018 | 8.123 |
|  |  |  |  |  |  |  |  |  |  |  | Placebo | -0.0895 | 3.4231 | 1 | -8.413 | 8.234 |
| hsCRP | 10mg | 34 | 1.612 | 1.8646 | 0.3198 | 2, 105 | 0.755 | 0.473 | -0.005 | 10mg | Placebo | 0.5361 | 0.4363 | 0.666 | -0.525 | 1.598 |
|  |  |  |  |  |  |  |  |  |  |  | 5mg | 0.2793 | 0.4363 | 1 | -0.782 | 1.341 |
|  | Placebo | 37 | 1.076 | 1.3206 | 0.2171 |  |  |  |  | Placebo | 10mg | -0.5361 | 0.4363 | 0.666 | -1.598 | 0.525 |
|  |  |  |  |  |  |  |  |  |  |  | 5mg | -0.2568 | 0.427 | 1 | -1.296 | 0.782 |
|  | 5mg | 37 | 1.332 | 2.215 | 0.3641 |  |  |  |  | 5mg | 10mg | -0.2793 | 0.4363 | 1 | -1.341 | 0.782 |
|  |  |  |  |  |  |  |  |  |  |  | Placebo | 0.2568 | 0.427 | 1 | -0.782 | 1.296 |
| Glucose | 10mg | 36 | 92.139 | 8.842 | 1.4737 | 2, 112 | 0.16 | 0.852 | -0.015 | 10mg | Placebo | 0.6517 | 2.29 | 1 | -4.914 | 6.218 |
|  |  |  |  |  |  |  |  |  |  |  | 5mg | 1.2889 | 2.2762 | 1 | -4.243 | 6.821 |
|  | Placebo | 39 | 91.487 | 11.1045 | 1.7781 |  |  |  |  | Placebo | 10mg | -0.6517 | 2.29 | 1 | -6.218 | 4.914 |
|  |  |  |  |  |  |  |  |  |  |  | 5mg | 0.6372 | 2.2297 | 1 | -4.782 | 6.056 |
|  | 5mg | 40 | 90.85 | 9.5717 | 1.5134 |  |  |  |  | 5mg | 10mg | -1.2889 | 2.2762 | 1 | -6.821 | 4.243 |
|  |  |  |  |  |  |  |  |  |  |  | Placebo | -0.6372 | 2.2297 | 1 | -6.056 | 4.782 |
| BUN* | 10mg | 36 | 15.806 | 2.8866 | 0.4811 | 2, 72.432 | 0.587 | 0.558 | -0.004 | 10mg | Placebo | -0.5278 | 0.8496 | 0.809 | -2.565 | 1.509 |
|  |  |  |  |  |  |  |  |  |  |  | 5mg | -0.9444 | 0.9089 | 0.555 | -3.125 | 1.236 |
|  | Placebo | 39 | 16.333 | 4.373 | 0.7002 |  |  |  |  | Placebo | 10mg | 0.5278 | 0.8496 | 0.809 | -1.509 | 2.565 |
|  |  |  |  |  |  |  |  |  |  |  | 5mg | -0.4167 | 1.0416 | 0.916 | -2.906 | 2.073 |
|  | 5mg | 40 | 16.75 | 4.8767 | 0.7711 |  |  |  |  | 5mg | 10mg | 0.9444 | 0.9089 | 0.555 | -1.236 | 3.125 |
|  |  |  |  |  |  |  |  |  |  |  | Placebo | 0.4167 | 1.0416 | 0.916 | -2.073 | 2.906 |
| Creatinine* | 10mg | 36 | 0.948 | 0.1579 | 0.0263 | 2, 72.871 | 0.986 | 0.378 | 0.001 | 10mg | Placebo | -0.025 | 0.0504 | 0.873 | -0.146 | 0.096 |
|  |  |  |  |  |  |  |  |  |  |  | 5mg | 0.0436 | 0.0405 | 0.533 | -0.053 | 0.141 |
|  | Placebo | 39 | 0.973 | 0.2688 | 0.043 |  |  |  |  | Placebo | 10mg | 0.025 | 0.0504 | 0.873 | -0.096 | 0.146 |
|  |  |  |  |  |  |  |  |  |  |  | 5mg | 0.0686 | 0.0529 | 0.403 | -0.058 | 0.195 |
|  | 5mg | 40 | 0.905 | 0.195 | 0.0308 |  |  |  |  | 5mg | 10mg | -0.0436 | 0.0405 | 0.533 | -0.141 | 0.053 |
|  |  |  |  |  |  |  |  |  |  |  | Placebo | -0.0686 | 0.0529 | 0.403 | -0.195 | 0.058 |
| eGFR | 10mg | 36 | 83.008 | 10.7781 | 1.7964 | 2, 108 | 2.007 | 0.139 | 0.018 | 10mg | Placebo | 3.8768 | 3.1603 | 0.668 | -3.809 | 11.562 |
|  |  |  |  |  |  |  |  |  |  |  | 5mg | -2.343 | 3.181 | 1 | -10.079 | 5.393 |
|  | Placebo | 38 | 79.132 | 15.3168 | 2.4847 |  |  |  |  | Placebo | 10mg | -3.8768 | 3.1603 | 0.668 | -11.562 | 3.809 |
|  |  |  |  |  |  |  |  |  |  |  | 5mg | -6.2198 | 3.1383 | 0.15 | -13.852 | 1.412 |
|  | 5mg | 37 | 85.351 | 14.1367 | 2.3241 |  |  |  |  | 5mg | 10mg | 2.343 | 3.181 | 1 | -5.393 | 10.079 |
|  |  |  |  |  |  |  |  |  |  |  | Placebo | 6.2198 | 3.1383 | 0.15 | -1.412 | 13.852 |
| Sodium | 10mg | 36 | 139.861 | 1.4571 | 0.2428 | 2, 112 | 3.095 | 0.049 | 0.035 | 10mg | Placebo | 0.938 | 0.3992 | 0.062 | -0.032 | 1.908 |
|  |  |  |  |  |  |  |  |  |  |  | 5mg | 0.2111 | 0.3968 | 1 | -0.753 | 1.176 |
|  | Placebo | 39 | 138.923 | 2.0311 | 0.3252 |  |  |  |  | Placebo | 10mg | -0.938 | 0.3992 | 0.062 | -1.908 | 0.032 |
|  |  |  |  |  |  |  |  |  |  |  | 5mg | -0.7269 | 0.3887 | 0.192 | -1.672 | 0.218 |
|  | 5mg | 40 | 139.65 | 1.6259 | 0.2571 |  |  |  |  | 5mg | 10mg | -0.2111 | 0.3968 | 1 | -1.176 | 0.753 |
|  |  |  |  |  |  |  |  |  |  |  | Placebo | 0.7269 | 0.3887 | 0.192 | -0.218 | 1.672 |
| Potassium | 10mg | 36 | 4.369 | 0.2649 | 0.0442 | 2, 112 | 0.399 | 0.672 | -0.011 | 10mg | Placebo | -0.0177 | 0.0803 | 1 | -0.213 | 0.177 |
|  |  |  |  |  |  |  |  |  |  |  | 5mg | -0.0681 | 0.0798 | 1 | -0.262 | 0.126 |
|  | Placebo | 39 | 4.387 | 0.3147 | 0.0504 |  |  |  |  | Placebo | 10mg | 0.0177 | 0.0803 | 1 | -0.177 | 0.213 |
|  |  |  |  |  |  |  |  |  |  |  | 5mg | -0.0503 | 0.0782 | 1 | -0.24 | 0.14 |
|  | 5mg | 40 | 4.437 | 0.4325 | 0.0684 |  |  |  |  | 5mg | 10mg | 0.0681 | 0.0798 | 1 | -0.126 | 0.262 |
|  |  |  |  |  |  |  |  |  |  |  | Placebo | 0.0503 | 0.0782 | 1 | -0.14 | 0.24 |
| Chloride | 10mg | 36 | 103.972 | 1.5581 | 0.2597 | 2, 112 | 2.788 | 0.066 | 0.03 | 10mg | Placebo | 0.5107 | 0.4192 | 0.677 | -0.508 | 1.53 |
|  |  |  |  |  |  |  |  |  |  |  | 5mg | -0.4528 | 0.4167 | 0.839 | -1.465 | 0.56 |
|  | Placebo | 39 | 103.462 | 1.9173 | 0.307 |  |  |  |  | Placebo | 10mg | -0.5107 | 0.4192 | 0.677 | -1.53 | 0.508 |
|  |  |  |  |  |  |  |  |  |  |  | 5mg | -0.9635 | 0.4082 | 0.06 | -1.955 | 0.029 |
|  | 5mg | 40 | 104.425 | 1.92 | 0.3036 |  |  |  |  | 5mg | 10mg | 0.4528 | 0.4167 | 0.839 | -0.56 | 1.465 |
|  |  |  |  |  |  |  |  |  |  |  | Placebo | 0.9635 | 0.4082 | 0.06 | -0.029 | 1.955 |
| Carbon Dioxide | 10mg | 36 | 28.714 | 2.0504 | 0.3417 | 2, 112 | 1.133 | 0.326 | 0.002 | 10mg | Placebo | 0.7652 | 0.5105 | 0.41 | -0.476 | 2.006 |
|  |  |  |  |  |  |  |  |  |  |  | 5mg | 0.3389 | 0.5074 | 1 | -0.894 | 1.572 |
|  | Placebo | 39 | 27.949 | 2.2237 | 0.3561 |  |  |  |  | Placebo | 10mg | -0.7652 | 0.5105 | 0.41 | -2.006 | 0.476 |
|  |  |  |  |  |  |  |  |  |  |  | 5mg | -0.4263 | 0.4971 | 1 | -1.634 | 0.782 |
|  | 5mg | 40 | 28.375 | 2.3281 | 0.3681 |  |  |  |  | 5mg | 10mg | -0.3389 | 0.5074 | 1 | -1.572 | 0.894 |
|  |  |  |  |  |  |  |  |  |  |  | Placebo | 0.4263 | 0.4971 | 1 | -0.782 | 1.634 |
| Calcium | 10mg | 36 | 9.486 | 0.3217 | 0.0536 | 2, 112 | 1.492 | 0.229 | 0.009 | 10mg | Placebo | -0.019 | 0.08 | 1 | -0.214 | 0.175 |
|  |  |  |  |  |  |  |  |  |  |  | 5mg | 0.1061 | 0.0795 | 0.555 | -0.087 | 0.299 |
|  | Placebo | 39 | 9.505 | 0.3008 | 0.0482 |  |  |  |  | Placebo | 10mg | 0.019 | 0.08 | 1 | -0.175 | 0.214 |
|  |  |  |  |  |  |  |  |  |  |  | 5mg | 0.1251 | 0.0779 | 0.333 | -0.064 | 0.314 |
|  | 5mg | 40 | 9.38 | 0.404 | 0.0639 |  |  |  |  | 5mg | 10mg | -0.1061 | 0.0795 | 0.555 | -0.299 | 0.087 |
|  |  |  |  |  |  |  |  |  |  |  | Placebo | -0.1251 | 0.0779 | 0.333 | -0.314 | 0.064 |
| Total Protein* | 10mg | 36 | 6.781 | 0.4547 | 0.0758 | 2, 71.615 | 2.373 | 0.101 | 0.007 | 10mg | Placebo | -0.063 | 0.0904 | 0.766 | -0.28 | 0.154 |
|  |  |  |  |  |  |  |  |  |  |  | 5mg | 0.0956 | 0.0926 | 0.56 | -0.127 | 0.318 |
|  | Placebo | 39 | 6.844 | 0.3076 | 0.0493 |  |  |  |  | Placebo | 10mg | 0.063 | 0.0904 | 0.766 | -0.154 | 0.28 |
|  |  |  |  |  |  |  |  |  |  |  | 5mg | 0.1586 | 0.0726 | 0.08 | -0.015 | 0.332 |
|  | 5mg | 40 | 6.685 | 0.3371 | 0.0533 |  |  |  |  | 5mg | 10mg | -0.0956 | 0.0926 | 0.56 | -0.318 | 0.127 |
|  |  |  |  |  |  |  |  |  |  |  | Placebo | -0.1586 | 0.0726 | 0.08 | -0.332 | 0.015 |
| Albumin | 10mg | 36 | 4.406 | 0.2124 | 0.0354 | 2, 112 | 0.307 | 0.736 | -0.012 | 10mg | Placebo | -0.0175 | 0.0533 | 1 | -0.147 | 0.112 |
|  |  |  |  |  |  |  |  |  |  |  | 5mg | 0.0231 | 0.053 | 1 | -0.106 | 0.152 |
|  | Placebo | 39 | 4.423 | 0.2299 | 0.0368 |  |  |  |  | Placebo | 10mg | 0.0175 | 0.0533 | 1 | -0.112 | 0.147 |
|  |  |  |  |  |  |  |  |  |  |  | 5mg | 0.0406 | 0.0519 | 1 | -0.086 | 0.167 |
|  | 5mg | 40 | 4.383 | 0.2469 | 0.039 |  |  |  |  | 5mg | 10mg | -0.0231 | 0.053 | 1 | -0.152 | 0.106 |
|  |  |  |  |  |  |  |  |  |  |  | Placebo | -0.0406 | 0.0519 | 1 | -0.167 | 0.086 |
| Globulin | 10mg | 35 | 2.349 | 0.3633 | 0.0614 | 2, 111 | 2.487 | 0.088 | 0.026 | 10mg | Placebo | -0.1309 | 0.09 | 0.446 | -0.35 | 0.088 |
|  |  |  |  |  |  |  |  |  |  |  | 5mg | 0.0593 | 0.0895 | 1 | -0.158 | 0.277 |
|  | Placebo | 39 | 2.479 | 0.4508 | 0.0722 |  |  |  |  | Placebo | 10mg | 0.1309 | 0.09 | 0.446 | -0.088 | 0.35 |
|  |  |  |  |  |  |  |  |  |  |  | 5mg | 0.1902 | 0.087 | 0.093 | -0.021 | 0.402 |
|  | 5mg | 40 | 2.289 | 0.3352 | 0.053 |  |  |  |  | 5mg | 10mg | -0.0593 | 0.0895 | 1 | -0.277 | 0.158 |
|  |  |  |  |  |  |  |  |  |  |  | Placebo | -0.1902 | 0.087 | 0.093 | -0.402 | 0.021 |
| Albumin Globulin Ratio | 10mg | 35 | 1.92 | 0.3095 | 0.0523 | 2, 111 | 0.686 | 0.506 | -0.006 | 10mg | Placebo | Placebo | 0.0585 | 0.0699 | 1 | -0.111 |
|  |  |  |  |  |  |  |  |  |  |  | 5mg | 5mg | -0.0175 | 0.0695 | 1 | -0.186 |
|  | Placebo | 39 | 1.862 | 0.2935 | 0.047 |  |  |  |  | Placebo | 10mg | 10mg | -0.0585 | 0.0699 | 1 | -0.228 |
|  |  |  |  |  |  |  |  |  |  |  | 5mg | 5mg | -0.076 | 0.0676 | 0.79 | -0.24 |
|  | 5mg | 40 | 1.937 | 0.2984 | 0.0472 |  |  |  |  | 5mg | 10mg | 10mg | 0.0175 | 0.0695 | 1 | -0.151 |
|  |  |  |  |  |  |  |  |  |  |  | Placebo | Placebo | 0.076 | 0.0676 | 0.79 | -0.088 |
| Total Bilirubin | 10mg | 36 | 0.722 | 0.2542 | 0.0424 | 2, 112 | 0.007 | 0.993 | -0.018 | 10mg | Placebo | -0.0034 | 0.0716 | 1 | -0.177 | 0.17 |
|  |  |  |  |  |  |  |  |  |  |  | 5mg | 0.0047 | 0.0711 | 1 | -0.168 | 0.178 |
|  | Placebo | 39 | 0.726 | 0.3537 | 0.0566 |  |  |  |  | Placebo | 10mg | 0.0034 | 0.0716 | 1 | -0.17 | 0.177 |
|  |  |  |  |  |  |  |  |  |  |  | 5mg | 0.0081 | 0.0697 | 1 | -0.161 | 0.177 |
|  | 5mg | 40 | 0.718 | 0.3088 | 0.0488 |  |  |  |  | 5mg | 10mg | -0.0047 | 0.0711 | 1 | -0.178 | 0.168 |
|  |  |  |  |  |  |  |  |  |  |  | Placebo | -0.0081 | 0.0697 | 1 | -0.177 | 0.161 |
| Alkaline Phosphatase | 10mg | 36 | 61.667 | 16.7792 | 2.7965 | 2, 112 | 0.406 | 0.667 | -0.011 | 10mg | Placebo | 1.0513 | 4.5349 | 1 | -9.971 | 12.073 |
|  |  |  |  |  |  |  |  |  |  |  | 5mg | 3.8917 | 4.5076 | 1 | -7.064 | 14.847 |
|  | Placebo | 39 | 60.615 | 20.4934 | 3.2816 |  |  |  |  | Placebo | 10mg | -1.0513 | 4.5349 | 1 | -12.073 | 9.971 |
|  |  |  |  |  |  |  |  |  |  |  | 5mg | 2.8404 | 4.4154 | 1 | -7.891 | 13.572 |
|  | 5mg | 40 | 57.775 | 21.0646 | 3.3306 |  |  |  |  | 5mg | 10mg | -3.8917 | 4.5076 | 1 | -14.847 | 7.064 |
|  |  |  |  |  |  |  |  |  |  |  | Placebo | -2.8404 | 4.4154 | 1 | -13.572 | 7.891 |
| AST | 10mg | 36 | 23.361 | 5.2544 | 0.8757 | 2, 112 | 0.154 | 0.857 | -0.015 | 10mg | Placebo | 0.7714 | 1.496 | 1 | -2.865 | 4.407 |
|  |  |  |  |  |  |  |  |  |  |  | 5mg | 0.6611 | 1.487 | 1 | -2.953 | 4.275 |
|  | Placebo | 39 | 22.59 | 6.7345 | 1.0784 |  |  |  |  | Placebo | 10mg | -0.7714 | 1.496 | 1 | -4.407 | 2.865 |
|  |  |  |  |  |  |  |  |  |  |  | 5mg | -0.1103 | 1.4566 | 1 | -3.65 | 3.43 |
|  | 5mg | 40 | 22.7 | 7.1654 | 1.133 |  |  |  |  | 5mg | 10mg | -0.6611 | 1.487 | 1 | -4.275 | 2.953 |
|  |  |  |  |  |  |  |  |  |  |  | Placebo | 0.1103 | 1.4566 | 1 | -3.43 | 3.65 |
| ALT | 10mg | 36 | 23.389 | 8.9739 | 1.4956 | 2, 112 | 0.141 | 0.869 | -0.015 | 10mg | Placebo | 0.953 | 2.5598 | 1 | -5.269 | 7.175 |
|  |  |  |  |  |  |  |  |  |  |  | 5mg | 1.3139 | 2.5444 | 1 | -4.87 | 7.498 |
|  | Placebo | 39 | 22.436 | 12.9245 | 2.0696 |  |  |  |  | Placebo | 10mg | -0.953 | 2.5598 | 1 | -7.175 | 5.269 |
|  |  |  |  |  |  |  |  |  |  |  | 5mg | 0.3609 | 2.4924 | 1 | -5.697 | 6.419 |
|  | 5mg | 40 | 22.075 | 10.8282 | 1.7121 |  |  |  |  | 5mg | 10mg | -1.3139 | 2.5444 | 1 | -7.498 | 4.87 |
|  |  |  |  |  |  |  |  |  |  |  | Placebo | -0.3609 | 2.4924 | 1 | -6.419 | 5.697 |
| Uric Acid | 10mg | 34 | 5.447 | 1.1634 | 0.1995 | 2, 110 | 0.844 | 0.433 | -0.003 | 10mg | Placebo | 0.3701 | 0.2984 | 0.653 | -0.355 | 1.096 |
|  |  |  |  |  |  |  |  |  |  |  | 5mg | 0.2946 | 0.2967 | 0.969 | -0.427 | 1.016 |
|  | Placebo | 39 | 5.077 | 1.1748 | 0.1881 |  |  |  |  | Placebo | 10mg | -0.3701 | 0.2984 | 0.653 | -1.096 | 0.355 |
|  |  |  |  |  |  |  |  |  |  |  | 5mg | -0.0756 | 0.2862 | 1 | -0.771 | 0.62 |
|  | 5mg | 40 | 5.153 | 1.4397 | 0.2276 |  |  |  |  | 5mg | 10mg | -0.2946 | 0.2967 | 0.969 | -1.016 | 0.427 |
|  |  |  |  |  |  |  |  |  |  |  | Placebo | 0.0756 | 0.2862 | 1 | -0.62 | 0.771 |
| Insulin | 10mg | 35 | 6.126 | 4.2242 | 0.714 | 2, 110 | 0.643 | 0.528 | -0.006 | 10mg | Placebo | 1.0994 | 1.0084 | 0.834 | -1.352 | 3.551 |
|  |  |  |  |  |  |  |  |  |  |  | 5mg | 0.3082 | 0.9962 | 1 | -2.114 | 2.73 |
|  | Placebo | 38 | 5.026 | 3.1809 | 0.516 |  |  |  |  | Placebo | 10mg | -1.0994 | 1.0084 | 0.834 | -3.551 | 1.352 |
|  |  |  |  |  |  |  |  |  |  |  | 5mg | -0.7912 | 0.975 | 1 | -3.162 | 1.579 |
|  | 5mg | 40 | 5.818 | 5.2056 | 0.8231 |  |  |  |  | 5mg | 10mg | -0.3082 | 0.9962 | 1 | -2.73 | 2.114 |
|  |  |  |  |  |  |  |  |  |  |  | Placebo | 0.7912 | 0.975 | 1 | -1.579 | 3.162 |
| WBC | 10mg | 36 | 5.25 | 1.2358 | 0.206 | 2, 112 | 0.288 | 0.751 | -0.013 | 10mg | Placebo | -0.0192 | 0.2774 | 1 | -0.693 | 0.655 |
|  |  |  |  |  |  |  |  |  |  |  | 5mg | 0.1675 | 0.2757 | 1 | -0.503 | 0.838 |
|  | Placebo | 39 | 5.269 | 1.1804 | 0.189 |  |  |  |  | Placebo | 10mg | 0.0192 | 0.2774 | 1 | -0.655 | 0.693 |
|  |  |  |  |  |  |  |  |  |  |  | 5mg | 0.1867 | 0.2701 | 1 | -0.47 | 0.843 |
|  | 5mg | 40 | 5.083 | 1.1867 | 0.1876 |  |  |  |  | 5mg | 10mg | -0.1675 | 0.2757 | 1 | -0.838 | 0.503 |
|  |  |  |  |  |  |  |  |  |  |  | Placebo | -0.1867 | 0.2701 | 1 | -0.843 | 0.47 |
| RBC | 10mg | 36 | 4.724 | 0.3946 | 0.0658 | 2, 112 | 0.032 | 0.968 | -0.017 | 10mg | Placebo | -0.0261 | 0.1128 | 1 | -0.3 | 0.248 |
|  |  |  |  |  |  |  |  |  |  |  | 5mg | -0.0033 | 0.1121 | 1 | -0.276 | 0.269 |
|  | Placebo | 39 | 4.751 | 0.5414 | 0.0867 |  |  |  |  | Placebo | 10mg | 0.0261 | 0.1128 | 1 | -0.248 | 0.3 |
|  |  |  |  |  |  |  |  |  |  |  | 5mg | 0.0228 | 0.1098 | 1 | -0.244 | 0.29 |
|  | 5mg | 40 | 4.728 | 0.5082 | 0.0803 |  |  |  |  | 5mg | 10mg | 0.0033 | 0.1121 | 1 | -0.269 | 0.276 |
|  |  |  |  |  |  |  |  |  |  |  | Placebo | -0.0228 | 0.1098 | 1 | -0.29 | 0.244 |
| Hemoglobin | 10mg | 36 | 14.639 | 1.2227 | 0.2038 | 2, 112 | 0.165 | 0.848 | -0.015 | 10mg | Placebo | 0.085 | 0.3413 | 1 | -0.745 | 0.915 |
|  |  |  |  |  |  |  |  |  |  |  | 5mg | 0.1939 | 0.3393 | 1 | -0.631 | 1.018 |
|  | Placebo | 39 | 14.554 | 1.5816 | 0.2533 |  |  |  |  | Placebo | 10mg | -0.085 | 0.3413 | 1 | -0.915 | 0.745 |
|  |  |  |  |  |  |  |  |  |  |  | 5mg | 0.1088 | 0.3323 | 1 | -0.699 | 0.917 |
|  | 5mg | 40 | 14.445 | 1.5761 | 0.2492 |  |  |  |  | 5mg | 10mg | -0.1939 | 0.3393 | 1 | -1.018 | 0.631 |
|  |  |  |  |  |  |  |  |  |  |  | Placebo | -0.1088 | 0.3323 | 1 | -0.917 | 0.699 |
| Hematocrit | 10mg | 36 | 43.439 | 3.4641 | 0.5774 | 2, 112 | 0.124 | 0.883 | -0.016 | 10mg | Placebo | 0.0902 | 0.9519 | 1 | -2.223 | 2.404 |
|  |  |  |  |  |  |  |  |  |  |  | 5mg | 0.4414 | 0.9462 | 1 | -1.858 | 2.741 |
|  | Placebo | 39 | 43.349 | 4.53 | 0.7254 |  |  |  |  | Placebo | 10mg | -0.0902 | 0.9519 | 1 | -2.404 | 2.223 |
|  |  |  |  |  |  |  |  |  |  |  | 5mg | 0.3512 | 0.9268 | 1 | -1.901 | 2.604 |
|  | 5mg | 40 | 42.997 | 4.2367 | 0.6699 |  |  |  |  | 5mg | 10mg | -0.4414 | 0.9462 | 1 | -2.741 | 1.858 |
|  |  |  |  |  |  |  |  |  |  |  | Placebo | -0.3512 | 0.9268 | 1 | -2.604 | 1.901 |
| MCV | 10mg | 36 | 92.064 | 4.0512 | 0.6752 | 2, 112 | 0.574 | 0.565 | -0.008 | 10mg | Placebo | 0.6536 | 0.9312 | 1 | -1.61 | 2.917 |
|  |  |  |  |  |  |  |  |  |  |  | 5mg | 0.9789 | 0.9256 | 0.878 | -1.271 | 3.228 |
|  | Placebo | 39 | 91.41 | 3.8635 | 0.6187 |  |  |  |  | Placebo | 10mg | -0.6536 | 0.9312 | 1 | -2.917 | 1.61 |
|  |  |  |  |  |  |  |  |  |  |  | 5mg | 0.3253 | 0.9066 | 1 | -1.878 | 2.529 |
|  | 5mg | 40 | 91.085 | 4.1644 | 0.6584 |  |  |  |  | 5mg | 10mg | -0.9789 | 0.9256 | 0.878 | -3.228 | 1.271 |
|  |  |  |  |  |  |  |  |  |  |  | Placebo | -0.3253 | 0.9066 | 1 | -2.529 | 1.878 |
| MCH | 10mg | 36 | 31.039 | 1.6741 | 0.279 | 2, 112 | 0.728 | 0.485 | -0.005 | 10mg | Placebo | 0.3517 | 0.3858 | 1 | -0.586 | 1.289 |
|  |  |  |  |  |  |  |  |  |  |  | 5mg | 0.4414 | 0.3835 | 0.757 | -0.491 | 1.373 |
|  | Placebo | 39 | 30.687 | 1.532 | 0.2453 |  |  |  |  | Placebo | 10mg | -0.3517 | 0.3858 | 1 | -1.289 | 0.586 |
|  |  |  |  |  |  |  |  |  |  |  | 5mg | 0.0897 | 0.3756 | 1 | -0.823 | 1.003 |
|  | 5mg | 40 | 30.598 | 1.7888 | 0.2828 |  |  |  |  | 5mg | 10mg | -0.4414 | 0.3835 | 0.757 | -1.373 | 0.491 |
|  |  |  |  |  |  |  |  |  |  |  | Placebo | -0.0897 | 0.3756 | 1 | -1.003 | 0.823 |
| MCHC | 10mg | 36 | 33.703 | 0.8098 | 0.135 | 2, 112 | 0.307 | 0.736 | -0.012 | 10mg | Placebo | 0.1361 | 0.1954 | 1 | -0.339 | 0.611 |
|  |  |  |  |  |  |  |  |  |  |  | 5mg | 0.1303 | 0.1943 | 1 | -0.342 | 0.602 |
|  | Placebo | 39 | 33.567 | 0.8998 | 0.1441 |  |  |  |  | Placebo | 10mg | -0.1361 | 0.1954 | 1 | -0.611 | 0.339 |
|  |  |  |  |  |  |  |  |  |  |  | 5mg | -0.0058 | 0.1903 | 1 | -0.468 | 0.457 |
|  | 5mg | 40 | 33.573 | 0.8221 | 0.13 |  |  |  |  | 5mg | 10mg | -0.1303 | 0.1943 | 1 | -0.602 | 0.342 |
|  |  |  |  |  |  |  |  |  |  |  | Placebo | 0.0058 | 0.1903 | 1 | -0.457 | 0.468 |
| RDW | 10mg | 36 | 12.472 | 0.573 | 0.0955 | 2, 112 | 2.107 | 0.126 | 0.019 | 10mg | Placebo | -0.2714 | 0.1576 | 0.263 | -0.654 | 0.112 |
|  |  |  |  |  |  |  |  |  |  |  | 5mg | -0.2903 | 0.1566 | 0.199 | -0.671 | 0.09 |
|  | Placebo | 39 | 12.744 | 0.6431 | 0.103 |  |  |  |  | Placebo | 10mg | 0.2714 | 0.1576 | 0.263 | -0.112 | 0.654 |
|  |  |  |  |  |  |  |  |  |  |  | 5mg | -0.0189 | 0.1534 | 1 | -0.392 | 0.354 |
|  | 5mg | 40 | 12.763 | 0.7983 | 0.1262 |  |  |  |  | 5mg | 10mg | 0.2903 | 0.1566 | 0.199 | -0.09 | 0.671 |
|  |  |  |  |  |  |  |  |  |  |  | Placebo | 0.0189 | 0.1534 | 1 | -0.354 | 0.392 |
| Platelet Count | 10mg | 36 | 228.111 | 46.2896 | 7.7149 | 2, 112 | 0.179 | 0.837 | -0.015 | 10mg | Placebo | -7.6325 | 13.6681 | 1 | -40.852 | 25.587 |
|  |  |  |  |  |  |  |  |  |  |  | 5mg | -6.4389 | 13.5859 | 1 | -39.459 | 26.581 |
|  | Placebo | 39 | 235.744 | 70.9053 | 11.3539 |  |  |  |  | Placebo | 10mg | 7.6325 | 13.6681 | 1 | -25.587 | 40.852 |
|  |  |  |  |  |  |  |  |  |  |  | 5mg | 1.1936 | 13.308 | 1 | -31.151 | 33.538 |
|  | 5mg | 40 | 234.55 | 56.7599 | 8.9745 |  |  |  |  | 5mg | 10mg | 6.4389 | 13.5859 | 1 | -26.581 | 39.459 |
|  |  |  |  |  |  |  |  |  |  |  | Placebo | -1.1936 | 13.308 | 1 | -33.538 | 31.151 |
| MPV | 10mg | 35 | 10.409 | 0.9281 | 0.1569 | 2, 111 | 0.73 | 0.484 | -0.005 | 10mg | Placebo | -0.1889 | 0.1925 | 0.986 | -0.657 | 0.279 |
|  |  |  |  |  |  |  |  |  |  |  | 5mg | -0.2139 | 0.1913 | 0.798 | -0.679 | 0.251 |
|  | Placebo | 39 | 10.597 | 0.7314 | 0.1171 |  |  |  |  | Placebo | 10mg | 0.1889 | 0.1925 | 0.986 | -0.279 | 0.657 |
|  |  |  |  |  |  |  |  |  |  |  | 5mg | -0.0251 | 0.186 | 1 | -0.477 | 0.427 |
|  | 5mg | 40 | 10.622 | 0.8201 | 0.1297 |  |  |  |  | 5mg | 10mg | 0.2139 | 0.1913 | 0.798 | -0.251 | 0.679 |
|  |  |  |  |  |  |  |  |  |  |  | Placebo | 0.0251 | 0.186 | 1 | -0.427 | 0.477 |
| Absolute Neutrophils | 10mg | 35 | 2925.143 | 1050.9935 | 177.6503 | 2, 111 | 0.186 | 0.83 | -0.015 | 10mg | Placebo | 116.9121 | 225.4747 | 1 | -431.172 | 664.997 |
|  |  |  |  |  |  |  |  |  |  |  | 5mg | 0.4929 | 224.1377 | 1 | -544.342 | 545.327 |
|  | Placebo | 39 | 2808.231 | 833.4329 | 133.4561 |  |  |  |  | Placebo | 10mg | -116.9121 | 225.4747 | 1 | -664.997 | 431.172 |
|  |  |  |  |  |  |  |  |  |  |  | 5mg | -116.4192 | 217.9212 | 1 | -646.143 | 413.304 |
|  | 5mg | 40 | 2924.65 | 1014.5274 | 160.4109 |  |  |  |  | 5mg | 10mg | -0.4929 | 224.1377 | 1 | -545.327 | 544.342 |
|  |  |  |  |  |  |  |  |  |  |  | Placebo | 116.4192 | 217.9212 | 1 | -413.304 | 646.143 |
| Absolute Lymphocytes | 10mg | 35 | 1640.629 | 480.3342 | 81.1913 | 2, 111 | 2.347 | 0.1 | 0.023 | 10mg | Placebo | -97.7817 | 109.1381 | 1 | -363.075 | 167.512 |
|  |  |  |  |  |  |  |  |  |  |  | 5mg | 129.9286 | 108.491 | 0.701 | -133.792 | 393.649 |
|  | Placebo | 39 | 1738.41 | 495.8723 | 79.4031 |  |  |  |  | Placebo | 10mg | 97.7817 | 109.1381 | 1 | -167.512 | 363.075 |
|  |  |  |  |  |  |  |  |  |  |  | 5mg | 227.7103 | 105.482 | 0.099 | -28.696 | 484.116 |
|  | 5mg | 40 | 1510.7 | 429.661 | 67.9354 |  |  |  |  | 5mg | 10mg | -129.9286 | 108.491 | 0.701 | -393.649 | 133.792 |
|  |  |  |  |  |  |  |  |  |  |  | Placebo | -227.7103 | 105.482 | 0.099 | -484.116 | 28.696 |
| Absolute Monocytes | 10mg | 35 | 469.829 | 141.4514 | 23.9097 | 2, 111 | 0.165 | 0.848 | -0.015 | 10mg | Placebo | -13.3766 | 32.6598 | 1 | -92.766 | 66.013 |
|  |  |  |  |  |  |  |  |  |  |  | 5mg | 4.0536 | 32.4662 | 1 | -74.865 | 82.972 |
|  | Placebo | 39 | 483.205 | 155.8996 | 24.9639 |  |  |  |  | Placebo | 10mg | 13.3766 | 32.6598 | 1 | -66.013 | 92.766 |
|  |  |  |  |  |  |  |  |  |  |  | 5mg | 17.4301 | 31.5657 | 1 | -59.3 | 94.16 |
|  | 5mg | 40 | 465.775 | 121.9633 | 19.2841 |  |  |  |  | 5mg | 10mg | -4.0536 | 32.4662 | 1 | -82.972 | 74.865 |
|  |  |  |  |  |  |  |  |  |  |  | Placebo | -17.4301 | 31.5657 | 1 | -94.16 | 59.3 |
| Absolute Eosinophils | 10mg | 35 | 148.143 | 104.8071 | 17.7156 | 2, 111 | 1.71 | 0.186 | 0.012 | 10mg | Placebo | -46.6264 | 33.9465 | 0.517 | -129.144 | 35.891 |
|  |  |  |  |  |  |  |  |  |  |  | 5mg | 10.8679 | 33.7452 | 1 | -71.16 | 92.896 |
|  | Placebo | 39 | 194.769 | 210.4472 | 33.6985 |  |  |  |  | Placebo | 10mg | 46.6264 | 33.9465 | 0.517 | -35.891 | 129.144 |
|  |  |  |  |  |  |  |  |  |  |  | 5mg | 57.4942 | 32.8092 | 0.247 | -22.259 | 137.247 |
|  | 5mg | 40 | 137.275 | 88.1493 | 13.9376 |  |  |  |  | 5mg | 10mg | -10.8679 | 33.7452 | 1 | -92.896 | 71.16 |
|  |  |  |  |  |  |  |  |  |  |  | Placebo | -57.4942 | 32.8092 | 0.247 | -137.247 | 22.259 |
| Absolute Basophils | 10mg | 35 | 39.286 | 18.6336 | 3.1496 | 2, 111 | 1.079 | 0.343 | 0.001 | 10mg | Placebo | -5.5604 | 4.1182 | 0.539 | -15.571 | 4.45 |
|  |  |  |  |  |  |  |  |  |  |  | 5mg | -4.9393 | 4.0938 | 0.691 | -14.891 | 5.012 |
|  | Placebo | 39 | 44.846 | 17.1641 | 2.7485 |  |  |  |  | Placebo | 10mg | 5.5604 | 4.1182 | 0.539 | -4.45 | 15.571 |
|  |  |  |  |  |  |  |  |  |  |  | 5mg | 0.6212 | 3.9803 | 1 | -9.054 | 10.296 |
|  | 5mg | 40 | 44.225 | 17.339 | 2.7415 |  |  |  |  | 5mg | 10mg | 4.9393 | 4.0938 | 0.691 | -5.012 | 14.891 |
|  |  |  |  |  |  |  |  |  |  |  | Placebo | -0.6212 | 3.9803 | 1 | -10.296 | 9.054 |
| Neutrophils | 10mg | 36 | 55.364 | 9.7675 | 1.6279 | 2, 112 | 1.794 | 0.171 | 0.014 | 10mg | Placebo | 2.4254 | 2.0044 | 0.686 | -2.446 | 7.297 |
|  |  |  |  |  |  |  |  |  |  |  | 5mg | -1.2136 | 1.9923 | 1 | -6.056 | 3.629 |
|  | Placebo | 39 | 52.938 | 8.1428 | 1.3039 |  |  |  |  | Placebo | 10mg | -2.4254 | 2.0044 | 0.686 | -7.297 | 2.446 |
|  |  |  |  |  |  |  |  |  |  |  | 5mg | -3.639 | 1.9516 | 0.195 | -8.382 | 1.104 |
|  | 5mg | 40 | 56.578 | 8.1094 | 1.2822 |  |  |  |  | 5mg | 10mg | 1.2136 | 1.9923 | 1 | -3.629 | 6.056 |
|  |  |  |  |  |  |  |  |  |  |  | Placebo | 3.639 | 1.9516 | 0.195 | -1.104 | 8.382 |
| Lymphocytes | 10mg | 36 | 32.008 | 9.4318 | 1.572 | 2, 112 | 1.193 | 0.307 | 0.003 | 10mg | Placebo | -1.2301 | 1.8553 | 1 | -5.739 | 3.279 |
|  |  |  |  |  |  |  |  |  |  |  | 5mg | 1.5533 | 1.8441 | 1 | -2.929 | 6.035 |
|  | Placebo | 39 | 33.238 | 6.8011 | 1.089 |  |  |  |  | Placebo | 10mg | 1.2301 | 1.8553 | 1 | -3.279 | 5.739 |
|  |  |  |  |  |  |  |  |  |  |  | 5mg | 2.7835 | 1.8064 | 0.379 | -1.607 | 7.174 |
|  | 5mg | 40 | 30.455 | 7.7556 | 1.2263 |  |  |  |  | 5mg | 10mg | -1.5533 | 1.8441 | 1 | -6.035 | 2.929 |
|  |  |  |  |  |  |  |  |  |  |  | Placebo | -2.7835 | 1.8064 | 0.379 | -7.174 | 1.607 |
| Monocytes | 10mg | 35 | 9.143 | 2.3966 | 0.4051 | 2, 111 | 0.06 | 0.942 | -0.017 | 10mg | Placebo | -0.1059 | 0.5308 | 1 | -1.396 | 1.184 |
|  |  |  |  |  |  |  |  |  |  |  | 5mg | -0.1821 | 0.5277 | 1 | -1.465 | 1.101 |
|  | Placebo | 39 | 9.249 | 2.2502 | 0.3603 |  |  |  |  | Placebo | 10mg | 0.1059 | 0.5308 | 1 | -1.184 | 1.396 |
|  |  |  |  |  |  |  |  |  |  |  | 5mg | -0.0763 | 0.5131 | 1 | -1.323 | 1.171 |
|  | 5mg | 40 | 9.325 | 2.2029 | 0.3483 |  |  |  |  | 5mg | 10mg | 0.1821 | 0.5277 | 1 | -1.101 | 1.465 |
|  |  |  |  |  |  |  |  |  |  |  | Placebo | 0.0763 | 0.5131 | 1 | -1.171 | 1.323 |
| Eosinophils | 10mg | 35 | 2.86 | 1.8436 | 0.3116 | 2, 111 | 1.308 | 0.275 | 0.005 | 10mg | Placebo | -0.8451 | 0.6592 | 0.608 | -2.448 | 0.757 |
|  |  |  |  |  |  |  |  |  |  |  | 5mg | 0.1025 | 0.6553 | 1 | -1.49 | 1.695 |
|  | Placebo | 39 | 3.705 | 4.0413 | 0.6471 |  |  |  |  | Placebo | 10mg | 0.8451 | 0.6592 | 0.608 | -0.757 | 2.448 |
|  |  |  |  |  |  |  |  |  |  |  | 5mg | 0.9476 | 0.6372 | 0.419 | -0.601 | 2.496 |
|  | 5mg | 40 | 2.758 | 1.9849 | 0.3138 |  |  |  |  | 5mg | 10mg | -0.1025 | 0.6553 | 1 | -1.695 | 1.49 |
|  |  |  |  |  |  |  |  |  |  |  | Placebo | -0.9476 | 0.6372 | 0.419 | -2.496 | 0.601 |
| Basophils | 10mg | 35 | 0.794 | 0.4385 | 0.0741 | 2, 111 | 0.669 | 0.514 | -0.006 | 10mg | Placebo | -0.0749 | 0.0834 | 1 | -0.278 | 0.128 |
|  |  |  |  |  |  |  |  |  |  |  | 5mg | -0.0907 | 0.0829 | 0.829 | -0.292 | 0.111 |
|  | Placebo | 39 | 0.869 | 0.3221 | 0.0516 |  |  |  |  | Placebo | 10mg | 0.0749 | 0.0834 | 1 | -0.128 | 0.278 |
|  |  |  |  |  |  |  |  |  |  |  | 5mg | -0.0158 | 0.0806 | 1 | -0.212 | 0.18 |
|  | 5mg | 40 | 0.885 | 0.311 | 0.0492 |  |  |  |  | 5mg | 10mg | 0.0907 | 0.0829 | 0.829 | -0.111 | 0.292 |
|  |  |  |  |  |  |  |  |  |  |  | Placebo | 0.0158 | 0.0806 | 1 | -0.18 | 0.212 |
| DHEA Sulfate | 10mg | 36 | 123.75 | 107.1203 | 17.8534 | 2, 106 | 0.126 | 0.882 | -0.016 | 10mg | Placebo | -1.4662 | 24.812 | 1 | -61.823 | 58.891 |
|  |  |  |  |  |  |  |  |  |  |  | 5mg | 10.0278 | 24.9813 | 1 | -50.741 | 70.797 |
|  | Placebo | 37 | 125.216 | 119.8198 | 19.6983 |  |  |  |  | Placebo | 10mg | 1.4662 | 24.812 | 1 | -58.891 | 61.823 |
|  |  |  |  |  |  |  |  |  |  |  | 5mg | 11.494 | 24.812 | 1 | -48.863 | 71.851 |
|  | 5mg | 36 | 113.722 | 88.1977 | 14.6996 |  |  |  |  | 5mg | 10mg | -10.0278 | 24.9813 | 1 | -70.797 | 50.741 |
|  |  |  |  |  |  |  |  |  |  |  | Placebo | -11.494 | 24.812 | 1 | -71.851 | 48.863 |
| Vitamin D, 25-OH | 10mg | 35 | 49.2 | 15.7924 | 2.6694 | 2, 105 | 3.418 | 0.036 | 0.001 | 10mg | Placebo | -0.6378 | 4.5713 | 1 | -11.76 | 10.484 |
|  |  |  |  |  |  |  |  |  |  |  | 5mg | -5.9944 | 4.602 | 0.587 | -17.191 | 5.202 |
|  | Placebo | 37 | 49.838 | 18.3644 | 3.0191 |  |  |  |  | Placebo | 10mg | 0.6378 | 4.5713 | 1 | -10.484 | 11.76 |
|  |  |  |  |  |  |  |  |  |  |  | 5mg | -5.3566 | 4.5385 | 0.722 | -16.399 | 5.685 |
|  | 5mg | 36 | 55.194 | 23.2032 | 3.8672 |  |  |  |  | 5mg | 10mg | 5.9944 | 4.602 | 0.587 | -5.202 | 17.191 |
|  |  |  |  |  |  |  |  |  |  |  | Placebo | 5.3566 | 4.5385 | 0.722 | -5.685 | 16.399 |
| Hemoglobin A1C | 10mg | 35 | 5.266 | 0.2338 | 0.0395 | 2, 106 | 0.433 | 0.651 | 0.043 | 10mg | Placebo | -0.0787 | 0.0585 | 0.543 | -0.221 | 0.063 |
|  |  |  |  |  |  |  |  |  |  |  | 5mg | 0.071 | 0.0577 | 0.664 | -0.069 | 0.211 |
|  | Placebo | 36 | 5.344 | 0.2656 | 0.0443 |  |  |  |  | Placebo | 10mg | 0.0787 | 0.0585 | 0.543 | -0.063 | 0.221 |
|  |  |  |  |  |  |  |  |  |  |  | 5mg | 0.1497 | 0.0573 | 0.031 | 0.01 | 0.289 |
|  | 5mg | 38 | 5.195 | 0.2382 | 0.0386 |  |  |  |  | 5mg | 10mg | -0.071 | 0.0577 | 0.664 | -0.211 | 0.069 |
|  |  |  |  |  |  |  |  |  |  |  | Placebo | -0.1497 | 0.0573 | 0.031 | -0.289 | -0.01 |
| ApoB | 10mg | 14 | 90.929 | 30.4693 | 8.1433 | 2, 42 | 0.433 | 0.651 | -0.026 | 10mg | Placebo | -1.1891 | 9.1322 | 1 | -23.962 | 21.584 |
|  |  |  |  |  |  |  |  |  |  |  | 5mg | 6.8571 | 9.5639 | 1 | -16.992 | 30.706 |
|  | Placebo | 17 | 92.118 | 18.9172 | 4.5881 |  |  |  |  | Placebo | 10mg | 1.1891 | 9.1322 | 1 | -21.584 | 23.962 |
|  |  |  |  |  |  |  |  |  |  |  | 5mg | 8.0462 | 9.1322 | 1 | -14.726 | 30.819 |
|  | 5mg | 14 | 84.071 | 26.453 | 7.0699 |  |  |  |  | 5mg | 10mg | -6.8571 | 9.5639 | 1 | -30.706 | 16.992 |
|  |  |  |  |  |  |  |  |  |  |  | Placebo | -8.0462 | 9.1322 | 1 | -30.819 | 14.726 |
| HOMA_IR | 10mg | 35 | 1.4427 | 1.2107 | 0.20465 | 2, 110 | 0.489 | 0.615 | -0.009 | 10mg | Placebo | 0.25737 | 0.2616 | 0.982 | -0.3786 | 0.8933 |
|  |  |  |  |  |  |  |  |  |  |  | 5mg | 0.11309 | 0.25844 | 1 | -0.5152 | 0.7414 |
|  | Placebo | 38 | 1.1853 | 0.87611 | 0.14212 |  |  |  |  | Placebo | 10mg | -0.25737 | 0.2616 | 0.982 | -0.8933 | 0.3786 |
|  |  |  |  |  |  |  |  |  |  |  | 5mg | -0.14429 | 0.25294 | 1 | -0.7592 | 0.4707 |
|  | 5mg | 40 | 1.3296 | 1.22903 | 0.19433 |  |  |  |  | 5mg | 10mg | -0.11309 | 0.25844 | 1 | -0.7414 | 0.5152 |
|  |  |  |  |  |  |  |  |  |  |  | Placebo | 0.14429 | 0.25294 | 1 | -0.4707 | 0.7592 |
|  |  |  |  |  |  |  |  |  |  |  |  |  |  |  |  |  |
|  | Group | N | Mean | Std. Deviation | Std. Error | df | F | p-value | Effect Size**** | Group 1 | Group 2 | Mean Difference | Std Error | p-value | 95% Confidence Interval | |
| **Body Composition** |  |  |  |  |  |  |  |  |  |  |  |  |  |  | Lower Bound | Upper Bound |
| BMI | 10mg | 33 | 25.584 | 3.2926 | 0.5732 | 2, 100 | 1.407 | 0.25 | 0.085 | 10mg | Placebo | 1.2847 | 0.7867 | 0.317 | -0.631 | 3.2 |
|  |  |  |  |  |  |  |  |  |  |  | 5mg | 0.4081 | 0.7977 | 1 | -1.534 | 2.35 |
|  | Placebo | 36 | 24.3 | 3.0265 | 0.5044 |  |  |  |  | Placebo | 10mg | -1.2847 | 0.7867 | 0.317 | -3.2 | 0.631 |
|  |  |  |  |  |  |  |  |  |  |  | 5mg | -0.8767 | 0.7807 | 0.792 | -2.778 | 1.024 |
|  | 5mg | 34 | 25.176 | 3.4735 | 0.5957 |  |  |  |  | 5mg | 10mg | -0.4081 | 0.7977 | 1 | -2.35 | 1.534 |
|  |  |  |  |  |  |  |  |  |  |  | Placebo | 0.8767 | 0.7807 | 0.792 | -1.024 | 2.778 |
| Bone mineral density* | 10mg | 35 | 1.2765 | 0.14216 | 0.02403 | 2, 70.147 | 4.24 | 0.018 | 0.104 | 10mg | Placebo | 0.02769 | 0.03564 | 0.718 | -0.0576 | 0.113 |
|  |  |  |  |  |  |  |  |  |  |  | 5mg | 0.19684 | 0.06722 | 0.014 | 0.0345 | 0.3592 |
|  | Placebo | 39 | 1.2488 | 0.16437 | 0.02632 |  |  |  |  | Placebo | 10mg | -0.02769 | 0.03564 | 0.718 | -0.113 | 0.0576 |
|  |  |  |  |  |  |  |  |  |  |  | 5mg | 0.16915 | 0.06807 | 0.042 | 0.0049 | 0.3333 |
|  | 5mg | 40 | 1.0797 | 0.39703 | 0.06278 |  |  |  |  | 5mg | 10mg | -0.19684 | 0.06722 | 0.014 | -0.3592 | -0.0345 |
|  |  |  |  |  |  |  |  |  |  |  | Placebo | -0.16915 | 0.06807 | 0.042 | -0.3333 | -0.0049 |
| Weight | 10mg | 34 | 79769.4896 | 13679.22619 | 2345.96794 | 2, 104 | 1.079 | 0.344 | 0.07 | 10mg | Placebo | 4596.16488 | 3422.78596 | 0.547 | -3732.5686 | 12924.8983 |
|  |  |  |  |  |  |  |  |  |  |  | 5mg | 4204.95031 | 3491.36952 | 0.694 | -4290.6689 | 12700.5695 |
|  | Placebo | 38 | 75173.3247 | 14491.26015 | 2350.79282 |  |  |  |  | Placebo | 10mg | -4596.16488 | 3422.78596 | 0.547 | -12924.8983 | 3732.5686 |
|  |  |  |  |  |  |  |  |  |  |  | 5mg | -391.21457 | 3396.88122 | 1 | -8656.9135 | 7874.4843 |
|  | 5mg | 35 | 75564.5393 | 15261.23817 | 2579.62007 |  |  |  |  | 5mg | 10mg | -4204.95031 | 3491.36952 | 0.694 | -12700.5695 | 4290.6689 |
|  |  |  |  |  |  |  |  |  |  |  | Placebo | 391.21457 | 3396.88122 | 1 | -7874.4843 | 8656.9135 |
| Visceral adipose tissue | 10mg | 34 | 1016.1926 | 800.40424 | 137.2682 | 2, 110 | 0.356 | 0.701 | 0.032 | 10mg | Placebo | 149.65802 | 184.62671 | 1 | -299.1958 | 598.5119 |
|  |  |  |  |  |  |  |  |  |  |  | 5mg | 43.74853 | 183.54868 | 1 | -402.4845 | 489.9816 |
|  | Placebo | 39 | 866.5346 | 635.91562 | 101.82799 |  |  |  |  | Placebo | 10mg | -149.65802 | 184.62671 | 1 | -598.5119 | 299.1958 |
|  |  |  |  |  |  |  |  |  |  |  | 5mg | -105.90949 | 177.07453 | 1 | -536.4029 | 324.584 |
|  | 5mg | 40 | 972.4441 | 900.15031 | 142.32626 |  |  |  |  | 5mg | 10mg | -43.74853 | 183.54868 | 1 | -489.9816 | 402.4845 |
|  |  |  |  |  |  |  |  |  |  |  | Placebo | 105.90949 | 177.07453 | 1 | -324.584 | 536.4029 |
| Bone mineral content* | 10mg | 34 | 2901.5863 | 493.36821 | 84.61195 | 2, 68.931 | 1.405 | 0.252 | 0.039 | 10mg | Placebo | 71.20193 | 138.5845 | 0.865 | -260.9277 | 403.3315 |
|  |  |  |  |  |  |  |  |  |  |  | 5mg | 221.21757 | 131.89645 | 0.222 | -95.1103 | 537.5455 |
|  | Placebo | 38 | 2830.3844 | 676.58425 | 109.75646 |  |  |  |  | Placebo | 10mg | -71.20193 | 138.5845 | 0.865 | -403.3315 | 260.9277 |
|  |  |  |  |  |  |  |  |  |  |  | 5mg | 150.01563 | 149.27817 | 0.576 | -207.3428 | 507.3741 |
|  | 5mg | 35 | 2680.3688 | 598.59187 | 101.18049 |  |  |  |  | 5mg | 10mg | -221.21757 | 131.89645 | 0.222 | -537.5455 | 95.1103 |
|  |  |  |  |  |  |  |  |  |  |  | Placebo | -150.01563 | 149.27817 | 0.576 | -507.3741 | 207.3428 |
| Lean tissue mass | 10mg | 34 | 54715.5899 | 10518.42634 | 1803.89523 | 2, 109 | 1.561 | 0.215 | 0.084 | 10mg | Placebo | 2829.04751 | 2667.15082 | 0.874 | -3656.0994 | 9314.1944 |
|  |  |  |  |  |  |  |  |  |  |  | 5mg | 4697.13779 | 2667.15082 | 0.243 | -1788.0091 | 11182.2847 |
|  | Placebo | 39 | 51886.5424 | 12339.89213 | 1975.96415 |  |  |  |  | Placebo | 10mg | -2829.04751 | 2667.15082 | 0.874 | -9314.1944 | 3656.0994 |
|  |  |  |  |  |  |  |  |  |  |  | 5mg | 1868.09028 | 2574.19001 | 1 | -4391.0235 | 8127.204 |
|  | 5mg | 39 | 50018.4521 | 11058.60718 | 1770.79435 |  |  |  |  | 5mg | 10mg | -4697.13779 | 2667.15082 | 0.243 | -11182.2847 | 1788.0091 |
|  |  |  |  |  |  |  |  |  |  |  | Placebo | -1868.09028 | 2574.19001 | 1 | -8127.204 | 4391.0235 |
|  |  |  |  |  |  |  |  |  |  |  |  |  |  |  |  |  |
|  | Group | N | Mean | Std. Deviation | Std. Error | df | F | p-value | Effect Size**** | Group 1 | Group 2 | Mean Difference | Std Error | p-value | 95% Confidence Interval | |
| **Baseline Self-Reported Health Habits** |  |  |  |  |  |  |  |  |  |  |  |  |  |  | Lower Bound | Upper Bound |
| Vigorous Activity | 10mg | 35 | 2.91 | 1.422 | 0.24 | 2, 107 | 0.146 | 0.864 | 0.014 | 10mg | Placebo | -0.169 | 0.356 | 1 | -1.03 | 0.7 |
|  |  |  |  |  |  |  |  |  |  |  | 5mg | -0.163 | 0.349 | 1 | -1.01 | 0.69 |
|  | Placebo | 36 | 3.08 | 1.5 | 0.25 |  |  |  |  | Placebo | 10mg | 0.169 | 0.356 | 1 | -0.7 | 1.03 |
|  |  |  |  |  |  |  |  |  |  |  | 5mg | 0.006 | 0.346 | 1 | -0.84 | 0.85 |
|  | 5mg | 39 | 3.08 | 1.562 | 0.25 |  |  |  |  | 5mg | 10mg | 0.163 | 0.349 | 1 | -0.69 | 1.01 |
|  |  |  |  |  |  |  |  |  |  |  | Placebo | -0.006 | 0.346 | 1 | -0.85 | 0.84 |
| Moderate Activity | 10mg | 35 | 4.2 | 1.132 | 0.191 | 2, 65.313 | 5.315 | 0.007 | 0.076 | 10mg | Placebo | -0.522 | 0.217 | 0.051 | -1.05 | 0 |
|  |  |  |  |  |  |  |  |  |  |  | 5mg | 0.046 | 0.264 | 0.983 | -0.59 | 0.68 |
|  | Placebo | 36 | 4.72 | 0.615 | 0.102 |  |  |  |  | Placebo | 10mg | 0.522 | 0.217 | 0.051 | 0 | 1.05 |
|  |  |  |  |  |  |  |  |  |  |  | 5mg | 0.568 | 0.209 | 0.023 | 0.07 | 1.07 |
|  | 5mg | 39 | 4.15 | 1.136 | 0.182 |  |  |  |  | 5mg | 10mg | -0.046 | 0.264 | 0.983 | -0.68 | 0.59 |
|  |  |  |  |  |  |  |  |  |  |  | Placebo | -0.568 | 0.209 | 0.023 | -1.07 | -0.07 |
| Balanced Diet | 10mg | 35 | 4.26 | 0.852 | 0.144 | 2, 107 | 0.473 | 0.624 | 0.04 | 10mg | Placebo | -0.187 | 0.193 | 1 | -0.66 | 0.28 |
|  |  |  |  |  |  |  |  |  |  |  | 5mg | -0.102 | 0.189 | 1 | -0.56 | 0.36 |
|  | Placebo | 36 | 4.44 | 0.652 | 0.109 |  |  |  |  | Placebo | 10mg | 0.187 | 0.193 | 1 | -0.28 | 0.66 |
|  |  |  |  |  |  |  |  |  |  |  | 5mg | 0.085 | 0.188 | 1 | -0.37 | 0.54 |
|  | 5mg | 39 | 4.36 | 0.903 | 0.145 |  |  |  |  | 5mg | 10mg | 0.102 | 0.189 | 1 | -0.36 | 0.56 |
|  |  |  |  |  |  |  |  |  |  |  | Placebo | -0.085 | 0.188 | 1 | -0.54 | 0.37 |
| Excess Eating | 10mg | 35 | 3.86 | 1.033 | 0.175 | 2, 107 | 1.283 | 0.282 | 0.076 | 10mg | Placebo | -0.282 | 0.233 | 0.687 | -0.85 | 0.28 |
|  |  |  |  |  |  |  |  |  |  |  | 5mg | -0.348 | 0.228 | 0.392 | -0.9 | 0.21 |
|  | Placebo | 36 | 4.14 | 0.867 | 0.144 |  |  |  |  | Placebo | 10mg | 0.282 | 0.233 | 0.687 | -0.28 | 0.85 |
|  |  |  |  |  |  |  |  |  |  |  | 5mg | -0.066 | 0.227 | 1 | -0.62 | 0.49 |
|  | 5mg | 39 | 4.21 | 1.031 | 0.165 |  |  |  |  | 5mg | 10mg | 0.348 | 0.228 | 0.392 | -0.21 | 0.9 |
|  |  |  |  |  |  |  |  |  |  |  | Placebo | 0.066 | 0.227 | 1 | -0.49 | 0.62 |
| Goal Weight | 10mg | 35 | 3.86 | 1.332 | 0.225 | 2, 107 | 0.442 | 0.644 | 0.039 | 10mg | Placebo | -0.254 | 0.282 | 1 | -0.94 | 0.43 |
|  |  |  |  |  |  |  |  |  |  |  | 5mg | -0.066 | 0.276 | 1 | -0.74 | 0.61 |
|  | Placebo | 36 | 4.11 | 1.036 | 0.173 |  |  |  |  | Placebo | 10mg | 0.254 | 0.282 | 1 | -0.43 | 0.94 |
|  |  |  |  |  |  |  |  |  |  |  | 5mg | 0.188 | 0.274 | 1 | -0.48 | 0.85 |
|  | 5mg | 39 | 3.92 | 1.178 | 0.189 |  |  |  |  | 5mg | 10mg | 0.066 | 0.276 | 1 | -0.61 | 0.74 |
|  |  |  |  |  |  |  |  |  |  |  | Placebo | -0.188 | 0.274 | 1 | -0.85 | 0.48 |
| Smoking | 10mg | 35 | 4.89 | 0.471 | 0.08 | 2, 107 | 0.506 | 0.604 | 0.042 | 10mg | Placebo | 0.08 | 0.17 | 1 | -0.33 | 0.49 |
|  |  |  |  |  |  |  |  |  |  |  | 5mg | 0.168 | 0.167 | 0.953 | -0.24 | 0.57 |
|  | Placebo | 36 | 4.81 | 0.71 | 0.118 |  |  |  |  | Placebo | 10mg | -0.08 | 0.17 | 1 | -0.49 | 0.33 |
|  |  |  |  |  |  |  |  |  |  |  | 5mg | 0.088 | 0.166 | 1 | -0.32 | 0.49 |
|  | 5mg | 39 | 4.72 | 0.887 | 0.142 |  |  |  |  | 5mg | 10mg | -0.168 | 0.167 | 0.953 | -0.57 | 0.24 |
|  |  |  |  |  |  |  |  |  |  |  | Placebo | -0.088 | 0.166 | 1 | -0.49 | 0.32 |
| Caffeine | 10mg | 35 | 3.8 | 0.473 | 0.08 | 2, 107 | 0.123 | 0.885 | 0.011 | 10mg | Placebo | -0.033 | 0.133 | 1 | -0.36 | 0.29 |
|  |  |  |  |  |  |  |  |  |  |  | 5mg | 0.031 | 0.13 | 1 | -0.29 | 0.35 |
|  | Placebo | 36 | 3.83 | 0.507 | 0.085 |  |  |  |  | Placebo | 10mg | 0.033 | 0.133 | 1 | -0.29 | 0.36 |
|  |  |  |  |  |  |  |  |  |  |  | 5mg | 0.064 | 0.129 | 1 | -0.25 | 0.38 |
|  | 5mg | 39 | 3.77 | 0.667 | 0.107 |  |  |  |  | 5mg | 10mg | -0.031 | 0.13 | 1 | -0.35 | 0.29 |
|  |  |  |  |  |  |  |  |  |  |  | Placebo | -0.064 | 0.129 | 1 | -0.38 | 0.25 |
| Alcohol | 10mg | 35 | 4.74 | 0.657 | 0.111 | 2, 107 | 0.234 | 0.792 | 0.024 | 10mg | Placebo | 0.104 | 0.178 | 1 | -0.33 | 0.54 |
|  |  |  |  |  |  |  |  |  |  |  | 5mg | -0.001 | 0.175 | 1 | -0.43 | 0.42 |
|  | Placebo | 36 | 4.64 | 0.931 | 0.155 |  |  |  |  | Placebo | 10mg | -0.104 | 0.178 | 1 | -0.54 | 0.33 |
|  |  |  |  |  |  |  |  |  |  |  | 5mg | -0.105 | 0.174 | 1 | -0.53 | 0.32 |
|  | 5mg | 39 | 4.74 | 0.637 | 0.102 |  |  |  |  | 5mg | 10mg | 0.001 | 0.175 | 1 | -0.42 | 0.43 |
|  |  |  |  |  |  |  |  |  |  |  | Placebo | 0.105 | 0.174 | 1 | -0.32 | 0.53 |
| Drinking | 10mg | 35 | 1.34 | 1.798 | 0.304 | 2, 107 | 0.068 | 0.934 | -0.001 | 10mg | Placebo | 0.148 | 0.423 | 1 | -0.88 | 1.18 |
|  |  |  |  |  |  |  |  |  |  |  | 5mg | 0.035 | 0.415 | 1 | -0.97 | 1.04 |
|  | Placebo | 36 | 1.19 | 1.754 | 0.292 |  |  |  |  | Placebo | 10mg | -0.148 | 0.423 | 1 | -1.18 | 0.88 |
|  |  |  |  |  |  |  |  |  |  |  | 5mg | -0.113 | 0.412 | 1 | -1.12 | 0.89 |
|  | 5mg | 39 | 1.31 | 1.794 | 0.287 |  |  |  |  | 5mg | 10mg | -0.035 | 0.415 | 1 | -1.04 | 0.97 |
|  |  |  |  |  |  |  |  |  |  |  | Placebo | 0.113 | 0.412 | 1 | -0.89 | 1.12 |
| *df = degrees of freedom, provided as: between groups, within groups* | | | | | | | | | | | | | | | | |
| **denotes use of Welch's ANOVA in instances that lack homogeneity of variances* | | | | | | | | | | | | | | | | |
| ***effect size provided as epsilon squared for ANOVA or omega squared for Welch's ANOVA* | | | | | | | | | | | | | | | | |
| *post hoc tests were performed using the Bonferroni method (or Games-Howell for Welch's ANOVA)* | | | | | | | | | | | | | | | | |

| **Supplementary Table 3. Detail of adverse and serious adverse events for all trial participants** | | | | |  |  |  |  |
| --- | --- | --- | --- | --- | --- | --- | --- | --- |
| **Summary of withdrawn participants and serious adverse event instances** | | | | |  |  |  |  |
| **Treatment Group** | **Event Type** | **Withdrawn?** | **Withdraw Reason** | **Short Description** | **Symptoms Resolved?** |  |  |  |
| 10mg | AE, Withdrawn | Yes | AEs | general cold/flu with GI symptoms, acne, canker sore, and a second GI event | Yes |  |  |  |
| 10mg | Procedure | Yes | Procedure | Bladder stone requiring removal | Yes |  |  |  |
| 10mg | AE, Withdrawn | Yes | AEs | Long-COVID symptoms following COVID19. Discontinued to optimize resolution of symptoms | Unknown |  |  |  |
| 10mg | SAE | No | N/A | Severe sore throat, some cough and shortness of breath. Treated with Amoxicillin and OTC medications. | Yes |  |  |  |
| 5mg | Withdrawn | Yes | Changed Mind | Withrew with spouse | N/A |  |  |  |
| 5mg | AE, Withdrawn | Yes | AEs | Worsening of previous constipation issues | Yes |  |  |  |
| 5mg | SAE | No | N/A | One incident of anemia, shortness of breath. Given one unit of blood. | Yes |  |  |  |
| 5mg | SAE | No | N/A | Cold/Flu like illness with higher than normal severity, required treatment with Prednisone, Doxycycline hyclate, & Albuterol inhaler | Yes |  |  |  |
| Placebo | AE, Withdrawn | Yes | AEs | GI symptoms, fatigue | Yes |  |  |  |
| Placebo | AE, Withdrawn | Yes | AEs | COVID19, bruising, rash, slow healing, eye pain | Yes |  |  |  |
| Placebo | AE, Withdrawn | Yes | AEs | Neurological symptoms and worsening of preexisting symptoms | Unknown |  |  |  |
| Placebo | AE, Withdrawn | Yes | AEs | Worsening of pre-existing tinnitus, muscle and bone pain symptoms | Unknown |  |  |  |
| Placebo | Withdrawn | Yes | Other Health | Patient diagnosed with metastatic, small-cell neuroendocrine adenocarcinoma, their clinican believes to be unrelated to trial | Yes |  |  |  |
| Placebo | SAE | Yes | Deceased | Patient suffered myocardial infarction not believed to be related to the trial participation. | No |  |  |  |
| Placebo | SAE | No | N/A | Stomach virus, urinary infection. Prescribed Nitrofurantoin mono/mac Ondansetron ODT | Yes |  |  |  |
| Placebo | AE | No | N/A | Worsening of gluteal pain, discovered hamstring tear. Treated with platelet rich plasma and PT | Yes |  |  |  |
|  |  |  |  |  |  |  |  |  |
| **Detailed records of all adverse events reported across the study for all participants** | | | | | |  |  |  |
| **Group** | **Number of AEs** | **AE detail** | |  |  |  |  |  |
| 10mg | 16 | Upper GI Symptoms, Mouth Sores, Mouth Sores, Musculoskeletal Pain, Lower GI Symptoms, Eye Infection/Disorder, Menopausal Symptoms, Superficial Wound / Skin Irritation, Mouth Sores, Cold/Flu/Sinus Symptoms, Mouth Sores, Mouth Sores, Altered Spatial Orientation, Musculoskeletal Pain, Mouth Sores, Mouth Sores | | | | | | |
| 10mg | 2 | Mouth Sores, Cold/Flu/Sinus Symptoms | | | | | | |
| 10mg | 8 | Cold/Flu/Sinus Symptoms, Superficial Wound / Skin Irritation, Mouth Sores, Cold/Flu/Sinus Symptoms, Superficial Wound / Skin Irritation, Mouth Sores, Upper GI Symptoms, Upper GI Symptoms | | | | | | |
| 10mg | 1 | Musculoskeletal Pain | | | | | | |
| 10mg | 0 | None reported | | | | | | |
| 10mg | 4 | Neuromuscular Symptoms, Superficial Wound / Skin Irritation, Superficial Wound / Skin Irritation, Cold/Flu/Sinus Symptoms | | | | | | |
| 10mg | 9 | Cold/Flu/Sinus Symptoms, Mouth Sores, Auditory Perception Symptoms, Cephalalgia, Lower GI Symptoms, Lower GI Symptoms, Cephalalgia, Lower GI Symptoms, Lower GI Symptoms | | | | | | |
| 10mg | 3 | Upper and Lower GI Symptoms, Upper and Lower GI Symptoms, Cold/Flu/Sinus Symptoms | | | | | | |
| 10mg | 5 | Cold/Flu/Sinus Symptoms, Kidney/Urinary symptoms, Musculoskeletal Pain, Musculoskeletal Pain, Cold/Flu/Sinus Symptoms | | | | | | |
| 10mg | 1 | Cold/Flu/Sinus Symptoms | | | | | | |
| 10mg | 3 | Cold/Flu/Sinus Symptoms, Musculoskeletal Pain, Cold/Flu/Sinus Symptoms | | | | | | |
| 10mg | 0 | None reported | | | | | | |
| 10mg | 0 | None reported | | | | | | |
| 10mg | 2 | Cold/Flu/Sinus Symptoms, Cephalalgia | | | | | | |
| 10mg | 5 | Sleep Disturbances, Lower GI Symptoms, Erectile Dysfunction, Auditory Perception Symptoms | | | | | | |
| 10mg | 9 | Cold/Flu/Sinus Symptoms, Cold/Flu/Sinus Symptoms, Cold/Flu/Sinus Symptoms, Mouth Sores, Musculoskeletal Pain, Cold/Flu/Sinus Symptoms, Dental Symptoms, Cold/Flu/Sinus Symptoms, Musculoskeletal Pain | | | | | | |
| 10mg | 4 | Cold/Flu/Sinus Symptoms, Superficial Wound / Skin Irritation, Superficial Wound / Skin Irritation, Superficial Wound / Skin Irritation | | | | | | |
| 10mg | 5 | Cold/Flu/Sinus Symptoms, Altered Spatial Orientation, Cold/Flu/Sinus Symptoms, Altered Spatial Orientation, Pain (not otherwise specified) | | | | | | |
| 10mg | 4 | Musculoskeletal Pain, Musculoskeletal Pain, Cold/Flu/Sinus Symptoms, Musculoskeletal Pain | | | | | | |
| 10mg | 1 | Cold/Flu/Sinus Symptoms | | | | | | |
| 10mg | 2 | Upper GI Issues, Auditory Perception Issues | | | | | | |
| 10mg | 0 | None reported | | | | | | |
| 10mg | 1 | Malaise | | | | | | |
| 10mg | 1 | Musculoskeletal Pain | | | | | | |
| 10mg | 3 | Musculoskeletal Pain, Lower GI Symptoms, Visual Perception Symptoms | | | | | | |
| 10mg | 1 | Kidney/Urinary symptoms | | | | | | |
| 10mg | 3 | Cold/Flu/Sinus Symptoms, Lower GI Symptoms, Cold/Flu/Sinus Symptoms | | | | | | |
| 10mg | 1 | Cold/Flu/Sinus Symptoms | | | | | | |
| 10mg | 5 | Musculoskeletal Pain, Cold/Flu/Sinus Symptoms, Musculoskeletal Pain, Musculoskeletal Pain, Cold/Flu/Sinus Symptoms | | | | | | |
| 10mg | 0 | None reported | | | | | | |
| 10mg | 5 | Musculoskeletal Pain, Musculoskeletal Pain, Lower GI Symptoms, Mental Health Symptoms, Cold/Flu/Sinus Symptoms | | | | | | |
| 10mg | 0 | None reported | | | | | | |
| 10mg | 2 | Superficial Wound / Skin Irritation, Cognitive Decline | | | | | | |
| 10mg | 0 | None reported | | | | | | |
| 10mg | 9 | Musculoskeletal Pain, Malaise, Altered Spatial Orientation, Dental Symptoms, Mouth Sores, Altered Spatial Orientation, Altered Spatial Orientation, Altered Spatial Orientation, Mouth Sores | | | | | | |
| 10mg | 2 | Superficial Wound / Skin Irritation, Musculoskeletal Pain | | | | | | |
| Placebo | 1 | Cold/Flu/Sinus Symptoms | | | | | | |
| Placebo | 0 | None reported | | | | | | |
| Placebo | 2 | Auditory Perception Symptoms, Musculoskeletal Pain | | | | | | |
| Placebo | 5 | Menopausal Symptoms, Kidney/Urinary symptoms, Altered Spatial Orientation, Cold/Flu/Sinus Symptoms, Musculoskeletal Pain | | | | | | |
| Placebo | 2 | Cold/Flu/Sinus Symptoms, Cold/Flu/Sinus Symptoms | | | | | | |
| Placebo | 0 | None reported | | | | | | |
| Placebo | 1 | Cold/Flu/Sinus Symptoms | | | | | | |
| Placebo | 36 | Cold/Flu/Sinus Symptoms, Musculoskeletal Pain, Mouth Sores, Mouth Sores, Mouth Sores, Musculoskeletal Pain, Deep Tissue Injury (Closed), Mouth Sores, Musculoskeletal Pain, Skin Bacterial/Fungal Infection, Musculoskeletal Pain, Cold/Flu/Sinus Symptoms, Musculoskeletal Pain, Mouth Sores, Musculoskeletal Pain, Mouth Sores, Mouth Sores, Musculoskeletal Pain, Mouth Sores, Musculoskeletal Pain, Mouth Sores, Mouth Sores, Mouth Sores, Mouth Sores, Mouth Sores, Mouth Sores, Dental Symptoms, Mouth Sores, Musculoskeletal Pain, Mouth Sores, Mouth Sores, Musculoskeletal Pain, Lower GI Symptoms, Mouth Sores, Mouth Sores, Mouth Sores | | | | | | |
| Placebo | 3 | Cold/Flu/Sinus Symptoms, Cold/Flu/Sinus Symptoms, Musculoskeletal Pain | | | | | | |
| Placebo | 1 | Malaise | | | | | | |
| Placebo | 4 | Superficial Wound / Skin Irritation, Cold/Flu/Sinus Symptoms, Superficial Wound / Skin Irritation, Cold/Flu/Sinus Symptoms | | | | | | |
| Placebo | 3 | Skin Bacterial/Fungal Infection, Superficial Wound / Skin Irritation, Mental Health Symptoms | | | | | | |
| Placebo | 8 | Deep Wound (Opened), Menopausal Symptoms, Superficial Wound / Skin Irritation, Musculoskeletal Pain, Mouth Sores, Mouth Sores, Mouth Sores, Cold/Flu/Sinus Symptoms | | | | | | |
| Placebo | 5 | Cephalalgia, Cold/Flu/Sinus Symptoms, Mouth Sores, Cold/Flu/Sinus Symptoms, Cold/Flu/Sinus Symptoms | | | | | | |
| Placebo | 5 | Cephalalgia, Musculoskeletal Pain, Cold/Flu/Sinus Symptoms, Cold/Flu/Sinus Symptoms, Upper and Lower GI Symptoms | | | | | | |
| Placebo | 1 | Cold/Flu/Sinus Symptoms | | | | | | |
| Placebo | 1 | Musculoskeletal Pain | | | | | | |
| Placebo | 3 | Mouth Sores, Lower GI Symptoms, Neuromuscular Symptoms | | | | | | |
| Placebo | 1 | Cold/Flu/Sinus Symptoms | | | | | | |
| Placebo | 3 | Musculoskeletal Pain, Musculoskeletal/Orthopedic Injury (Closed), Superficial Wound / Skin Irritation | | | | | | |
| Placebo | 1 | Cold/Flu/Sinus Symptoms | | | | | | |
| Placebo | 1 | Lower GI Symptoms | | | | | | |
| Placebo | 4 | Cold/Flu/Sinus Symptoms, Cold/Flu/Sinus Symptoms, Superficial Wound / Skin Irritation, Superficial Wound / Skin Irritation | | | | | | |
| Placebo | 5 | Superficial Wound / Skin Irritation, Cold/Flu/Sinus Symptoms, Superficial Wound / Skin Irritation, Cold/Flu/Sinus Symptomss, Kidney/Urinary symptoms | | | | | | |
| Placebo | 1 | Pain (not otherwise specified) | | | | | | |
| Placebo | 3 | Superficial Wound / Skin Irritation, Cold/Flu/Sinus Symptoms, Neuromuscular Symptoms | | | | | | |
| Placebo | 3 | Deep Wound (Opened), Musculoskeletal Pain, Cold/Flu/Sinus Symptoms | | | | | | |
| Placebo | 2 | Mouth Sores, Cold/Flu/Sinus Symptoms | | | | | | |
| Placebo | 3 | Cold/Flu/Sinus Symptoms, Eye Infection/Disorder, Cold/Flu/Sinus Symptoms | | | | | | |
| Placebo | 0 | None reported | | | | | | |
| Placebo | 2 | Mouth Sores, Musculoskeletal Pain | | | | | | |
| Placebo | 2 | Superficial Wound / Skin Irritation, Musculoskeletal Pain | | | | | | |
| Placebo | 3 | Cold/Flu/Sinus Symptoms, Cardiological Event | | | | | | |
| Placebo | 0 | None reported | | | | | | |
| Placebo | 3 | Superficial Wound / Skin Irritation, Cold/Flu/Sinus Symptoms, Cold/Flu/Sinus Symptoms | | | | | | |
| Placebo | 0 | None reported | | | | | | |
| Placebo | 2 | Altered Spatial Orientation, Altered Spatial Orientation | | | | | | |
| Placebo | 1 | Cold/Flu/Sinus Symptoms | | | | | | |
| Placebo | 1 | Auditory Perception Symptoms | | | | | | |
| 5mg | 0 | None reported | | | | | | |
| 5mg | 4 | Kidney/Urinary symptoms, Musculoskeletal Pain, Cold/Flu/Sinus Symptoms, Skin Bacterial/Fungal Infection | | | | | | |
| 5mg | 2 | Mouth Sores, Cold/Flu/Sinus Symptoms | | | | | | |
| 5mg | 8 | Mouth Sores, Musculoskeletal Pain, Lower GI Symptoms, Musculoskeletal Pain, Cold/Flu/Sinus Symptoms, Musculoskeletal Pain | | | | | | |
| 5mg | 12 | Malaise, Malaise, Mouth Sores, Malaise, Malaise, Malaise, Mouth Sores, Cold/Flu/Sinus Symptoms, Malaise, Malaise, Malaise, Malaise | | | | | | |
| 5mg | 7 | Cold/Flu/Sinus Symptoms, Superficial Wound / Skin Irritation, Musculoskeletal Pain, Cephalalgia, Cold/Flu/Sinus Symptoms, Auditory Perception Symptoms, Superficial Wound / Skin Irritation | | | | | | |
| 5mg | 3 | Visual Perception Symptoms, Cold/Flu/Sinus Symptoms, Kidney/Urinary symptoms | | | | | | |
| 5mg | 4 | Lower GI Symptoms, Musculoskeletal Pain, Lower GI Symptoms, Pain (not otherwise specified) | | | | | | |
| 5mg | 8 | Cephalalgia, Superficial Wound / Skin Irritation, Menopausal Symptoms, Cold/Flu/Sinus Symptoms, Superficial Wound / Skin Irritation, Upper GI Symptoms, Cold/Flu/Sinus Symptoms, Altered Spatial Orientation | | | | | | |
| 5mg | 3 | Superficial Wound / Skin Irritation, Cold/Flu/Sinus Symptoms, Cold/Flu/Sinus Symptoms | | | | | | |
| 5mg | 1 | Cold/Flu/Sinus Symptoms | | | | | | |
| 5mg | 2 | Cold/Flu/Sinus Symptoms, Musculoskeletal Pain | | | | | | |
| 5mg | 0 | None reported | | | | | | |
| 5mg | 0 | None reported | | | | | | |
| 5mg | 0 | None reported | | | | | | |
| 5mg | 2 | Cold/Flu/Sinus Symptoms, Cold/Flu/Sinus Symptoms | | | | | | |
| 5mg | 1 | Superficial Wound / Skin Irritation | | | | | | |
| 5mg | 4 | Musculoskeletal Pain, Musculoskeletal Pain, Deep Wound (Opened), Respiratory Symptoms, Respiratory Symptoms | | | | | | |
| 5mg | 1 | Cold/Flu/Sinus Symptoms | | | | | | |
| 5mg | 5 | Upper GI Symptoms, Cold/Flu/Sinus Symptoms, Upper GI Symptoms, Upper GI Symptoms, Upper GI Symptoms | | | | | | |
| 5mg | 2 | Cold/Flu/Sinus Symptoms, Cold/Flu/Sinus Symptoms | | | | | | |
| 5mg | 0 | None reported | | | | | | |
| 5mg | 0 | None reported | | | | | | |
| 5mg | 1 | Cold/Flu/Sinus Symptoms | | | | | | |
| 5mg | 2 | Musculoskeletal Pain, Cold/Flu/Sinus Symptoms | | | | | | |
| 5mg | 1 | Cold/Flu/Sinus Symptoms | | | | | | |
| 5mg | 3 | Eye Infection/Disorder, Superficial Wound / Skin Irritation, Cold/Flu/Sinus Symptoms | | | | | | |
| 5mg | 8 | Musculoskeletal Pain, Musculoskeletal Pain, Musculoskeletal Pain, Musculoskeletal Pain, | | | | | | |
| 5mg | 2 | Musculoskeletal Pain, Dental Symptoms, Dental Symptoms, Musculoskeletal Pain | | | | | | |
| 5mg | 1 | Cold/Flu/Sinus Symptoms, Upper and Lower GI Symptoms | | | | | | |
| 5mg | 2 | Malaise | | | | | | |
| 5mg | 0 | Deep Tissue Injury (Closed), Deep Tissue Injury (Closed) | | | | | | |
| 5mg | 4 | None reported | | | | | | |
| 5mg | 6 | Cold/Flu/Sinus Symptoms, Dental Symptoms, Musculoskeletal Pain, Cold/Flu/Sinus Symptoms | | | | | | |
| 5mg | 0 | Upper and Lower GI Symptoms, Mental Health Symptoms, Musculoskeletal Pain, Kidney/Urinary symptoms, Musculoskeletal Pain, Malaise | | | | | | |
| 5mg | 2 | None reported | | | | | | |
| 5mg | 0 | Auditory Perception Symptoms, Cold/Flu/Sinus Symptoms | | | | | | |
| 5mg | 7 | None reported | | | | | | |
| 5mg | 1 | Discomfort/Tightness in Chest (not otherwise specified), Kidney/Urinary symptoms, Cold/Flu/Sinus Symptoms, Kidney/Urinary symptoms, Superficial Wound / Skin Irritation, Cold/Flu/Sinus Symptoms | | | | | | |
| 5mg | 7 | Lower GI Symptoms | | | | | | |

| **Supplementary Table 4. Analysis of body composition changes over time** | | | | | | |
| --- | --- | --- | --- | --- | --- | --- |
|  |  |  |  |  |  |  |
| **Means and Standard Deviations** | | |  |  |  |  |
| Group |  | N | Minimum | Maximum | Mean | Std. Deviation |
| 10mg | VAT Chg 0 to 24 - pct | 31 | -83.990 | 80.650 | 0.452 | 28.063 |
|  | BMD Chg 0 to 24 - pct | 35 | -100.000 | 4.920 | -11.407 | 32.358 |
|  | BMC Chg 0 to 24 - pct | 31 | -7.770 | 5.000 | 0.458 | 2.644 |
|  | LTM Chg 0 to 24 - pct | 30 | -3.960 | 8.920 | 0.371 | 2.913 |
|  | VAT Chg 0 to 48 - pct | 25 | -50.390 | 150.000 | 5.871 | 43.532 |
|  | BMD Chg 0 to 48 - pct | 35 | -100.000 | 7.210 | -11.305 | 32.429 |
|  | BMC Chg 0 to 48 - pct | 26 | -9.330 | 10.720 | 0.541 | 3.678 |
|  | LTM Chg 0 to 48 - pct | 26 | -5.230 | 19.420 | 1.649 | 5.425 |
|  |  |  |  |  |  |  |
| Placebo | VAT Chg 0 to 24 - pct | 36 | -48.330 | 111.760 | 1.109 | 30.687 |
|  | BMD Chg 0 to 24 - pct | 39 | -100.000 | 2.160 | -8.198 | 26.902 |
|  | BMC Chg 0 to 24 - pct | 36 | -4.440 | 11.080 | -0.054 | 2.946 |
|  | LTM Chg 0 to 24 - pct | 36 | -8.360 | 7.890 | -0.571 | 2.983 |
|  | VAT Chg 0 to 48 - pct | 31 | -47.700 | 152.630 | 13.105 | 41.097 |
|  | BMD Chg 0 to 48 - pct | 39 | -100.000 | 4.030 | -15.465 | 36.581 |
|  | BMC Chg 0 to 48 - pct | 31 | -11.380 | 10.140 | -0.365 | 3.445 |
|  | LTM Chg 0 to 48 - pct | 31 | -8.090 | 6.230 | -0.849 | 3.248 |
|  |  |  |  |  |  |  |
| 5mg | VAT Chg 0 to 24 - pct | 38 | -65.220 | 106.820 | -2.522 | 38.980 |
|  | BMD Chg 0 to 24 - pct | 36 | -100.000 | 87.500 | -3.498 | 27.962 |
|  | BMC Chg 0 to 24 - pct | 34 | -8.860 | 12.770 | 0.142 | 4.040 |
|  | LTM Chg 0 to 24 - pct | 38 | -10.050 | 5.590 | -0.416 | 3.210 |
|  | VAT Chg 0 to 48 - pct | 33 | -48.750 | 130.300 | 5.193 | 41.568 |
|  | BMD Chg 0 to 48 - pct | 36 | -100.000 | 100.000 | -8.502 | 37.336 |
|  | BMC Chg 0 to 48 - pct | 29 | -7.780 | 2.350 | -1.074 | 2.932 |
|  | LTM Chg 0 to 48 - pct | 33 | -11.710 | 10.870 | -0.050 | 4.613 |

| **Means and Standard Deviations by Gender** | | | | | | |
| --- | --- | --- | --- | --- | --- | --- |
| Group - Females | | N | Minimum | Maximum | Mean | Std. Deviation |
| 10mg | VAT Chg 0 to 24 - pct | 8 | -83.990 | 20.780 | -14.494 | 33.632 |
|  | BMD Chg 0 to 24 - pct | 8 | -3.620 | 2.390 | -0.508 | 2.377 |
|  | BMC Chg 0 to 24 - pct | 8 | -7.770 | 3.390 | -0.891 | 3.361 |
|  | LTM Chg 0 to 24 - pct | 7 | -1.760 | 8.920 | 2.725 | 3.212 |
|  | VAT Chg 0 to 48 - pct | 6 | -38.630 | 111.540 | 7.301 | 54.671 |
|  | BMD Chg 0 to 48 - pct | 8 | -4.940 | 3.740 | -0.632 | 2.513 |
|  | BMC Chg 0 to 48 - pct | 7 | -9.330 | 1.690 | -1.897 | 3.729 |
|  | LTM Chg 0 to 48 - pct | 7 | 1.150 | 19.420 | 5.425 | 6.322 |
|  |  |  |  |  |  |  |
| Placebo | VAT Chg 0 to 24 - pct | 15 | -42.310 | 111.760 | 5.465 | 37.267 |
|  | BMD Chg 0 to 24 - pct | 15 | -2.460 | 1.690 | -0.423 | 1.199 |
|  | BMC Chg 0 to 24 - pct | 15 | -4.440 | 11.080 | 0.035 | 3.460 |
|  | LTM Chg 0 to 24 - pct | 15 | -8.360 | 1.640 | -0.880 | 2.798 |
|  | VAT Chg 0 to 48 - pct | 11 | -22.390 | 105.880 | 18.693 | 35.169 |
|  | BMD Chg 0 to 48 - pct | 15 | -100.000 | 3.610 | -13.464 | 35.233 |
|  | BMC Chg 0 to 48 - pct | 11 | -11.380 | 2.560 | -1.128 | 3.974 |
|  | LTM Chg 0 to 48 - pct | 11 | -8.090 | 3.040 | -0.769 | 3.077 |
|  |  |  |  |  |  |  |
| 5mg | VAT Chg 0 to 24 - pct | 16 | -65.220 | 106.820 | 13.082 | 49.882 |
|  | BMD Chg 0 to 24 - pct | 15 | -5.600 | 2.510 | -0.332 | 1.758 |
|  | BMC Chg 0 to 24 - pct | 15 | -3.850 | 12.770 | 0.970 | 4.001 |
|  | LTM Chg 0 to 24 - pct | 17 | -6.880 | 5.590 | -1.050 | 3.210 |
|  | VAT Chg 0 to 48 - pct | 15 | -48.750 | 130.300 | 16.150 | 52.281 |
|  | BMD Chg 0 to 48 - pct | 15 | -100.000 | 2.780 | -7.702 | 25.597 |
|  | BMC Chg 0 to 48 - pct | 13 | -7.780 | 2.350 | -0.991 | 3.058 |
|  | LTM Chg 0 to 48 - pct | 15 | -7.210 | 7.970 | -0.139 | 4.051 |

| Group - Males |  | N | Minimum | Maximum | Mean | Std. Deviation |
| --- | --- | --- | --- | --- | --- | --- |
| 10mg | VAT Chg 0 to 24 - pct | 23 | -42.520 | 80.650 | 5.650 | 24.587 |
|  | BMD Chg 0 to 24 - pct | 27 | -100.000 | 4.920 | -14.637 | 36.336 |
|  | BMC Chg 0 to 24 - pct | 23 | -2.900 | 5.000 | 0.928 | 2.246 |
|  | LTM Chg 0 to 24 - pct | 23 | -3.960 | 5.930 | -0.346 | 2.464 |
|  | VAT Chg 0 to 48 - pct | 19 | -50.390 | 150.000 | 5.420 | 41.177 |
|  | BMD Chg 0 to 48 - pct | 27 | -100.000 | 7.210 | -14.467 | 36.443 |
|  | BMC Chg 0 to 48 - pct | 19 | -3.510 | 10.720 | 1.438 | 3.315 |
|  | LTM Chg 0 to 48 - pct | 19 | -5.230 | 13.670 | 0.258 | 4.469 |
|  |  |  |  |  |  |  |
| Placebo | VAT Chg 0 to 24 - pct | 21 | -48.330 | 66.670 | -2.003 | 25.521 |
|  | BMD Chg 0 to 24 - pct | 24 | -100.000 | 2.160 | -13.058 | 33.626 |
|  | BMC Chg 0 to 24 - pct | 21 | -4.350 | 5.170 | -0.118 | 2.607 |
|  | LTM Chg 0 to 24 - pct | 21 | -7.180 | 7.890 | -0.350 | 3.157 |
|  | VAT Chg 0 to 48 - pct | 20 | -47.700 | 152.630 | 10.031 | 44.584 |
|  | BMD Chg 0 to 48 - pct | 24 | -100.000 | 4.030 | -16.715 | 38.093 |
|  | BMC Chg 0 to 48 - pct | 20 | -3.370 | 10.140 | 0.055 | 3.148 |
|  | LTM Chg 0 to 48 - pct | 20 | -7.740 | 6.230 | -0.894 | 3.417 |
|  |  |  |  |  |  |  |
| 5mg | VAT Chg 0 to 24 - pct | 22 | -63.160 | 34.920 | -13.870 | 24.071 |
|  | BMD Chg 0 to 24 - pct | 21 | -100.000 | 87.500 | -5.760 | 36.787 |
|  | BMC Chg 0 to 24 - pct | 19 | -8.860 | 10.020 | -0.511 | 4.056 |
|  | LTM Chg 0 to 24 - pct | 21 | -10.050 | 5.420 | 0.097 | 3.194 |
|  | VAT Chg 0 to 48 - pct | 18 | -41.180 | 75.000 | -3.937 | 28.416 |
|  | BMD Chg 0 to 48 - pct | 21 | -100.000 | 100.000 | -9.073 | 44.497 |
|  | BMC Chg 0 to 48 - pct | 16 | -7.210 | 2.040 | -1.142 | 2.924 |
|  | LTM Chg 0 to 48 - pct | 18 | -11.710 | 10.870 | 0.024 | 5.150 |

| *df = degrees of freedom, provided as: between groups, within groups* |
| --- |
| **denotes use of Welch's ANOVA in instances that lack homogeneity of variances* |
| ***effect size provided as epsilon squared for ANOVA or omega squared for Welch's ANOVA* |
| *post hoc tests were performed using the Bonferroni method (or Games-Howell for Welch's ANOVA)* |

| **Repeated Measures Mixed ANOVA** | | | | |
| --- | --- | --- | --- | --- |
| All Genders | df | F | p-value | Partial Eta Squared |
| VAT | 2, 85 | 0.060 | 0.942 | 0.001 |
| BMD | 2, 107 | 0.573 | 0.565 | 0.011 |
| BMC | 2, 82 | 0.470 | 0.627 | 0.011 |
| LTM | 2, 85 | 0.751 | 0.475 | 0.017 |

| **Repeated Measures Mixed ANOVA by Gender** | | | | |
| --- | --- | --- | --- | --- |
| Females | df | F | p-value | Partial Eta Squared |
| VAT | 2, 29 | 0.462 | 0.635 | 0.031 |
| BMD | 2, 35 | 0.578 | 0.566 | 0.032 |
| BMC | 2, 28 | 0.010 | 0.990 | 0.001 |
| LTM | 2, 29 | 1.310 | 0.285 | 0.083 |

| Males | df | F | p-value | Partial Eta Squared |
| --- | --- | --- | --- | --- |
| VAT | 2, 53 | 0.370 | 0.692 | 0.014 |
| BMD | 2, 69 | 0.114 | 0.893 | 0.003 |
| BMC | 2, 51 | 0.485 | 0.619 | 0.019 |
| LTM | 2, 53 | 0.166 | 0.848 | 0.006 |

| **Repeated Measures ANOVA** | | | | | |
| --- | --- | --- | --- | --- | --- |
| Group - All Genders |  | df | F | p-value | Partial Eta Squared |
| 10mg | VAT | 1, 23 | 0.283 | 0.600 | 0.012 |
|  | BMD | 1, 34 | 0.001 | 0.981 | 0.000 |
|  | BMC | 1, 24 | 0.000 | 0.989 | 0.000 |
|  | LTM | 1, 23 | 0.983 | 0.332 | 0.041 |
| Placebo | VAT | 1, 30 | 2.108 | 0.157 | 0.066 |
|  | BMD | 1, 38 | 2.810 | 0.102 | 0.069 |
|  | BMC | 1, 30 | 0.223 | 0.640 | 0.007 |
|  | LTM | 1, 30 | 0.528 | 0.473 | 0.017 |
| 5mg | VAT | 1, 32 | 1.277 | 0.267 | 0.038 |
|  | BMD | 1, 35 | 0.656 | 0.424 | 0.018 |
|  | BMC | 1, 28 | 1.342 | 0.257 | 0.046 |
|  | LTM | 1, 32 | 0.180 | 0.674 | 0.006 |

| **Repeated Measures ANOVA by Gender** |  |  |  |  |  |
| --- | --- | --- | --- | --- | --- |
| Group - Females |  | df | F | p-value | Partial Eta Squared |
| 10mg | VAT | 1, 5 | 0.716 | 0.436 | 0.125 |
|  | BMD | 1, 7 | 0.023 | 0.884 | 0.003 |
|  | BMC | 1, 6 | 0.842 | 0.394 | 0.123 |
|  | LTM | 1, 5 | 2.013 | 0.215 | 0.287 |
| Placebo | VAT | 1, 10 | 0.754 | 0.406 | 0.070 |
|  | BMD | 1, 14 | 2.030 | 0.176 | 0.127 |
|  | BMC | 1, 10 | 0.384 | 0.549 | 0.037 |
|  | LTM | 1, 10 | 0.274 | 0.612 | 0.027 |
| 5mg | VAT | 1, 14 | 0.012 | 0.913 | 0.001 |
|  | BMD | 1, 14 | 1.252 | 0.282 | 0.082 |
|  | BMC | 1, 12 | 1.739 | 0.212 | 0.127 |
|  | LTM | 1, 14 | 0.461 | 0.508 | 0.032 |

| Group - Males |  | df | F | p-value | Partial Eta Squared |
| --- | --- | --- | --- | --- | --- |
| 10mg | VAT | 1, 17 | 0.004 | 0.949 | 0.000 |
|  | BMD | 1, 26 | 0.001 | 0.975 | 0.000 |
|  | BMC | 1, 17 | 0.192 | 0.667 | 0.011 |
|  | LTM | 1, 17 | 0.102 | 0.753 | 0.006 |
| Placebo | VAT | 1, 19 | 1.298 | 0.269 | 0.064 |
|  | BMD | 1, 23 | 0.782 | 0.386 | 0.033 |
|  | BMC | 1, 19 | 0.002 | 0.969 | 0.000 |
|  | LTM | 1, 19 | 0.302 | 0.589 | 0.016 |
| 5mg | VAT | 1, 17 | 2.624 | 0.124 | 0.134 |
|  | BMD | 1, 20 | 0.119 | 0.734 | 0.006 |
|  | BMC | 1, 15 | 0.497 | 0.492 | 0.032 |
|  | LTM | 1, 17 | 0.000 | 0.995 | 0.000 |

| **Paired T-Tests for changes between 24 and 48 weeks in each treatment group** | | | | | | | |
| --- | --- | --- | --- | --- | --- | --- | --- |
|  |  |  |  |  |  | 95% Confidence Interval | |
| Group - All Genders |  | t score | df | p value | Cohen's d | Lower | Upper |
| 10mg | VAT | -0.532 | 23 | 0.6 | -0.151 | -0.74 | 0.438 |
|  | BMD | 0.024 | 34 | 0.981 | 0.003 | -0.26 | 0.266 |
|  | BMC | -0.014 | 24 | 0.989 | -0.003 | -0.396 | 0.39 |
|  | LTM | -0.992 | 23 | 0.332 | -0.174 | -0.542 | 0.193 |
| Placebo | VAT | -1.452 | 30 | 0.157 | -0.253 | -0.616 | 0.109 |
|  | BMD | -1.676 | 38 | 0.102 | -0.216 | -0.482 | 0.05 |
|  | BMC | 0.472 | 30 | 0.64 | 0.089 | -0.295 | 0.473 |
|  | LTM | 0.727 | 30 | 0.473 | 0.138 | -0.252 | 0.528 |
| 5mg | VAT | -1.13 | 32 | 0.267 | -0.167 | -0.47 | 0.137 |
|  | BMD | -0.81 | 35 | 0.424 | -0.15 | -0.527 | 0.228 |
|  | BMC | 1.158 | 28 | 0.257 | 0.274 | -0.216 | 0.764 |
|  | LTM | -0.424 | 32 | 0.674 | -0.077 | -0.449 | 0.295 |

| **Paired T-Tests for changes between 24 and 48 weeks in each treatment group by Gender** | | | | | | | |
| --- | --- | --- | --- | --- | --- | --- | --- |
|  |  |  |  |  |  | 95% Confidence Interval | |
| Group - Females | | t score | df | p value | Cohen's d | Lower | Upper |
| 10mg | VAT | -0.846 | 5 | 0.436 | -0.435 | -1.804 | 0.933 |
|  | BMD | -0.151 | 7 | 0.884 | -0.051 | -0.846 | 0.745 |
|  | BMC | 0.917 | 6 | 0.394 | 0.239 | -0.421 | 0.899 |
|  | LTM | -1.419 | 5 | 0.215 | -0.322 | -0.962 | 0.318 |
| Placebo | VAT | -0.868 | 10 | 0.406 | -0.227 | -0.822 | 0.367 |
|  | BMD | -1.425 | 14 | 0.176 | -0.562 | -1.438 | 0.314 |
|  | BMC | 0.62 | 10 | 0.549 | 0.217 | -0.572 | 1.007 |
|  | LTM | 0.524 | 10 | 0.612 | 0.128 | -0.421 | 0.678 |
| 5mg | VAT | -0.111 | 14 | 0.913 | -0.022 | -0.454 | 0.409 |
|  | BMD | -1.119 | 14 | 0.282 | -0.392 | -1.159 | 0.376 |
|  | BMC | 1.319 | 12 | 0.212 | 0.301 | -0.214 | 0.816 |
|  | LTM | -0.679 | 14 | 0.508 | -0.184 | -0.769 | 0.401 |

|  |  |  |  |  |  | 95% Confidence Interval | |
| --- | --- | --- | --- | --- | --- | --- | --- |
| Group - Males | | t score | df | p value | Cohen's d | Lower | Upper |
| 10mg | VAT | -0.065 | 17 | 0.949 | -0.022 | -0.744 | 0.7 |
|  | BMD | 0.031 | 26 | 0.975 | 0.005 | -0.303 | 0.313 |
|  | BMC | -0.438 | 17 | 0.667 | -0.12 | -0.697 | 0.458 |
|  | LTM | -0.32 | 17 | 0.753 | -0.085 | -0.646 | 0.476 |
| Placebo | VAT | -1.139 | 19 | 0.269 | -0.25 | -0.717 | 0.217 |
|  | BMD | -0.885 | 23 | 0.386 | -0.1 | -0.335 | 0.135 |
|  | BMC | 0.04 | 19 | 0.969 | 0.009 | -0.446 | 0.464 |
|  | LTM | 0.55 | 19 | 0.589 | 0.138 | -0.388 | 0.663 |
| 5mg | VAT | -1.62 | 17 | 0.124 | -0.435 | -1.024 | 0.153 |
|  | BMD | -0.345 | 20 | 0.734 | -0.081 | -0.57 | 0.408 |
|  | BMC | 0.705 | 15 | 0.492 | 0.249 | -0.511 | 1.009 |
|  | LTM | -0.006 | 17 | 0.995 | -0.002 | -0.517 | 0.514 |

| **One-way ANOVA for 24 weeks** | | | | | | | | |  | 95% Confidence Interval | |
| --- | --- | --- | --- | --- | --- | --- | --- | --- | --- | --- | --- |
| All Genders | df | F | p value | Effect Size** | Group 1 | Group 2 | Mean Difference | Std Error | p-value | Lower Bound | Upper Bound |
| VAT | 2, 102 | 0.125 | 0.883 | -0.017 | 10mg | Placebo | -0.65691 | 8.14832 | 1 | -20.4908 | 19.177 |
|  |  |  |  |  |  | 5mg | 2.97362 | 8.0485 | 1 | -16.6173 | 22.5645 |
|  |  |  |  |  | Placebo | 10mg | 0.65691 | 8.14832 | 1 | -19.177 | 20.4908 |
|  |  |  |  |  |  | 5mg | 3.63053 | 7.73456 | 1 | -15.1962 | 22.4573 |
|  |  |  |  |  | 5mg | 10mg | -2.97362 | 8.0485 | 1 | -22.5645 | 16.6173 |
|  |  |  |  |  |  | Placebo | -3.63053 | 7.73456 | 1 | -22.4573 | 15.1962 |
| BMD | 2, 107 | 0.666 | 0.516 | -0.006 | 10mg | Placebo | -3.2092 | 6.77016 | 1 | -19.6756 | 13.2572 |
|  |  |  |  |  |  | 5mg | -7.90949 | 6.9023 | 0.763 | -24.6973 | 8.8783 |
|  |  |  |  |  | Placebo | 10mg | 3.2092 | 6.77016 | 1 | -13.2572 | 19.6756 |
|  |  |  |  |  |  | 5mg | -4.70029 | 6.72042 | 1 | -21.0457 | 11.6451 |
|  |  |  |  |  | 5mg | 10mg | 7.90949 | 6.9023 | 0.763 | -8.8783 | 24.6973 |
|  |  |  |  |  |  | Placebo | 4.70029 | 6.72042 | 1 | -11.6451 | 21.0457 |
| BMC | 2, 98 | 0.206 | 0.814 | -0.016 | 10mg | Placebo | 0.51272 | 0.80279 | 1 | -1.4427 | 2.4681 |
|  |  |  |  |  |  | 5mg | 0.31612 | 0.81364 | 1 | -1.6657 | 2.298 |
|  |  |  |  |  | Placebo | 10mg | -0.51272 | 0.80279 | 1 | -2.4681 | 1.4427 |
|  |  |  |  |  |  | 5mg | -0.19659 | 0.78353 | 1 | -2.1051 | 1.7119 |
|  |  |  |  |  | 5mg | 10mg | -0.31612 | 0.81364 | 1 | -2.298 | 1.6657 |
|  |  |  |  |  |  | Placebo | 0.19659 | 0.78353 | 1 | -1.7119 | 2.1051 |
| LTM | 2, 101 | 0.877 | 0.419 | -0.002 | 10mg | Placebo | 0.94132 | 0.75367 | 0.644 | -0.8935 | 2.7761 |
|  |  |  |  |  |  | 5mg | 0.78668 | 0.7446 | 0.88 | -1.0261 | 2.5994 |
|  |  |  |  |  | Placebo | 10mg | -0.94132 | 0.75367 | 0.644 | -2.7761 | 0.8935 |
|  |  |  |  |  |  | 5mg | -0.15464 | 0.70908 | 1 | -1.8809 | 1.5716 |
|  |  |  |  |  | 5mg | 10mg | -0.78668 | 0.7446 | 0.88 | -2.5994 | 1.0261 |
|  |  |  |  |  |  | Placebo | 0.15464 | 0.70908 | 1 | -1.5716 | 1.8809 |

| Females | df | F | p-value | Effect Size** | Group 1 | Group 2 | Mean Difference | Std Error | p-value | Lower Bound | Upper Bound |
| --- | --- | --- | --- | --- | --- | --- | --- | --- | --- | --- | --- |
| VAT | 2, 36 | 1.135 | 0.333 | 0.007 | 10mg | Placebo | -19.95911 | 18.55764 | 0.868 | -66.5581 | 26.6399 |
|  |  |  |  |  |  | 5mg | -27.57582 | 18.35482 | 0.425 | -73.6655 | 18.5139 |
|  |  |  |  |  | Placebo | 10mg | 19.95911 | 18.55764 | 0.868 | -26.6399 | 66.5581 |
|  |  |  |  |  |  | 5mg | -7.6167 | 15.23438 | 1 | -45.8708 | 30.6374 |
|  |  |  |  |  | 5mg | 10mg | 27.57582 | 18.35482 | 0.425 | -18.5139 | 73.6655 |
|  |  |  |  |  |  | Placebo | -0.08426 | 0.75078 | 1 | -1.9721 | 1.8036 |
| BMD | 2, 35 | 0.029 | 0.972 | -0.055 | 10mg | Placebo | -0.17587 | 0.75078 | 1 | -2.0637 | 1.712 |
|  |  |  |  |  |  | 5mg | 0.08426 | 0.75078 | 1 | -1.8036 | 1.9721 |
|  |  |  |  |  | Placebo | 10mg | -0.09161 | 0.6262 | 1 | -1.6662 | 1.483 |
|  |  |  |  |  |  | 5mg | 0.17587 | 0.75078 | 1 | -1.712 | 2.0637 |
|  |  |  |  |  | 5mg | 10mg | 0.09161 | 0.6262 | 1 | -1.483 | 1.6662 |
|  |  |  |  |  |  | Placebo | -0.92574 | 1.60565 | 1 | -4.9632 | 3.1117 |
| BMC | 2, 35 | 0.699 | 0.504 | -0.017 | 10mg | Placebo | -1.86067 | 1.60565 | 0.763 | -5.8981 | 2.1768 |
|  |  |  |  |  |  | 5mg | 0.92574 | 1.60565 | 1 | -3.1117 | 4.9632 |
|  |  |  |  |  | Placebo | 10mg | -0.93493 | 1.3392 | 1 | -4.3024 | 2.4325 |
|  |  |  |  |  |  | 5mg | 1.86067 | 1.60565 | 0.763 | -2.1768 | 5.8981 |
|  |  |  |  |  | 5mg | 10mg | 0.93493 | 1.3392 | 1 | -2.4325 | 4.3024 |
|  |  |  |  |  |  | Placebo | 3.60472* | 1.39919 | 0.043 | 0.0913 | 7.1182 |
| LTM | 2, 36 | 4.208 | 0.023 | 0.144 | 10mg | Placebo | 3.77419* | 1.37276 | 0.028 | 0.3271 | 7.2212 |
|  |  |  |  |  |  | 5mg | -3.60472* | 1.39919 | 0.043 | -7.1182 | -0.0913 |
|  |  |  |  |  | Placebo | 10mg | 0.16947 | 1.08284 | 1 | -2.5496 | 2.8885 |
|  |  |  |  |  |  | 5mg | -3.77419* | 1.37276 | 0.028 | -7.2212 | -0.3271 |
|  |  |  |  |  | 5mg | 10mg | -0.16947 | 1.08284 | 1 | -2.8885 | 2.5496 |
|  |  |  |  |  |  | Placebo |  |  |  |  |  |

| Males | df | F | p-value | Effect Size** | Group 1 | Group 2 | Mean Difference | Std Error | p-value | Lower Bound | Upper Bound |
| --- | --- | --- | --- | --- | --- | --- | --- | --- | --- | --- | --- |
| VAT | 2, 63 | 3.548 | 0.035 | 0.073 | 10mg | Placebo | 7.65337 | 7.46072 | 0.927 | -10.6969 | 26.0036 |
|  |  |  |  |  |  | 5mg | 19.52029* | 7.37156 | 0.031 | 1.3893 | 37.6513 |
|  |  |  |  |  | Placebo | 10mg | -7.65337 | 7.46072 | 0.927 | -26.0036 | 10.6969 |
|  |  |  |  |  |  | 5mg | 11.86692 | 7.54122 | 0.362 | -6.6813 | 30.4152 |
|  |  |  |  |  | 5mg | 10mg | -19.52029* | 7.37156 | 0.031 | -37.6513 | -1.3893 |
|  |  |  |  |  |  | Placebo | -11.86692 | 7.54122 | 0.362 | -30.4152 | 6.6813 |
| BMD | 2, 69 | 0.401 | 0.671 | -0.017 | 10mg | Placebo | -1.57949 | 9.98452 | 1 | -26.079 | 22.92 |
|  |  |  |  |  |  | 5mg | -8.8775 | 10.3552 | 1 | -34.2865 | 16.5315 |
|  |  |  |  |  | Placebo | 10mg | 1.57949 | 9.98452 | 1 | -22.92 | 26.079 |
|  |  |  |  |  |  | 5mg | -7.29801 | 10.63458 | 1 | -33.3926 | 18.7965 |
|  |  |  |  |  | 5mg | 10mg | 8.8775 | 10.3552 | 1 | -16.5315 | 34.2865 |
|  |  |  |  |  |  | Placebo | 7.29801 | 10.63458 | 1 | -18.7965 | 33.3926 |
| BMC | 2, 60 | 1.311 | 0.277 | 0.01 | 10mg | Placebo | 1.04581 | 0.90798 | 0.762 | -1.1905 | 3.2821 |
|  |  |  |  |  |  | 5mg | 1.43883 | 0.93263 | 0.384 | -0.8582 | 3.7358 |
|  |  |  |  |  | Placebo | 10mg | -1.04581 | 0.90798 | 0.762 | -3.2821 | 1.1905 |
|  |  |  |  |  |  | 5mg | 0.39302 | 0.95251 | 1 | -1.953 | 2.739 |
|  |  |  |  |  | 5mg | 10mg | -1.43883 | 0.93263 | 0.384 | -3.7358 | 0.8582 |
|  |  |  |  |  |  | Placebo | -0.39302 | 0.95251 | 1 | -2.739 | 1.953 |
| LTM | 2, 62 | 0.162 | 0.851 | -0.027 | 10mg | Placebo | 0.00392 | 0.88827 | 1 | -2.1818 | 2.1897 |
|  |  |  |  |  |  | 5mg | -0.44255 | 0.88827 | 1 | -2.6283 | 1.7432 |
|  |  |  |  |  | Placebo | 10mg | -0.00392 | 0.88827 | 1 | -2.1897 | 2.1818 |
|  |  |  |  |  |  | 5mg | -0.44648 | 0.90823 | 1 | -2.6813 | 1.7884 |
|  |  |  |  |  | 5mg | 10mg | 0.44255 | 0.88827 | 1 | -1.7432 | 2.6283 |
|  |  |  |  |  |  | Placebo | 0.44648 | 0.90823 | 1 | -1.7884 | 2.6813 |

| **One-way ANOVA for 48 weeks** | | | | | | | | |  | 95% Confidence Interval | |
| --- | --- | --- | --- | --- | --- | --- | --- | --- | --- | --- | --- |
| All Genders | df | F | p-value | Effect Size** | Group 1 | Group 2 | Mean Difference | Std Error | p-value | Lower Bound | Upper Bound |
| VAT | 2, 86 | 0.335 | 0.716 | -0.015 | 10mg | Placebo | -7.23321 | 11.28021 | 1 | -34.7762 | 20.3098 |
|  |  |  |  |  |  | 5mg | 0.67798 | 11.12656 | 1 | -26.4899 | 27.8458 |
|  |  |  |  |  | Placebo | 10mg | 7.23321 | 11.28021 | 1 | -20.3098 | 34.7762 |
|  |  |  |  |  |  | 5mg | 7.91119 | 10.49606 | 1 | -17.7171 | 33.5395 |
|  |  |  |  |  | 5mg | 10mg | -0.67798 | 11.12656 | 1 | -27.8458 | 26.4899 |
|  |  |  |  |  |  | Placebo | -7.91119 | 10.49606 | 1 | -33.5395 | 17.7171 |
| BMD | 2, 107 | 0.365 | 0.695 | -0.012 | 10mg | Placebo | 4.15946 | 8.28251 | 1 | -15.9853 | 24.3042 |
|  |  |  |  |  |  | 5mg | -2.80332 | 8.44416 | 1 | -23.3412 | 17.7346 |
|  |  |  |  |  | Placebo | 10mg | -4.15946 | 8.28251 | 1 | -24.3042 | 15.9853 |
|  |  |  |  |  |  | 5mg | -6.96278 | 8.22166 | 1 | -26.9595 | 13.0339 |
|  |  |  |  |  | 5mg | 10mg | 2.80332 | 8.44416 | 1 | -17.7346 | 23.3412 |
|  |  |  |  |  |  | Placebo | 6.96278 | 8.22166 | 1 | -13.0339 | 26.9595 |
| BMC | 2, 83 | 1.589 | 0.21 | 0.014 | 10mg | Placebo | 0.905 | 0.89251 | 0.941 | -1.2758 | 3.0858 |
|  |  |  |  |  |  | 5mg | 1.61468 | 0.90644 | 0.236 | -0.6002 | 3.8295 |
|  |  |  |  |  | Placebo | 10mg | -0.905 | 0.89251 | 0.941 | -3.0858 | 1.2758 |
|  |  |  |  |  |  | 5mg | 0.70968 | 0.86704 | 1 | -1.4089 | 2.8283 |
|  |  |  |  |  | 5mg | 10mg | -1.61468 | 0.90644 | 0.236 | -3.8295 | 0.6002 |
|  |  |  |  |  |  | Placebo | -0.70968 | 0.86704 | 1 | -2.8283 | 1.4089 |
| LTM | 2, 87 | 2.276 | 0.109 | 0.028 | 10mg | Placebo | 2.49823 | 1.18697 | 0.115 | -0.3993 | 5.3958 |
|  |  |  |  |  |  | 5mg | 1.69882 | 1.17044 | 0.451 | -1.1584 | 4.556 |
|  |  |  |  |  | Placebo | 10mg | -2.49823 | 1.18697 | 0.115 | -5.3958 | 0.3993 |
|  |  |  |  |  |  | 5mg | -0.79942 | 1.1164 | 1 | -3.5247 | 1.9259 |
|  |  |  |  |  | 5mg | 10mg | -1.69882 | 1.17044 | 0.451 | -4.556 | 1.1584 |
|  |  |  |  |  |  | Placebo | 0.79942 | 1.1164 | 1 | -1.9259 | 3.5247 |

| Females | df | F | p-value | Effect Size** | Group 1 | Group 2 | Mean Difference | Std Error | p-value | Lower Bound | Upper Bound |
| --- | --- | --- | --- | --- | --- | --- | --- | --- | --- | --- | --- |
| VAT | 2, 29 | 0.115 | 0.892 | -0.061 | 10mg | Placebo | -11.39146 | 24.13444 | 1 | -72.7149 | 49.9319 |
|  |  |  |  |  |  | 5mg | -8.8483 | 22.97064 | 1 | -67.2146 | 49.518 |
|  |  |  |  |  | Placebo | 10mg | 11.39146 | 24.13444 | 1 | -49.9319 | 72.7149 |
|  |  |  |  |  |  | 5mg | 2.54316 | 18.87685 | 1 | -45.4212 | 50.5075 |
|  |  |  |  |  | 5mg | 10mg | 8.8483 | 22.97064 | 1 | -49.518 | 67.2146 |
|  |  |  |  |  |  | Placebo | -2.54316 | 18.87685 | 1 | -50.5075 | 45.4212 |
| BMD | 2, 35 | 0.575 | 0.568 | -0.023 | 10mg | Placebo | 12.83206 | 12.06834 | 0.885 | -17.5143 | 43.1784 |
|  |  |  |  |  |  | 5mg | 7.06968 | 12.06834 | 1 | -23.2767 | 37.416 |
|  |  |  |  |  | Placebo | 10mg | -12.83206 | 12.06834 | 0.885 | -43.1784 | 17.5143 |
|  |  |  |  |  |  | 5mg | -5.76237 | 10.06569 | 1 | -31.073 | 19.5482 |
|  |  |  |  |  | 5mg | 10mg | -7.06968 | 12.06834 | 1 | -37.416 | 23.2767 |
|  |  |  |  |  |  | Placebo | 5.76237 | 10.06569 | 1 | -19.5482 | 31.073 |
| BMC | 2, 28 | 0.157 | 0.856 | -0.06 | 10mg | Placebo | -0.76923 | 1.71819 | 1 | -5.1445 | 3.6061 |
|  |  |  |  |  |  | 5mg | -0.90536 | 1.666 | 1 | -5.1478 | 3.3371 |
|  |  |  |  |  | Placebo | 10mg | 0.76923 | 1.71819 | 1 | -3.6061 | 5.1445 |
|  |  |  |  |  |  | 5mg | -0.13613 | 1.45586 | 1 | -3.8434 | 3.5712 |
|  |  |  |  |  | 5mg | 10mg | 0.90536 | 1.666 | 1 | -3.3371 | 5.1478 |
|  |  |  |  |  |  | Placebo | 0.13613 | 1.45586 | 1 | -3.5712 | 3.8434 |
| LTM | 2, 30 | 5.052 | 0.013 | 0.202 | 10mg | Placebo | 6.19390* | 2.09667 | 0.018 | 0.8773 | 11.5105 |
|  |  |  |  |  |  | 5mg | 5.56454* | 1.98498 | 0.026 | 0.5311 | 10.5979 |
|  |  |  |  |  | Placebo | 10mg | -6.19390* | 2.09667 | 0.018 | -11.5105 | -0.8773 |
|  |  |  |  |  |  | 5mg | -0.62936 | 1.72141 | 1 | -4.9944 | 3.7357 |
|  |  |  |  |  | 5mg | 10mg | -5.56454* | 1.98498 | 0.026 | -10.5979 | -0.5311 |
|  |  |  |  |  |  | Placebo | 0.62936 | 1.72141 | 1 | -3.7357 | 4.9944 |

| Males | df | F | p-value | Effect Size** | Group 1 | Group 2 | Mean Difference | Std Error | p-value | Lower Bound | Upper Bound |
| --- | --- | --- | --- | --- | --- | --- | --- | --- | --- | --- | --- |
| VAT | 2, 54 | 0.625 | 0.539 | -0.014 | 10mg | Placebo | -4.61129 | 12.4849 | 1 | -35.4596 | 26.237 |
|  |  |  |  |  |  | 5mg | 9.35657 | 12.81836 | 1 | -22.3157 | 41.0288 |
|  |  |  |  |  | Placebo | 10mg | 4.61129 | 12.4849 | 1 | -26.237 | 35.4596 |
|  |  |  |  |  |  | 5mg | 13.96786 | 12.6615 | 0.825 | -17.3168 | 45.2525 |
|  |  |  |  |  | 5mg | 10mg | -9.35657 | 12.81836 | 1 | -41.0288 | 22.3157 |
|  |  |  |  |  |  | Placebo | -13.96786 | 12.6615 | 0.825 | -45.2525 | 17.3168 |
| BMD | 2, 69 | 0.219 | 0.804 | -0.022 | 10mg | Placebo | 2.24729 | 11.0736 | 1 | -24.9245 | 29.4191 |
|  |  |  |  |  |  | 5mg | -5.39434 | 11.48471 | 1 | -33.5749 | 22.7862 |
|  |  |  |  |  | Placebo | 10mg | -2.24729 | 11.0736 | 1 | -29.4191 | 24.9245 |
|  |  |  |  |  |  | 5mg | -7.64163 | 11.79457 | 1 | -36.5825 | 21.2992 |
|  |  |  |  |  | 5mg | 10mg | 5.39434 | 11.48471 | 1 | -22.7862 | 33.5749 |
|  |  |  |  |  |  | Placebo | 7.64163 | 11.79457 | 1 | -21.2992 | 36.5825 |
| BMC | 2, 52 | 2.949 | 0.061 | 0.067 | 10mg | Placebo | 1.38327 | 1.00751 | 0.527 | -1.1092 | 3.8757 |
|  |  |  |  |  |  | 5mg | 2.57988 | 1.0671 | 0.057 | -0.06 | 5.2198 |
|  |  |  |  |  | Placebo | 10mg | -1.38327 | 1.00751 | 0.527 | -3.8757 | 1.1092 |
|  |  |  |  |  |  | 5mg | 1.19661 | 1.05483 | 0.785 | -1.4129 | 3.8062 |
|  |  |  |  |  | 5mg | 10mg | -2.57988 | 1.0671 | 0.057 | -5.2198 | 0.06 |
|  |  |  |  |  |  | Placebo | -1.19661 | 1.05483 | 0.785 | -3.8062 | 1.4129 |
| LTM | 2, 54 | 0.379 | 0.686 | -0.023 | 10mg | Placebo | 1.15136 | 1.40058 | 1 | -2.3093 | 4.612 |
|  |  |  |  |  |  | 5mg | 0.23314 | 1.43799 | 1 | -3.3199 | 3.7862 |
|  |  |  |  |  | Placebo | 10mg | -1.15136 | 1.40058 | 1 | -4.612 | 2.3093 |
|  |  |  |  |  |  | 5mg | -0.91822 | 1.4204 | 1 | -4.4278 | 2.5914 |
|  |  |  |  |  | 5mg | 10mg | -0.23314 | 1.43799 | 1 | -3.7862 | 3.3199 |
|  |  |  |  |  |  | Placebo | 0.91822 | 1.4204 | 1 | -2.5914 | 4.4278 |

| **Supplementary Table 5. Analysis of blood marker changes over time** | | | | | | | | | | | | | |
| --- | --- | --- | --- | --- | --- | --- | --- | --- | --- | --- | --- | --- | --- |
|  |  |  |  |  |  |  |  |  |  |  |  |  |  |
| **Repeated Measures Mixed ANOVA** | | | | | | | | | | | | | |
| All Genders |  |  |  |  |  |  |  |  |  |  |  |  |  |
|  |  | df 1 | df 2 | F | p-value | Partial Eta Squared |  |  | df | df 2 | F | p-value | Partial Eta Squared |
| Time by Group |  |  |  |  |  |  | Time Only |  |  |  |  |  |  |
|  | Total Cholesterol^ | 3.765 | 180.728 | 1.505 | 0.205 | 0.03 |  | Total Cholesterol^ | 1.883 | 180.728 | 1.478 | 0.231 | 0.015 |
|  | HDL Cholesterol^ | 3.642 | 174.804 | 0.158 | 0.949 | 0.003 |  | HDL Cholesterol^ | 1.821 | 174.804 | 0.464 | 0.612 | 0.005 |
|  | Triglycerides^ | 3.401 | 163.228 | 1.4 | 0.242 | 0.028 |  | Triglycerides^ | 1.7 | 163.228 | 0.018 | 0.97 | 0 |
|  | LDL Cholesterol^ | 3.699 | 177.572 | 1.256 | 0.29 | 0.026 |  | LDL Cholesterol^ | 1.85 | 177.572 | 1.979 | 0.145 | 0.02 |
|  | CHOL/HDLC Ratio^ | 3.549 | 170.371 | 0.817 | 0.504 | 0.017 |  | CHOL/HDLC Ratio^ | 1.775 | 170.371 | 2.183 | 0.122 | 0.022 |
|  | Non HDL Cholesterol | 4 | 192 | 2.45 | 0.048 | 0.049 |  | Non HDL Cholesterol | 2 | 192 | 0.815 | 0.444 | 0.008 |
|  | VLDL Cholesterol^ | 3.792 | 182.019 | 0.255 | 0.898 | 0.005 |  | VLDL Cholesterol^ | 1.896 | 182.019 | 0.13 | 0.868 | 0.001 |
|  | hsCRP | 4 | 160 | 1.208 | 0.31 | 0.029 |  | hsCRP | 2 | 160 | 0.245 | 0.783 | 0.003 |
|  | Glucose | 4 | 160 | 0.135 | 0.969 | 0.003 |  | Glucose | 2 | 160 | 1.383 | 0.254 | 0.017 |
|  | BUN | 4 | 160 | 1.917 | 0.11 | 0.046 |  | BUN | 2 | 160 | 1.856 | 0.16 | 0.023 |
|  | Creatinine | 4 | 160 | 0.994 | 0.413 | 0.024 |  | Creatinine | 2 | 160 | 0.52 | 0.595 | 0.006 |
|  | eGFR | 4 | 160 | 0.271 | 0.896 | 0.007 |  | eGFR | 2 | 160 | 0.834 | 0.436 | 0.01 |
|  | Sodium | 4 | 160 | 1.558 | 0.188 | 0.037 |  | Sodium | 2 | 160 | 1.16 | 0.316 | 0.014 |
|  | Potassium^ | 3.693 | 147.719 | 0.824 | 0.504 | 0.02 |  | Potassium^ | 1.846 | 147.719 | 1.862 | 0.162 | 0.023 |
|  | Chloride | 4 | 160 | 1.503 | 0.204 | 0.036 |  | Chloride | 2 | 160 | 0.86 | 0.425 | 0.011 |
|  | Carbon Dioxide | 4 | 160 | 1.925 | 0.109 | 0.046 |  | Carbon Dioxide | 2 | 160 | 0.86 | 0.425 | 0.011 |
|  | Calcium | 4 | 160 | 2.233 | 0.068 | 0.053 |  | Calcium | 2 | 160 | 8.803 | <.001 | 0.099 |
|  | Total Protein | 4 | 190 | 1.855 | 0.12 | 0.038 |  | Total Protein | 2 | 190 | 2.94 | 0.055 | 0.03 |
|  | Albumin | 4 | 190 | 1.822 | 0.126 | 0.037 |  | Albumin | 2 | 190 | 2.077 | 0.128 | 0.021 |
|  | Globulin | 4 | 190 | 0.702 | 0.591 | 0.015 |  | Globulin | 2 | 190 | 0.911 | 0.404 | 0.009 |
|  | Albumin Globulin Ratio | 4 | 190 | 0.467 | 0.76 | 0.01 |  | Albumin Globulin Ratio | 2 | 190 | 0.502 | 0.606 | 0.005 |
|  | Total Bilirubin^ | 3.464 | 190 | 0.394 | 0.813 | 0.008 |  | Total Bilirubin | 2 | 190 | 0.413 | 0.662 | 0.004 |
|  | Alkaline Phosphatase^ | 3.566 | 164.539 | 0.24 | 0.893 | 0.005 |  | Alkaline Phosphatase^ | 1.732 | 164.539 | 0.405 | 0.638 | 0.004 |
|  | AST | 2 | 169.395 | 0.815 | 0.505 | 0.017 |  | AST^ | 1.783 | 169.395 | 0.181 | 0.81 | 0.002 |
|  | ALT^ | 2.821 | 133.978 | 0.714 | 0.537 | 0.015 |  | ALT^ | 1.41 | 133.978 | 1.434 | 0.241 | 0.015 |
|  | Uric Acid^ | 3.493 | 165.937 | 0.293 | 0.859 | 0.006 |  | Uric Acid^ | 1.747 | 165.937 | 3.603 | 0.035 | 0.037 |
|  | Insulin | 4 | 190 | 0.141 | 0.967 | 0.003 |  | Insulin | 2 | 190 | 1.148 | 0.32 | 0.012 |
|  | WBC^ | 3.606 | 178.475 | 1.564 | 0.191 | 0.031 |  | WBC^ | 1.803 | 178.475 | 1.346 | 0.262 | 0.013 |
|  | RBC | 4 | 198 | 2.677 | 0.033 | 0.051 |  | RBC | 2 | 198 | 2.024 | 0.135 | 0.02 |
|  | Hemoglobin | 4 | 198 | 1.511 | 0.2 | 0.03 |  | Hemoglobin | 2 | 198 | 1.191 | 0.306 | 0.012 |
|  | Hematocrit | 4 | 198 | 2.022 | 0.093 | 0.039 |  | Hematocrit | 2 | 198 | 1.439 | 0.24 | 0.014 |
|  | MCV^ | 3.652 | 180.788 | 2.103 | 0.088 | 0.041 |  | MCV^ | 1.826 | 180.788 | 1.633 | 0.2 | 0.016 |
|  | MCH^ | 3.639 | 180.115 | 2.999 | 0.024 | 0.057 |  | MCH^ | 1.819 | 180.115 | 1.666 | 0.194 | 0.017 |
|  | MCHC | 4 | 198 | 0.711 | 0.585 | 0.014 |  | MCHC | 2 | 198 | 0.237 | 0.789 | 0.002 |
|  | RDW^ | 3.599 | 178.166 | 1.378 | 0.247 | 0.027 |  | RDW^ | 1.8 | 178.166 | 2.354 | 0.104 | 0.023 |
|  | Platelet Count | 4 | 198 | 2.153 | 0.076 | 0.042 |  | Platelet Count | 2 | 198 | 0.703 | 0.496 | 0.007 |
|  | MPV^ | 3.96 | 198 | 2.917 | 0.023 | 0.056 |  | MPV | 2 | 198 | 0.081 | 0.922 | 0.001 |
|  | Absolute Neutrophils^ | 3.736 | 184.927 | 0.995 | 0.408 | 0.02 |  | Absolute Neutrophils^ | 1.868 | 184.927 | 1.344 | 0.263 | 0.013 |
|  | Absolute Lymphocytes^ | 3.74 | 185.107 | 2.209 | 0.074 | 0.043 |  | Absolute Lymphocytes^ | 1.87 | 185.107 | 2.787 | 0.068 | 0.027 |
|  | Absolute Monocytes^ | 3.393 | 167.95 | 0.728 | 0.552 | 0.015 |  | Absolute Monocytes^ | 1.696 | 167.95 | 0.872 | 0.405 | 0.009 |
|  | Absolute Eosinophils^ | 3.664 | 181.37 | 0.335 | 0.838 | 0.007 |  | Absolute Eosinophils^ | 1.832 | 181.37 | 0.002 | 0.996 | 0 |
|  | Absolute Basophils^ | 3.775 | 186.871 | 1.127 | 0.344 | 0.022 |  | Absolute Basophils^ | 1.888 | 186.871 | 0.935 | 0.39 | 0.009 |
|  | Neutrophils | 4 | 198 | 0.361 | 0.836 | 0.007 |  | Neutrophils | 2 | 198 | 2.326 | 0.1 | 0.023 |
|  | Lymphocytes | 4 | 198 | 0.795 | 0.53 | 0.016 |  | Lymphocytes | 2 | 198 | 2.3 | 0.103 | 0.023 |
|  | Monocytes | 4 | 198 | 0.972 | 0.424 | 0.019 |  | Monocytes | 2 | 198 | 0.633 | 0.532 | 0.006 |
|  | Eosinophils | 4 | 198 | 0.475 | 0.754 | 0.01 |  | Eosinophils | 2 | 198 | 0.083 | 0.92 | 0.001 |
|  | Basophils^ | 3.238 | 160.269 | 1.238 | 0.298 | 0.024 |  | Basophils^ | 1.619 | 160.269 | 0.671 | 0.483 | 0.007 |
|  | DHEA Sulfate | 4 | 66 | 0.507 | 0.731 | 0.03 |  | DHEA Sulfate | 2 | 66 | 2.845 | 0.065 | 0.079 |
|  | Vitamin D, 25-OH | 4 | 66 | 0.389 | 0.816 | 0.023 |  | Vitamin D, 25-OH | 2 | 66 | 2.498 | 0.09 | 0.07 |
|  | Hemoglobin A1C | 4 | 66 | 1.263 | 0.294 | 0.071 |  | Hemoglobin A1C | 2 | 66 | 7.566 | 0.001 | 0.187 |
|  | ApoB | 4 | 66 | 0.872 | 0.485 | 0.05 |  | ApoB | 2 | 66 | 3.116 | 0.051 | 0.086 |
|  | HOMA_IR | 4 | 66 | 0.677 | 0.61 | 0.039 |  | HOMA_IR | 2 | 66 | 0.669 | 0.516 | 0.02 |

| Females |  |  |  |  |  |  |  |  |  |  |  |  |
| --- | --- | --- | --- | --- | --- | --- | --- | --- | --- | --- | --- | --- |
|  | df 1 | df 2 | F | p-value | Partial Eta Squared |  |  | df | df 2 | F | p-value | Partial Eta Squared |
| Time by Group |  |  |  |  |  |  | Time Only |  |  |  |  |  |
| Total Cholesterol^ | 3.264 | 53.85 | 0.794 | 0.512 | 0.046 |  | Total Cholesterol^ | 1.632 | 53.85 | 0.548 | 0.546 | 0.016 |
| HDL Cholesterol | 4 | 66 | 0.296 | 0.88 | 0.018 |  | HDL Cholesterol | 2 | 66 | 1.134 | 0.328 | 0.033 |
| Triglycerides | 4 | 66 | 1.644 | 0.174 | 0.091 |  | Triglycerides | 2 | 66 | 0.649 | 0.526 | 0.019 |
| LDL Cholesterol^ | 3.153 | 52.016 | 0.577 | 0.641 | 0.034 |  | LDL Cholesterol^ | 1.576 | 52.016 | 0.924 | 0.383 | 0.027 |
| CHOL/HDLC Ratio^ | 3.385 | 55.85 | 0.851 | 0.483 | 0.049 |  | CHOL/HDLC Ratio^ | 1.692 | 55.85 | 1.409 | 0.252 | 0.041 |
| Non HDL Cholesterol^ | 3.161 | 52.157 | 0.906 | 0.449 | 0.052 |  | Non HDL Cholesterol^ | 1.581 | 52.157 | 0.933 | 0.38 | 0.027 |
| VLDL Cholesterol^ | 3.184 | 52.544 | 0.313 | 0.827 | 0.019 |  | VLDL Cholesterol^ | 1.592 | 52.544 | 0.2 | 0.769 | 0.006 |
| hsCRP^ | 3.169 | 41.198 | 0.505 | 0.691 | 0.037 |  | hsCRP^ | 1.585 | 41.198 | 0.471 | 0.584 | 0.018 |
| Glucose | 4 | 52 | 1.707 | 0.163 | 0.116 |  | Glucose | 2 | 52 | 1.191 | 0.312 | 0.044 |
| BUN | 4 | 52 | 0.146 | 0.964 | 0.011 |  | BUN | 2 | 52 | 0.85 | 0.433 | 0.032 |
| Creatinine | 4 | 52 | 0.627 | 0.645 | 0.046 |  | Creatinine | 2 | 52 | 1.184 | 0.314 | 0.044 |
| eGFR | 4 | 52 | 0.962 | 0.436 | 0.069 |  | eGFR | 2 | 52 | 0.558 | 0.576 | 0.021 |
| Sodium | 4 | 52 | 1.143 | 0.347 | 0.081 |  | Sodium | 2 | 52 | 1.591 | 0.214 | 0.058 |
| Potassium | 4 | 52 | 0.2 | 0.937 | 0.015 |  | Potassium | 2 | 52 | 0.614 | 0.545 | 0.023 |
| Chloride | 4 | 52 | 1.427 | 0.238 | 0.099 |  | Chloride | 2 | 52 | 0.248 | 0.781 | 0.009 |
| Carbon Dioxide | 4 | 52 | 4.996 | 0.002 | 0.278 |  | Carbon Dioxide | 2 | 52 | 5.568 | 0.006 | 0.176 |
| Calcium | 4 | 52 | 2.199 | 0.082 | 0.145 |  | Calcium | 2 | 52 | 4.866 | 0.012 | 0.158 |
| Total Protein | 4 | 70 | 0.613 | 0.655 | 0.034 |  | Total Protein | 2 | 70 | 2.245 | 0.114 | 0.06 |
| Albumin | 4 | 70 | 0.891 | 0.474 | 0.048 |  | Albumin | 2 | 70 | 2.439 | 0.095 | 0.065 |
| Globulin ^ | 3.13 | 54.775 | 0.584 | 0.635 | 0.032 |  | Globulin^ | 1.565 | 54.775 | 0.806 | 0.425 | 0.023 |
| Albumin Globulin Ratio | 4 | 70 | 0.936 | 0.448 | 0.051 |  | Albumin Globulin Ratio | 2 | 70 | 0.635 | 0.533 | 0.018 |
| Total Bilirubin | 4 | 70 | 0.209 | 0.933 | 0.012 |  | Total Bilirubin | 2 | 70 | 0.718 | 0.492 | 0.02 |
| Alkaline Phosphatase^ | 2.972 | 52.018 | 1.204 | 0.317 | 0.064 |  | Alkaline Phosphatase^ | 1.486 | 52.018 | 1.222 | 0.293 | 0.034 |
| AST | 4 | 70 | 1.992 | 0.105 | 0.102 |  | AST | 2 | 70 | 0.179 | 0.836 | 0.005 |
| ALT^ | 2.469 | 43.211 | 0.425 | 0.698 | 0.024 |  | ALT^ | 1.235 | 43.211 | 1.671 | 0.205 | 0.046 |
| Uric Acid^ | 4 | 70 | 1.185 | 0.325 | 0.063 |  | Uric Acid | 2 | 70 | 3.551 | 0.034 | 0.092 |
| Insulin | 4 | 70 | 0.434 | 0.784 | 0.024 |  | Insulin | 2 | 70 | 0.63 | 0.535 | 0.018 |
| WBC^ | 2.902 | 50.78 | 0.533 | 0.656 | 0.03 |  | WBC^ | 1.451 | 50.78 | 4.863 | 0.02 | 0.122 |
| RBC | 4 | 70 | 0.236 | 0.917 | 0.013 |  | RBC | 2 | 70 | 3.142 | 0.049 | 0.082 |
| Hemoglobin | 4 | 70 | 0.073 | 0.99 | 0.004 |  | Hemoglobin | 2 | 70 | 1.425 | 0.247 | 0.039 |
| Hematocrit | 4 | 70 | 0.235 | 0.918 | 0.013 |  | Hematocrit | 2 | 70 | 3.668 | 0.031 | 0.095 |
| MCV | 4 | 70 | 0.208 | 0.933 | 0.012 |  | MCV | 2 | 70 | 0.83 | 0.44 | 0.023 |
| MCH | 4 | 70 | 0.704 | 0.592 | 0.039 |  | MCH | 2 | 70 | 1.45 | 0.242 | 0.04 |
| MCHC | 4 | 70 | 0.623 | 0.648 | 0.034 |  | MCHC | 2 | 70 | 3.007 | 0.056 | 0.079 |
| RDW^ | 2.895 | 50.662 | 0.182 | 0.903 | 0.01 |  | RDW^ | 1.447 | 50.662 | 1.474 | 0.238 | 0.04 |
| Platelet Count | 4 | 70 | 1.464 | 0.222 | 0.077 |  | Platelet Count | 2 | 70 | 0.289 | 0.75 | 0.008 |
| MPV | 4 | 70 | 1.034 | 0.396 | 0.056 |  | MPV | 2 | 70 | 0.295 | 0.746 | 0.008 |
| Absolute Neutrophils^ | 3.147 | 55.08 | 0.7 | 0.563 | 0.038 |  | Absolute Neutrophils^ | 1.574 | 55.08 | 4.484 | 0.023 | 0.114 |
| Absolute Lymphocytes^ | 3.095 | 54.161 | 1.718 | 0.173 | 0.089 |  | Absolute Lymphocytes^ | 1.547 | 54.161 | 3.538 | 0.047 | 0.092 |
| Absolute Monocytes^ | 2.937 | 51.405 | 0.494 | 0.684 | 0.027 |  | Absolute Monocytes^ | 1.469 | 51.405 | 4.107 | 0.033 | 0.105 |
| Absolute Eosinophils^ | 3.432 | 60.058 | 0.07 | 0.984 | 0.004 |  | Absolute Eosinophils^ | 1.716 | 60.058 | 1.413 | 0.251 | 0.039 |
| Absolute Basophils | 4 | 70 | 2.175 | 0.081 | 0.111 |  | Absolute Basophils | 2 | 70 | 0.968 | 0.385 | 0.027 |
| Neutrophils | 4 | 70 | 0.942 | 0.445 | 0.051 |  | Neutrophils | 2 | 70 | 5.996 | 0.004 | 0.146 |
| Lymphocytes | 4 | 70 | 1.653 | 0.171 | 0.086 |  | Lymphocytes | 2 | 70 | 4.253 | 0.018 | 0.108 |
| Monocytes | 4 | 70 | 0.345 | 0.846 | 0.019 |  | Monocytes | 2 | 70 | 2.023 | 0.14 | 0.055 |
| Eosinophils | 4 | 70 | 0.231 | 0.92 | 0.013 |  | Eosinophils | 2 | 70 | 2.706 | 0.074 | 0.072 |
| Basophils^ | 2.777 | 48.595 | 1.767 | 0.17 | 0.092 |  | Basophils^ | 1.388 | 48.595 | 0.796 | 0.415 | 0.022 |
| DHEA Sulfate | 4 | 26 | 0.404 | 0.804 | 0.059 |  | DHEA Sulfate | 2 | 26 | 0.111 | 0.895 | 0.008 |
| Vitamin D, 25-OH | 4 | 26 | 0.84 | 0.512 | 0.114 |  | Vitamin D, 25-OH | 2 | 26 | 0.756 | 0.479 | 0.055 |
| Hemoglobin A1C | 4 | 26 | 0.961 | 0.445 | 0.129 |  | Hemoglobin A1C | 2 | 26 | 1.52 | 0.237 | 0.105 |
| ApoB | 4 | 26 | 0.444 | 0.776 | 0.064 |  | ApoB | 2 | 26 | 0.892 | 0.422 | 0.064 |
| HOMA_IR | 4 | 26 | 1.948 | 0.132 | 0.231 |  | HOMA_IR | 2 | 26 | 2.394 | 0.111 | 0.156 |

| Males |  |  |  |  |  |  |  |  |  |  |  |  |
| --- | --- | --- | --- | --- | --- | --- | --- | --- | --- | --- | --- | --- |
|  | df 1 | df 2 | F | p-value | Partial Eta Squared |  |  | df | df 2 | F | p-value | Partial Eta Squared |
| Time by Group |  |  |  |  |  |  | Time Only |  |  |  |  |  |
| Total Cholesterol | 4 | 120 | 0.907 | 0.462 | 0.029 |  | Total Cholesterol | 2 | 120 | 0.664 | 0.517 | 0.011 |
| HDL Cholesterol^ | 3.53 | 105.89 | 0.203 | 0.919 | 0.007 |  | HDL Cholesterol^ | 1.765 | 105.89 | 1.001 | 0.363 | 0.016 |
| Triglycerides^ | 3.222 | 96.662 | 0.553 | 0.66 | 0.018 |  | Triglycerides^ | 1.611 | 96.662 | 0.218 | 0.756 | 0.004 |
| LDL Cholesterol | 4 | 120 | 1.035 | 0.392 | 0.033 |  | LDL Cholesterol | 2 | 120 | 0.763 | 0.469 | 0.013 |
| CHOL/HDLC Ratio^ | 3.461 | 103.843 | 0.897 | 0.457 | 0.029 |  | CHOL/HDLC Ratio^ | 1.731 | 103.843 | 1.28 | 0.28 | 0.021 |
| Non HDL Cholesterol^ | 3.646 | 109.371 | 2.172 | 0.083 | 0.068 |  | Non HDL Cholesterol^ | 1.823 | 109.371 | 0.192 | 0.806 | 0.003 |
| VLDL Cholesterol^ | 3.08 | 92.403 | 0.727 | 0.542 | 0.024 |  | VLDL Cholesterol^ | 1.54 | 92.403 | 0.321 | 0.669 | 0.005 |
| hsCRP | 4 | 102 | 0.804 | 0.526 | 0.031 |  | hsCRP | 2 | 102 | 0.082 | 0.922 | 0.002 |
| Glucose | 4 | 102 | 0.934 | 0.447 | 0.035 |  | Glucose | 2 | 102 | 0.532 | 0.589 | 0.01 |
| BUN | 4 | 102 | 2.805 | 0.03 | 0.099 |  | BUN | 2 | 102 | 0.816 | 0.445 | 0.016 |
| Creatinine | 4 | 102 | 1.536 | 0.197 | 0.057 |  | Creatinine | 2 | 102 | 0.533 | 0.589 | 0.01 |
| eGFR | 4 | 102 | 0.85 | 0.497 | 0.032 |  | eGFR | 2 | 102 | 2.637 | 0.076 | 0.049 |
| Sodium | 4 | 102 | 0.58 | 0.678 | 0.022 |  | Sodium | 2 | 102 | 0.284 | 0.754 | 0.006 |
| Potassium | 4 | 102 | 0.789 | 0.535 | 0.03 |  | Potassium | 2 | 102 | 2.347 | 0.101 | 0.044 |
| Chloride | 4 | 102 | 0.951 | 0.438 | 0.036 |  | Chloride | 2 | 102 | 1.818 | 0.168 | 0.034 |
| Carbon Dioxide | 4 | 102 | 0.988 | 0.417 | 0.037 |  | Carbon Dioxide | 2 | 102 | 3.604 | 0.031 | 0.066 |
| Calcium | 4 | 102 | 1.382 | 0.245 | 0.051 |  | Calcium | 2 | 102 | 4.9 | 0.009 | 0.088 |
| Total Protein | 4 | 114 | 1.02 | 0.4 | 0.035 |  | Total Protein | 2 | 114 | 1.536 | 0.22 | 0.026 |
| Albumin | 4 | 114 | 1.146 | 0.338 | 0.039 |  | Albumin | 2 | 114 | 0.868 | 0.423 | 0.015 |
| Globulin | 4 | 114 | 0.591 | 0.67 | 0.02 |  | Globulin | 2 | 114 | 0.459 | 0.633 | 0.008 |
| Albumin Globulin Ratio | 4 | 114 | 0.45 | 0.772 | 0.016 |  | Albumin Globulin Ratio | 2 | 114 | 0.723 | 0.488 | 0.013 |
| Total Bilirubin | 4 | 114 | 0.321 | 0.863 | 0.011 |  | Total Bilirubin | 2 | 114 | 0.912 | 0.405 | 0.016 |
| Alkaline Phosphatase^ | 3.624 | 103.27 | 1.712 | 0.159 | 0.057 |  | Alkaline Phosphatase^ | 1.812 | 103.27 | 0.717 | 0.478 | 0.012 |
| AST^ | 3.571 | 101.769 | 1.35 | 0.259 | 0.045 |  | AST^ | 1.785 | 101.769 | 0.296 | 0.72 | 0.005 |
| ALT^ | 2.919 | 83.185 | 0.962 | 0.413 | 0.033 |  | ALT^ | 1.459 | 83.185 | 0.737 | 0.442 | 0.013 |
| Uric Acid^ | 3.275 | 93.338 | 0.217 | 0.899 | 0.008 |  | Uric Acid^ | 1.638 | 93.338 | 1.675 | 0.197 | 0.029 |
| Insulin | 4 | 114 | 0.26 | 0.903 | 0.009 |  | Insulin | 2 | 114 | 0.676 | 0.51 | 0.012 |
| WBC | 4 | 122 | 1.779 | 0.137 | 0.055 |  | WBC | 2 | 122 | 0.783 | 0.459 | 0.013 |
| RBC | 4 | 122 | 2.995 | 0.021 | 0.089 |  | RBC | 2 | 122 | 0.328 | 0.721 | 0.005 |
| Hemoglobin | 4 | 122 | 1.705 | 0.153 | 0.053 |  | Hemoglobin | 2 | 122 | 0.344 | 0.71 | 0.006 |
| Hematocrit | 4 | 122 | 1.837 | 0.126 | 0.057 |  | Hematocrit | 2 | 122 | 0.055 | 0.947 | 0.001 |
| MCV | 4 | 122 | 2.099 | 0.085 | 0.064 |  | MCV | 2 | 122 | 0.731 | 0.484 | 0.012 |
| MCH^ | 3.605 | 109.939 | 3.142 | 0.021 | 0.093 |  | MCH^ | 1.802 | 109.939 | 0.986 | 0.369 | 0.016 |
| MCHC | 4 | 122 | 0.711 | 0.586 | 0.023 |  | MCHC | 2 | 122 | 0.502 | 0.607 | 0.008 |
| RDW^ | 3.63 | 110.71 | 1.863 | 0.128 | 0.058 |  | RDW^ | 1.815 | 110.71 | 2.024 | 0.141 | 0.032 |
| Platelet Count | 4 | 122 | 1.949 | 0.107 | 0.06 |  | Platelet Count | 2 | 122 | 1.52 | 0.223 | 0.024 |
| MPV | 4 | 122 | 2.151 | 0.079 | 0.066 |  | MPV | 2 | 122 | 0.379 | 0.685 | 0.006 |
| Absolute Neutrophils | 4 | 122 | 1.774 | 0.138 | 0.055 |  | Absolute Neutrophils | 2 | 122 | 0.9 | 0.409 | 0.015 |
| Absolute Lymphocytes^ | 3.381 | 103.132 | 1.156 | 0.332 | 0.037 |  | Absolute Lymphocytes^ | 1.691 | 103.132 | 0.08 | 0.895 | 0.001 |
| Absolute Monocytes | 4 | 122 | 0.642 | 0.634 | 0.021 |  | Absolute Monocytes | 2 | 122 | 0.289 | 0.749 | 0.005 |
| Absolute Eosinophils | 4 | 122 | 0.606 | 0.659 | 0.019 |  | Absolute Eosinophils | 2 | 122 | 1.502 | 0.227 | 0.024 |
| Absolute Basophils^ | 3.586 | 109.36 | 0.194 | 0.927 | 0.006 |  | Absolute Basophils^ | 1.793 | 109.36 | 0.065 | 0.921 | 0.001 |
| Neutrophils | 4 | 122 | 1.158 | 0.333 | 0.037 |  | Neutrophils | 2 | 122 | 0.675 | 0.511 | 0.011 |
| Lymphocytes | 4 | 122 | 1.473 | 0.214 | 0.046 |  | Lymphocytes | 2 | 122 | 0.768 | 0.466 | 0.012 |
| Monocytes^ | 3.592 | 109.56 | 0.694 | 0.583 | 0.022 |  | Monocytes^ | 1.796 | 109.56 | 0.021 | 0.971 | 0 |
| Eosinophils | 4 | 122 | 0.256 | 0.906 | 0.008 |  | Eosinophils | 2 | 122 | 1.736 | 0.181 | 0.028 |
| Basophils | 4 | 122 | 0.09 | 0.986 | 0.003 |  | Basophils | 2 | 122 | 0.031 | 0.969 | 0.001 |
| DHEA Sulfate^ | 2.739 | 23.28 | 0.847 | 0.473 | 0.091 |  | DHEA Sulfate^ | 1.369 | 23.28 | 4.004 | 0.046 | 0.191 |
| Vitamin D, 25-OH | 4 | 34 | 1.522 | 0.218 | 0.152 |  | Vitamin D, 25-OH | 2 | 34 | 2.351 | 0.111 | 0.121 |
| Hemoglobin A1C | 4 | 34 | 0.591 | 0.671 | 0.065 |  | Hemoglobin A1C | 2 | 34 | 7.055 | 0.003 | 0.293 |
| ApoB | 4 | 34 | 0.803 | 0.532 | 0.086 |  | ApoB | 2 | 34 | 2.213 | 0.125 | 0.115 |
| HOMA_IR | 4 | 34 | 0.947 | 0.449 | 0.1 |  | HOMA_IR | 2 | 34 | 0.095 | 0.909 | 0.006 |

| **Pairwise Comparisons** | | | | | | |  |  |  |  |  |  |  |  |  |  |  |  |
| --- | --- | --- | --- | --- | --- | --- | --- | --- | --- | --- | --- | --- | --- | --- | --- | --- | --- | --- |
|  |  |  |  | All Genders | | | | | Females Only | | | | | Males Only | | | | |
|  | Group | Time 1 | Time 2 | Mean Difference | Std. Error | p-value | 95% Confidence Interval | | Mean Difference | Std. Error | p-value | 95% Confidence Interval | | Mean Difference | Std. Error | p-value | 95% Confidence Interval | |
|  |  |  |  |  |  |  | Lower Bound | Upper Bound |  |  |  | Lower Bound | Upper Bound |  |  |  | Lower Bound | Upper Bound |
| Total Cholesterol | 10mg | 1 | 2 | -2.6 | 4.457 | 1 | -13.46 | 8.26 | -1.25 | 7.531 | 1 | -20.244 | 17.744 | -3.091 | 5.65 | 1 | -17.006 | 10.824 |
|  |  |  | 3 | 3.433 | 5.208 | 1 | -9.257 | 16.123 | -0.625 | 10.9 | 1 | -28.118 | 26.868 | 4.909 | 5.914 | 1 | -9.657 | 19.476 |
|  |  | 2 | 1 | 2.6 | 4.457 | 1 | -8.26 | 13.46 | 1.25 | 7.531 | 1 | -17.744 | 20.244 | 3.091 | 5.65 | 1 | -10.824 | 17.006 |
|  |  |  | 3 | 6.033 | 5.56 | 0.842 | -7.515 | 19.582 | 0.625 | 11.829 | 1 | -29.21 | 30.46 | 8 | 6.242 | 0.615 | -7.373 | 23.373 |
|  |  | 3 | 1 | -3.433 | 5.208 | 1 | -16.123 | 9.257 | 0.625 | 10.9 | 1 | -26.868 | 28.118 | -4.909 | 5.914 | 1 | -19.476 | 9.657 |
|  |  |  | 2 | -6.033 | 5.56 | 0.842 | -19.582 | 7.515 | -0.625 | 11.829 | 1 | -30.46 | 29.21 | -8 | 6.242 | 0.615 | -23.373 | 7.373 |
|  | Placebo | 1 | 2 | 10.333 | 4.25 | 0.051 | -0.021 | 20.688 | 12.154 | 5.908 | 0.143 | -2.746 | 27.054 | 9.15 | 5.925 | 0.383 | -5.444 | 23.744 |
|  |  |  | 3 | 10.152 | 4.966 | 0.131 | -1.948 | 22.251 | 13.154 | 8.551 | 0.401 | -8.414 | 34.721 | 8.2 | 6.203 | 0.574 | -7.078 | 23.478 |
|  |  | 2 | 1 | -10.333 | 4.25 | 0.051 | -20.688 | 0.021 | -12.154 | 5.908 | 0.143 | -27.054 | 2.746 | -9.15 | 5.925 | 0.383 | -23.744 | 5.444 |
|  |  |  | 3 | -0.182 | 5.302 | 1 | -13.1 | 12.736 | 1 | 9.279 | 1 | -22.404 | 24.404 | -0.95 | 6.546 | 1 | -17.073 | 15.173 |
|  |  | 3 | 1 | -10.152 | 4.966 | 0.131 | -22.251 | 1.948 | -13.154 | 8.551 | 0.401 | -34.721 | 8.414 | -8.2 | 6.203 | 0.574 | -23.478 | 7.078 |
|  |  |  | 2 | 0.182 | 5.302 | 1 | -12.736 | 13.1 | -1 | 9.279 | 1 | -24.404 | 22.404 | 0.95 | 6.546 | 1 | -15.173 | 17.073 |
|  | 5mg | 1 | 2 | -4.944 | 4.069 | 0.682 | -14.858 | 4.969 | -5.733 | 5.5 | 0.914 | -19.605 | 8.138 | -4.381 | 5.783 | 1 | -18.623 | 9.861 |
|  |  |  | 3 | 0.139 | 4.754 | 1 | -11.445 | 11.723 | 2.933 | 7.961 | 1 | -17.145 | 23.012 | -1.857 | 6.053 | 1 | -16.766 | 13.052 |
|  |  | 2 | 1 | 4.944 | 4.069 | 0.682 | -4.969 | 14.858 | 5.733 | 5.5 | 0.914 | -8.138 | 19.605 | 4.381 | 5.783 | 1 | -9.861 | 18.623 |
|  |  |  | 3 | 5.083 | 5.076 | 0.957 | -7.285 | 17.452 | 8.667 | 8.639 | 0.969 | -13.122 | 30.455 | 2.524 | 6.389 | 1 | -13.211 | 18.259 |
|  |  | 3 | 1 | -0.139 | 4.754 | 1 | -11.723 | 11.445 | -2.933 | 7.961 | 1 | -23.012 | 17.145 | 1.857 | 6.053 | 1 | -13.052 | 16.766 |
|  |  |  | 2 | -5.083 | 5.076 | 0.957 | -17.452 | 7.285 | -8.667 | 8.639 | 0.969 | -30.455 | 13.122 | -2.524 | 6.389 | 1 | -18.259 | 13.211 |
| HDL Cholesterol | 10mg | 1 | 2 | -0.4 | 1.147 | 1 | -3.195 | 2.395 | 0.5 | 2.063 | 1 | -4.704 | 5.704 | -0.727 | 1.39 | 1 | -4.15 | 2.695 |
|  |  |  | 3 | 0 | 1.521 | 1 | -3.706 | 3.706 | 0.5 | 2.759 | 1 | -6.46 | 7.46 | -0.182 | 1.866 | 1 | -4.777 | 4.414 |
|  |  | 2 | 1 | 0.4 | 1.147 | 1 | -2.395 | 3.195 | -0.5 | 2.063 | 1 | -5.704 | 4.704 | 0.727 | 1.39 | 1 | -2.695 | 4.15 |
|  |  |  | 3 | 0.4 | 1.395 | 1 | -2.998 | 3.798 | 1.42E-14 | 2.936 | 1 | -7.405 | 7.405 | 0.545 | 1.513 | 1 | -3.181 | 4.272 |
|  |  | 3 | 1 | 0 | 1.521 | 1 | -3.706 | 3.706 | -0.5 | 2.759 | 1 | -7.46 | 6.46 | 0.182 | 1.866 | 1 | -4.414 | 4.777 |
|  |  |  | 2 | -0.4 | 1.395 | 1 | -3.798 | 2.998 | -1.42E-14 | 2.936 | 1 | -7.405 | 7.405 | -0.545 | 1.513 | 1 | -4.272 | 3.181 |
|  | Placebo | 1 | 2 | -0.424 | 1.094 | 1 | -3.089 | 2.241 | 0.923 | 1.619 | 1 | -3.159 | 5.005 | -1.3 | 1.457 | 1 | -4.89 | 2.29 |
|  |  |  | 3 | -1.273 | 1.45 | 1 | -4.807 | 2.261 | -1.615 | 2.165 | 1 | -7.075 | 3.844 | -1.05 | 1.957 | 1 | -5.87 | 3.77 |
|  |  | 2 | 1 | 0.424 | 1.094 | 1 | -2.241 | 3.089 | -0.923 | 1.619 | 1 | -5.005 | 3.159 | 1.3 | 1.457 | 1 | -2.29 | 4.89 |
|  |  |  | 3 | -0.848 | 1.33 | 1 | -4.089 | 2.392 | -2.538 | 2.303 | 0.835 | -8.347 | 3.271 | 0.25 | 1.587 | 1 | -3.659 | 4.159 |
|  |  | 3 | 1 | 1.273 | 1.45 | 1 | -2.261 | 4.807 | 1.615 | 2.165 | 1 | -3.844 | 7.075 | 1.05 | 1.957 | 1 | -3.77 | 5.87 |
|  |  |  | 2 | 0.848 | 1.33 | 1 | -2.392 | 4.089 | 2.538 | 2.303 | 0.835 | -3.271 | 8.347 | -0.25 | 1.587 | 1 | -4.159 | 3.659 |
|  | 5mg | 1 | 2 | -0.694 | 1.047 | 1 | -3.246 | 1.857 | 0.933 | 1.507 | 1 | -2.867 | 4.734 | -1.857 | 1.422 | 0.59 | -5.36 | 1.646 |
|  |  |  | 3 | -0.833 | 1.389 | 1 | -4.217 | 2.55 | -2.267 | 2.015 | 0.806 | -7.349 | 2.816 | 0.19 | 1.91 | 1 | -4.513 | 4.894 |
|  |  | 2 | 1 | 0.694 | 1.047 | 1 | -1.857 | 3.246 | -0.933 | 1.507 | 1 | -4.734 | 2.867 | 1.857 | 1.422 | 0.59 | -1.646 | 5.36 |
|  |  |  | 3 | -0.139 | 1.273 | 1 | -3.241 | 2.963 | -3.2 | 2.144 | 0.435 | -8.608 | 2.208 | 2.048 | 1.549 | 0.573 | -1.767 | 5.862 |
|  |  | 3 | 1 | 0.833 | 1.389 | 1 | -2.55 | 4.217 | 2.267 | 2.015 | 0.806 | -2.816 | 7.349 | -0.19 | 1.91 | 1 | -4.894 | 4.513 |
|  |  |  | 2 | 0.139 | 1.273 | 1 | -2.963 | 3.241 | 3.2 | 2.144 | 0.435 | -2.208 | 8.608 | -2.048 | 1.549 | 0.573 | -5.862 | 1.767 |
| Triglycerides | 10mg | 1 | 2 | 0.5 | 10.075 | 1 | -24.05 | 25.05 | -22.5 | 23.036 | 1 | -80.603 | 35.603 | 8.864 | 10.457 | 1 | -16.893 | 34.62 |
|  |  |  | 3 | -5.933 | 7.946 | 1 | -25.295 | 13.428 | -37.25 | 17.494 | 0.122 | -81.375 | 6.875 | 5.455 | 8.201 | 1 | -14.744 | 25.653 |
|  |  | 2 | 1 | -0.5 | 10.075 | 1 | -25.05 | 24.05 | 22.5 | 23.036 | 1 | -35.603 | 80.603 | -8.864 | 10.457 | 1 | -34.62 | 16.893 |
|  |  |  | 3 | -6.433 | 7.173 | 1 | -23.911 | 11.044 | -14.75 | 17.727 | 1 | -59.462 | 29.962 | -3.409 | 6.867 | 1 | -20.323 | 13.505 |
|  |  | 3 | 1 | 5.933 | 7.946 | 1 | -13.428 | 25.295 | 37.25 | 17.494 | 0.122 | -6.875 | 81.375 | -5.455 | 8.201 | 1 | -25.653 | 14.744 |
|  |  |  | 2 | 6.433 | 7.173 | 1 | -11.044 | 23.911 | 14.75 | 17.727 | 1 | -29.962 | 59.462 | 3.409 | 6.867 | 1 | -13.505 | 20.323 |
|  | Placebo | 1 | 2 | 10.03 | 9.606 | 0.897 | -13.377 | 33.438 | 13.077 | 18.071 | 1 | -32.503 | 58.656 | 8.05 | 10.968 | 1 | -18.963 | 35.063 |
|  |  |  | 3 | 7.121 | 7.576 | 1 | -11.339 | 25.582 | 15.615 | 13.724 | 0.79 | -18.999 | 50.23 | 1.6 | 8.601 | 1 | -19.584 | 22.784 |
|  |  | 2 | 1 | -10.03 | 9.606 | 0.897 | -33.438 | 13.377 | -13.077 | 18.071 | 1 | -58.656 | 32.503 | -8.05 | 10.968 | 1 | -35.063 | 18.963 |
|  |  |  | 3 | -2.909 | 6.839 | 1 | -19.573 | 13.755 | 2.538 | 13.906 | 1 | -32.536 | 37.613 | -6.45 | 7.203 | 1 | -24.19 | 11.29 |
|  |  | 3 | 1 | -7.121 | 7.576 | 1 | -25.582 | 11.339 | -15.615 | 13.724 | 0.79 | -50.23 | 18.999 | -1.6 | 8.601 | 1 | -22.784 | 19.584 |
|  |  |  | 2 | 2.909 | 6.839 | 1 | -13.755 | 19.573 | -2.538 | 13.906 | 1 | -37.613 | 32.536 | 6.45 | 7.203 | 1 | -11.29 | 24.19 |
|  | 5mg | 1 | 2 | -13.056 | 9.197 | 0.477 | -35.467 | 9.356 | -21.733 | 16.823 | 0.616 | -64.165 | 20.699 | -6.857 | 10.704 | 1 | -33.219 | 19.505 |
|  |  |  | 3 | -1.722 | 7.254 | 1 | -19.397 | 15.952 | -2.6 | 12.776 | 1 | -34.824 | 29.624 | -1.095 | 8.394 | 1 | -21.769 | 19.578 |
|  |  | 2 | 1 | 13.056 | 9.197 | 0.477 | -9.356 | 35.467 | 21.733 | 16.823 | 0.616 | -20.699 | 64.165 | 6.857 | 10.704 | 1 | -19.505 | 33.219 |
|  |  |  | 3 | 11.333 | 6.548 | 0.26 | -4.621 | 27.288 | 19.133 | 12.946 | 0.447 | -13.519 | 51.786 | 5.762 | 7.029 | 1 | -11.55 | 23.074 |
|  |  | 3 | 1 | 1.722 | 7.254 | 1 | -15.952 | 19.397 | 2.6 | 12.776 | 1 | -29.624 | 34.824 | 1.095 | 8.394 | 1 | -19.578 | 21.769 |
|  |  |  | 2 | -11.333 | 6.548 | 0.26 | -27.288 | 4.621 | -19.133 | 12.946 | 0.447 | -51.786 | 13.519 | -5.762 | 7.029 | 1 | -23.074 | 11.55 |
| LDL Cholesterol | 10mg | 1 | 2 | -2.333 | 4.03 | 1 | -12.154 | 7.487 | 1.125 | 6.98 | 1 | -16.481 | 18.731 | -3.591 | 5.054 | 1 | -16.038 | 8.856 |
|  |  |  | 3 | 3.533 | 4.968 | 1 | -8.573 | 15.64 | 1.375 | 10.258 | 1 | -24.499 | 27.249 | 4.318 | 5.693 | 1 | -9.702 | 18.339 |
|  |  | 2 | 1 | 2.333 | 4.03 | 1 | -7.487 | 12.154 | -1.125 | 6.98 | 1 | -18.731 | 16.481 | 3.591 | 5.054 | 1 | -8.856 | 16.038 |
|  |  |  | 3 | 5.867 | 5.173 | 0.779 | -6.738 | 18.471 | 0.25 | 11.533 | 1 | -28.839 | 29.339 | 7.909 | 5.554 | 0.479 | -5.769 | 21.588 |
|  |  | 3 | 1 | -3.533 | 4.968 | 1 | -15.64 | 8.573 | -1.375 | 10.258 | 1 | -27.249 | 24.499 | -4.318 | 5.693 | 1 | -18.339 | 9.702 |
|  |  |  | 2 | -5.867 | 5.173 | 0.779 | -18.471 | 6.738 | -0.25 | 11.533 | 1 | -29.339 | 28.839 | -7.909 | 5.554 | 0.479 | -21.588 | 5.769 |
|  | Placebo | 1 | 2 | 9.394* | 3.843 | 0.049 | 0.031 | 18.757 | 9.462 | 5.476 | 0.28 | -4.35 | 23.273 | 9.35 | 5.3 | 0.248 | -3.704 | 22.404 |
|  |  |  | 3 | 10.364 | 4.737 | 0.093 | -1.179 | 21.906 | 12.615 | 8.047 | 0.38 | -7.682 | 32.912 | 8.9 | 5.97 | 0.424 | -5.805 | 23.605 |
|  |  | 2 | 1 | -9.394* | 3.843 | 0.049 | -18.757 | -0.031 | -9.462 | 5.476 | 0.28 | -23.273 | 4.35 | -9.35 | 5.3 | 0.248 | -22.404 | 3.704 |
|  |  |  | 3 | 0.97 | 4.932 | 1 | -11.048 | 12.987 | 3.154 | 9.047 | 1 | -19.666 | 25.973 | -0.45 | 5.825 | 1 | -14.796 | 13.896 |
|  |  | 3 | 1 | -10.364 | 4.737 | 0.093 | -21.906 | 1.179 | -12.615 | 8.047 | 0.38 | -32.912 | 7.682 | -8.9 | 5.97 | 0.424 | -23.605 | 5.805 |
|  |  |  | 2 | -0.97 | 4.932 | 1 | -12.987 | 11.048 | -3.154 | 9.047 | 1 | -25.973 | 19.666 | 0.45 | 5.825 | 1 | -13.896 | 14.796 |
|  | 5mg | 1 | 2 | -2.889 | 3.679 | 1 | -11.854 | 6.076 | -4.6 | 5.098 | 1 | -17.458 | 8.258 | -1.667 | 5.172 | 1 | -14.406 | 11.073 |
|  |  |  | 3 | 1.25 | 4.535 | 1 | -9.801 | 12.301 | 5.067 | 7.492 | 1 | -13.829 | 23.962 | -1.476 | 5.827 | 1 | -15.827 | 12.874 |
|  |  | 2 | 1 | 2.889 | 3.679 | 1 | -6.076 | 11.854 | 4.6 | 5.098 | 1 | -8.258 | 17.458 | 1.667 | 5.172 | 1 | -11.073 | 14.406 |
|  |  |  | 3 | 4.139 | 4.722 | 1 | -7.367 | 15.645 | 9.667 | 8.423 | 0.778 | -11.577 | 30.911 | 0.19 | 5.684 | 1 | -13.81 | 14.191 |
|  |  | 3 | 1 | -1.25 | 4.535 | 1 | -12.301 | 9.801 | -5.067 | 7.492 | 1 | -23.962 | 13.829 | 1.476 | 5.827 | 1 | -12.874 | 15.827 |
|  |  |  | 2 | -4.139 | 4.722 | 1 | -15.645 | 7.367 | -9.667 | 8.423 | 0.778 | -30.911 | 11.577 | -0.19 | 5.684 | 1 | -14.191 | 13.81 |
| CHOL/HDLC Ratio | 10mg | 1 | 2 | 0.047 | 0.09 | 1 | -0.172 | 0.265 | -0.05 | 0.14 | 1 | -0.402 | 0.302 | 0.082 | 0.116 | 1 | -0.204 | 0.367 |
|  |  |  | 3 | 0.117 | 0.124 | 1 | -0.184 | 0.418 | -0.05 | 0.193 | 1 | -0.536 | 0.436 | 0.177 | 0.159 | 0.804 | -0.213 | 0.568 |
|  |  | 2 | 1 | -0.047 | 0.09 | 1 | -0.265 | 0.172 | 0.05 | 0.14 | 1 | -0.302 | 0.402 | -0.082 | 0.116 | 1 | -0.367 | 0.204 |
|  |  |  | 3 | 0.07 | 0.108 | 1 | -0.194 | 0.334 | -4.44E-16 | 0.208 | 1 | -0.524 | 0.524 | 0.095 | 0.126 | 1 | -0.214 | 0.405 |
|  |  | 3 | 1 | -0.117 | 0.124 | 1 | -0.418 | 0.184 | 0.05 | 0.193 | 1 | -0.436 | 0.536 | -0.177 | 0.159 | 0.804 | -0.568 | 0.213 |
|  |  |  | 2 | -0.07 | 0.108 | 1 | -0.334 | 0.194 | 4.44E-16 | 0.208 | 1 | -0.524 | 0.524 | -0.095 | 0.126 | 1 | -0.405 | 0.214 |
|  | Placebo | 1 | 2 | 0.206 | 0.086 | 0.054 | -0.002 | 0.415 | 0.138 | 0.11 | 0.646 | -0.138 | 0.415 | 0.25 | 0.122 | 0.132 | -0.049 | 0.549 |
|  |  |  | 3 | 0.227 | 0.118 | 0.17 | -0.06 | 0.514 | 0.246 | 0.151 | 0.339 | -0.135 | 0.628 | 0.215 | 0.166 | 0.603 | -0.195 | 0.625 |
|  |  | 2 | 1 | -0.206 | 0.086 | 0.054 | -0.415 | 0.002 | -0.138 | 0.11 | 0.646 | -0.415 | 0.138 | -0.25 | 0.122 | 0.132 | -0.549 | 0.049 |
|  |  |  | 3 | 0.021 | 0.103 | 1 | -0.231 | 0.273 | 0.108 | 0.163 | 1 | -0.303 | 0.519 | -0.035 | 0.132 | 1 | -0.36 | 0.29 |
|  |  | 3 | 1 | -0.227 | 0.118 | 0.17 | -0.514 | 0.06 | -0.246 | 0.151 | 0.339 | -0.628 | 0.135 | -0.215 | 0.166 | 0.603 | -0.625 | 0.195 |
|  |  |  | 2 | -0.021 | 0.103 | 1 | -0.273 | 0.231 | -0.108 | 0.163 | 1 | -0.519 | 0.303 | 0.035 | 0.132 | 1 | -0.29 | 0.36 |
|  | 5mg | 1 | 2 | -0.031 | 0.082 | 1 | -0.23 | 0.169 | -0.1 | 0.102 | 1 | -0.357 | 0.157 | 0.019 | 0.119 | 1 | -0.273 | 0.311 |
|  |  |  | 3 | 0.028 | 0.113 | 1 | -0.247 | 0.303 | 0.187 | 0.141 | 0.582 | -0.168 | 0.542 | -0.086 | 0.162 | 1 | -0.485 | 0.314 |
|  |  | 2 | 1 | 0.031 | 0.082 | 1 | -0.169 | 0.23 | 0.1 | 0.102 | 1 | -0.157 | 0.357 | -0.019 | 0.119 | 1 | -0.311 | 0.273 |
|  |  |  | 3 | 0.058 | 0.099 | 1 | -0.183 | 0.299 | 0.287 | 0.152 | 0.203 | -0.096 | 0.669 | -0.105 | 0.129 | 1 | -0.422 | 0.212 |
|  |  | 3 | 1 | -0.028 | 0.113 | 1 | -0.303 | 0.247 | -0.187 | 0.141 | 0.582 | -0.542 | 0.168 | 0.086 | 0.162 | 1 | -0.314 | 0.485 |
|  |  |  | 2 | -0.058 | 0.099 | 1 | -0.299 | 0.183 | -0.287 | 0.152 | 0.203 | -0.669 | 0.096 | 0.105 | 0.129 | 1 | -0.212 | 0.422 |
| Non HDL Cholesterol | 10mg | 1 | 2 | -2.133 | 5.373 | 1 | -15.225 | 10.959 | -1.75 | 7.193 | 1 | -19.892 | 16.392 | -2.273 | 7.225 | 1 | -20.068 | 15.522 |
|  |  |  | 3 | 3.5 | 6.275 | 1 | -11.791 | 18.791 | -1.125 | 10.824 | 1 | -28.425 | 26.175 | 5.182 | 7.722 | 1 | -13.837 | 24.201 |
|  |  | 2 | 1 | 2.133 | 5.373 | 1 | -10.959 | 15.225 | 1.75 | 7.193 | 1 | -16.392 | 19.892 | 2.273 | 7.225 | 1 | -15.522 | 20.068 |
|  |  |  | 3 | 5.633 | 5.391 | 0.896 | -7.502 | 18.768 | 0.625 | 11.853 | 1 | -29.272 | 30.522 | 7.455 | 5.839 | 0.62 | -6.928 | 21.837 |
|  |  | 3 | 1 | -3.5 | 6.275 | 1 | -18.791 | 11.791 | 1.125 | 10.824 | 1 | -26.175 | 28.425 | -5.182 | 7.722 | 1 | -24.201 | 13.837 |
|  |  |  | 2 | -5.633 | 5.391 | 0.896 | -18.768 | 7.502 | -0.625 | 11.853 | 1 | -30.522 | 29.272 | -7.455 | 5.839 | 0.62 | -21.837 | 6.928 |
|  | Placebo | 1 | 2 | 10.758 | 5.123 | 0.115 | -1.725 | 23.24 | 11.231 | 5.643 | 0.165 | -3.001 | 25.463 | 10.45 | 7.578 | 0.519 | -8.214 | 29.114 |
|  |  |  | 3 | 11.424 | 5.983 | 0.178 | -3.155 | 26.004 | 14.769 | 8.491 | 0.274 | -6.647 | 36.185 | 9.25 | 8.099 | 0.774 | -10.697 | 29.197 |
|  |  | 2 | 1 | -10.758 | 5.123 | 0.115 | -23.24 | 1.725 | -11.231 | 5.643 | 0.165 | -25.463 | 3.001 | -10.45 | 7.578 | 0.519 | -29.114 | 8.214 |
|  |  |  | 3 | 0.667 | 5.14 | 1 | -11.857 | 13.19 | 3.538 | 9.299 | 1 | -19.915 | 26.991 | -1.2 | 6.124 | 1 | -16.284 | 13.884 |
|  |  | 3 | 1 | -11.424 | 5.983 | 0.178 | -26.004 | 3.155 | -14.769 | 8.491 | 0.274 | -36.185 | 6.647 | -9.25 | 8.099 | 0.774 | -29.197 | 10.697 |
|  |  |  | 2 | -0.667 | 5.14 | 1 | -13.19 | 11.857 | -3.538 | 9.299 | 1 | -26.991 | 19.915 | 1.2 | 6.124 | 1 | -13.884 | 16.284 |
|  | 5mg | 1 | 2 | -11.361 | 4.905 | 0.068 | -23.312 | 0.59 | -6.667 | 5.253 | 0.64 | -19.916 | 6.583 | -14.714 | 7.395 | 0.154 | -32.928 | 3.5 |
|  |  |  | 3 | -6.139 | 5.729 | 0.86 | -20.098 | 7.82 | 5.2 | 7.905 | 1 | -14.737 | 25.137 | -14.238 | 7.904 | 0.23 | -33.705 | 5.228 |
|  |  | 2 | 1 | 11.361 | 4.905 | 0.068 | -0.59 | 23.312 | 6.667 | 5.253 | 0.64 | -6.583 | 19.916 | 14.714 | 7.395 | 0.154 | -3.5 | 32.928 |
|  |  |  | 3 | 5.222 | 4.921 | 0.874 | -6.768 | 17.213 | 11.867 | 8.657 | 0.539 | -9.967 | 33.7 | 0.476 | 5.977 | 1 | -14.244 | 15.197 |
|  |  | 3 | 1 | 6.139 | 5.729 | 0.86 | -7.82 | 20.098 | -5.2 | 7.905 | 1 | -25.137 | 14.737 | 14.238 | 7.904 | 0.23 | -5.228 | 33.705 |
|  |  |  | 2 | -5.222 | 4.921 | 0.874 | -17.213 | 6.768 | -11.867 | 8.657 | 0.539 | -33.7 | 9.967 | -0.476 | 5.977 | 1 | -15.197 | 14.244 |
| VLDL Cholesterol | 10mg | 1 | 2 | -0.933 | 3.451 | 1 | -9.342 | 7.475 | -0.625 | 9.738 | 1 | -25.186 | 23.936 | -1.045 | 2.567 | 1 | -7.368 | 5.277 |
|  |  |  | 3 | 1.033 | 4.172 | 1 | -9.131 | 11.198 | 0.5 | 11.461 | 1 | -28.407 | 29.407 | 1.227 | 3.34 | 1 | -6.998 | 9.453 |
|  |  | 2 | 1 | 0.933 | 3.451 | 1 | -7.475 | 9.342 | 0.625 | 9.738 | 1 | -23.936 | 25.186 | 1.045 | 2.567 | 1 | -5.277 | 7.368 |
|  |  |  | 3 | 1.967 | 3.633 | 1 | -6.885 | 10.818 | 1.125 | 7.114 | 1 | -16.817 | 19.067 | 2.273 | 4.209 | 1 | -8.095 | 12.64 |
|  |  | 3 | 1 | -1.033 | 4.172 | 1 | -11.198 | 9.131 | -0.5 | 11.461 | 1 | -29.407 | 28.407 | -1.227 | 3.34 | 1 | -9.453 | 6.998 |
|  |  |  | 2 | -1.967 | 3.633 | 1 | -10.818 | 6.885 | -1.125 | 7.114 | 1 | -19.067 | 16.817 | -2.273 | 4.209 | 1 | -12.64 | 8.095 |
|  | Placebo | 1 | 2 | 1.121 | 3.29 | 1 | -6.896 | 9.138 | 5.692 | 7.639 | 1 | -13.575 | 24.96 | -1.85 | 2.692 | 1 | -8.481 | 4.781 |
|  |  |  | 3 | -1.455 | 3.977 | 1 | -11.146 | 8.237 | -3.923 | 8.991 | 1 | -26.599 | 18.753 | 0.15 | 3.503 | 1 | -8.477 | 8.777 |
|  |  | 2 | 1 | -1.121 | 3.29 | 1 | -9.138 | 6.896 | -5.692 | 7.639 | 1 | -24.96 | 13.575 | 1.85 | 2.692 | 1 | -4.781 | 8.481 |
|  |  |  | 3 | -2.576 | 3.464 | 1 | -11.015 | 5.864 | -9.615 | 5.58 | 0.283 | -23.69 | 4.46 | 2 | 4.415 | 1 | -8.874 | 12.874 |
|  |  | 3 | 1 | 1.455 | 3.977 | 1 | -8.237 | 11.146 | 3.923 | 8.991 | 1 | -18.753 | 26.599 | -0.15 | 3.503 | 1 | -8.777 | 8.477 |
|  |  |  | 2 | 2.576 | 3.464 | 1 | -5.864 | 11.015 | 9.615 | 5.58 | 0.283 | -4.46 | 23.69 | -2 | 4.415 | 1 | -12.874 | 8.874 |
|  | 5mg | 1 | 2 | 0.111 | 3.15 | 1 | -7.565 | 7.787 | 2.2 | 7.112 | 1 | -15.737 | 20.137 | -1.381 | 2.627 | 1 | -7.852 | 5.09 |
|  |  |  | 3 | -2.167 | 3.808 | 1 | -11.446 | 7.112 | 2.6 | 8.37 | 1 | -18.51 | 23.71 | -5.571 | 3.418 | 0.325 | -13.991 | 2.848 |
|  |  | 2 | 1 | -0.111 | 3.15 | 1 | -7.787 | 7.565 | -2.2 | 7.112 | 1 | -20.137 | 15.737 | 1.381 | 2.627 | 1 | -5.09 | 7.852 |
|  |  |  | 3 | -2.278 | 3.316 | 1 | -10.358 | 5.802 | 0.4 | 5.195 | 1 | -12.703 | 13.503 | -4.19 | 4.309 | 1 | -14.802 | 6.421 |
|  |  | 3 | 1 | 2.167 | 3.808 | 1 | -7.112 | 11.446 | -2.6 | 8.37 | 1 | -23.71 | 18.51 | 5.571 | 3.418 | 0.325 | -2.848 | 13.991 |
|  |  |  | 2 | 2.278 | 3.316 | 1 | -5.802 | 10.358 | -0.4 | 5.195 | 1 | -13.503 | 12.703 | 4.19 | 4.309 | 1 | -6.421 | 14.802 |
| hsCRP | 10mg | 1 | 2 | -0.581 | 0.502 | 0.752 | -1.808 | 0.646 | -0.725 | 0.697 | 0.923 | -2.507 | 1.057 | -0.517 | 0.674 | 1 | -2.185 | 1.151 |
|  |  |  | 3 | 0.069 | 0.543 | 1 | -1.258 | 1.396 | 0.175 | 0.886 | 1 | -2.093 | 2.443 | 0.022 | 0.695 | 1 | -1.699 | 1.743 |
|  |  | 2 | 1 | 0.581 | 0.502 | 0.752 | -0.646 | 1.808 | 0.725 | 0.697 | 0.923 | -1.057 | 2.507 | 0.517 | 0.674 | 1 | -1.151 | 2.185 |
|  |  |  | 3 | 0.65 | 0.584 | 0.807 | -0.778 | 2.078 | 0.9 | 1.103 | 1 | -1.923 | 3.723 | 0.539 | 0.703 | 1 | -1.203 | 2.28 |
|  |  | 3 | 1 | -0.069 | 0.543 | 1 | -1.396 | 1.258 | -0.175 | 0.886 | 1 | -2.443 | 2.093 | -0.022 | 0.695 | 1 | -1.743 | 1.699 |
|  |  |  | 2 | -0.65 | 0.584 | 0.807 | -2.078 | 0.778 | -0.9 | 1.103 | 1 | -3.723 | 1.923 | -0.539 | 0.703 | 1 | -2.28 | 1.203 |
|  | Placebo | 1 | 2 | -0.167 | 0.492 | 1 | -1.371 | 1.037 | -0.318 | 0.594 | 1 | -1.838 | 1.202 | -0.063 | 0.715 | 1 | -1.832 | 1.707 |
|  |  |  | 3 | -0.341 | 0.533 | 1 | -1.643 | 0.962 | -0.691 | 0.756 | 1 | -2.625 | 1.244 | -0.1 | 0.737 | 1 | -1.926 | 1.726 |
|  |  | 2 | 1 | 0.167 | 0.492 | 1 | -1.037 | 1.371 | 0.318 | 0.594 | 1 | -1.202 | 1.838 | 0.063 | 0.715 | 1 | -1.707 | 1.832 |
|  |  |  | 3 | -0.174 | 0.573 | 1 | -1.575 | 1.227 | -0.373 | 0.941 | 1 | -2.78 | 2.034 | -0.037 | 0.746 | 1 | -1.885 | 1.81 |
|  |  | 3 | 1 | 0.341 | 0.533 | 1 | -0.962 | 1.643 | 0.691 | 0.756 | 1 | -1.244 | 2.625 | 0.1 | 0.737 | 1 | -1.726 | 1.926 |
|  |  |  | 2 | 0.174 | 0.573 | 1 | -1.227 | 1.575 | 0.373 | 0.941 | 1 | -2.034 | 2.78 | 0.037 | 0.746 | 1 | -1.81 | 1.885 |
|  | 5mg | 1 | 2 | 0.557 | 0.467 | 0.711 | -0.586 | 1.699 | -0.04 | 0.623 | 1 | -1.634 | 1.554 | 0.855 | 0.639 | 0.561 | -0.727 | 2.437 |
|  |  |  | 3 | -0.353 | 0.505 | 1 | -1.589 | 0.882 | -0.8 | 0.793 | 0.967 | -2.829 | 1.229 | -0.13 | 0.66 | 1 | -1.763 | 1.503 |
|  |  | 2 | 1 | -0.557 | 0.467 | 0.711 | -1.699 | 0.586 | 0.04 | 0.623 | 1 | -1.554 | 1.634 | -0.855 | 0.639 | 0.561 | -2.437 | 0.727 |
|  |  |  | 3 | -0.91 | 0.544 | 0.294 | -2.239 | 0.419 | -0.76 | 0.987 | 1 | -3.285 | 1.765 | -0.985 | 0.667 | 0.438 | -2.637 | 0.667 |
|  |  | 3 | 1 | 0.353 | 0.505 | 1 | -0.882 | 1.589 | 0.8 | 0.793 | 0.967 | -1.229 | 2.829 | 0.13 | 0.66 | 1 | -1.503 | 1.763 |
|  |  |  | 2 | 0.91 | 0.544 | 0.294 | -0.419 | 2.239 | 0.76 | 0.987 | 1 | -1.765 | 3.285 | 0.985 | 0.667 | 0.438 | -0.667 | 2.637 |
| Glucose | 10mg | 1 | 2 | 1.346 | 1.966 | 1 | -3.462 | 6.154 | -3.375 | 3.958 | 1 | -13.503 | 6.753 | 3.444 | 2.126 | 0.334 | -1.819 | 8.708 |
|  |  |  | 3 | 0.577 | 1.829 | 1 | -3.897 | 5.05 | -5.5 | 3.117 | 0.268 | -13.476 | 2.476 | 3.278 | 2.156 | 0.404 | -2.06 | 8.616 |
|  |  | 2 | 1 | -1.346 | 1.966 | 1 | -6.154 | 3.462 | 3.375 | 3.958 | 1 | -6.753 | 13.503 | -3.444 | 2.126 | 0.334 | -8.708 | 1.819 |
|  |  |  | 3 | -0.769 | 1.579 | 1 | -4.629 | 3.091 | -2.125 | 2.742 | 1 | -9.141 | 4.891 | -0.167 | 1.954 | 1 | -5.005 | 4.671 |
|  |  | 3 | 1 | -0.577 | 1.829 | 1 | -5.05 | 3.897 | 5.5 | 3.117 | 0.268 | -2.476 | 13.476 | -3.278 | 2.156 | 0.404 | -8.616 | 2.06 |
|  |  |  | 2 | 0.769 | 1.579 | 1 | -3.091 | 4.629 | 2.125 | 2.742 | 1 | -4.891 | 9.141 | 0.167 | 1.954 | 1 | -4.671 | 5.005 |
|  | Placebo | 1 | 2 | 1.37 | 1.929 | 1 | -3.348 | 6.089 | 5.818 | 3.375 | 0.29 | -2.819 | 14.456 | -1.688 | 2.255 | 1 | -7.27 | 3.895 |
|  |  |  | 3 | 0.889 | 1.795 | 1 | -3.501 | 5.279 | 4.273 | 2.658 | 0.36 | -2.529 | 11.074 | -1.438 | 2.287 | 1 | -7.1 | 4.225 |
|  |  | 2 | 1 | -1.37 | 1.929 | 1 | -6.089 | 3.348 | -5.818 | 3.375 | 0.29 | -14.456 | 2.819 | 1.688 | 2.255 | 1 | -3.895 | 7.27 |
|  |  |  | 3 | -0.481 | 1.549 | 1 | -4.269 | 3.306 | -1.545 | 2.338 | 1 | -7.529 | 4.438 | 0.25 | 2.073 | 1 | -4.882 | 5.382 |
|  |  | 3 | 1 | -0.889 | 1.795 | 1 | -5.279 | 3.501 | -4.273 | 2.658 | 0.36 | -11.074 | 2.529 | 1.438 | 2.287 | 1 | -4.225 | 7.1 |
|  |  |  | 2 | 0.481 | 1.549 | 1 | -3.306 | 4.269 | 1.545 | 2.338 | 1 | -4.438 | 7.529 | -0.25 | 2.073 | 1 | -5.382 | 4.882 |
|  | 5mg | 1 | 2 | 2.2 | 1.83 | 0.699 | -2.276 | 6.676 | 2.9 | 3.54 | 1 | -6.159 | 11.959 | 1.85 | 2.017 | 1 | -3.144 | 6.844 |
|  |  |  | 3 | 0.067 | 1.703 | 1 | -4.098 | 4.231 | -1.4 | 2.788 | 1 | -8.534 | 5.734 | 0.8 | 2.046 | 1 | -4.264 | 5.864 |
|  |  | 2 | 1 | -2.2 | 1.83 | 0.699 | -6.676 | 2.276 | -2.9 | 3.54 | 1 | -11.959 | 6.159 | -1.85 | 2.017 | 1 | -6.844 | 3.144 |
|  |  |  | 3 | -2.133 | 1.47 | 0.451 | -5.727 | 1.46 | -4.3 | 2.452 | 0.274 | -10.576 | 1.976 | -1.05 | 1.854 | 1 | -5.64 | 3.54 |
|  |  | 3 | 1 | -0.067 | 1.703 | 1 | -4.231 | 4.098 | 1.4 | 2.788 | 1 | -5.734 | 8.534 | -0.8 | 2.046 | 1 | -5.864 | 4.264 |
|  |  |  | 2 | 2.133 | 1.47 | 0.451 | -1.46 | 5.727 | 4.3 | 2.452 | 0.274 | -1.976 | 10.576 | 1.05 | 1.854 | 1 | -3.54 | 5.64 |
| BUN | 10mg | 1 | 2 | 0.385 | 0.716 | 1 | -1.365 | 2.135 | 0.375 | 1.424 | 1 | -3.268 | 4.018 | 0.389 | 0.831 | 1 | -1.668 | 2.446 |
|  |  |  | 3 | -1.692 | 0.694 | 0.051 | -3.39 | 0.005 | -0.5 | 1.259 | 1 | -3.722 | 2.722 | -2.222* | 0.833 | 0.031 | -4.283 | -0.161 |
|  |  | 2 | 1 | -0.385 | 0.716 | 1 | -2.135 | 1.365 | -0.375 | 1.424 | 1 | -4.018 | 3.268 | -0.389 | 0.831 | 1 | -2.446 | 1.668 |
|  |  |  | 3 | -2.077* | 0.733 | 0.017 | -3.869 | -0.285 | -0.875 | 1.148 | 1 | -3.812 | 2.062 | -2.611* | 0.934 | 0.022 | -4.922 | -0.3 |
|  |  | 3 | 1 | 1.692 | 0.694 | 0.051 | -0.005 | 3.39 | 0.5 | 1.259 | 1 | -2.722 | 3.722 | 2.222* | 0.833 | 0.031 | 0.161 | 4.283 |
|  |  |  | 2 | 2.077* | 0.733 | 0.017 | 0.285 | 3.869 | 0.875 | 1.148 | 1 | -2.062 | 3.812 | 2.611* | 0.934 | 0.022 | 0.3 | 4.922 |
|  | Placebo | 1 | 2 | -0.444 | 0.702 | 1 | -2.162 | 1.273 | -0.818 | 1.214 | 1 | -3.925 | 2.288 | -0.188 | 0.881 | 1 | -2.369 | 1.994 |
|  |  |  | 3 | -0.148 | 0.681 | 1 | -1.814 | 1.518 | -1.091 | 1.074 | 0.957 | -3.839 | 1.657 | 0.5 | 0.883 | 1 | -1.686 | 2.686 |
|  |  | 2 | 1 | 0.444 | 0.702 | 1 | -1.273 | 2.162 | 0.818 | 1.214 | 1 | -2.288 | 3.925 | 0.188 | 0.881 | 1 | -1.994 | 2.369 |
|  |  |  | 3 | 0.296 | 0.719 | 1 | -1.462 | 2.055 | -0.273 | 0.979 | 1 | -2.777 | 2.232 | 0.688 | 0.99 | 1 | -1.764 | 3.139 |
|  |  | 3 | 1 | 0.148 | 0.681 | 1 | -1.518 | 1.814 | 1.091 | 1.074 | 0.957 | -1.657 | 3.839 | -0.5 | 0.883 | 1 | -2.686 | 1.686 |
|  |  |  | 2 | -0.296 | 0.719 | 1 | -2.055 | 1.462 | 0.273 | 0.979 | 1 | -2.232 | 2.777 | -0.688 | 0.99 | 1 | -3.139 | 1.764 |
|  | 5mg | 1 | 2 | -0.7 | 0.666 | 0.89 | -2.329 | 0.929 | -3.55E-15 | 1.273 | 1 | -3.258 | 3.258 | -1.05 | 0.788 | 0.566 | -3.001 | 0.901 |
|  |  |  | 3 | -0.433 | 0.646 | 1 | -2.014 | 1.147 | -0.9 | 1.126 | 1 | -3.782 | 1.982 | -0.2 | 0.79 | 1 | -2.155 | 1.755 |
|  |  | 2 | 1 | 0.7 | 0.666 | 0.89 | -0.929 | 2.329 | 3.55E-15 | 1.273 | 1 | -3.258 | 3.258 | 1.05 | 0.788 | 0.566 | -0.901 | 3.001 |
|  |  |  | 3 | 0.267 | 0.682 | 1 | -1.402 | 1.935 | -0.9 | 1.026 | 1 | -3.527 | 1.727 | 0.85 | 0.886 | 1 | -1.343 | 3.043 |
|  |  | 3 | 1 | 0.433 | 0.646 | 1 | -1.147 | 2.014 | 0.9 | 1.126 | 1 | -1.982 | 3.782 | 0.2 | 0.79 | 1 | -1.755 | 2.155 |
|  |  |  | 2 | -0.267 | 0.682 | 1 | -1.935 | 1.402 | 0.9 | 1.026 | 1 | -1.727 | 3.527 | -0.85 | 0.886 | 1 | -3.043 | 1.343 |
| Creatinine | 10mg | 1 | 2 | 0.01 | 0.017 | 1 | -0.033 | 0.052 | 0.004 | 0.022 | 1 | -0.053 | 0.06 | 0.012 | 0.023 | 1 | -0.045 | 0.069 |
|  |  |  | 3 | -0.004 | 0.018 | 1 | -0.047 | 0.04 | -0.001 | 0.025 | 1 | -0.065 | 0.063 | -0.005 | 0.024 | 1 | -0.063 | 0.053 |
|  |  | 2 | 1 | -0.01 | 0.017 | 1 | -0.052 | 0.033 | -0.004 | 0.022 | 1 | -0.06 | 0.053 | -0.012 | 0.023 | 1 | -0.069 | 0.045 |
|  |  |  | 3 | -0.013 | 0.02 | 1 | -0.062 | 0.035 | -0.005 | 0.024 | 1 | -0.066 | 0.056 | -0.017 | 0.027 | 1 | -0.084 | 0.05 |
|  |  | 3 | 1 | 0.004 | 0.018 | 1 | -0.04 | 0.047 | 0.001 | 0.025 | 1 | -0.063 | 0.065 | 0.005 | 0.024 | 1 | -0.053 | 0.063 |
|  |  |  | 2 | 0.013 | 0.02 | 1 | -0.035 | 0.062 | 0.005 | 0.024 | 1 | -0.056 | 0.066 | 0.017 | 0.027 | 1 | -0.05 | 0.084 |
|  | Placebo | 1 | 2 | 0.018 | 0.017 | 0.862 | -0.023 | 0.06 | -0.021 | 0.019 | 0.835 | -0.069 | 0.027 | 0.045 | 0.024 | 0.216 | -0.016 | 0.106 |
|  |  |  | 3 | -0.009 | 0.017 | 1 | -0.052 | 0.034 | -0.012 | 0.021 | 1 | -0.067 | 0.043 | -0.007 | 0.025 | 1 | -0.069 | 0.055 |
|  |  | 2 | 1 | -0.018 | 0.017 | 0.862 | -0.06 | 0.023 | 0.021 | 0.019 | 0.835 | -0.027 | 0.069 | -0.045 | 0.024 | 0.216 | -0.106 | 0.016 |
|  |  |  | 3 | -0.027 | 0.02 | 0.511 | -0.075 | 0.021 | 0.009 | 0.02 | 1 | -0.043 | 0.061 | -0.052 | 0.029 | 0.227 | -0.123 | 0.019 |
|  |  | 3 | 1 | 0.009 | 0.017 | 1 | -0.034 | 0.052 | 0.012 | 0.021 | 1 | -0.043 | 0.067 | 0.007 | 0.025 | 1 | -0.055 | 0.069 |
|  |  |  | 2 | 0.027 | 0.02 | 0.511 | -0.021 | 0.075 | -0.009 | 0.02 | 1 | -0.061 | 0.043 | 0.052 | 0.029 | 0.227 | -0.019 | 0.123 |
|  | 5mg | 1 | 2 | -0.024 | 0.016 | 0.402 | -0.064 | 0.015 | -0.024 | 0.02 | 0.709 | -0.075 | 0.027 | -0.024 | 0.022 | 0.806 | -0.079 | 0.03 |
|  |  |  | 3 | -0.013 | 0.017 | 1 | -0.053 | 0.028 | -0.043 | 0.022 | 0.2 | -0.1 | 0.014 | 0.003 | 0.022 | 1 | -0.053 | 0.058 |
|  |  | 2 | 1 | 0.024 | 0.016 | 0.402 | -0.015 | 0.064 | 0.024 | 0.02 | 0.709 | -0.027 | 0.075 | 0.024 | 0.022 | 0.806 | -0.03 | 0.079 |
|  |  |  | 3 | 0.012 | 0.019 | 1 | -0.034 | 0.057 | -0.019 | 0.021 | 1 | -0.074 | 0.036 | 0.027 | 0.026 | 0.888 | -0.036 | 0.09 |
|  |  | 3 | 1 | 0.013 | 0.017 | 1 | -0.028 | 0.053 | 0.043 | 0.022 | 0.2 | -0.014 | 0.1 | -0.003 | 0.022 | 1 | -0.058 | 0.053 |
|  |  |  | 2 | -0.012 | 0.019 | 1 | -0.057 | 0.034 | 0.019 | 0.021 | 1 | -0.036 | 0.074 | -0.027 | 0.026 | 0.888 | -0.09 | 0.036 |
| eGFR | 10mg | 1 | 2 | -1.308 | 1.613 | 1 | -5.253 | 2.637 | -0.25 | 2.357 | 1 | -6.281 | 5.781 | -1.778 | 2.047 | 1 | -6.846 | 3.291 |
|  |  |  | 3 | -0.615 | 1.624 | 1 | -4.586 | 3.355 | -0.875 | 2.889 | 1 | -8.269 | 6.519 | -0.5 | 1.93 | 1 | -5.277 | 4.277 |
|  |  | 2 | 1 | 1.308 | 1.613 | 1 | -2.637 | 5.253 | 0.25 | 2.357 | 1 | -5.781 | 6.281 | 1.778 | 2.047 | 1 | -3.291 | 6.846 |
|  |  |  | 3 | 0.692 | 1.548 | 1 | -3.094 | 4.479 | -0.625 | 2.214 | 1 | -6.291 | 5.041 | 1.278 | 2.006 | 1 | -3.687 | 6.243 |
|  |  | 3 | 1 | 0.615 | 1.624 | 1 | -3.355 | 4.586 | 0.875 | 2.889 | 1 | -6.519 | 8.269 | 0.5 | 1.93 | 1 | -4.277 | 5.277 |
|  |  |  | 2 | -0.692 | 1.548 | 1 | -4.479 | 3.094 | 0.625 | 2.214 | 1 | -5.041 | 6.291 | -1.278 | 2.006 | 1 | -6.243 | 3.687 |
|  | Placebo | 1 | 2 | -2.148 | 1.583 | 0.536 | -6.02 | 1.723 | 2.091 | 2.01 | 0.923 | -3.052 | 7.234 | -5.063 | 2.172 | 0.071 | -10.438 | 0.313 |
|  |  |  | 3 | -1.407 | 1.593 | 1 | -5.303 | 2.489 | -0.182 | 2.464 | 1 | -6.487 | 6.124 | -2.25 | 2.047 | 0.83 | -7.317 | 2.817 |
|  |  | 2 | 1 | 2.148 | 1.583 | 0.536 | -1.723 | 6.02 | -2.091 | 2.01 | 0.923 | -7.234 | 3.052 | 5.063 | 2.172 | 0.071 | -0.313 | 10.438 |
|  |  |  | 3 | 0.741 | 1.52 | 1 | -2.975 | 4.457 | -2.273 | 1.888 | 0.719 | -7.104 | 2.559 | 2.813 | 2.127 | 0.576 | -2.454 | 8.079 |
|  |  | 3 | 1 | 1.407 | 1.593 | 1 | -2.489 | 5.303 | 0.182 | 2.464 | 1 | -6.124 | 6.487 | 2.25 | 2.047 | 0.83 | -2.817 | 7.317 |
|  |  |  | 2 | -0.741 | 1.52 | 1 | -4.457 | 2.975 | 2.273 | 1.888 | 0.719 | -2.559 | 7.104 | -2.813 | 2.127 | 0.576 | -8.079 | 2.454 |
|  | 5mg | 1 | 2 | 0.033 | 1.502 | 1 | -3.639 | 3.706 | 2.1 | 2.108 | 0.985 | -3.294 | 7.494 | -1 | 1.942 | 1 | -5.808 | 3.808 |
|  |  |  | 3 | -0.167 | 1.511 | 1 | -3.863 | 3.529 | 4.3 | 2.584 | 0.324 | -2.313 | 10.913 | -2.4 | 1.831 | 0.587 | -6.932 | 2.132 |
|  |  | 2 | 1 | -0.033 | 1.502 | 1 | -3.706 | 3.639 | -2.1 | 2.108 | 0.985 | -7.494 | 3.294 | 1 | 1.942 | 1 | -3.808 | 5.808 |
|  |  |  | 3 | -0.2 | 1.442 | 1 | -3.725 | 3.325 | 2.2 | 1.98 | 0.83 | -2.868 | 7.268 | -1.4 | 1.903 | 1 | -6.11 | 3.31 |
|  |  | 3 | 1 | 0.167 | 1.511 | 1 | -3.529 | 3.863 | -4.3 | 2.584 | 0.324 | -10.913 | 2.313 | 2.4 | 1.831 | 0.587 | -2.132 | 6.932 |
|  |  |  | 2 | 0.2 | 1.442 | 1 | -3.325 | 3.725 | -2.2 | 1.98 | 0.83 | -7.268 | 2.868 | 1.4 | 1.903 | 1 | -3.31 | 6.11 |
| Sodium | 10mg | 1 | 2 | -0.538 | 0.394 | 0.528 | -1.503 | 0.426 | -1.125 | 0.806 | 0.524 | -3.189 | 0.939 | -0.278 | 0.446 | 1 | -1.381 | 0.826 |
|  |  |  | 3 | 0.038 | 0.359 | 1 | -0.839 | 0.916 | 0.125 | 0.734 | 1 | -1.754 | 2.004 | 0 | 0.4 | 1 | -0.989 | 0.989 |
|  |  | 2 | 1 | 0.538 | 0.394 | 0.528 | -0.426 | 1.503 | 1.125 | 0.806 | 0.524 | -0.939 | 3.189 | 0.278 | 0.446 | 1 | -0.826 | 1.381 |
|  |  |  | 3 | 0.577 | 0.422 | 0.525 | -0.454 | 1.608 | 1.25 | 0.917 | 0.553 | -1.095 | 3.595 | 0.278 | 0.448 | 1 | -0.832 | 1.388 |
|  |  | 3 | 1 | -0.038 | 0.359 | 1 | -0.916 | 0.839 | -0.125 | 0.734 | 1 | -2.004 | 1.754 | 0 | 0.4 | 1 | -0.989 | 0.989 |
|  |  |  | 2 | -0.577 | 0.422 | 0.525 | -1.608 | 0.454 | -1.25 | 0.917 | 0.553 | -3.595 | 1.095 | -0.278 | 0.448 | 1 | -1.388 | 0.832 |
|  | Placebo | 1 | 2 | 0.667 | 0.387 | 0.267 | -0.28 | 1.613 | 1 | 0.688 | 0.474 | -0.76 | 2.76 | 0.438 | 0.473 | 1 | -0.733 | 1.608 |
|  |  |  | 3 | 0.333 | 0.352 | 1 | -0.528 | 1.195 | 0.818 | 0.626 | 0.608 | -0.784 | 2.42 | -2.84E-14 | 0.424 | 1 | -1.049 | 1.049 |
|  |  | 2 | 1 | -0.667 | 0.387 | 0.267 | -1.613 | 0.28 | -1 | 0.688 | 0.474 | -2.76 | 0.76 | -0.438 | 0.473 | 1 | -1.608 | 0.733 |
|  |  |  | 3 | -0.333 | 0.414 | 1 | -1.345 | 0.678 | -0.182 | 0.782 | 1 | -2.182 | 1.818 | -0.438 | 0.476 | 1 | -1.615 | 0.74 |
|  |  | 3 | 1 | -0.333 | 0.352 | 1 | -1.195 | 0.528 | -0.818 | 0.626 | 0.608 | -2.42 | 0.784 | 2.84E-14 | 0.424 | 1 | -1.049 | 1.049 |
|  |  |  | 2 | 0.333 | 0.414 | 1 | -0.678 | 1.345 | 0.182 | 0.782 | 1 | -1.818 | 2.182 | 0.438 | 0.476 | 1 | -0.74 | 1.615 |
|  | 5mg | 1 | 2 | 0.4 | 0.367 | 0.838 | -0.498 | 1.298 | 0.4 | 0.721 | 1 | -1.446 | 2.246 | 0.4 | 0.423 | 1 | -0.647 | 1.447 |
|  |  |  | 3 | 0.633 | 0.334 | 0.185 | -0.184 | 1.45 | 1.2 | 0.657 | 0.237 | -0.48 | 2.88 | 0.35 | 0.379 | 1 | -0.588 | 1.288 |
|  |  | 2 | 1 | -0.4 | 0.367 | 0.838 | -1.298 | 0.498 | -0.4 | 0.721 | 1 | -2.246 | 1.446 | -0.4 | 0.423 | 1 | -1.447 | 0.647 |
|  |  |  | 3 | 0.233 | 0.392 | 1 | -0.726 | 1.193 | 0.8 | 0.82 | 1 | -1.298 | 2.898 | -0.05 | 0.425 | 1 | -1.103 | 1.003 |
|  |  | 3 | 1 | -0.633 | 0.334 | 0.185 | -1.45 | 0.184 | -1.2 | 0.657 | 0.237 | -2.88 | 0.48 | -0.35 | 0.379 | 1 | -1.288 | 0.588 |
|  |  |  | 2 | -0.233 | 0.392 | 1 | -1.193 | 0.726 | -0.8 | 0.82 | 1 | -2.898 | 1.298 | 0.05 | 0.425 | 1 | -1.003 | 1.103 |
| Potassium | 10mg | 1 | 2 | 0.1 | 0.08 | 0.652 | -0.097 | 0.297 | 0.037 | 0.124 | 1 | -0.28 | 0.355 | 0.128 | 0.103 | 0.662 | -0.127 | 0.383 |
|  |  |  | 3 | 0.054 | 0.083 | 1 | -0.15 | 0.258 | -0.025 | 0.102 | 1 | -0.286 | 0.236 | 0.089 | 0.113 | 1 | -0.191 | 0.369 |
|  |  | 2 | 1 | -0.1 | 0.08 | 0.652 | -0.297 | 0.097 | -0.037 | 0.124 | 1 | -0.355 | 0.28 | -0.128 | 0.103 | 0.662 | -0.383 | 0.127 |
|  |  |  | 3 | -0.046 | 0.065 | 1 | -0.205 | 0.112 | -0.063 | 0.083 | 1 | -0.274 | 0.149 | -0.039 | 0.089 | 1 | -0.26 | 0.182 |
|  |  | 3 | 1 | -0.054 | 0.083 | 1 | -0.258 | 0.15 | 0.025 | 0.102 | 1 | -0.236 | 0.286 | -0.089 | 0.113 | 1 | -0.369 | 0.191 |
|  |  |  | 2 | 0.046 | 0.065 | 1 | -0.112 | 0.205 | 0.063 | 0.083 | 1 | -0.149 | 0.274 | 0.039 | 0.089 | 1 | -0.182 | 0.26 |
|  | Placebo | 1 | 2 | 0.019 | 0.079 | 1 | -0.174 | 0.211 | 0.009 | 0.106 | 1 | -0.262 | 0.28 | 0.025 | 0.109 | 1 | -0.246 | 0.296 |
|  |  |  | 3 | -0.052 | 0.082 | 1 | -0.252 | 0.148 | -0.091 | 0.087 | 0.918 | -0.314 | 0.132 | -0.025 | 0.12 | 1 | -0.322 | 0.272 |
|  |  | 2 | 1 | -0.019 | 0.079 | 1 | -0.211 | 0.174 | -0.009 | 0.106 | 1 | -0.28 | 0.262 | -0.025 | 0.109 | 1 | -0.296 | 0.246 |
|  |  |  | 3 | -0.07 | 0.064 | 0.817 | -0.226 | 0.085 | -0.1 | 0.07 | 0.503 | -0.28 | 0.08 | -0.05 | 0.095 | 1 | -0.284 | 0.184 |
|  |  | 3 | 1 | 0.052 | 0.082 | 1 | -0.148 | 0.252 | 0.091 | 0.087 | 0.918 | -0.132 | 0.314 | 0.025 | 0.12 | 1 | -0.272 | 0.322 |
|  |  |  | 2 | 0.07 | 0.064 | 0.817 | -0.085 | 0.226 | 0.1 | 0.07 | 0.503 | -0.08 | 0.28 | 0.05 | 0.095 | 1 | -0.184 | 0.284 |
|  | 5mg | 1 | 2 | 0.13 | 0.075 | 0.259 | -0.053 | 0.313 | -0.04 | 0.111 | 1 | -0.324 | 0.244 | 0.215 | 0.098 | 0.097 | -0.027 | 0.457 |
|  |  |  | 3 | 0.133 | 0.078 | 0.27 | -0.057 | 0.323 | -0.04 | 0.091 | 1 | -0.274 | 0.194 | 0.22 | 0.107 | 0.137 | -0.046 | 0.486 |
|  |  | 2 | 1 | -0.13 | 0.075 | 0.259 | -0.313 | 0.053 | 0.04 | 0.111 | 1 | -0.244 | 0.324 | -0.215 | 0.098 | 0.097 | -0.457 | 0.027 |
|  |  |  | 3 | 0.003 | 0.06 | 1 | -0.144 | 0.151 | 2.67E-15 | 0.074 | 1 | -0.189 | 0.189 | 0.005 | 0.085 | 1 | -0.205 | 0.215 |
|  |  | 3 | 1 | -0.133 | 0.078 | 0.27 | -0.323 | 0.057 | 0.04 | 0.091 | 1 | -0.194 | 0.274 | -0.22 | 0.107 | 0.137 | -0.486 | 0.046 |
|  |  |  | 2 | -0.003 | 0.06 | 1 | -0.151 | 0.144 | -2.67E-15 | 0.074 | 1 | -0.189 | 0.189 | -0.005 | 0.085 | 1 | -0.215 | 0.205 |
| Chloride | 10mg | 1 | 2 | -1.077* | 0.389 | 0.021 | -2.027 | -0.127 | -1.625 | 0.844 | 0.196 | -3.786 | 0.536 | -0.833 | 0.391 | 0.114 | -1.802 | 0.135 |
|  |  |  | 3 | -0.692 | 0.466 | 0.424 | -1.831 | 0.447 | -0.75 | 1.072 | 1 | -3.494 | 1.994 | -0.667 | 0.469 | 0.484 | -1.828 | 0.494 |
|  |  | 2 | 1 | 1.077* | 0.389 | 0.021 | 0.127 | 2.027 | 1.625 | 0.844 | 0.196 | -0.536 | 3.786 | 0.833 | 0.391 | 0.114 | -0.135 | 1.802 |
|  |  |  | 3 | 0.385 | 0.453 | 1 | -0.723 | 1.492 | 0.875 | 0.877 | 0.983 | -1.37 | 3.12 | 0.167 | 0.523 | 1 | -1.127 | 1.46 |
|  |  | 3 | 1 | 0.692 | 0.466 | 0.424 | -0.447 | 1.831 | 0.75 | 1.072 | 1 | -1.994 | 3.494 | 0.667 | 0.469 | 0.484 | -0.494 | 1.828 |
|  |  |  | 2 | -0.385 | 0.453 | 1 | -1.492 | 0.723 | -0.875 | 0.877 | 0.983 | -3.12 | 1.37 | -0.167 | 0.523 | 1 | -1.46 | 1.127 |
|  | Placebo | 1 | 2 | -0.037 | 0.381 | 1 | -0.97 | 0.896 | 1 | 0.72 | 0.53 | -0.843 | 2.843 | -0.75 | 0.415 | 0.23 | -1.777 | 0.277 |
|  |  |  | 3 | 0.259 | 0.457 | 1 | -0.859 | 1.377 | 0.545 | 0.914 | 1 | -1.794 | 2.885 | 0.063 | 0.497 | 1 | -1.169 | 1.294 |
|  |  | 2 | 1 | 0.037 | 0.381 | 1 | -0.896 | 0.97 | -1 | 0.72 | 0.53 | -2.843 | 0.843 | 0.75 | 0.415 | 0.23 | -0.277 | 1.777 |
|  |  |  | 3 | 0.296 | 0.444 | 1 | -0.79 | 1.383 | -0.455 | 0.748 | 1 | -2.369 | 1.46 | 0.813 | 0.554 | 0.447 | -0.56 | 2.185 |
|  |  | 3 | 1 | -0.259 | 0.457 | 1 | -1.377 | 0.859 | -0.545 | 0.914 | 1 | -2.885 | 1.794 | -0.063 | 0.497 | 1 | -1.294 | 1.169 |
|  |  |  | 2 | -0.296 | 0.444 | 1 | -1.383 | 0.79 | 0.455 | 0.748 | 1 | -1.46 | 2.369 | -0.813 | 0.554 | 0.447 | -2.185 | 0.56 |
|  | 5mg | 1 | 2 | 0.233 | 0.362 | 1 | -0.651 | 1.118 | 0.6 | 0.755 | 1 | -1.332 | 2.532 | 0.05 | 0.371 | 1 | -0.869 | 0.969 |
|  |  |  | 3 | 0.333 | 0.434 | 1 | -0.727 | 1.394 | 1.1 | 0.959 | 0.785 | -1.354 | 3.554 | -0.05 | 0.445 | 1 | -1.151 | 1.051 |
|  |  | 2 | 1 | -0.233 | 0.362 | 1 | -1.118 | 0.651 | -0.6 | 0.755 | 1 | -2.532 | 1.332 | -0.05 | 0.371 | 1 | -0.969 | 0.869 |
|  |  |  | 3 | 0.1 | 0.422 | 1 | -0.931 | 1.131 | 0.5 | 0.785 | 1 | -1.508 | 2.508 | -0.1 | 0.496 | 1 | -1.327 | 1.127 |
|  |  | 3 | 1 | -0.333 | 0.434 | 1 | -1.394 | 0.727 | -1.1 | 0.959 | 0.785 | -3.554 | 1.354 | 0.05 | 0.445 | 1 | -1.051 | 1.151 |
|  |  |  | 2 | -0.1 | 0.422 | 1 | -1.131 | 0.931 | -0.5 | 0.785 | 1 | -2.508 | 1.508 | 0.1 | 0.496 | 1 | -1.127 | 1.327 |
| Carbon Dioxide | 10mg | 1 | 2 | 1.192* | 0.439 | 0.024 | 0.12 | 2.265 | 0.375 | 0.681 | 1 | -1.367 | 2.117 | 1.556* | 0.535 | 0.016 | 0.232 | 2.879 |
|  |  |  | 3 | 1.308* | 0.406 | 0.006 | 0.315 | 2.301 | 1.375 | 0.568 | 0.069 | -0.079 | 2.829 | 1.278 | 0.544 | 0.068 | -0.069 | 2.625 |
|  |  | 2 | 1 | -1.192* | 0.439 | 0.024 | -2.265 | -0.12 | -0.375 | 0.681 | 1 | -2.117 | 1.367 | -1.556* | 0.535 | 0.016 | -2.879 | -0.232 |
|  |  |  | 3 | 0.115 | 0.463 | 1 | -1.016 | 1.247 | 1 | 0.801 | 0.67 | -1.051 | 3.051 | -0.278 | 0.538 | 1 | -1.609 | 1.054 |
|  |  | 3 | 1 | -1.308* | 0.406 | 0.006 | -2.301 | -0.315 | -1.375 | 0.568 | 0.069 | -2.829 | 0.079 | -1.278 | 0.544 | 0.068 | -2.625 | 0.069 |
|  |  |  | 2 | -0.115 | 0.463 | 1 | -1.247 | 1.016 | -1 | 0.801 | 0.67 | -3.051 | 1.051 | 0.278 | 0.538 | 1 | -1.054 | 1.609 |
|  | Placebo | 1 | 2 | 0.074 | 0.43 | 1 | -0.978 | 1.127 | 0.091 | 0.581 | 1 | -1.395 | 1.576 | 0.063 | 0.567 | 1 | -1.341 | 1.466 |
|  |  |  | 3 | 0.63 | 0.399 | 0.354 | -0.345 | 1.604 | 1.091 | 0.485 | 0.099 | -0.149 | 2.331 | 0.313 | 0.577 | 1 | -1.116 | 1.741 |
|  |  | 2 | 1 | -0.074 | 0.43 | 1 | -1.127 | 0.978 | -0.091 | 0.581 | 1 | -1.576 | 1.395 | -0.063 | 0.567 | 1 | -1.466 | 1.341 |
|  |  |  | 3 | 0.556 | 0.454 | 0.675 | -0.555 | 1.666 | 1 | 0.683 | 0.466 | -0.749 | 2.749 | 0.25 | 0.57 | 1 | -1.162 | 1.662 |
|  |  | 3 | 1 | -0.63 | 0.399 | 0.354 | -1.604 | 0.345 | -1.091 | 0.485 | 0.099 | -2.331 | 0.149 | -0.313 | 0.577 | 1 | -1.741 | 1.116 |
|  |  |  | 2 | -0.556 | 0.454 | 0.675 | -1.666 | 0.555 | -1 | 0.683 | 0.466 | -2.749 | 0.749 | -0.25 | 0.57 | 1 | -1.662 | 1.162 |
|  | 5mg | 1 | 2 | 1.300* | 0.408 | 0.006 | 0.301 | 2.299 | 2.800* | 0.609 | <.001 | 1.242 | 4.358 | 0.55 | 0.507 | 0.85 | -0.705 | 1.805 |
|  |  |  | 3 | 0.6 | 0.378 | 0.349 | -0.324 | 1.524 | 0.6 | 0.508 | 0.746 | -0.701 | 1.901 | 0.6 | 0.516 | 0.752 | -0.678 | 1.878 |
|  |  | 2 | 1 | -1.300* | 0.408 | 0.006 | -2.299 | -0.301 | -2.800* | 0.609 | <.001 | -4.358 | -1.242 | -0.55 | 0.507 | 0.85 | -1.805 | 0.705 |
|  |  |  | 3 | -0.7 | 0.431 | 0.325 | -1.754 | 0.354 | -2.200* | 0.717 | 0.015 | -4.034 | -0.366 | 0.05 | 0.51 | 1 | -1.213 | 1.313 |
|  |  | 3 | 1 | -0.6 | 0.378 | 0.349 | -1.524 | 0.324 | -0.6 | 0.508 | 0.746 | -1.901 | 0.701 | -0.6 | 0.516 | 0.752 | -1.878 | 0.678 |
|  |  |  | 2 | 0.7 | 0.431 | 0.325 | -0.354 | 1.754 | 2.200* | 0.717 | 0.015 | 0.366 | 4.034 | -0.05 | 0.51 | 1 | -1.313 | 1.213 |
| Calcium | 10mg | 1 | 2 | .158* | 0.061 | 0.034 | 0.009 | 0.306 | .263* | 0.1 | 0.042 | 0.008 | 0.517 | 0.111 | 0.076 | 0.449 | -0.077 | 0.299 |
|  |  |  | 3 | .238* | 0.069 | 0.003 | 0.07 | 0.406 | .363* | 0.12 | 0.017 | 0.055 | 0.67 | 0.183 | 0.084 | 0.1 | -0.024 | 0.391 |
|  |  | 2 | 1 | -.158* | 0.061 | 0.034 | -0.306 | -0.009 | -.263* | 0.1 | 0.042 | -0.517 | -0.008 | -0.111 | 0.076 | 0.449 | -0.299 | 0.077 |
|  |  |  | 3 | 0.081 | 0.066 | 0.665 | -0.08 | 0.241 | 0.1 | 0.111 | 1 | -0.184 | 0.384 | 0.072 | 0.082 | 1 | -0.13 | 0.275 |
|  |  | 3 | 1 | -.238* | 0.069 | 0.003 | -0.406 | -0.07 | -.363* | 0.12 | 0.017 | -0.67 | -0.055 | -0.183 | 0.084 | 0.1 | -0.391 | 0.024 |
|  |  |  | 2 | -0.081 | 0.066 | 0.665 | -0.241 | 0.08 | -0.1 | 0.111 | 1 | -0.384 | 0.184 | -0.072 | 0.082 | 1 | -0.275 | 0.13 |
|  | Placebo | 1 | 2 | .200* | 0.06 | 0.004 | 0.054 | 0.346 | 0.164 | 0.085 | 0.195 | -0.054 | 0.381 | .225* | 0.081 | 0.022 | 0.026 | 0.424 |
|  |  |  | 3 | 0.107 | 0.067 | 0.345 | -0.057 | 0.272 | 0.182 | 0.102 | 0.262 | -0.08 | 0.444 | 0.056 | 0.089 | 1 | -0.164 | 0.276 |
|  |  | 2 | 1 | -.200* | 0.06 | 0.004 | -0.346 | -0.054 | -0.164 | 0.085 | 0.195 | -0.381 | 0.054 | -.225* | 0.081 | 0.022 | -0.424 | -0.026 |
|  |  |  | 3 | -0.093 | 0.064 | 0.462 | -0.25 | 0.065 | 0.018 | 0.095 | 1 | -0.224 | 0.26 | -0.169 | 0.087 | 0.172 | -0.383 | 0.046 |
|  |  | 3 | 1 | -0.107 | 0.067 | 0.345 | -0.272 | 0.057 | -0.182 | 0.102 | 0.262 | -0.444 | 0.08 | -0.056 | 0.089 | 1 | -0.276 | 0.164 |
|  |  |  | 2 | 0.093 | 0.064 | 0.462 | -0.065 | 0.25 | -0.018 | 0.095 | 1 | -0.26 | 0.224 | 0.169 | 0.087 | 0.172 | -0.046 | 0.383 |
|  | 5mg | 1 | 2 | 0.01 | 0.057 | 1 | -0.128 | 0.148 | -0.1 | 0.089 | 0.816 | -0.328 | 0.128 | 0.065 | 0.072 | 1 | -0.113 | 0.243 |
|  |  |  | 3 | 0.077 | 0.064 | 0.702 | -0.08 | 0.233 | 5.33E-15 | 0.107 | 1 | -0.275 | 0.275 | 0.115 | 0.08 | 0.463 | -0.082 | 0.312 |
|  |  | 2 | 1 | -0.01 | 0.057 | 1 | -0.148 | 0.128 | 0.1 | 0.089 | 0.816 | -0.128 | 0.328 | -0.065 | 0.072 | 1 | -0.243 | 0.113 |
|  |  |  | 3 | 0.067 | 0.061 | 0.834 | -0.083 | 0.216 | 0.1 | 0.099 | 0.968 | -0.154 | 0.354 | 0.05 | 0.078 | 1 | -0.142 | 0.242 |
|  |  | 3 | 1 | -0.077 | 0.064 | 0.702 | -0.233 | 0.08 | -5.33E-15 | 0.107 | 1 | -0.275 | 0.275 | -0.115 | 0.08 | 0.463 | -0.312 | 0.082 |
|  |  |  | 2 | -0.067 | 0.061 | 0.834 | -0.216 | 0.083 | -0.1 | 0.099 | 0.968 | -0.354 | 0.154 | -0.05 | 0.078 | 1 | -0.242 | 0.142 |
| Total Protein | 10mg | 1 | 2 | 0.082 | 0.057 | 0.456 | -0.057 | 0.221 | 0.075 | 0.109 | 1 | -0.2 | 0.35 | 0.085 | 0.068 | 0.649 | -0.083 | 0.253 |
|  |  |  | 3 | 0.104 | 0.058 | 0.234 | -0.038 | 0.245 | 0.037 | 0.103 | 1 | -0.221 | 0.296 | 0.13 | 0.072 | 0.226 | -0.047 | 0.307 |
|  |  | 2 | 1 | -0.082 | 0.057 | 0.456 | -0.221 | 0.057 | -0.075 | 0.109 | 1 | -0.35 | 0.2 | -0.085 | 0.068 | 0.649 | -0.253 | 0.083 |
|  |  |  | 3 | 0.021 | 0.064 | 1 | -0.135 | 0.178 | -0.038 | 0.124 | 1 | -0.35 | 0.275 | 0.045 | 0.076 | 1 | -0.142 | 0.232 |
|  |  | 3 | 1 | -0.104 | 0.058 | 0.234 | -0.245 | 0.038 | -0.037 | 0.103 | 1 | -0.296 | 0.221 | -0.13 | 0.072 | 0.226 | -0.307 | 0.047 |
|  |  |  | 2 | -0.021 | 0.064 | 1 | -0.178 | 0.135 | 0.038 | 0.124 | 1 | -0.275 | 0.35 | -0.045 | 0.076 | 1 | -0.232 | 0.142 |
|  | Placebo | 1 | 2 | .133* | 0.052 | 0.038 | 0.006 | 0.261 | 0.143 | 0.083 | 0.277 | -0.065 | 0.35 | 0.126 | 0.07 | 0.226 | -0.046 | 0.298 |
|  |  |  | 3 | -0.015 | 0.054 | 1 | -0.146 | 0.115 | -0.05 | 0.078 | 1 | -0.245 | 0.145 | 0.011 | 0.074 | 1 | -0.171 | 0.192 |
|  |  | 2 | 1 | -.133* | 0.052 | 0.038 | -0.261 | -0.006 | -0.143 | 0.083 | 0.277 | -0.35 | 0.065 | -0.126 | 0.07 | 0.226 | -0.298 | 0.046 |
|  |  |  | 3 | -.148* | 0.059 | 0.042 | -0.293 | -0.004 | -0.193 | 0.094 | 0.142 | -0.429 | 0.043 | -0.116 | 0.078 | 0.425 | -0.307 | 0.076 |
|  |  | 3 | 1 | 0.015 | 0.054 | 1 | -0.115 | 0.146 | 0.05 | 0.078 | 1 | -0.145 | 0.245 | -0.011 | 0.074 | 1 | -0.192 | 0.171 |
|  |  |  | 2 | .148* | 0.059 | 0.042 | 0.004 | 0.293 | 0.193 | 0.094 | 0.142 | -0.043 | 0.429 | 0.116 | 0.078 | 0.425 | -0.076 | 0.307 |
|  | 5mg | 1 | 2 | 0.005 | 0.05 | 1 | -0.115 | 0.126 | 0.006 | 0.077 | 1 | -0.188 | 0.2 | 0.005 | 0.066 | 1 | -0.159 | 0.168 |
|  |  |  | 3 | -0.046 | 0.051 | 1 | -0.169 | 0.077 | -0.1 | 0.073 | 0.532 | -0.283 | 0.083 | -0.005 | 0.07 | 1 | -0.177 | 0.168 |
|  |  | 2 | 1 | -0.005 | 0.05 | 1 | -0.126 | 0.115 | -0.006 | 0.077 | 1 | -0.2 | 0.188 | -0.005 | 0.066 | 1 | -0.168 | 0.159 |
|  |  |  | 3 | -0.051 | 0.056 | 1 | -0.188 | 0.085 | -0.106 | 0.088 | 0.703 | -0.327 | 0.115 | -0.01 | 0.074 | 1 | -0.192 | 0.173 |
|  |  | 3 | 1 | 0.046 | 0.051 | 1 | -0.077 | 0.169 | 0.1 | 0.073 | 0.532 | -0.083 | 0.283 | 0.005 | 0.07 | 1 | -0.168 | 0.177 |
|  |  |  | 2 | 0.051 | 0.056 | 1 | -0.085 | 0.188 | 0.106 | 0.088 | 0.703 | -0.115 | 0.327 | 0.01 | 0.074 | 1 | -0.173 | 0.192 |
| Albumin | 10mg | 1 | 2 | 0.079 | 0.041 | 0.174 | -0.021 | 0.178 | 0.137 | 0.076 | 0.242 | -0.055 | 0.33 | 0.055 | 0.049 | 0.809 | -0.067 | 0.177 |
|  |  |  | 3 | 0.089 | 0.043 | 0.119 | -0.015 | 0.194 | 0.075 | 0.085 | 1 | -0.139 | 0.289 | 0.095 | 0.049 | 0.173 | -0.026 | 0.216 |
|  |  | 2 | 1 | -0.079 | 0.041 | 0.174 | -0.178 | 0.021 | -0.137 | 0.076 | 0.242 | -0.33 | 0.055 | -0.055 | 0.049 | 0.809 | -0.177 | 0.067 |
|  |  |  | 3 | 0.011 | 0.048 | 1 | -0.105 | 0.127 | -0.062 | 0.087 | 1 | -0.282 | 0.157 | 0.04 | 0.057 | 1 | -0.101 | 0.181 |
|  |  | 3 | 1 | -0.089 | 0.043 | 0.119 | -0.194 | 0.015 | -0.075 | 0.085 | 1 | -0.289 | 0.139 | -0.095 | 0.049 | 0.173 | -0.216 | 0.026 |
|  |  |  | 2 | -0.011 | 0.048 | 1 | -0.127 | 0.105 | 0.062 | 0.087 | 1 | -0.157 | 0.282 | -0.04 | 0.057 | 1 | -0.181 | 0.101 |
|  | Placebo | 1 | 2 | 0.076 | 0.038 | 0.143 | -0.016 | 0.168 | 0.1 | 0.058 | 0.277 | -0.045 | 0.245 | 0.058 | 0.051 | 0.772 | -0.067 | 0.183 |
|  |  |  | 3 | -0.015 | 0.039 | 1 | -0.111 | 0.081 | -0.007 | 0.064 | 1 | -0.169 | 0.155 | -0.021 | 0.05 | 1 | -0.145 | 0.103 |
|  |  | 2 | 1 | -0.076 | 0.038 | 0.143 | -0.168 | 0.016 | -0.1 | 0.058 | 0.277 | -0.245 | 0.045 | -0.058 | 0.051 | 0.772 | -0.183 | 0.067 |
|  |  |  | 3 | -0.091 | 0.044 | 0.123 | -0.198 | 0.016 | -0.107 | 0.066 | 0.338 | -0.273 | 0.058 | -0.079 | 0.059 | 0.555 | -0.224 | 0.066 |
|  |  | 3 | 1 | 0.015 | 0.039 | 1 | -0.081 | 0.111 | 0.007 | 0.064 | 1 | -0.155 | 0.169 | 0.021 | 0.05 | 1 | -0.103 | 0.145 |
|  |  |  | 2 | 0.091 | 0.044 | 0.123 | -0.016 | 0.198 | 0.107 | 0.066 | 0.338 | -0.058 | 0.273 | 0.079 | 0.059 | 0.555 | -0.066 | 0.224 |
|  | 5mg | 1 | 2 | -0.011 | 0.036 | 1 | -0.098 | 0.076 | -0.012 | 0.054 | 1 | -0.148 | 0.123 | -0.01 | 0.048 | 1 | -0.128 | 0.109 |
|  |  |  | 3 | -0.016 | 0.037 | 1 | -0.107 | 0.075 | -0.075 | 0.06 | 0.665 | -0.227 | 0.077 | 0.029 | 0.048 | 1 | -0.09 | 0.147 |
|  |  | 2 | 1 | 0.011 | 0.036 | 1 | -0.076 | 0.098 | 0.012 | 0.054 | 1 | -0.123 | 0.148 | 0.01 | 0.048 | 1 | -0.109 | 0.128 |
|  |  |  | 3 | -0.005 | 0.041 | 1 | -0.106 | 0.096 | -0.063 | 0.062 | 0.952 | -0.217 | 0.092 | 0.038 | 0.056 | 1 | -0.1 | 0.176 |
|  |  | 3 | 1 | 0.016 | 0.037 | 1 | -0.075 | 0.107 | 0.075 | 0.06 | 0.665 | -0.077 | 0.227 | -0.029 | 0.048 | 1 | -0.147 | 0.09 |
|  |  |  | 2 | 0.005 | 0.041 | 1 | -0.096 | 0.106 | 0.063 | 0.062 | 0.952 | -0.092 | 0.217 | -0.038 | 0.056 | 1 | -0.176 | 0.1 |
| Globulin | 10mg | 1 | 2 | 0.004 | 0.042 | 1 | -0.099 | 0.106 | -0.063 | 0.069 | 1 | -0.236 | 0.111 | 0.03 | 0.054 | 1 | -0.103 | 0.163 |
|  |  |  | 3 | 0.014 | 0.043 | 1 | -0.09 | 0.118 | -0.038 | 0.047 | 1 | -0.156 | 0.081 | 0.035 | 0.06 | 1 | -0.113 | 0.183 |
|  |  | 2 | 1 | -0.004 | 0.042 | 1 | -0.106 | 0.099 | 0.063 | 0.069 | 1 | -0.111 | 0.236 | -0.03 | 0.054 | 1 | -0.163 | 0.103 |
|  |  |  | 3 | 0.011 | 0.042 | 1 | -0.091 | 0.112 | 0.025 | 0.079 | 1 | -0.173 | 0.223 | 0.005 | 0.05 | 1 | -0.119 | 0.129 |
|  |  | 3 | 1 | -0.014 | 0.043 | 1 | -0.118 | 0.09 | 0.038 | 0.047 | 1 | -0.081 | 0.156 | -0.035 | 0.06 | 1 | -0.183 | 0.113 |
|  |  |  | 2 | -0.011 | 0.042 | 1 | -0.112 | 0.091 | -0.025 | 0.079 | 1 | -0.223 | 0.173 | -0.005 | 0.05 | 1 | -0.129 | 0.119 |
|  | Placebo | 1 | 2 | 0.058 | 0.039 | 0.421 | -0.037 | 0.152 | 0.043 | 0.052 | 1 | -0.089 | 0.174 | 0.068 | 0.055 | 0.66 | -0.068 | 0.205 |
|  |  |  | 3 | 0 | 0.039 | 1 | -0.096 | 0.096 | -0.043 | 0.036 | 0.712 | -0.133 | 0.047 | 0.032 | 0.062 | 1 | -0.121 | 0.184 |
|  |  | 2 | 1 | -0.058 | 0.039 | 0.421 | -0.152 | 0.037 | -0.043 | 0.052 | 1 | -0.174 | 0.089 | -0.068 | 0.055 | 0.66 | -0.205 | 0.068 |
|  |  |  | 3 | -0.058 | 0.038 | 0.413 | -0.151 | 0.036 | -0.086 | 0.059 | 0.475 | -0.235 | 0.064 | -0.037 | 0.052 | 1 | -0.164 | 0.09 |
|  |  | 3 | 1 | 0 | 0.039 | 1 | -0.096 | 0.096 | 0.043 | 0.036 | 0.712 | -0.047 | 0.133 | -0.032 | 0.062 | 1 | -0.184 | 0.121 |
|  |  |  | 2 | 0.058 | 0.038 | 0.413 | -0.036 | 0.151 | 0.086 | 0.059 | 0.475 | -0.064 | 0.235 | 0.037 | 0.052 | 1 | -0.09 | 0.164 |
|  | 5mg | 1 | 2 | -0.001 | 0.037 | 1 | -0.09 | 0.088 | 0.019 | 0.049 | 1 | -0.104 | 0.142 | -0.016 | 0.052 | 1 | -0.145 | 0.114 |
|  |  |  | 3 | -0.044 | 0.037 | 0.714 | -0.134 | 0.046 | -0.025 | 0.033 | 1 | -0.109 | 0.059 | -0.059 | 0.059 | 0.969 | -0.203 | 0.086 |
|  |  | 2 | 1 | 0.001 | 0.037 | 1 | -0.088 | 0.09 | -0.019 | 0.049 | 1 | -0.142 | 0.104 | 0.016 | 0.052 | 1 | -0.114 | 0.145 |
|  |  |  | 3 | -0.043 | 0.036 | 0.711 | -0.132 | 0.045 | -0.044 | 0.056 | 1 | -0.184 | 0.096 | -0.043 | 0.049 | 1 | -0.164 | 0.078 |
|  |  | 3 | 1 | 0.044 | 0.037 | 0.714 | -0.046 | 0.134 | 0.025 | 0.033 | 1 | -0.059 | 0.109 | 0.059 | 0.059 | 0.969 | -0.086 | 0.203 |
|  |  |  | 2 | 0.043 | 0.036 | 0.711 | -0.045 | 0.132 | 0.044 | 0.056 | 1 | -0.096 | 0.184 | 0.043 | 0.049 | 1 | -0.078 | 0.164 |
| Albumin Globulin Ratio | 10mg | 1 | 2 | 0.029 | 0.037 | 1 | -0.061 | 0.118 | 0.112 | 0.063 | 0.249 | -0.046 | 0.271 | -0.005 | 0.046 | 1 | -0.118 | 0.108 |
|  |  |  | 3 | 0.014 | 0.041 | 1 | -0.086 | 0.115 | 0.075 | 0.058 | 0.62 | -0.072 | 0.222 | -0.01 | 0.055 | 1 | -0.146 | 0.126 |
|  |  | 2 | 1 | -0.029 | 0.037 | 1 | -0.118 | 0.061 | -0.112 | 0.063 | 0.249 | -0.271 | 0.046 | 0.005 | 0.046 | 1 | -0.108 | 0.118 |
|  |  |  | 3 | -0.014 | 0.04 | 1 | -0.111 | 0.083 | -0.038 | 0.071 | 1 | -0.216 | 0.141 | -0.005 | 0.049 | 1 | -0.127 | 0.117 |
|  |  | 3 | 1 | -0.014 | 0.041 | 1 | -0.115 | 0.086 | -0.075 | 0.058 | 0.62 | -0.222 | 0.072 | 0.01 | 0.055 | 1 | -0.126 | 0.146 |
|  |  |  | 2 | 0.014 | 0.04 | 1 | -0.083 | 0.111 | 0.038 | 0.071 | 1 | -0.141 | 0.216 | 0.005 | 0.049 | 1 | -0.117 | 0.127 |
|  | Placebo | 1 | 2 | -0.033 | 0.034 | 0.985 | -0.116 | 0.049 | 0.007 | 0.048 | 1 | -0.113 | 0.127 | -0.063 | 0.047 | 0.547 | -0.179 | 0.052 |
|  |  |  | 3 | 0.006 | 0.038 | 1 | -0.087 | 0.099 | 0.036 | 0.044 | 1 | -0.075 | 0.147 | -0.016 | 0.057 | 1 | -0.156 | 0.124 |
|  |  | 2 | 1 | 0.033 | 0.034 | 0.985 | -0.049 | 0.116 | -0.007 | 0.048 | 1 | -0.127 | 0.113 | 0.063 | 0.047 | 0.547 | -0.052 | 0.179 |
|  |  |  | 3 | 0.039 | 0.037 | 0.854 | -0.05 | 0.129 | 0.029 | 0.054 | 1 | -0.106 | 0.163 | 0.047 | 0.051 | 1 | -0.077 | 0.172 |
|  |  | 3 | 1 | -0.006 | 0.038 | 1 | -0.099 | 0.087 | -0.036 | 0.044 | 1 | -0.147 | 0.075 | 0.016 | 0.057 | 1 | -0.124 | 0.156 |
|  |  |  | 2 | -0.039 | 0.037 | 0.854 | -0.129 | 0.05 | -0.029 | 0.054 | 1 | -0.163 | 0.106 | -0.047 | 0.051 | 1 | -0.172 | 0.077 |
|  | 5mg | 1 | 2 | -0.019 | 0.032 | 1 | -0.097 | 0.059 | -0.031 | 0.045 | 1 | -0.143 | 0.081 | -0.01 | 0.045 | 1 | -0.119 | 0.1 |
|  |  |  | 3 | 0.019 | 0.036 | 1 | -0.069 | 0.106 | -0.019 | 0.041 | 1 | -0.122 | 0.085 | 0.048 | 0.054 | 1 | -0.085 | 0.181 |
|  |  | 2 | 1 | 0.019 | 0.032 | 1 | -0.059 | 0.097 | 0.031 | 0.045 | 1 | -0.081 | 0.143 | 0.01 | 0.045 | 1 | -0.1 | 0.119 |
|  |  |  | 3 | 0.038 | 0.035 | 0.829 | -0.046 | 0.122 | 0.013 | 0.05 | 1 | -0.113 | 0.138 | 0.057 | 0.048 | 0.719 | -0.061 | 0.176 |
|  |  | 3 | 1 | -0.019 | 0.036 | 1 | -0.106 | 0.069 | 0.019 | 0.041 | 1 | -0.085 | 0.122 | -0.048 | 0.054 | 1 | -0.181 | 0.085 |
|  |  |  | 2 | -0.038 | 0.035 | 0.829 | -0.122 | 0.046 | -0.013 | 0.05 | 1 | -0.138 | 0.113 | -0.057 | 0.048 | 0.719 | -0.176 | 0.061 |
| Total Bilirubin | 10mg | 1 | 2 | -0.039 | 0.043 | 1 | -0.144 | 0.065 | -0.063 | 0.058 | 0.868 | -0.209 | 0.084 | -0.03 | 0.058 | 1 | -0.174 | 0.114 |
|  |  |  | 3 | -0.021 | 0.042 | 1 | -0.125 | 0.082 | -2.22E-16 | 0.053 | 1 | -0.134 | 0.134 | -0.03 | 0.059 | 1 | -0.176 | 0.116 |
|  |  | 2 | 1 | 0.039 | 0.043 | 1 | -0.065 | 0.144 | 0.063 | 0.058 | 0.868 | -0.084 | 0.209 | 0.03 | 0.058 | 1 | -0.114 | 0.174 |
|  |  |  | 3 | 0.018 | 0.042 | 1 | -0.083 | 0.119 | 0.063 | 0.062 | 0.959 | -0.093 | 0.218 | -1.11E-16 | 0.054 | 1 | -0.134 | 0.134 |
|  |  | 3 | 1 | 0.021 | 0.042 | 1 | -0.082 | 0.125 | 2.22E-16 | 0.053 | 1 | -0.134 | 0.134 | 0.03 | 0.059 | 1 | -0.116 | 0.176 |
|  |  |  | 2 | -0.018 | 0.042 | 1 | -0.119 | 0.083 | -0.063 | 0.062 | 0.959 | -0.218 | 0.093 | 1.11E-16 | 0.054 | 1 | -0.134 | 0.134 |
|  | Placebo | 1 | 2 | 0.006 | 0.039 | 1 | -0.09 | 0.102 | -0.014 | 0.044 | 1 | -0.125 | 0.096 | 0.021 | 0.06 | 1 | -0.127 | 0.169 |
|  |  |  | 3 | -0.033 | 0.039 | 1 | -0.129 | 0.062 | -1.11E-16 | 0.04 | 1 | -0.101 | 0.101 | -0.058 | 0.061 | 1 | -0.207 | 0.092 |
|  |  | 2 | 1 | -0.006 | 0.039 | 1 | -0.102 | 0.09 | 0.014 | 0.044 | 1 | -0.096 | 0.125 | -0.021 | 0.06 | 1 | -0.169 | 0.127 |
|  |  |  | 3 | -0.039 | 0.038 | 0.916 | -0.133 | 0.054 | 0.014 | 0.047 | 1 | -0.103 | 0.132 | -0.079 | 0.056 | 0.49 | -0.217 | 0.059 |
|  |  | 3 | 1 | 0.033 | 0.039 | 1 | -0.062 | 0.129 | 1.11E-16 | 0.04 | 1 | -0.101 | 0.101 | 0.058 | 0.061 | 1 | -0.092 | 0.207 |
|  |  |  | 2 | 0.039 | 0.038 | 0.916 | -0.054 | 0.133 | -0.014 | 0.047 | 1 | -0.132 | 0.103 | 0.079 | 0.056 | 0.49 | -0.059 | 0.217 |
|  | 5mg | 1 | 2 | 0.016 | 0.037 | 1 | -0.075 | 0.107 | -0.006 | 0.041 | 1 | -0.11 | 0.097 | 0.033 | 0.057 | 1 | -0.107 | 0.174 |
|  |  |  | 3 | -0.005 | 0.037 | 1 | -0.095 | 0.085 | 0.006 | 0.038 | 1 | -0.088 | 0.101 | -0.014 | 0.058 | 1 | -0.156 | 0.128 |
|  |  | 2 | 1 | -0.016 | 0.037 | 1 | -0.107 | 0.075 | 0.006 | 0.041 | 1 | -0.097 | 0.11 | -0.033 | 0.057 | 1 | -0.174 | 0.107 |
|  |  |  | 3 | -0.022 | 0.036 | 1 | -0.11 | 0.066 | 0.013 | 0.044 | 1 | -0.098 | 0.123 | -0.048 | 0.053 | 1 | -0.179 | 0.084 |
|  |  | 3 | 1 | 0.005 | 0.037 | 1 | -0.085 | 0.095 | -0.006 | 0.038 | 1 | -0.101 | 0.088 | 0.014 | 0.058 | 1 | -0.128 | 0.156 |
|  |  |  | 2 | 0.022 | 0.036 | 1 | -0.066 | 0.11 | -0.013 | 0.044 | 1 | -0.123 | 0.098 | 0.048 | 0.053 | 1 | -0.084 | 0.179 |
| Alkaline Phosphatase | 10mg | 1 | 2 | -0.893 | 2.223 | 1 | -6.31 | 4.525 | -7.625 | 5.365 | 0.492 | -21.115 | 5.865 | 1.8 | 1.947 | 1 | -3.003 | 6.603 |
|  |  |  | 3 | 0.321 | 2.41 | 1 | -5.552 | 6.195 | -7 | 4.782 | 0.457 | -19.025 | 5.025 | 3.25 | 2.603 | 0.651 | -3.172 | 9.672 |
|  |  | 2 | 1 | 0.893 | 2.223 | 1 | -4.525 | 6.31 | 7.625 | 5.365 | 0.492 | -5.865 | 21.115 | -1.8 | 1.947 | 1 | -6.603 | 3.003 |
|  |  |  | 3 | 1.214 | 3.038 | 1 | -6.191 | 8.619 | 0.625 | 7.583 | 1 | -18.443 | 19.693 | 1.45 | 2.417 | 1 | -4.512 | 7.412 |
|  |  | 3 | 1 | -0.321 | 2.41 | 1 | -6.195 | 5.552 | 7 | 4.782 | 0.457 | -5.025 | 19.025 | -3.25 | 2.603 | 0.651 | -9.672 | 3.172 |
|  |  |  | 2 | -1.214 | 3.038 | 1 | -8.619 | 6.191 | -0.625 | 7.583 | 1 | -19.693 | 18.443 | -1.45 | 2.417 | 1 | -7.412 | 4.512 |
|  | Placebo | 1 | 2 | -2.242 | 2.048 | 0.829 | -7.233 | 2.748 | -5.429 | 4.055 | 0.568 | -15.626 | 4.769 | 0.105 | 1.998 | 1 | -4.823 | 5.034 |
|  |  |  | 3 | -0.758 | 2.22 | 1 | -6.168 | 4.653 | 4.643 | 3.615 | 0.622 | -4.447 | 13.733 | -4.737 | 2.671 | 0.245 | -11.325 | 1.852 |
|  |  | 2 | 1 | 2.242 | 2.048 | 0.829 | -2.748 | 7.233 | 5.429 | 4.055 | 0.568 | -4.769 | 15.626 | -0.105 | 1.998 | 1 | -5.034 | 4.823 |
|  |  |  | 3 | 1.485 | 2.799 | 1 | -5.336 | 8.306 | 10.071 | 5.732 | 0.263 | -4.342 | 24.485 | -4.842 | 2.48 | 0.167 | -10.959 | 1.275 |
|  |  | 3 | 1 | 0.758 | 2.22 | 1 | -4.653 | 6.168 | -4.643 | 3.615 | 0.622 | -13.733 | 4.447 | 4.737 | 2.671 | 0.245 | -1.852 | 11.325 |
|  |  |  | 2 | -1.485 | 2.799 | 1 | -8.306 | 5.336 | -10.071 | 5.732 | 0.263 | -24.485 | 4.342 | 4.842 | 2.48 | 0.167 | -1.275 | 10.959 |
|  | 5mg | 1 | 2 | -0.595 | 1.934 | 1 | -5.307 | 4.118 | -0.062 | 3.793 | 1 | -9.601 | 9.476 | -1 | 1.9 | 1 | -5.688 | 3.688 |
|  |  |  | 3 | -1.757 | 2.096 | 1 | -6.866 | 3.353 | -1.187 | 3.382 | 1 | -9.691 | 7.316 | -2.19 | 2.541 | 1 | -8.458 | 4.077 |
|  |  | 2 | 1 | 0.595 | 1.934 | 1 | -4.118 | 5.307 | 0.062 | 3.793 | 1 | -9.476 | 9.601 | 1 | 1.9 | 1 | -3.688 | 5.688 |
|  |  |  | 3 | -1.162 | 2.643 | 1 | -7.604 | 5.28 | -1.125 | 5.362 | 1 | -14.608 | 12.358 | -1.19 | 2.359 | 1 | -7.009 | 4.628 |
|  |  | 3 | 1 | 1.757 | 2.096 | 1 | -3.353 | 6.866 | 1.187 | 3.382 | 1 | -7.316 | 9.691 | 2.19 | 2.541 | 1 | -4.077 | 8.458 |
|  |  |  | 2 | 1.162 | 2.643 | 1 | -5.28 | 7.604 | 1.125 | 5.362 | 1 | -12.358 | 14.608 | 1.19 | 2.359 | 1 | -4.628 | 7.009 |
| AST | 10mg | 1 | 2 | -0.75 | 1.346 | 1 | -4.031 | 2.531 | -2.75 | 1.893 | 0.466 | -7.51 | 2.01 | 0.05 | 1.804 | 1 | -4.399 | 4.499 |
|  |  |  | 3 | -0.607 | 1.043 | 1 | -3.148 | 1.934 | 1.25 | 1.316 | 1 | -2.059 | 4.559 | -1.35 | 1.401 | 1 | -4.807 | 2.107 |
|  |  | 2 | 1 | 0.75 | 1.346 | 1 | -2.531 | 4.031 | 2.75 | 1.893 | 0.466 | -2.01 | 7.51 | -0.05 | 1.804 | 1 | -4.499 | 4.399 |
|  |  |  | 3 | 0.143 | 1.428 | 1 | -3.337 | 3.623 | 4 | 1.855 | 0.114 | -0.665 | 8.665 | -1.4 | 1.916 | 1 | -6.126 | 3.326 |
|  |  | 3 | 1 | 0.607 | 1.043 | 1 | -1.934 | 3.148 | -1.25 | 1.316 | 1 | -4.559 | 2.059 | 1.35 | 1.401 | 1 | -2.107 | 4.807 |
|  |  |  | 2 | -0.143 | 1.428 | 1 | -3.623 | 3.337 | -4 | 1.855 | 0.114 | -8.665 | 0.665 | 1.4 | 1.916 | 1 | -3.326 | 6.126 |
|  | Placebo | 1 | 2 | -0.303 | 1.24 | 1 | -3.326 | 2.72 | 1.071 | 1.431 | 1 | -2.527 | 4.67 | -1.316 | 1.85 | 1 | -5.88 | 3.249 |
|  |  |  | 3 | 0.091 | 0.96 | 1 | -2.25 | 2.432 | -0.143 | 0.995 | 1 | -2.644 | 2.359 | 0.263 | 1.438 | 1 | -3.284 | 3.81 |
|  |  | 2 | 1 | 0.303 | 1.24 | 1 | -2.72 | 3.326 | -1.071 | 1.431 | 1 | -4.67 | 2.527 | 1.316 | 1.85 | 1 | -3.249 | 5.88 |
|  |  |  | 3 | 0.394 | 1.315 | 1 | -2.812 | 3.6 | -1.214 | 1.402 | 1 | -4.741 | 2.312 | 1.579 | 1.966 | 1 | -3.27 | 6.428 |
|  |  | 3 | 1 | -0.091 | 0.96 | 1 | -2.432 | 2.25 | 0.143 | 0.995 | 1 | -2.359 | 2.644 | -0.263 | 1.438 | 1 | -3.81 | 3.284 |
|  |  |  | 2 | -0.394 | 1.315 | 1 | -3.6 | 2.812 | 1.214 | 1.402 | 1 | -2.312 | 4.741 | -1.579 | 1.966 | 1 | -6.428 | 3.27 |
|  | 5mg | 1 | 2 | 1.784 | 1.171 | 0.393 | -1.071 | 4.638 | 0.938 | 1.339 | 1 | -2.428 | 4.303 | 2.429 | 1.76 | 0.519 | -1.913 | 6.77 |
|  |  |  | 3 | 1.757 | 0.907 | 0.167 | -0.454 | 3.967 | -0.375 | 0.931 | 1 | -2.715 | 1.965 | 3.381* | 1.368 | 0.049 | 0.007 | 6.755 |
|  |  | 2 | 1 | -1.784 | 1.171 | 0.393 | -4.638 | 1.071 | -0.938 | 1.339 | 1 | -4.303 | 2.428 | -2.429 | 1.76 | 0.519 | -6.77 | 1.913 |
|  |  |  | 3 | -0.027 | 1.242 | 1 | -3.055 | 3 | -1.313 | 1.312 | 0.972 | -4.611 | 1.986 | 0.952 | 1.87 | 1 | -3.66 | 5.564 |
|  |  | 3 | 1 | -1.757 | 0.907 | 0.167 | -3.967 | 0.454 | 0.375 | 0.931 | 1 | -1.965 | 2.715 | -3.381* | 1.368 | 0.049 | -6.755 | -0.007 |
|  |  |  | 2 | 0.027 | 1.242 | 1 | -3 | 3.055 | 1.313 | 1.312 | 0.972 | -1.986 | 4.611 | -0.952 | 1.87 | 1 | -5.564 | 3.66 |
| ALT | 10mg | 1 | 2 | -2.214 | 1.941 | 0.771 | -6.945 | 2.516 | -2.875 | 3.664 | 1 | -12.089 | 6.339 | -1.95 | 2.336 | 1 | -7.712 | 3.812 |
|  |  |  | 3 | -1.571 | 1.506 | 0.898 | -5.241 | 2.098 | 2 | 3.434 | 1 | -6.636 | 10.636 | -3 | 1.415 | 0.115 | -6.491 | 0.491 |
|  |  | 2 | 1 | 2.214 | 1.941 | 0.771 | -2.516 | 6.945 | 2.875 | 3.664 | 1 | -6.339 | 12.089 | 1.95 | 2.336 | 1 | -3.812 | 7.712 |
|  |  |  | 3 | 0.643 | 2.653 | 1 | -5.823 | 7.109 | 4.875 | 6.092 | 1 | -10.443 | 20.193 | -1.05 | 2.632 | 1 | -7.542 | 5.442 |
|  |  | 3 | 1 | 1.571 | 1.506 | 0.898 | -2.098 | 5.241 | -2 | 3.434 | 1 | -10.636 | 6.636 | 3 | 1.415 | 0.115 | -0.491 | 6.491 |
|  |  |  | 2 | -0.643 | 2.653 | 1 | -7.109 | 5.823 | -4.875 | 6.092 | 1 | -20.193 | 10.443 | 1.05 | 2.632 | 1 | -5.442 | 7.542 |
|  | Placebo | 1 | 2 | -1.97 | 1.788 | 0.82 | -6.327 | 2.388 | -1.286 | 2.77 | 1 | -8.251 | 5.68 | -2.474 | 2.397 | 0.919 | -8.385 | 3.438 |
|  |  |  | 3 | 1.212 | 1.387 | 1 | -2.168 | 4.592 | 4.714 | 2.596 | 0.234 | -1.814 | 11.242 | -1.368 | 1.452 | 1 | -4.95 | 2.213 |
|  |  | 2 | 1 | 1.97 | 1.788 | 0.82 | -2.388 | 6.327 | 1.286 | 2.77 | 1 | -5.68 | 8.251 | 2.474 | 2.397 | 0.919 | -3.438 | 8.385 |
|  |  |  | 3 | 3.182 | 2.444 | 0.588 | -2.774 | 9.138 | 6 | 4.605 | 0.603 | -5.579 | 17.579 | 1.105 | 2.7 | 1 | -5.555 | 7.766 |
|  |  | 3 | 1 | -1.212 | 1.387 | 1 | -4.592 | 2.168 | -4.714 | 2.596 | 0.234 | -11.242 | 1.814 | 1.368 | 1.452 | 1 | -2.213 | 4.95 |
|  |  |  | 2 | -3.182 | 2.444 | 0.588 | -9.138 | 2.774 | -6 | 4.605 | 0.603 | -17.579 | 5.579 | -1.105 | 2.7 | 1 | -7.766 | 5.555 |
|  | 5mg | 1 | 2 | 0.595 | 1.689 | 1 | -3.521 | 4.71 | 1.562 | 2.591 | 1 | -4.953 | 8.078 | -0.143 | 2.28 | 1 | -5.766 | 5.48 |
|  |  |  | 3 | 2.405 | 1.31 | 0.208 | -0.786 | 5.597 | 2.125 | 2.428 | 1 | -3.982 | 8.232 | 2.619 | 1.381 | 0.189 | -0.788 | 6.026 |
|  |  | 2 | 1 | -0.595 | 1.689 | 1 | -4.71 | 3.521 | -1.562 | 2.591 | 1 | -8.078 | 4.953 | 0.143 | 2.28 | 1 | -5.48 | 5.766 |
|  |  |  | 3 | 1.811 | 2.308 | 1 | -3.814 | 7.436 | 0.563 | 4.308 | 1 | -10.269 | 11.394 | 2.762 | 2.568 | 0.86 | -3.574 | 9.097 |
|  |  | 3 | 1 | -2.405 | 1.31 | 0.208 | -5.597 | 0.786 | -2.125 | 2.428 | 1 | -8.232 | 3.982 | -2.619 | 1.381 | 0.189 | -6.026 | 0.788 |
|  |  |  | 2 | -1.811 | 2.308 | 1 | -7.436 | 3.814 | -0.563 | 4.308 | 1 | -11.394 | 10.269 | -2.762 | 2.568 | 0.86 | -9.097 | 3.574 |
| Uric Acid | 10mg | 1 | 2 | 0.107 | 0.148 | 1 | -0.255 | 0.469 | 0.275 | 0.19 | 0.469 | -0.202 | 0.752 | 0.04 | 0.205 | 1 | -0.465 | 0.545 |
|  |  |  | 3 | 0.268 | 0.128 | 0.119 | -0.045 | 0.581 | 0.475 | 0.208 | 0.085 | -0.048 | 0.998 | 0.185 | 0.164 | 0.795 | -0.22 | 0.59 |
|  |  | 2 | 1 | -0.107 | 0.148 | 1 | -0.469 | 0.255 | -0.275 | 0.19 | 0.469 | -0.752 | 0.202 | -0.04 | 0.205 | 1 | -0.545 | 0.465 |
|  |  |  | 3 | 0.161 | 0.178 | 1 | -0.274 | 0.595 | 0.2 | 0.211 | 1 | -0.33 | 0.73 | 0.145 | 0.25 | 1 | -0.472 | 0.762 |
|  |  | 3 | 1 | -0.268 | 0.128 | 0.119 | -0.581 | 0.045 | -0.475 | 0.208 | 0.085 | -0.998 | 0.048 | -0.185 | 0.164 | 0.795 | -0.59 | 0.22 |
|  |  |  | 2 | -0.161 | 0.178 | 1 | -0.595 | 0.274 | -0.2 | 0.211 | 1 | -0.73 | 0.33 | -0.145 | 0.25 | 1 | -0.762 | 0.472 |
|  | Placebo | 1 | 2 | 0.152 | 0.137 | 0.812 | -0.182 | 0.485 | 0.071 | 0.143 | 1 | -0.289 | 0.432 | 0.211 | 0.21 | 0.962 | -0.308 | 0.729 |
|  |  |  | 3 | 0.121 | 0.118 | 0.925 | -0.167 | 0.41 | -0.014 | 0.157 | 1 | -0.409 | 0.381 | 0.221 | 0.169 | 0.585 | -0.195 | 0.637 |
|  |  | 2 | 1 | -0.152 | 0.137 | 0.812 | -0.485 | 0.182 | -0.071 | 0.143 | 1 | -0.432 | 0.289 | -0.211 | 0.21 | 0.962 | -0.729 | 0.308 |
|  |  |  | 3 | -0.03 | 0.164 | 1 | -0.43 | 0.37 | -0.086 | 0.159 | 1 | -0.486 | 0.315 | 0.011 | 0.257 | 1 | -0.622 | 0.643 |
|  |  | 3 | 1 | -0.121 | 0.118 | 0.925 | -0.41 | 0.167 | 0.014 | 0.157 | 1 | -0.381 | 0.409 | -0.221 | 0.169 | 0.585 | -0.637 | 0.195 |
|  |  |  | 2 | 0.03 | 0.164 | 1 | -0.37 | 0.43 | 0.086 | 0.159 | 1 | -0.315 | 0.486 | -0.011 | 0.257 | 1 | -0.643 | 0.622 |
|  | 5mg | 1 | 2 | 0.211 | 0.129 | 0.317 | -0.104 | 0.525 | 0.119 | 0.134 | 1 | -0.219 | 0.456 | 0.281 | 0.2 | 0.495 | -0.212 | 0.774 |
|  |  |  | 3 | 0.251 | 0.112 | 0.08 | -0.021 | 0.524 | 0.312 | 0.147 | 0.122 | -0.057 | 0.682 | 0.205 | 0.16 | 0.62 | -0.191 | 0.6 |
|  |  | 2 | 1 | -0.211 | 0.129 | 0.317 | -0.525 | 0.104 | -0.119 | 0.134 | 1 | -0.456 | 0.219 | -0.281 | 0.2 | 0.495 | -0.774 | 0.212 |
|  |  |  | 3 | 0.041 | 0.155 | 1 | -0.337 | 0.418 | 0.194 | 0.149 | 0.606 | -0.181 | 0.568 | -0.076 | 0.244 | 1 | -0.678 | 0.526 |
|  |  | 3 | 1 | -0.251 | 0.112 | 0.08 | -0.524 | 0.021 | -0.312 | 0.147 | 0.122 | -0.682 | 0.057 | -0.205 | 0.16 | 0.62 | -0.6 | 0.191 |
|  |  |  | 2 | -0.041 | 0.155 | 1 | -0.418 | 0.337 | -0.194 | 0.149 | 0.606 | -0.568 | 0.181 | 0.076 | 0.244 | 1 | -0.526 | 0.678 |
| Insulin | 10mg | 1 | 2 | -0.229 | 0.73 | 1 | -2.008 | 1.551 | -1.588 | 1.697 | 1 | -5.855 | 2.68 | 0.315 | 0.719 | 1 | -1.46 | 2.09 |
|  |  |  | 3 | -0.875 | 0.779 | 0.793 | -2.775 | 1.025 | -1.75 | 1.718 | 0.946 | -6.069 | 2.569 | -0.525 | 0.822 | 1 | -2.552 | 1.502 |
|  |  | 2 | 1 | 0.229 | 0.73 | 1 | -1.551 | 2.008 | 1.588 | 1.697 | 1 | -2.68 | 5.855 | -0.315 | 0.719 | 1 | -2.09 | 1.46 |
|  |  |  | 3 | -0.646 | 0.677 | 1 | -2.297 | 1.004 | -0.162 | 1.352 | 1 | -3.563 | 3.238 | -0.84 | 0.776 | 0.85 | -2.754 | 1.074 |
|  |  | 3 | 1 | 0.875 | 0.779 | 0.793 | -1.025 | 2.775 | 1.75 | 1.718 | 0.946 | -2.569 | 6.069 | 0.525 | 0.822 | 1 | -1.502 | 2.552 |
|  |  |  | 2 | 0.646 | 0.677 | 1 | -1.004 | 2.297 | 0.162 | 1.352 | 1 | -3.238 | 3.563 | 0.84 | 0.776 | 0.85 | -1.074 | 2.754 |
|  | Placebo | 1 | 2 | -0.273 | 0.673 | 1 | -1.912 | 1.367 | -0.293 | 1.283 | 1 | -3.519 | 2.933 | -0.258 | 0.738 | 1 | -2.079 | 1.563 |
|  |  |  | 3 | -0.482 | 0.718 | 1 | -2.232 | 1.268 | -0.021 | 1.298 | 1 | -3.286 | 3.243 | -0.821 | 0.843 | 1 | -2.901 | 1.259 |
|  |  | 2 | 1 | 0.273 | 0.673 | 1 | -1.367 | 1.912 | 0.293 | 1.283 | 1 | -2.933 | 3.519 | 0.258 | 0.738 | 1 | -1.563 | 2.079 |
|  |  |  | 3 | -0.209 | 0.624 | 1 | -1.729 | 1.311 | 0.271 | 1.022 | 1 | -2.299 | 2.842 | -0.563 | 0.796 | 1 | -2.527 | 1.4 |
|  |  | 3 | 1 | 0.482 | 0.718 | 1 | -1.268 | 2.232 | 0.021 | 1.298 | 1 | -3.243 | 3.286 | 0.821 | 0.843 | 1 | -1.259 | 2.901 |
|  |  |  | 2 | 0.209 | 0.624 | 1 | -1.311 | 1.729 | -0.271 | 1.022 | 1 | -2.842 | 2.299 | 0.563 | 0.796 | 1 | -1.4 | 2.527 |
|  | 5mg | 1 | 2 | 0.189 | 0.635 | 1 | -1.359 | 1.737 | 0.394 | 1.2 | 1 | -2.624 | 3.412 | 0.033 | 0.702 | 1 | -1.699 | 1.765 |
|  |  |  | 3 | -0.322 | 0.678 | 1 | -1.974 | 1.331 | -0.8 | 1.215 | 1 | -3.854 | 2.254 | 0.043 | 0.802 | 1 | -1.935 | 2.021 |
|  |  | 2 | 1 | -0.189 | 0.635 | 1 | -1.737 | 1.359 | -0.394 | 1.2 | 1 | -3.412 | 2.624 | -0.033 | 0.702 | 1 | -1.765 | 1.699 |
|  |  |  | 3 | -0.511 | 0.589 | 1 | -1.946 | 0.925 | -1.194 | 0.956 | 0.661 | -3.598 | 1.211 | 0.01 | 0.757 | 1 | -1.858 | 1.877 |
|  |  | 3 | 1 | 0.322 | 0.678 | 1 | -1.331 | 1.974 | 0.8 | 1.215 | 1 | -2.254 | 3.854 | -0.043 | 0.802 | 1 | -2.021 | 1.935 |
|  |  |  | 2 | 0.511 | 0.589 | 1 | -0.925 | 1.946 | 1.194 | 0.956 | 0.661 | -1.211 | 3.598 | -0.01 | 0.757 | 1 | -1.877 | 1.858 |
| WBC | 10mg | 1 | 2 | -0.016 | 0.239 | 1 | -0.598 | 0.566 | -0.825 | 0.593 | 0.52 | -2.317 | 0.667 | 0.265 | 0.207 | 0.613 | -0.244 | 0.774 |
|  |  |  | 3 | 0.174 | 0.187 | 1 | -0.282 | 0.63 | 0.075 | 0.326 | 1 | -0.745 | 0.895 | 0.209 | 0.234 | 1 | -0.368 | 0.785 |
|  |  | 2 | 1 | 0.016 | 0.239 | 1 | -0.566 | 0.598 | 0.825 | 0.593 | 0.52 | -0.667 | 2.317 | -0.265 | 0.207 | 0.613 | -0.774 | 0.244 |
|  |  |  | 3 | 0.19 | 0.252 | 1 | -0.423 | 0.804 | 0.9 | 0.607 | 0.442 | -0.627 | 2.427 | -0.057 | 0.233 | 1 | -0.631 | 0.518 |
|  |  | 3 | 1 | -0.174 | 0.187 | 1 | -0.63 | 0.282 | -0.075 | 0.326 | 1 | -0.895 | 0.745 | -0.209 | 0.234 | 1 | -0.785 | 0.368 |
|  |  |  | 2 | -0.19 | 0.252 | 1 | -0.804 | 0.423 | -0.9 | 0.607 | 0.442 | -2.427 | 0.627 | 0.057 | 0.233 | 1 | -0.518 | 0.631 |
|  | Placebo | 1 | 2 | -0.365 | 0.228 | 0.339 | -0.92 | 0.191 | -0.543 | 0.449 | 0.703 | -1.671 | 0.585 | -0.24 | 0.222 | 0.85 | -0.786 | 0.306 |
|  |  |  | 3 | 0.003 | 0.179 | 1 | -0.433 | 0.439 | 0.171 | 0.247 | 1 | -0.449 | 0.792 | -0.115 | 0.251 | 1 | -0.733 | 0.503 |
|  |  | 2 | 1 | 0.365 | 0.228 | 0.339 | -0.191 | 0.92 | 0.543 | 0.449 | 0.703 | -0.585 | 1.671 | 0.24 | 0.222 | 0.85 | -0.306 | 0.786 |
|  |  |  | 3 | 0.368 | 0.24 | 0.388 | -0.218 | 0.953 | 0.714 | 0.459 | 0.386 | -0.44 | 1.869 | 0.125 | 0.25 | 1 | -0.491 | 0.741 |
|  |  | 3 | 1 | -0.003 | 0.179 | 1 | -0.439 | 0.433 | -0.171 | 0.247 | 1 | -0.792 | 0.449 | 0.115 | 0.251 | 1 | -0.503 | 0.733 |
|  |  |  | 2 | -0.368 | 0.24 | 0.388 | -0.953 | 0.218 | -0.714 | 0.459 | 0.386 | -1.869 | 0.44 | -0.125 | 0.25 | 1 | -0.741 | 0.491 |
|  | 5mg | 1 | 2 | -0.236 | 0.219 | 0.846 | -0.769 | 0.296 | -0.794 | 0.42 | 0.201 | -1.849 | 0.261 | 0.188 | 0.216 | 1 | -0.345 | 0.721 |
|  |  |  | 3 | -.441* | 0.171 | 0.035 | -0.858 | -0.023 | -0.513 | 0.231 | 0.099 | -1.093 | 0.068 | -0.386 | 0.245 | 0.362 | -0.989 | 0.218 |
|  |  | 2 | 1 | 0.236 | 0.219 | 0.846 | -0.296 | 0.769 | 0.794 | 0.42 | 0.201 | -0.261 | 1.849 | -0.188 | 0.216 | 1 | -0.721 | 0.345 |
|  |  |  | 3 | -0.204 | 0.23 | 1 | -0.765 | 0.357 | 0.281 | 0.43 | 1 | -0.799 | 1.361 | -0.574 | 0.244 | 0.066 | -1.175 | 0.027 |
|  |  | 3 | 1 | .441* | 0.171 | 0.035 | 0.023 | 0.858 | 0.513 | 0.231 | 0.099 | -0.068 | 1.093 | 0.386 | 0.245 | 0.362 | -0.218 | 0.989 |
|  |  |  | 2 | 0.204 | 0.23 | 1 | -0.357 | 0.765 | -0.281 | 0.43 | 1 | -1.361 | 0.799 | 0.574 | 0.244 | 0.066 | -0.027 | 1.175 |
| RBC | 10mg | 1 | 2 | -0.064 | 0.043 | 0.424 | -0.169 | 0.041 | -0.005 | 0.075 | 1 | -0.193 | 0.183 | -0.084 | 0.053 | 0.352 | -0.215 | 0.046 |
|  |  |  | 3 | -0.041 | 0.046 | 1 | -0.154 | 0.071 | -0.075 | 0.092 | 1 | -0.307 | 0.157 | -0.03 | 0.053 | 1 | -0.16 | 0.101 |
|  |  | 2 | 1 | 0.064 | 0.043 | 0.424 | -0.041 | 0.169 | 0.005 | 0.075 | 1 | -0.183 | 0.193 | 0.084 | 0.053 | 0.352 | -0.046 | 0.215 |
|  |  |  | 3 | 0.023 | 0.046 | 1 | -0.089 | 0.134 | -0.07 | 0.082 | 1 | -0.277 | 0.137 | 0.055 | 0.056 | 0.989 | -0.082 | 0.192 |
|  |  | 3 | 1 | 0.041 | 0.046 | 1 | -0.071 | 0.154 | 0.075 | 0.092 | 1 | -0.157 | 0.307 | 0.03 | 0.053 | 1 | -0.101 | 0.16 |
|  |  |  | 2 | -0.023 | 0.046 | 1 | -0.134 | 0.089 | 0.07 | 0.082 | 1 | -0.137 | 0.277 | -0.055 | 0.056 | 0.989 | -0.192 | 0.082 |
|  | Placebo | 1 | 2 | 0.082 | 0.041 | 0.146 | -0.018 | 0.182 | 0.011 | 0.056 | 1 | -0.13 | 0.153 | 0.131 | 0.057 | 0.073 | -0.009 | 0.272 |
|  |  |  | 3 | 0 | 0.044 | 1 | -0.107 | 0.108 | -0.096 | 0.07 | 0.538 | -0.271 | 0.08 | 0.068 | 0.057 | 0.72 | -0.073 | 0.208 |
|  |  | 2 | 1 | -0.082 | 0.041 | 0.146 | -0.182 | 0.018 | -0.011 | 0.056 | 1 | -0.153 | 0.13 | -0.131 | 0.057 | 0.073 | -0.272 | 0.009 |
|  |  |  | 3 | -0.082 | 0.044 | 0.191 | -0.188 | 0.024 | -0.107 | 0.062 | 0.281 | -0.264 | 0.049 | -0.064 | 0.06 | 0.866 | -0.211 | 0.083 |
|  |  | 3 | 1 | 0 | 0.044 | 1 | -0.108 | 0.107 | 0.096 | 0.07 | 0.538 | -0.08 | 0.271 | -0.068 | 0.057 | 0.72 | -0.208 | 0.073 |
|  |  |  | 2 | 0.082 | 0.044 | 0.191 | -0.024 | 0.188 | 0.107 | 0.062 | 0.281 | -0.049 | 0.264 | 0.064 | 0.06 | 0.866 | -0.083 | 0.211 |
|  | 5mg | 1 | 2 | -0.093 | 0.039 | 0.06 | -0.189 | 0.003 | -0.064 | 0.053 | 0.704 | -0.196 | 0.069 | -0.116 | 0.056 | 0.125 | -0.253 | 0.021 |
|  |  |  | 3 | -.109* | 0.042 | 0.034 | -0.212 | -0.006 | -0.114 | 0.065 | 0.271 | -0.278 | 0.05 | -0.106 | 0.056 | 0.185 | -0.242 | 0.031 |
|  |  | 2 | 1 | 0.093 | 0.039 | 0.06 | -0.003 | 0.189 | 0.064 | 0.053 | 0.704 | -0.069 | 0.196 | 0.116 | 0.056 | 0.125 | -0.021 | 0.253 |
|  |  |  | 3 | -0.016 | 0.042 | 1 | -0.118 | 0.086 | -0.05 | 0.058 | 1 | -0.196 | 0.096 | 0.01 | 0.058 | 1 | -0.134 | 0.154 |
|  |  | 3 | 1 | .109* | 0.042 | 0.034 | 0.006 | 0.212 | 0.114 | 0.065 | 0.271 | -0.05 | 0.278 | 0.106 | 0.056 | 0.185 | -0.031 | 0.242 |
|  |  |  | 2 | 0.016 | 0.042 | 1 | -0.086 | 0.118 | 0.05 | 0.058 | 1 | -0.096 | 0.196 | -0.01 | 0.058 | 1 | -0.154 | 0.134 |
| Hemoglobin | 10mg | 1 | 2 | 0.019 | 0.143 | 1 | -0.329 | 0.367 | 0.05 | 0.251 | 1 | -0.581 | 0.681 | 0.009 | 0.176 | 1 | -0.425 | 0.442 |
|  |  |  | 3 | 0.071 | 0.157 | 1 | -0.311 | 0.453 | -0.087 | 0.268 | 1 | -0.762 | 0.587 | 0.126 | 0.197 | 1 | -0.36 | 0.612 |
|  |  | 2 | 1 | -0.019 | 0.143 | 1 | -0.367 | 0.329 | -0.05 | 0.251 | 1 | -0.681 | 0.581 | -0.009 | 0.176 | 1 | -0.442 | 0.425 |
|  |  |  | 3 | 0.052 | 0.151 | 1 | -0.317 | 0.42 | -0.137 | 0.263 | 1 | -0.799 | 0.524 | 0.117 | 0.189 | 1 | -0.348 | 0.583 |
|  |  | 3 | 1 | -0.071 | 0.157 | 1 | -0.453 | 0.311 | 0.087 | 0.268 | 1 | -0.587 | 0.762 | -0.126 | 0.197 | 1 | -0.612 | 0.36 |
|  |  |  | 2 | -0.052 | 0.151 | 1 | -0.42 | 0.317 | 0.137 | 0.263 | 1 | -0.524 | 0.799 | -0.117 | 0.189 | 1 | -0.583 | 0.348 |
|  | Placebo | 1 | 2 | 0.165 | 0.136 | 0.69 | -0.167 | 0.497 | -0.029 | 0.19 | 1 | -0.505 | 0.448 | 0.3 | 0.189 | 0.353 | -0.165 | 0.765 |
|  |  |  | 3 | -0.135 | 0.15 | 1 | -0.5 | 0.229 | -0.25 | 0.203 | 0.678 | -0.76 | 0.26 | -0.055 | 0.212 | 1 | -0.576 | 0.466 |
|  |  | 2 | 1 | -0.165 | 0.136 | 0.69 | -0.497 | 0.167 | 0.029 | 0.19 | 1 | -0.448 | 0.505 | -0.3 | 0.189 | 0.353 | -0.765 | 0.165 |
|  |  |  | 3 | -0.3 | 0.145 | 0.122 | -0.652 | 0.052 | -0.221 | 0.199 | 0.819 | -0.721 | 0.279 | -0.355 | 0.203 | 0.255 | -0.854 | 0.144 |
|  |  | 3 | 1 | 0.135 | 0.15 | 1 | -0.229 | 0.5 | 0.25 | 0.203 | 0.678 | -0.26 | 0.76 | 0.055 | 0.212 | 1 | -0.466 | 0.576 |
|  |  |  | 2 | 0.3 | 0.145 | 0.122 | -0.052 | 0.652 | 0.221 | 0.199 | 0.819 | -0.279 | 0.721 | 0.355 | 0.203 | 0.255 | -0.144 | 0.854 |
|  | 5mg | 1 | 2 | -0.192 | 0.131 | 0.436 | -0.51 | 0.127 | -0.05 | 0.177 | 1 | -0.496 | 0.396 | -0.3 | 0.184 | 0.327 | -0.754 | 0.154 |
|  |  |  | 3 | -0.273 | 0.143 | 0.18 | -0.622 | 0.076 | -0.225 | 0.19 | 0.731 | -0.702 | 0.252 | -0.31 | 0.206 | 0.417 | -0.818 | 0.199 |
|  |  | 2 | 1 | 0.192 | 0.131 | 0.436 | -0.127 | 0.51 | 0.05 | 0.177 | 1 | -0.396 | 0.496 | 0.3 | 0.184 | 0.327 | -0.154 | 0.754 |
|  |  |  | 3 | -0.081 | 0.139 | 1 | -0.418 | 0.256 | -0.175 | 0.186 | 1 | -0.643 | 0.293 | -0.01 | 0.198 | 1 | -0.497 | 0.478 |
|  |  | 3 | 1 | 0.273 | 0.143 | 0.18 | -0.076 | 0.622 | 0.225 | 0.19 | 0.731 | -0.252 | 0.702 | 0.31 | 0.206 | 0.417 | -0.199 | 0.818 |
|  |  |  | 2 | 0.081 | 0.139 | 1 | -0.256 | 0.418 | 0.175 | 0.186 | 1 | -0.293 | 0.643 | 0.01 | 0.198 | 1 | -0.478 | 0.497 |
| Hematocrit | 10mg | 1 | 2 | -0.042 | 0.424 | 1 | -1.074 | 0.99 | 0.288 | 0.715 | 1 | -1.51 | 2.085 | -0.157 | 0.532 | 1 | -1.467 | 1.154 |
|  |  |  | 3 | 0.1 | 0.461 | 1 | -1.022 | 1.222 | -0.5 | 0.86 | 1 | -2.661 | 1.661 | 0.309 | 0.553 | 1 | -1.052 | 1.67 |
|  |  | 2 | 1 | 0.042 | 0.424 | 1 | -0.99 | 1.074 | -0.288 | 0.715 | 1 | -2.085 | 1.51 | 0.157 | 0.532 | 1 | -1.154 | 1.467 |
|  |  |  | 3 | 0.142 | 0.451 | 1 | -0.955 | 1.239 | -0.788 | 0.724 | 0.853 | -2.608 | 1.033 | 0.465 | 0.573 | 1 | -0.944 | 1.875 |
|  |  | 3 | 1 | -0.1 | 0.461 | 1 | -1.222 | 1.022 | 0.5 | 0.86 | 1 | -1.661 | 2.661 | -0.309 | 0.553 | 1 | -1.67 | 1.052 |
|  |  |  | 2 | -0.142 | 0.451 | 1 | -1.239 | 0.955 | 0.788 | 0.724 | 0.853 | -1.033 | 2.608 | -0.465 | 0.573 | 1 | -1.875 | 0.944 |
|  | Placebo | 1 | 2 | 0.671 | 0.404 | 0.302 | -0.314 | 1.656 | 0.164 | 0.54 | 1 | -1.194 | 1.523 | 1.025 | 0.571 | 0.233 | -0.38 | 2.43 |
|  |  |  | 3 | -0.203 | 0.44 | 1 | -1.275 | 0.869 | -0.907 | 0.65 | 0.514 | -2.541 | 0.727 | 0.29 | 0.593 | 1 | -1.17 | 1.75 |
|  |  | 2 | 1 | -0.671 | 0.404 | 0.302 | -1.656 | 0.314 | -0.164 | 0.54 | 1 | -1.523 | 1.194 | -1.025 | 0.571 | 0.233 | -2.43 | 0.38 |
|  |  |  | 3 | -0.874 | 0.43 | 0.135 | -1.921 | 0.174 | -1.071 | 0.547 | 0.175 | -2.448 | 0.305 | -0.735 | 0.614 | 0.708 | -2.247 | 0.777 |
|  |  | 3 | 1 | 0.203 | 0.44 | 1 | -0.869 | 1.275 | 0.907 | 0.65 | 0.514 | -0.727 | 2.541 | -0.29 | 0.593 | 1 | -1.75 | 1.17 |
|  |  |  | 2 | 0.874 | 0.43 | 0.135 | -0.174 | 1.921 | 1.071 | 0.547 | 0.175 | -0.305 | 2.448 | 0.735 | 0.614 | 0.708 | -0.777 | 2.247 |
|  | 5mg | 1 | 2 | -0.719 | 0.388 | 0.2 | -1.663 | 0.225 | -0.4 | 0.505 | 1 | -1.671 | 0.871 | -0.962 | 0.557 | 0.268 | -2.333 | 0.409 |
|  |  |  | 3 | -1.024 | 0.422 | 0.051 | -2.052 | 0.003 | -1.163 | 0.608 | 0.192 | -2.691 | 0.366 | -0.919 | 0.579 | 0.352 | -2.344 | 0.505 |
|  |  | 2 | 1 | 0.719 | 0.388 | 0.2 | -0.225 | 1.663 | 0.4 | 0.505 | 1 | -0.871 | 1.671 | 0.962 | 0.557 | 0.268 | -0.409 | 2.333 |
|  |  |  | 3 | -0.305 | 0.412 | 1 | -1.31 | 0.699 | -0.762 | 0.512 | 0.436 | -2.05 | 0.525 | 0.043 | 0.599 | 1 | -1.432 | 1.518 |
|  |  | 3 | 1 | 1.024 | 0.422 | 0.051 | -0.003 | 2.052 | 1.163 | 0.608 | 0.192 | -0.366 | 2.691 | 0.919 | 0.579 | 0.352 | -0.505 | 2.344 |
|  |  |  | 2 | 0.305 | 0.412 | 1 | -0.699 | 1.31 | 0.762 | 0.512 | 0.436 | -0.525 | 2.05 | -0.043 | 0.599 | 1 | -1.518 | 1.432 |
| MCV | 10mg | 1 | 2 | 1.165* | 0.363 | 0.005 | 0.281 | 2.048 | 8.37E-01 | 0.711 | 0.741 | -0.952 | 2.627 | 1.278* | 0.429 | 0.012 | 0.223 | 2.334 |
|  |  |  | 3 | 0.961 | 0.46 | 0.118 | -0.16 | 2.082 | 0.425 | 0.943 | 1 | -1.945 | 2.795 | 1.148 | 0.531 | 0.104 | -0.16 | 2.455 |
|  |  | 2 | 1 | -1.165* | 0.363 | 0.005 | -2.048 | -0.281 | -8.37E-01 | 0.711 | 0.741 | -2.627 | 0.952 | -1.278* | 0.429 | 0.012 | -2.334 | -0.223 |
|  |  |  | 3 | -0.203 | 0.379 | 1 | -1.126 | 0.719 | -0.413 | 0.711 | 1 | -2.201 | 1.376 | -0.13 | 0.459 | 1 | -1.261 | 1 |
|  |  | 3 | 1 | -0.961 | 0.46 | 0.118 | -2.082 | 0.16 | -0.425 | 0.943 | 1 | -2.795 | 1.945 | -1.148 | 0.531 | 0.104 | -2.455 | 0.16 |
|  |  |  | 2 | 0.203 | 0.379 | 1 | -0.719 | 1.126 | 0.413 | 0.711 | 1 | -1.376 | 2.201 | 0.13 | 0.459 | 1 | -1 | 1.261 |
|  | Placebo | 1 | 2 | -0.226 | 0.346 | 1 | -1.07 | 0.617 | 0.114 | 0.538 | 1 | -1.238 | 1.467 | -0.465 | 0.46 | 0.947 | -1.597 | 0.667 |
|  |  |  | 3 | -0.365 | 0.44 | 1 | -1.435 | 0.706 | -0.1 | 0.712 | 1 | -1.892 | 1.692 | -0.55 | 0.57 | 1 | -1.952 | 0.852 |
|  |  | 2 | 1 | 0.226 | 0.346 | 1 | -0.617 | 1.07 | -0.114 | 0.538 | 1 | -1.467 | 1.238 | 0.465 | 0.46 | 0.947 | -0.667 | 1.597 |
|  |  |  | 3 | -0.138 | 0.362 | 1 | -1.019 | 0.743 | -0.214 | 0.538 | 1 | -1.566 | 1.137 | -0.085 | 0.492 | 1 | -1.297 | 1.127 |
|  |  | 3 | 1 | 0.365 | 0.44 | 1 | -0.706 | 1.435 | 0.1 | 0.712 | 1 | -1.692 | 1.892 | 0.55 | 0.57 | 1 | -0.852 | 1.952 |
|  |  |  | 2 | 0.138 | 0.362 | 1 | -0.743 | 1.019 | 0.214 | 0.538 | 1 | -1.137 | 1.566 | 0.085 | 0.492 | 1 | -1.127 | 1.297 |
|  | 5mg | 1 | 2 | 0.268 | 0.332 | 1 | -0.541 | 1.076 | 0.362 | 0.503 | 1 | -0.903 | 1.628 | 0.195 | 0.449 | 1 | -0.909 | 1.3 |
|  |  |  | 3 | -0.049 | 0.421 | 1 | -1.075 | 0.978 | -0.25 | 0.666 | 1 | -1.926 | 1.426 | 0.105 | 0.556 | 1 | -1.264 | 1.473 |
|  |  | 2 | 1 | -0.268 | 0.332 | 1 | -1.076 | 0.541 | -0.362 | 0.503 | 1 | -1.628 | 0.903 | -0.195 | 0.449 | 1 | -1.3 | 0.909 |
|  |  |  | 3 | -0.316 | 0.347 | 1 | -1.161 | 0.528 | -0.612 | 0.503 | 0.694 | -1.877 | 0.652 | -0.09 | 0.48 | 1 | -1.273 | 1.092 |
|  |  | 3 | 1 | 0.049 | 0.421 | 1 | -0.978 | 1.075 | 0.25 | 0.666 | 1 | -1.426 | 1.926 | -0.105 | 0.556 | 1 | -1.473 | 1.264 |
|  |  |  | 2 | 0.316 | 0.347 | 1 | -0.528 | 1.161 | 0.612 | 0.503 | 0.694 | -0.652 | 1.877 | 0.09 | 0.48 | 1 | -1.092 | 1.273 |
| MCH | 10mg | 1 | 2 | .477* | 0.157 | 0.009 | 0.095 | 0.86 | 0.212 | 0.299 | 1 | -0.539 | 0.964 | .570* | 0.188 | 0.011 | 0.107 | 1.033 |
|  |  |  | 3 | 0.442 | 0.189 | 0.064 | -0.018 | 0.902 | 0.363 | 0.342 | 0.888 | -0.497 | 1.222 | 0.47 | 0.228 | 0.132 | -0.093 | 1.032 |
|  |  | 2 | 1 | -.477* | 0.157 | 0.009 | -0.86 | -0.095 | -0.212 | 0.299 | 1 | -0.964 | 0.539 | -.570* | 0.188 | 0.011 | -1.033 | -0.107 |
|  |  |  | 3 | -0.035 | 0.146 | 1 | -0.392 | 0.321 | 0.15 | 0.266 | 1 | -0.52 | 0.82 | -0.1 | 0.175 | 1 | -0.531 | 0.331 |
|  |  | 3 | 1 | -0.442 | 0.189 | 0.064 | -0.902 | 0.018 | -0.363 | 0.342 | 0.888 | -1.222 | 0.497 | -0.47 | 0.228 | 0.132 | -1.032 | 0.093 |
|  |  |  | 2 | 0.035 | 0.146 | 1 | -0.321 | 0.392 | -0.15 | 0.266 | 1 | -0.82 | 0.52 | 0.1 | 0.175 | 1 | -0.331 | 0.531 |
|  | Placebo | 1 | 2 | -0.197 | 0.15 | 0.577 | -0.563 | 0.169 | -0.164 | 0.226 | 1 | -0.732 | 0.404 | -0.22 | 0.202 | 0.839 | -0.717 | 0.277 |
|  |  |  | 3 | -0.241 | 0.18 | 0.551 | -0.68 | 0.198 | 0.107 | 0.258 | 1 | -0.542 | 0.757 | -0.485 | 0.245 | 0.157 | -1.088 | 0.118 |
|  |  | 2 | 1 | 0.197 | 0.15 | 0.577 | -0.169 | 0.563 | 0.164 | 0.226 | 1 | -0.404 | 0.732 | 0.22 | 0.202 | 0.839 | -0.277 | 0.717 |
|  |  |  | 3 | -0.044 | 0.14 | 1 | -0.384 | 0.296 | 0.271 | 0.201 | 0.559 | -0.235 | 0.778 | -0.265 | 0.188 | 0.49 | -0.727 | 0.197 |
|  |  | 3 | 1 | 0.241 | 0.18 | 0.551 | -0.198 | 0.68 | -0.107 | 0.258 | 1 | -0.757 | 0.542 | 0.485 | 0.245 | 0.157 | -0.118 | 1.088 |
|  |  |  | 2 | 0.044 | 0.14 | 1 | -0.296 | 0.384 | -0.271 | 0.201 | 0.559 | -0.778 | 0.235 | 0.265 | 0.188 | 0.49 | -0.197 | 0.727 |
|  | 5mg | 1 | 2 | 0.203 | 0.144 | 0.486 | -0.148 | 0.553 | 0.331 | 0.211 | 0.378 | -0.2 | 0.862 | 0.105 | 0.197 | 1 | -0.38 | 0.589 |
|  |  |  | 3 | 0.151 | 0.173 | 1 | -0.269 | 0.572 | 0.275 | 0.242 | 0.788 | -0.332 | 0.882 | 0.057 | 0.239 | 1 | -0.531 | 0.646 |
|  |  | 2 | 1 | -0.203 | 0.144 | 0.486 | -0.553 | 0.148 | -0.331 | 0.211 | 0.378 | -0.862 | 0.2 | -0.105 | 0.197 | 1 | -0.589 | 0.38 |
|  |  |  | 3 | -0.051 | 0.134 | 1 | -0.377 | 0.275 | -0.056 | 0.188 | 1 | -0.53 | 0.417 | -0.048 | 0.183 | 1 | -0.499 | 0.404 |
|  |  | 3 | 1 | -0.151 | 0.173 | 1 | -0.572 | 0.269 | -0.275 | 0.242 | 0.788 | -0.882 | 0.332 | -0.057 | 0.239 | 1 | -0.646 | 0.531 |
|  |  |  | 2 | 0.051 | 0.134 | 1 | -0.275 | 0.377 | 0.056 | 0.188 | 1 | -0.417 | 0.53 | 0.048 | 0.183 | 1 | -0.404 | 0.499 |
| MCHC | 10mg | 1 | 2 | 0.068 | 0.148 | 1 | -0.292 | 0.428 | -0.113 | 0.257 | 1 | -0.759 | 0.534 | 0.13 | 0.184 | 1 | -0.323 | 0.584 |
|  |  |  | 3 | 0.116 | 0.143 | 1 | -0.233 | 0.465 | 0.2 | 0.246 | 1 | -0.419 | 0.819 | 0.087 | 0.175 | 1 | -0.343 | 0.517 |
|  |  | 2 | 1 | -0.068 | 0.148 | 1 | -0.428 | 0.292 | 0.113 | 0.257 | 1 | -0.534 | 0.759 | -0.13 | 0.184 | 1 | -0.584 | 0.323 |
|  |  |  | 3 | 0.048 | 0.162 | 1 | -0.345 | 0.442 | 0.313 | 0.297 | 0.899 | -0.434 | 1.059 | -0.043 | 0.191 | 1 | -0.513 | 0.426 |
|  |  | 3 | 1 | -0.116 | 0.143 | 1 | -0.465 | 0.233 | -0.2 | 0.246 | 1 | -0.819 | 0.419 | -0.087 | 0.175 | 1 | -0.517 | 0.343 |
|  |  |  | 2 | -0.048 | 0.162 | 1 | -0.442 | 0.345 | -0.313 | 0.297 | 0.899 | -1.059 | 0.434 | 0.043 | 0.191 | 1 | -0.426 | 0.513 |
|  | Placebo | 1 | 2 | -0.124 | 0.141 | 1 | -0.467 | 0.22 | -0.214 | 0.194 | 0.833 | -0.703 | 0.274 | -0.06 | 0.198 | 1 | -0.547 | 0.427 |
|  |  |  | 3 | -0.129 | 0.137 | 1 | -0.463 | 0.204 | 0.157 | 0.186 | 1 | -0.311 | 0.625 | -0.33 | 0.187 | 0.249 | -0.791 | 0.131 |
|  |  | 2 | 1 | 0.124 | 0.141 | 1 | -0.22 | 0.467 | 0.214 | 0.194 | 0.833 | -0.274 | 0.703 | 0.06 | 0.198 | 1 | -0.427 | 0.547 |
|  |  |  | 3 | -0.006 | 0.154 | 1 | -0.382 | 0.37 | 0.371 | 0.224 | 0.32 | -0.193 | 0.936 | -0.27 | 0.204 | 0.575 | -0.773 | 0.233 |
|  |  | 3 | 1 | 0.129 | 0.137 | 1 | -0.204 | 0.463 | -0.157 | 0.186 | 1 | -0.625 | 0.311 | 0.33 | 0.187 | 0.249 | -0.131 | 0.791 |
|  |  |  | 2 | 0.006 | 0.154 | 1 | -0.37 | 0.382 | -0.371 | 0.224 | 0.32 | -0.936 | 0.193 | 0.27 | 0.204 | 0.575 | -0.233 | 0.773 |
|  | 5mg | 1 | 2 | 0.105 | 0.135 | 1 | -0.224 | 0.435 | 0.206 | 0.182 | 0.792 | -0.251 | 0.663 | 0.029 | 0.193 | 1 | -0.446 | 0.504 |
|  |  |  | 3 | 0.181 | 0.131 | 0.512 | -0.138 | 0.501 | 0.394 | 0.174 | 0.09 | -0.044 | 0.831 | 0.019 | 0.183 | 1 | -0.431 | 0.469 |
|  |  | 2 | 1 | -0.105 | 0.135 | 1 | -0.435 | 0.224 | -0.206 | 0.182 | 0.792 | -0.663 | 0.251 | -0.029 | 0.193 | 1 | -0.504 | 0.446 |
|  |  |  | 3 | 0.076 | 0.148 | 1 | -0.285 | 0.436 | 0.188 | 0.21 | 1 | -0.34 | 0.715 | -0.01 | 0.2 | 1 | -0.501 | 0.482 |
|  |  | 3 | 1 | -0.181 | 0.131 | 0.512 | -0.501 | 0.138 | -0.394 | 0.174 | 0.09 | -0.831 | 0.044 | -0.019 | 0.183 | 1 | -0.469 | 0.431 |
|  |  |  | 2 | -0.076 | 0.148 | 1 | -0.436 | 0.285 | -0.188 | 0.21 | 1 | -0.715 | 0.34 | 0.01 | 0.2 | 1 | -0.482 | 0.501 |
| RDW | 10mg | 1 | 2 | -0.168 | 0.11 | 0.393 | -0.436 | 0.1 | -0.15 | 0.177 | 1 | -0.595 | 0.295 | -0.174 | 0.142 | 0.675 | -0.523 | 0.175 |
|  |  |  | 3 | -0.229 | 0.116 | 0.152 | -0.511 | 0.053 | -0.25 | 0.285 | 1 | -0.966 | 0.466 | -0.222 | 0.111 | 0.149 | -0.494 | 0.051 |
|  |  | 2 | 1 | 0.168 | 0.11 | 0.393 | -0.1 | 0.436 | 0.15 | 0.177 | 1 | -0.295 | 0.595 | 0.174 | 0.142 | 0.675 | -0.175 | 0.523 |
|  |  |  | 3 | -0.061 | 0.143 | 1 | -0.409 | 0.286 | -0.1 | 0.331 | 1 | -0.932 | 0.732 | -0.048 | 0.146 | 1 | -0.408 | 0.312 |
|  |  | 3 | 1 | 0.229 | 0.116 | 0.152 | -0.053 | 0.511 | 0.25 | 0.285 | 1 | -0.466 | 0.966 | 0.222 | 0.111 | 0.149 | -0.051 | 0.494 |
|  |  |  | 2 | 0.061 | 0.143 | 1 | -0.286 | 0.409 | 0.1 | 0.331 | 1 | -0.732 | 0.932 | 0.048 | 0.146 | 1 | -0.312 | 0.408 |
|  | Placebo | 1 | 2 | 0.024 | 0.105 | 1 | -0.233 | 0.28 | -0.007 | 0.134 | 1 | -0.344 | 0.329 | 0.045 | 0.152 | 1 | -0.33 | 0.42 |
|  |  |  | 3 | -0.035 | 0.111 | 1 | -0.305 | 0.234 | -0.221 | 0.215 | 0.931 | -0.762 | 0.32 | 0.095 | 0.119 | 1 | -0.197 | 0.387 |
|  |  | 2 | 1 | -0.024 | 0.105 | 1 | -0.28 | 0.233 | 0.007 | 0.134 | 1 | -0.329 | 0.344 | -0.045 | 0.152 | 1 | -0.42 | 0.33 |
|  |  |  | 3 | -0.059 | 0.136 | 1 | -0.39 | 0.273 | -0.214 | 0.25 | 1 | -0.843 | 0.415 | 0.05 | 0.157 | 1 | -0.336 | 0.436 |
|  |  | 3 | 1 | 0.035 | 0.111 | 1 | -0.234 | 0.305 | 0.221 | 0.215 | 0.931 | -0.32 | 0.762 | -0.095 | 0.119 | 1 | -0.387 | 0.197 |
|  |  |  | 2 | 0.059 | 0.136 | 1 | -0.273 | 0.39 | 0.214 | 0.25 | 1 | -0.415 | 0.843 | -0.05 | 0.157 | 1 | -0.436 | 0.336 |
|  | 5mg | 1 | 2 | -.276* | 0.101 | 0.022 | -0.521 | -0.03 | -0.2 | 0.125 | 0.357 | -0.515 | 0.115 | -0.333 | 0.149 | 0.085 | -0.699 | 0.032 |
|  |  |  | 3 | -0.073 | 0.106 | 1 | -0.331 | 0.185 | -0.2 | 0.201 | 0.982 | -0.706 | 0.306 | 0.024 | 0.116 | 1 | -0.261 | 0.309 |
|  |  | 2 | 1 | .276* | 0.101 | 0.022 | 0.03 | 0.521 | 0.2 | 0.125 | 0.357 | -0.115 | 0.515 | 0.333 | 0.149 | 0.085 | -0.032 | 0.699 |
|  |  |  | 3 | 0.203 | 0.131 | 0.371 | -0.115 | 0.521 | 1.24E-14 | 0.234 | 1 | -0.588 | 0.588 | 0.357 | 0.153 | 0.069 | -0.02 | 0.734 |
|  |  | 3 | 1 | 0.073 | 0.106 | 1 | -0.185 | 0.331 | 0.2 | 0.201 | 0.982 | -0.306 | 0.706 | -0.024 | 0.116 | 1 | -0.309 | 0.261 |
|  |  |  | 2 | -0.203 | 0.131 | 0.371 | -0.521 | 0.115 | -1.24E-14 | 0.234 | 1 | -0.588 | 0.588 | -0.357 | 0.153 | 0.069 | -0.734 | 0.02 |
| Platelet | 10mg | 1 | 2 | 9.097 | 4.62 | 0.155 | -2.155 | 20.348 | 12.875 | 8.082 | 0.36 | -7.448 | 33.198 | 7.783 | 5.53 | 0.493 | -5.831 | 21.396 |
|  |  |  | 3 | 1.097 | 5.04 | 1 | -11.178 | 13.372 | 3.625 | 9.223 | 1 | -19.566 | 26.816 | 0.217 | 6.136 | 1 | -14.888 | 15.322 |
|  |  | 2 | 1 | -9.097 | 4.62 | 0.155 | -20.348 | 2.155 | -12.875 | 8.082 | 0.36 | -33.198 | 7.448 | -7.783 | 5.53 | 0.493 | -21.396 | 5.831 |
|  |  |  | 3 | -8 | 5.436 | 0.433 | -21.24 | 5.24 | -9.25 | 8.448 | 0.843 | -30.494 | 11.994 | -7.565 | 6.921 | 0.836 | -24.602 | 9.472 |
|  |  | 3 | 1 | -1.097 | 5.04 | 1 | -13.372 | 11.178 | -3.625 | 9.223 | 1 | -26.816 | 19.566 | -0.217 | 6.136 | 1 | -15.322 | 14.888 |
|  |  |  | 2 | 8 | 5.436 | 0.433 | -5.24 | 21.24 | 9.25 | 8.448 | 0.843 | -11.994 | 30.494 | 7.565 | 6.921 | 0.836 | -9.472 | 24.602 |
|  | Placebo | 1 | 2 | 5.971 | 4.412 | 0.537 | -4.773 | 16.714 | -7 | 6.11 | 0.779 | -22.363 | 8.363 | 15.050* | 5.93 | 0.041 | 0.451 | 29.649 |
|  |  |  | 3 | 8.059 | 4.813 | 0.292 | -3.662 | 19.78 | 6.357 | 6.972 | 1 | -11.174 | 23.888 | 9.25 | 6.58 | 0.495 | -6.948 | 25.448 |
|  |  | 2 | 1 | -5.971 | 4.412 | 0.537 | -16.714 | 4.773 | 7 | 6.11 | 0.779 | -8.363 | 22.363 | -15.050* | 5.93 | 0.041 | -29.649 | -0.451 |
|  |  |  | 3 | 2.088 | 5.191 | 1 | -10.554 | 14.73 | 13.357 | 6.386 | 0.131 | -2.702 | 29.416 | -5.8 | 7.422 | 1 | -24.07 | 12.47 |
|  |  | 3 | 1 | -8.059 | 4.813 | 0.292 | -19.78 | 3.662 | -6.357 | 6.972 | 1 | -23.888 | 11.174 | -9.25 | 6.58 | 0.495 | -25.448 | 6.948 |
|  |  |  | 2 | -2.088 | 5.191 | 1 | -14.73 | 10.554 | -13.357 | 6.386 | 0.131 | -29.416 | 2.702 | 5.8 | 7.422 | 1 | -12.47 | 24.07 |
|  | 5mg | 1 | 2 | -5.73 | 4.229 | 0.536 | -16.029 | 4.569 | -3.375 | 5.715 | 1 | -17.746 | 10.996 | -7.524 | 5.787 | 0.595 | -21.771 | 6.724 |
|  |  |  | 3 | -7.378 | 4.614 | 0.339 | -18.614 | 3.857 | -0.875 | 6.522 | 1 | -17.274 | 15.524 | -12.333 | 6.421 | 0.178 | -28.141 | 3.475 |
|  |  | 2 | 1 | 5.73 | 4.229 | 0.536 | -4.569 | 16.029 | 3.375 | 5.715 | 1 | -10.996 | 17.746 | 7.524 | 5.787 | 0.595 | -6.724 | 21.771 |
|  |  |  | 3 | -1.649 | 4.976 | 1 | -13.767 | 10.47 | 2.5 | 5.974 | 1 | -12.522 | 17.522 | -4.81 | 7.243 | 1 | -22.639 | 13.02 |
|  |  | 3 | 1 | 7.378 | 4.614 | 0.339 | -3.857 | 18.614 | 0.875 | 6.522 | 1 | -15.524 | 17.274 | 12.333 | 6.421 | 0.178 | -3.475 | 28.141 |
|  |  |  | 2 | 1.649 | 4.976 | 1 | -10.47 | 13.767 | -2.5 | 5.974 | 1 | -17.522 | 12.522 | 4.81 | 7.243 | 1 | -13.02 | 22.639 |
| MPV | 10mg | 1 | 2 | 0.032 | 0.075 | 1 | -0.15 | 0.214 | -0.012 | 0.157 | 1 | -0.406 | 0.381 | 0.048 | 0.084 | 1 | -0.159 | 0.255 |
|  |  |  | 3 | 0.048 | 0.069 | 1 | -0.119 | 0.216 | 0 | 0.139 | 1 | -0.349 | 0.349 | 0.065 | 0.08 | 1 | -0.132 | 0.263 |
|  |  | 2 | 1 | -0.032 | 0.075 | 1 | -0.214 | 0.15 | 0.012 | 0.157 | 1 | -0.381 | 0.406 | -0.048 | 0.084 | 1 | -0.255 | 0.159 |
|  |  |  | 3 | 0.016 | 0.073 | 1 | -0.162 | 0.194 | 0.012 | 0.141 | 1 | -0.343 | 0.368 | 0.017 | 0.086 | 1 | -0.196 | 0.23 |
|  |  | 3 | 1 | -0.048 | 0.069 | 1 | -0.216 | 0.119 | 0 | 0.139 | 1 | -0.349 | 0.349 | -0.065 | 0.08 | 1 | -0.263 | 0.132 |
|  |  |  | 2 | -0.016 | 0.073 | 1 | -0.194 | 0.162 | -0.012 | 0.141 | 1 | -0.368 | 0.343 | -0.017 | 0.086 | 1 | -0.23 | 0.196 |
|  | Placebo | 1 | 2 | -0.147 | 0.071 | 0.127 | -0.321 | 0.027 | -0.05 | 0.118 | 1 | -0.348 | 0.248 | -0.215 | 0.09 | 0.061 | -0.437 | 0.007 |
|  |  |  | 3 | -0.135 | 0.066 | 0.125 | -0.295 | 0.024 | -0.107 | 0.105 | 0.944 | -0.371 | 0.157 | -0.155 | 0.086 | 0.229 | -0.367 | 0.057 |
|  |  | 2 | 1 | 0.147 | 0.071 | 0.127 | -0.027 | 0.321 | 0.05 | 0.118 | 1 | -0.248 | 0.348 | 0.215 | 0.09 | 0.061 | -0.007 | 0.437 |
|  |  |  | 3 | 0.012 | 0.07 | 1 | -0.158 | 0.182 | -0.057 | 0.107 | 1 | -0.326 | 0.211 | 0.06 | 0.093 | 1 | -0.168 | 0.288 |
|  |  | 3 | 1 | 0.135 | 0.066 | 0.125 | -0.024 | 0.295 | 0.107 | 0.105 | 0.944 | -0.157 | 0.371 | 0.155 | 0.086 | 0.229 | -0.057 | 0.367 |
|  |  |  | 2 | -0.012 | 0.07 | 1 | -0.182 | 0.158 | 0.057 | 0.107 | 1 | -0.211 | 0.326 | -0.06 | 0.093 | 1 | -0.288 | 0.168 |
|  | 5mg | 1 | 2 | 0.141 | 0.068 | 0.128 | -0.026 | 0.307 | 0.213 | 0.111 | 0.189 | -0.066 | 0.491 | 0.086 | 0.088 | 1 | -0.131 | 0.302 |
|  |  |  | 3 | 0.135 | 0.063 | 0.102 | -0.018 | 0.288 | 0.131 | 0.098 | 0.57 | -0.116 | 0.378 | 0.138 | 0.084 | 0.314 | -0.068 | 0.345 |
|  |  | 2 | 1 | -0.141 | 0.068 | 0.128 | -0.307 | 0.026 | -0.213 | 0.111 | 0.189 | -0.491 | 0.066 | -0.086 | 0.088 | 1 | -0.302 | 0.131 |
|  |  |  | 3 | -0.005 | 0.067 | 1 | -0.168 | 0.157 | -0.081 | 0.1 | 1 | -0.332 | 0.17 | 0.052 | 0.091 | 1 | -0.17 | 0.275 |
|  |  | 3 | 1 | -0.135 | 0.063 | 0.102 | -0.288 | 0.018 | -0.131 | 0.098 | 0.57 | -0.378 | 0.116 | -0.138 | 0.084 | 0.314 | -0.345 | 0.068 |
|  |  |  | 2 | 0.005 | 0.067 | 1 | -0.157 | 0.168 | 0.081 | 0.1 | 1 | -0.17 | 0.332 | -0.052 | 0.091 | 1 | -0.275 | 0.17 |
| Absolute Neutrophils | 10mg | 1 | 2 | -129.29 | 210.774 | 1 | -642.597 | 384.017 | -1003.375 | 477.289 | 0.128 | -2203.538 | 196.788 | 174.739 | 196.776 | 1 | -309.683 | 659.161 |
|  |  |  | 3 | 48.613 | 181.494 | 1 | -393.386 | 490.612 | -236 | 339.081 | 1 | -1088.633 | 616.633 | 147.609 | 219.134 | 1 | -391.854 | 687.071 |
|  |  | 2 | 1 | 129.29 | 210.774 | 1 | -384.017 | 642.597 | 1003.375 | 477.289 | 0.128 | -196.788 | 2203.538 | -174.739 | 196.776 | 1 | -659.161 | 309.683 |
|  |  |  | 3 | 177.903 | 229.893 | 1 | -381.964 | 737.77 | 767.375 | 557.231 | 0.532 | -633.807 | 2168.557 | -27.13 | 216.835 | 1 | -560.933 | 506.672 |
|  |  | 3 | 1 | -48.613 | 181.494 | 1 | -490.612 | 393.386 | 236 | 339.081 | 1 | -616.633 | 1088.633 | -147.609 | 219.134 | 1 | -687.071 | 391.854 |
|  |  |  | 2 | -177.903 | 229.893 | 1 | -737.77 | 381.964 | -767.375 | 557.231 | 0.532 | -2168.557 | 633.807 | 27.13 | 216.835 | 1 | -506.672 | 560.933 |
|  | Placebo | 1 | 2 | -228.912 | 201.261 | 0.774 | -719.05 | 261.226 | -252.571 | 360.796 | 1 | -1159.809 | 654.666 | -212.35 | 211.019 | 0.955 | -731.835 | 307.135 |
|  |  |  | 3 | -97.676 | 173.302 | 1 | -519.725 | 324.372 | -92.714 | 256.321 | 1 | -737.244 | 551.815 | -101.15 | 234.995 | 1 | -679.659 | 477.359 |
|  |  | 2 | 1 | 228.912 | 201.261 | 0.774 | -261.226 | 719.05 | 252.571 | 360.796 | 1 | -654.666 | 1159.809 | 212.35 | 211.019 | 0.955 | -307.135 | 731.835 |
|  |  |  | 3 | 131.235 | 219.516 | 1 | -403.361 | 665.832 | 159.857 | 421.227 | 1 | -899.337 | 1219.051 | 111.2 | 232.529 | 1 | -461.239 | 683.639 |
|  |  | 3 | 1 | 97.676 | 173.302 | 1 | -324.372 | 519.725 | 92.714 | 256.321 | 1 | -551.815 | 737.244 | 101.15 | 234.995 | 1 | -477.359 | 679.659 |
|  |  |  | 2 | -131.235 | 219.516 | 1 | -665.832 | 403.361 | -159.857 | 421.227 | 1 | -1219.051 | 899.337 | -111.2 | 232.529 | 1 | -683.639 | 461.239 |
|  | 5mg | 1 | 2 | -156.297 | 192.929 | 1 | -626.145 | 313.55 | -751.313 | 337.494 | 0.098 | -1599.956 | 97.331 | 297.048 | 205.933 | 0.463 | -209.917 | 804.013 |
|  |  |  | 3 | -413.324* | 166.128 | 0.044 | -817.901 | -8.747 | -565.625 | 239.766 | 0.072 | -1168.527 | 37.277 | -297.286 | 229.332 | 0.599 | -861.853 | 267.281 |
|  |  | 2 | 1 | 156.297 | 192.929 | 1 | -313.55 | 626.145 | 751.313 | 337.494 | 0.098 | -97.331 | 1599.956 | -297.048 | 205.933 | 0.463 | -804.013 | 209.917 |
|  |  |  | 3 | -257.027 | 210.429 | 0.674 | -769.493 | 255.439 | 185.688 | 394.022 | 1 | -805.098 | 1176.473 | -594.333* | 226.925 | 0.033 | -1152.977 | -35.69 |
|  |  | 3 | 1 | 413.324* | 166.128 | 0.044 | 8.747 | 817.901 | 565.625 | 239.766 | 0.072 | -37.277 | 1168.527 | 297.286 | 229.332 | 0.599 | -267.281 | 861.853 |
|  |  |  | 2 | 257.027 | 210.429 | 0.674 | -255.439 | 769.493 | -185.688 | 394.022 | 1 | -1176.473 | 805.098 | 594.333* | 226.925 | 0.033 | 35.69 | 1152.977 |
| Absolute Lymphocytes | 10mg | 1 | 2 | 128.452 | 80.791 | 0.345 | -68.301 | 325.204 | 199.125 | 197.555 | 0.961 | -297.634 | 695.884 | 103.87 | 77.286 | 0.552 | -86.392 | 294.132 |
|  |  |  | 3 | 104.871 | 64.017 | 0.314 | -51.032 | 260.774 | 270 | 124.127 | 0.109 | -42.122 | 582.122 | 47.435 | 73.99 | 1 | -134.713 | 229.583 |
|  |  | 2 | 1 | -128.452 | 80.791 | 0.345 | -325.204 | 68.301 | -199.125 | 197.555 | 0.961 | -695.884 | 297.634 | -103.87 | 77.286 | 0.552 | -294.132 | 86.392 |
|  |  |  | 3 | -23.581 | 76.646 | 1 | -210.24 | 163.079 | 70.875 | 210.771 | 1 | -459.118 | 600.868 | -56.435 | 52.191 | 0.851 | -184.919 | 72.05 |
|  |  | 3 | 1 | -104.871 | 64.017 | 0.314 | -260.774 | 51.032 | -270 | 124.127 | 0.109 | -582.122 | 42.122 | -47.435 | 73.99 | 1 | -229.583 | 134.713 |
|  |  |  | 2 | 23.581 | 76.646 | 1 | -163.079 | 210.24 | -70.875 | 210.771 | 1 | -600.868 | 459.118 | 56.435 | 52.191 | 0.851 | -72.05 | 184.919 |
|  | Placebo | 1 | 2 | -125.382 | 77.144 | 0.322 | -313.254 | 62.489 | -291.357 | 149.337 | 0.177 | -666.872 | 84.158 | -9.2 | 82.88 | 1 | -213.233 | 194.833 |
|  |  |  | 3 | 82.824 | 61.128 | 0.536 | -66.043 | 231.69 | 180.643 | 93.831 | 0.187 | -55.299 | 416.585 | 14.35 | 79.346 | 1 | -180.982 | 209.682 |
|  |  | 2 | 1 | 125.382 | 77.144 | 0.322 | -62.489 | 313.254 | 291.357 | 149.337 | 0.177 | -84.158 | 666.872 | 9.2 | 82.88 | 1 | -194.833 | 213.233 |
|  |  |  | 3 | 208.206* | 73.187 | 0.016 | 29.972 | 386.44 | 472.000* | 159.328 | 0.016 | 71.363 | 872.637 | 23.55 | 55.969 | 1 | -114.234 | 161.334 |
|  |  | 3 | 1 | -82.824 | 61.128 | 0.536 | -231.69 | 66.043 | -180.643 | 93.831 | 0.187 | -416.585 | 55.299 | -14.35 | 79.346 | 1 | -209.682 | 180.982 |
|  |  |  | 2 | -208.206* | 73.187 | 0.016 | -386.44 | -29.972 | -472.000* | 159.328 | 0.016 | -872.637 | -71.363 | -23.55 | 55.969 | 1 | -161.334 | 114.234 |
|  | 5mg | 1 | 2 | -89.135 | 73.95 | 0.693 | -269.229 | 90.959 | -66.75 | 139.692 | 1 | -418.012 | 284.512 | -106.19 | 80.883 | 0.582 | -305.307 | 92.926 |
|  |  |  | 3 | 9.405 | 58.597 | 1 | -133.298 | 152.109 | 55.813 | 87.771 | 1 | -164.891 | 276.516 | -25.952 | 77.433 | 1 | -216.577 | 164.672 |
|  |  | 2 | 1 | 89.135 | 73.95 | 0.693 | -90.959 | 269.229 | 66.75 | 139.692 | 1 | -284.512 | 418.012 | 106.19 | 80.883 | 0.582 | -92.926 | 305.307 |
|  |  |  | 3 | 98.541 | 70.157 | 0.49 | -72.315 | 269.396 | 122.563 | 149.038 | 1 | -252.199 | 497.324 | 80.238 | 54.62 | 0.441 | -54.226 | 214.702 |
|  |  | 3 | 1 | -9.405 | 58.597 | 1 | -152.109 | 133.298 | -55.813 | 87.771 | 1 | -276.516 | 164.891 | 25.952 | 77.433 | 1 | -164.672 | 216.577 |
|  |  |  | 2 | -98.541 | 70.157 | 0.49 | -269.396 | 72.315 | -122.563 | 149.038 | 1 | -497.324 | 252.199 | -80.238 | 54.62 | 0.441 | -214.702 | 54.226 |
| Absolute Monocytes | 10mg | 1 | 2 | -6.194 | 20.346 | 1 | -55.744 | 43.357 | -43 | 43.642 | 0.994 | -152.74 | 66.74 | 6.609 | 22.491 | 1 | -48.759 | 61.977 |
|  |  |  | 3 | 19.258 | 16.152 | 0.708 | -20.078 | 58.594 | 26 | 27.352 | 1 | -42.777 | 94.777 | 16.913 | 19.899 | 1 | -32.074 | 65.9 |
|  |  | 2 | 1 | 6.194 | 20.346 | 1 | -43.357 | 55.744 | 43 | 43.642 | 0.994 | -66.74 | 152.74 | -6.609 | 22.491 | 1 | -61.977 | 48.759 |
|  |  |  | 3 | 25.452 | 23.733 | 0.858 | -32.346 | 83.249 | 69 | 50.144 | 0.533 | -57.088 | 195.088 | 10.304 | 25.522 | 1 | -52.526 | 73.134 |
|  |  | 3 | 1 | -19.258 | 16.152 | 0.708 | -58.594 | 20.078 | -26 | 27.352 | 1 | -94.777 | 42.777 | -16.913 | 19.899 | 1 | -65.9 | 32.074 |
|  |  |  | 2 | -25.452 | 23.733 | 0.858 | -83.249 | 32.346 | -69 | 50.144 | 0.533 | -195.088 | 57.088 | -10.304 | 25.522 | 1 | -73.134 | 52.526 |
|  | Placebo | 1 | 2 | -10.706 | 19.428 | 1 | -58.02 | 36.608 | -28 | 32.99 | 1 | -110.955 | 54.955 | 1.4 | 24.119 | 1 | -57.975 | 60.775 |
|  |  |  | 3 | 17.941 | 15.423 | 0.743 | -19.619 | 55.501 | 49.786 | 20.676 | 0.064 | -2.205 | 101.776 | -4.35 | 21.339 | 1 | -56.882 | 48.182 |
|  |  | 2 | 1 | 10.706 | 19.428 | 1 | -36.608 | 58.02 | 28 | 32.99 | 1 | -54.955 | 110.955 | -1.4 | 24.119 | 1 | -60.775 | 57.975 |
|  |  |  | 3 | 28.647 | 22.662 | 0.627 | -26.542 | 83.836 | 77.786 | 37.905 | 0.143 | -17.528 | 173.099 | -5.75 | 27.369 | 1 | -73.128 | 61.628 |
|  |  | 3 | 1 | -17.941 | 15.423 | 0.743 | -55.501 | 19.619 | -49.786 | 20.676 | 0.064 | -101.776 | 2.205 | 4.35 | 21.339 | 1 | -48.182 | 56.882 |
|  |  |  | 2 | -28.647 | 22.662 | 0.627 | -83.836 | 26.542 | -77.786 | 37.905 | 0.143 | -173.099 | 17.528 | 5.75 | 27.369 | 1 | -61.628 | 73.128 |
|  | 5mg | 1 | 2 | -2.622 | 18.624 | 1 | -47.977 | 42.734 | -16.938 | 30.86 | 1 | -94.535 | 60.66 | 8.286 | 23.538 | 1 | -49.659 | 66.23 |
|  |  |  | 3 | -12.351 | 14.785 | 1 | -48.357 | 23.654 | 7.437 | 19.34 | 1 | -41.195 | 56.07 | -27.429 | 20.825 | 0.578 | -78.695 | 23.838 |
|  |  | 2 | 1 | 2.622 | 18.624 | 1 | -42.734 | 47.977 | 16.938 | 30.86 | 1 | -60.66 | 94.535 | -8.286 | 23.538 | 1 | -66.23 | 49.659 |
|  |  |  | 3 | -9.73 | 21.723 | 1 | -62.634 | 43.174 | 24.375 | 35.457 | 1 | -64.783 | 113.533 | -35.714 | 26.71 | 0.558 | -101.468 | 30.039 |
|  |  | 3 | 1 | 12.351 | 14.785 | 1 | -23.654 | 48.357 | -7.437 | 19.34 | 1 | -56.07 | 41.195 | 27.429 | 20.825 | 0.578 | -23.838 | 78.695 |
|  |  |  | 2 | 9.73 | 21.723 | 1 | -43.174 | 62.634 | -24.375 | 35.457 | 1 | -113.533 | 64.783 | 35.714 | 26.71 | 0.558 | -30.039 | 101.468 |
| Absolute Eosinophils | 10mg | 1 | 2 | -10.871 | 15.977 | 1 | -49.78 | 28.038 | 15.25 | 32.766 | 1 | -67.141 | 97.641 | -19.957 | 17.637 | 0.787 | -63.374 | 23.461 |
|  |  |  | 3 | 4.194 | 19.408 | 1 | -43.071 | 51.459 | 20.5 | 39.368 | 1 | -78.493 | 119.493 | -1.478 | 21.466 | 1 | -54.324 | 51.367 |
|  |  | 2 | 1 | 10.871 | 15.977 | 1 | -28.038 | 49.78 | -15.25 | 32.766 | 1 | -97.641 | 67.141 | 19.957 | 17.637 | 0.787 | -23.461 | 63.374 |
|  |  |  | 3 | 15.065 | 20.964 | 1 | -35.99 | 66.119 | 5.25 | 46.933 | 1 | -112.765 | 123.265 | 18.478 | 22.708 | 1 | -37.423 | 74.379 |
|  |  | 3 | 1 | -4.194 | 19.408 | 1 | -51.459 | 43.071 | -20.5 | 39.368 | 1 | -119.493 | 78.493 | 1.478 | 21.466 | 1 | -51.367 | 54.324 |
|  |  |  | 2 | -15.065 | 20.964 | 1 | -66.119 | 35.99 | -5.25 | 46.933 | 1 | -123.265 | 112.765 | -18.478 | 22.708 | 1 | -74.379 | 37.423 |
|  | Placebo | 1 | 2 | 0.529 | 15.256 | 1 | -36.624 | 37.682 | 28.857 | 24.769 | 0.756 | -33.425 | 91.139 | -19.3 | 18.913 | 0.935 | -65.86 | 27.26 |
|  |  |  | 3 | -3.235 | 18.532 | 1 | -48.367 | 41.896 | 26.214 | 29.76 | 1 | -48.617 | 101.046 | -23.85 | 23.02 | 0.913 | -80.52 | 32.82 |
|  |  | 2 | 1 | -0.529 | 15.256 | 1 | -37.682 | 36.624 | -28.857 | 24.769 | 0.756 | -91.139 | 33.425 | 19.3 | 18.913 | 0.935 | -27.26 | 65.86 |
|  |  |  | 3 | -3.765 | 20.018 | 1 | -52.515 | 44.986 | -2.643 | 35.478 | 1 | -91.854 | 86.568 | -4.55 | 24.351 | 1 | -64.497 | 55.397 |
|  |  | 3 | 1 | 3.235 | 18.532 | 1 | -41.896 | 48.367 | -26.214 | 29.76 | 1 | -101.046 | 48.617 | 23.85 | 23.02 | 0.913 | -32.82 | 80.52 |
|  |  |  | 2 | 3.765 | 20.018 | 1 | -44.986 | 52.515 | 2.643 | 35.478 | 1 | -86.568 | 91.854 | 4.55 | 24.351 | 1 | -55.397 | 64.497 |
|  | 5mg | 1 | 2 | 10.216 | 14.624 | 1 | -25.399 | 45.831 | 36.812 | 23.169 | 0.363 | -21.447 | 95.072 | -10.048 | 18.457 | 1 | -55.486 | 35.39 |
|  |  |  | 3 | -2.838 | 17.765 | 1 | -46.101 | 40.425 | 40.125 | 27.837 | 0.475 | -29.874 | 110.124 | -35.571 | 22.465 | 0.356 | -90.876 | 19.733 |
|  |  | 2 | 1 | -10.216 | 14.624 | 1 | -45.831 | 25.399 | -36.812 | 23.169 | 0.363 | -95.072 | 21.447 | 10.048 | 18.457 | 1 | -35.39 | 55.486 |
|  |  |  | 3 | -13.054 | 19.189 | 1 | -59.786 | 33.678 | 3.313 | 33.187 | 1 | -80.137 | 86.762 | -25.524 | 23.764 | 0.861 | -84.026 | 32.979 |
|  |  | 3 | 1 | 2.838 | 17.765 | 1 | -40.425 | 46.101 | -40.125 | 27.837 | 0.475 | -110.124 | 29.874 | 35.571 | 22.465 | 0.356 | -19.733 | 90.876 |
|  |  |  | 2 | 13.054 | 19.189 | 1 | -33.678 | 59.786 | -3.313 | 33.187 | 1 | -86.762 | 80.137 | 25.524 | 23.764 | 0.861 | -32.979 | 84.026 |
| Absolute Basophils | 10mg | 1 | 2 | 1.613 | 2.857 | 1 | -5.344 | 8.57 | 6.75 | 6.828 | 0.989 | -10.418 | 23.918 | -0.174 | 2.887 | 1 | -7.28 | 6.933 |
|  |  |  | 3 | -1 | 2.88 | 1 | -8.013 | 6.013 | -5.375 | 6.537 | 1 | -21.812 | 11.062 | 0.522 | 2.894 | 1 | -6.602 | 7.645 |
|  |  | 2 | 1 | -1.613 | 2.857 | 1 | -8.57 | 5.344 | -6.75 | 6.828 | 0.989 | -23.918 | 10.418 | 0.174 | 2.887 | 1 | -6.933 | 7.28 |
|  |  |  | 3 | -2.613 | 3.414 | 1 | -10.927 | 5.701 | -12.125 | 7.212 | 0.305 | -30.261 | 6.011 | 0.696 | 3.672 | 1 | -8.345 | 9.736 |
|  |  | 3 | 1 | 1 | 2.88 | 1 | -6.013 | 8.013 | 5.375 | 6.537 | 1 | -11.062 | 21.812 | -0.522 | 2.894 | 1 | -7.645 | 6.602 |
|  |  |  | 2 | 2.613 | 3.414 | 1 | -5.701 | 10.927 | 12.125 | 7.212 | 0.305 | -6.011 | 30.261 | -0.696 | 3.672 | 1 | -9.736 | 8.345 |
|  | Placebo | 1 | 2 | -0.353 | 2.728 | 1 | -6.996 | 6.29 | -7.11E-15 | 5.161 | 1 | -12.978 | 12.978 | -0.6 | 3.096 | 1 | -8.221 | 7.021 |
|  |  |  | 3 | 4.985 | 2.75 | 0.219 | -1.712 | 11.682 | 12.321 | 4.941 | 0.053 | -0.104 | 24.747 | -0.15 | 3.103 | 1 | -7.789 | 7.489 |
|  |  | 2 | 1 | 0.353 | 2.728 | 1 | -6.29 | 6.996 | 7.11E-15 | 5.161 | 1 | -12.978 | 12.978 | 0.6 | 3.096 | 1 | -7.021 | 8.221 |
|  |  |  | 3 | 5.338 | 3.26 | 0.314 | -2.601 | 13.277 | 12.321 | 5.452 | 0.09 | -1.388 | 26.031 | 0.45 | 3.938 | 1 | -9.244 | 10.144 |
|  |  | 3 | 1 | -4.985 | 2.75 | 0.219 | -11.682 | 1.712 | -12.321 | 4.941 | 0.053 | -24.747 | 0.104 | 0.15 | 3.103 | 1 | -7.489 | 7.789 |
|  |  |  | 2 | -5.338 | 3.26 | 0.314 | -13.277 | 2.601 | -12.321 | 5.452 | 0.09 | -26.031 | 1.388 | -0.45 | 3.938 | 1 | -10.144 | 9.244 |
|  | 5mg | 1 | 2 | 3.359 | 2.615 | 0.606 | -3.009 | 9.727 | 4.062 | 4.828 | 1 | -8.077 | 16.202 | 2.824 | 3.021 | 1 | -4.613 | 10.261 |
|  |  |  | 3 | 2.811 | 2.636 | 0.867 | -3.609 | 9.23 | 5.812 | 4.622 | 0.651 | -5.81 | 17.435 | 0.524 | 3.028 | 1 | -6.931 | 7.979 |
|  |  | 2 | 1 | -3.359 | 2.615 | 0.606 | -9.727 | 3.009 | -4.062 | 4.828 | 1 | -16.202 | 8.077 | -2.824 | 3.021 | 1 | -10.261 | 4.613 |
|  |  |  | 3 | -0.549 | 3.125 | 1 | -8.159 | 7.062 | 1.75 | 5.1 | 1 | -11.074 | 14.574 | -2.3 | 3.843 | 1 | -11.761 | 7.161 |
|  |  | 3 | 1 | -2.811 | 2.636 | 0.867 | -9.23 | 3.609 | -5.812 | 4.622 | 0.651 | -17.435 | 5.81 | -0.524 | 3.028 | 1 | -7.979 | 6.931 |
|  |  |  | 2 | 0.549 | 3.125 | 1 | -7.062 | 8.159 | -1.75 | 5.1 | 1 | -14.574 | 11.074 | 2.3 | 3.843 | 1 | -7.161 | 11.761 |
| Neutrophils | 10mg | 1 | 2 | -1.384 | 1.592 | 1 | -5.261 | 2.493 | -6.513 | 2.671 | 0.06 | -13.229 | 0.204 | 0.4 | 1.861 | 1 | -4.181 | 4.981 |
|  |  |  | 3 | -0.9 | 1.501 | 1 | -4.557 | 2.757 | -5.8 | 2.858 | 0.15 | -12.987 | 1.387 | 0.804 | 1.733 | 1 | -3.462 | 5.07 |
|  |  | 2 | 1 | 1.384 | 1.592 | 1 | -2.493 | 5.261 | 6.513 | 2.671 | 0.06 | -0.204 | 13.229 | -0.4 | 1.861 | 1 | -4.981 | 4.181 |
|  |  |  | 3 | 0.484 | 1.511 | 1 | -3.195 | 4.163 | 0.713 | 3.328 | 1 | -7.657 | 9.082 | 0.404 | 1.623 | 1 | -3.59 | 4.399 |
|  |  | 3 | 1 | 0.9 | 1.501 | 1 | -2.757 | 4.557 | 5.8 | 2.858 | 0.15 | -1.387 | 12.987 | -0.804 | 1.733 | 1 | -5.07 | 3.462 |
|  |  |  | 2 | -0.484 | 1.511 | 1 | -4.163 | 3.195 | -0.713 | 3.328 | 1 | -9.082 | 7.657 | -0.404 | 1.623 | 1 | -4.399 | 3.59 |
|  | Placebo | 1 | 2 | -0.932 | 1.52 | 1 | -4.635 | 2.77 | -0.329 | 2.019 | 1 | -5.406 | 4.749 | -1.355 | 1.996 | 1 | -6.268 | 3.558 |
|  |  |  | 3 | -2.085 | 1.434 | 0.447 | -5.577 | 1.406 | -2.786 | 2.161 | 0.617 | -8.218 | 2.647 | -1.595 | 1.858 | 1 | -6.17 | 2.98 |
|  |  | 2 | 1 | 0.932 | 1.52 | 1 | -2.77 | 4.635 | 0.329 | 2.019 | 1 | -4.749 | 5.406 | 1.355 | 1.996 | 1 | -3.558 | 6.268 |
|  |  |  | 3 | -1.153 | 1.442 | 1 | -4.666 | 2.36 | -2.457 | 2.516 | 1 | -8.784 | 3.869 | -0.24 | 1.74 | 1 | -4.523 | 4.043 |
|  |  | 3 | 1 | 2.085 | 1.434 | 0.447 | -1.406 | 5.577 | 2.786 | 2.161 | 0.617 | -2.647 | 8.218 | 1.595 | 1.858 | 1 | -2.98 | 6.17 |
|  |  |  | 2 | 1.153 | 1.442 | 1 | -2.36 | 4.666 | 2.457 | 2.516 | 1 | -3.869 | 8.784 | 0.24 | 1.74 | 1 | -4.043 | 4.523 |
|  | 5mg | 1 | 2 | -0.562 | 1.457 | 1 | -4.111 | 2.987 | -5.288* | 1.889 | 0.025 | -10.037 | -0.538 | 3.038 | 1.948 | 0.372 | -1.757 | 7.833 |
|  |  |  | 3 | -2.503 | 1.374 | 0.215 | -5.85 | 0.844 | -4.794 | 2.021 | 0.07 | -9.876 | 0.288 | -0.757 | 1.814 | 1 | -5.222 | 3.707 |
|  |  | 2 | 1 | 0.562 | 1.457 | 1 | -2.987 | 4.111 | 5.288* | 1.889 | 0.025 | 0.538 | 10.037 | -3.038 | 1.948 | 0.372 | -7.833 | 1.757 |
|  |  |  | 3 | -1.941 | 1.383 | 0.491 | -5.308 | 1.427 | 0.494 | 2.353 | 1 | -5.424 | 6.412 | -3.795 | 1.698 | 0.087 | -7.975 | 0.385 |
|  |  | 3 | 1 | 2.503 | 1.374 | 0.215 | -0.844 | 5.85 | 4.794 | 2.021 | 0.07 | -0.288 | 9.876 | 0.757 | 1.814 | 1 | -3.707 | 5.222 |
|  |  |  | 2 | 1.941 | 1.383 | 0.491 | -1.427 | 5.308 | -0.494 | 2.353 | 1 | -6.412 | 5.424 | 3.795 | 1.698 | 0.087 | -0.385 | 7.975 |
| Lymphocytes | 10mg | 1 | 2 | 1.81 | 1.417 | 0.613 | -1.641 | 5.26 | 5.55 | 2.249 | 0.056 | -0.106 | 11.206 | 0.509 | 1.736 | 1 | -3.765 | 4.783 |
|  |  |  | 3 | 0.881 | 1.235 | 1 | -2.126 | 3.887 | 4.762 | 2.335 | 0.147 | -1.108 | 10.633 | -0.47 | 1.453 | 1 | -4.045 | 3.106 |
|  |  | 2 | 1 | -1.81 | 1.417 | 0.613 | -5.26 | 1.641 | -5.55 | 2.249 | 0.056 | -11.206 | 0.106 | -0.509 | 1.736 | 1 | -4.783 | 3.765 |
|  |  |  | 3 | -0.929 | 1.236 | 1 | -3.938 | 2.08 | -0.788 | 2.56 | 1 | -7.224 | 5.649 | -0.978 | 1.372 | 1 | -4.357 | 2.4 |
|  |  | 3 | 1 | -0.881 | 1.235 | 1 | -3.887 | 2.126 | -4.762 | 2.335 | 0.147 | -10.633 | 1.108 | 0.47 | 1.453 | 1 | -3.106 | 4.045 |
|  |  |  | 2 | 0.929 | 1.236 | 1 | -2.08 | 3.938 | 0.788 | 2.56 | 1 | -5.649 | 7.224 | 0.978 | 1.372 | 1 | -2.4 | 4.357 |
|  | Placebo | 1 | 2 | 0.215 | 1.353 | 1 | -3.08 | 3.509 | -1.314 | 1.7 | 1 | -5.59 | 2.961 | 1.285 | 1.862 | 1 | -3.298 | 5.868 |
|  |  |  | 3 | 1.959 | 1.179 | 0.299 | -0.912 | 4.83 | 1.986 | 1.765 | 0.805 | -2.452 | 6.423 | 1.94 | 1.558 | 0.653 | -1.895 | 5.775 |
|  |  | 2 | 1 | -0.215 | 1.353 | 1 | -3.509 | 3.08 | 1.314 | 1.7 | 1 | -2.961 | 5.59 | -1.285 | 1.862 | 1 | -5.868 | 3.298 |
|  |  |  | 3 | 1.744 | 1.18 | 0.427 | -1.129 | 4.617 | 3.3 | 1.935 | 0.291 | -1.565 | 8.165 | 0.655 | 1.472 | 1 | -2.968 | 4.278 |
|  |  | 3 | 1 | -1.959 | 1.179 | 0.299 | -4.83 | 0.912 | -1.986 | 1.765 | 0.805 | -6.423 | 2.452 | -1.94 | 1.558 | 0.653 | -5.775 | 1.895 |
|  |  |  | 2 | -1.744 | 1.18 | 0.427 | -4.617 | 1.129 | -3.3 | 1.935 | 0.291 | -8.165 | 1.565 | -0.655 | 1.472 | 1 | -4.278 | 2.968 |
|  | 5mg | 1 | 2 | -0.084 | 1.297 | 1 | -3.242 | 3.074 | 3.225 | 1.59 | 0.151 | -0.774 | 7.224 | -2.605 | 1.817 | 0.47 | -7.078 | 1.868 |
|  |  |  | 3 | 1.759 | 1.13 | 0.368 | -0.992 | 4.511 | 2.763 | 1.651 | 0.309 | -1.389 | 6.914 | 0.995 | 1.52 | 1 | -2.747 | 4.738 |
|  |  | 2 | 1 | 0.084 | 1.297 | 1 | -3.074 | 3.242 | -3.225 | 1.59 | 0.151 | -7.224 | 0.774 | 2.605 | 1.817 | 0.47 | -1.868 | 7.078 |
|  |  |  | 3 | 1.843 | 1.131 | 0.319 | -0.911 | 4.597 | -0.463 | 1.81 | 1 | -5.014 | 4.089 | 3.600* | 1.436 | 0.045 | 0.064 | 7.136 |
|  |  | 3 | 1 | -1.759 | 1.13 | 0.368 | -4.511 | 0.992 | -2.763 | 1.651 | 0.309 | -6.914 | 1.389 | -0.995 | 1.52 | 1 | -4.738 | 2.747 |
|  |  |  | 2 | -1.843 | 1.131 | 0.319 | -4.597 | 0.911 | 0.463 | 1.81 | 1 | -4.089 | 5.014 | -3.600* | 1.436 | 0.045 | -7.136 | -0.064 |
| Monocytes | 10mg | 1 | 2 | -0.2 | 0.331 | 1 | -1.006 | 0.606 | 0.2 | 0.567 | 1 | -1.225 | 1.625 | -0.339 | 0.41 | 1 | -1.348 | 0.669 |
|  |  |  | 3 | 0.035 | 0.32 | 1 | -0.743 | 0.814 | 0.475 | 0.746 | 1 | -1.4 | 2.35 | -0.117 | 0.327 | 1 | -0.924 | 0.689 |
|  |  | 2 | 1 | 0.2 | 0.331 | 1 | -0.606 | 1.006 | -0.2 | 0.567 | 1 | -1.625 | 1.225 | 0.339 | 0.41 | 1 | -0.669 | 1.348 |
|  |  |  | 3 | 0.235 | 0.348 | 1 | -0.611 | 1.082 | 0.275 | 0.576 | 1 | -1.174 | 1.724 | 0.222 | 0.444 | 1 | -0.872 | 1.315 |
|  |  | 3 | 1 | -0.035 | 0.32 | 1 | -0.814 | 0.743 | -0.475 | 0.746 | 1 | -2.35 | 1.4 | 0.117 | 0.327 | 1 | -0.689 | 0.924 |
|  |  |  | 2 | -0.235 | 0.348 | 1 | -1.082 | 0.611 | -0.275 | 0.576 | 1 | -1.724 | 1.174 | -0.222 | 0.444 | 1 | -1.315 | 0.872 |
|  | Placebo | 1 | 2 | 0.421 | 0.316 | 0.559 | -0.349 | 1.19 | 0.507 | 0.428 | 0.733 | -0.57 | 1.584 | 0.36 | 0.439 | 1 | -0.722 | 1.442 |
|  |  |  | 3 | 0.044 | 0.305 | 1 | -0.7 | 0.788 | 0.271 | 0.564 | 1 | -1.146 | 1.689 | -0.115 | 0.351 | 1 | -0.979 | 0.749 |
|  |  | 2 | 1 | -0.421 | 0.316 | 0.559 | -1.19 | 0.349 | -0.507 | 0.428 | 0.733 | -1.584 | 0.57 | -0.36 | 0.439 | 1 | -1.442 | 0.722 |
|  |  |  | 3 | -0.376 | 0.332 | 0.779 | -1.185 | 0.432 | -0.236 | 0.436 | 1 | -1.331 | 0.859 | -0.475 | 0.476 | 0.968 | -1.648 | 0.698 |
|  |  | 3 | 1 | -0.044 | 0.305 | 1 | -0.788 | 0.7 | -0.271 | 0.564 | 1 | -1.689 | 1.146 | 0.115 | 0.351 | 1 | -0.749 | 0.979 |
|  |  |  | 2 | 0.376 | 0.332 | 0.779 | -0.432 | 1.185 | 0.236 | 0.436 | 1 | -0.859 | 1.331 | 0.475 | 0.476 | 0.968 | -0.698 | 1.648 |
|  | 5mg | 1 | 2 | 0.268 | 0.303 | 1 | -0.47 | 1.005 | 0.831 | 0.401 | 0.136 | -0.176 | 1.839 | -0.162 | 0.429 | 1 | -1.217 | 0.894 |
|  |  |  | 3 | 0.497 | 0.293 | 0.277 | -0.216 | 1.21 | 0.894 | 0.527 | 0.297 | -0.432 | 2.219 | 0.195 | 0.343 | 1 | -0.648 | 1.039 |
|  |  | 2 | 1 | -0.268 | 0.303 | 1 | -1.005 | 0.47 | -0.831 | 0.401 | 0.136 | -1.839 | 0.176 | 0.162 | 0.429 | 1 | -0.894 | 1.217 |
|  |  |  | 3 | 0.23 | 0.318 | 1 | -0.545 | 1.005 | 0.063 | 0.407 | 1 | -0.962 | 1.087 | 0.357 | 0.465 | 1 | -0.787 | 1.502 |
|  |  | 3 | 1 | -0.497 | 0.293 | 0.277 | -1.21 | 0.216 | -0.894 | 0.527 | 0.297 | -2.219 | 0.432 | -0.195 | 0.343 | 1 | -1.039 | 0.648 |
|  |  |  | 2 | -0.23 | 0.318 | 1 | -1.005 | 0.545 | -0.063 | 0.407 | 1 | -1.087 | 0.962 | -0.357 | 0.465 | 1 | -1.502 | 0.787 |
| Eosinophils | 10mg | 1 | 2 | -0.281 | 0.366 | 1 | -1.171 | 0.61 | 0.525 | 0.82 | 1 | -1.536 | 2.586 | -0.561 | 0.361 | 0.376 | -1.449 | 0.328 |
|  |  |  | 3 | 0.013 | 0.33 | 1 | -0.79 | 0.816 | 0.612 | 0.686 | 1 | -1.113 | 2.338 | -0.196 | 0.354 | 1 | -1.068 | 0.677 |
|  |  | 2 | 1 | 0.281 | 0.366 | 1 | -0.61 | 1.171 | -0.525 | 0.82 | 1 | -2.586 | 1.536 | 0.561 | 0.361 | 0.376 | -0.328 | 1.449 |
|  |  |  | 3 | 0.294 | 0.394 | 1 | -0.666 | 1.253 | 0.087 | 0.928 | 1 | -2.247 | 2.422 | 0.365 | 0.405 | 1 | -0.632 | 1.362 |
|  |  | 3 | 1 | -0.013 | 0.33 | 1 | -0.816 | 0.79 | -0.612 | 0.686 | 1 | -2.338 | 1.113 | 0.196 | 0.354 | 1 | -0.677 | 1.068 |
|  |  |  | 2 | -0.294 | 0.394 | 1 | -1.253 | 0.666 | -0.087 | 0.928 | 1 | -2.422 | 2.247 | -0.365 | 0.405 | 1 | -1.362 | 0.632 |
|  | Placebo | 1 | 2 | 0.232 | 0.349 | 1 | -0.618 | 1.083 | 1.071 | 0.62 | 0.278 | -0.486 | 2.629 | -0.355 | 0.387 | 1 | -1.308 | 0.598 |
|  |  |  | 3 | 0.012 | 0.315 | 1 | -0.755 | 0.779 | 0.421 | 0.519 | 1 | -0.883 | 1.726 | -0.275 | 0.38 | 1 | -1.211 | 0.661 |
|  |  | 2 | 1 | -0.232 | 0.349 | 1 | -1.083 | 0.618 | -1.071 | 0.62 | 0.278 | -2.629 | 0.486 | 0.355 | 0.387 | 1 | -0.598 | 1.308 |
|  |  |  | 3 | -0.221 | 0.376 | 1 | -1.137 | 0.696 | -0.65 | 0.702 | 1 | -2.415 | 1.115 | 0.08 | 0.434 | 1 | -0.989 | 1.149 |
|  |  | 3 | 1 | -0.012 | 0.315 | 1 | -0.779 | 0.755 | -0.421 | 0.519 | 1 | -1.726 | 0.883 | 0.275 | 0.38 | 1 | -0.661 | 1.211 |
|  |  |  | 2 | 0.221 | 0.376 | 1 | -0.696 | 1.137 | 0.65 | 0.702 | 1 | -1.115 | 2.415 | -0.08 | 0.434 | 1 | -1.149 | 0.989 |
|  | 5mg | 1 | 2 | 0.286 | 0.335 | 1 | -0.529 | 1.101 | 1.031 | 0.58 | 0.252 | -0.426 | 2.488 | -0.281 | 0.378 | 1 | -1.211 | 0.649 |
|  |  |  | 3 | 0.149 | 0.302 | 1 | -0.587 | 0.884 | 0.944 | 0.485 | 0.18 | -0.277 | 2.164 | -0.457 | 0.371 | 0.668 | -1.37 | 0.456 |
|  |  | 2 | 1 | -0.286 | 0.335 | 1 | -1.101 | 0.529 | -1.031 | 0.58 | 0.252 | -2.488 | 0.426 | 0.281 | 0.378 | 1 | -0.649 | 1.211 |
|  |  |  | 3 | -0.138 | 0.361 | 1 | -1.016 | 0.74 | -0.088 | 0.657 | 1 | -1.738 | 1.563 | -0.176 | 0.424 | 1 | -1.219 | 0.867 |
|  |  | 3 | 1 | -0.149 | 0.302 | 1 | -0.884 | 0.587 | -0.944 | 0.485 | 0.18 | -2.164 | 0.277 | 0.457 | 0.371 | 0.668 | -0.456 | 1.37 |
|  |  |  | 2 | 0.138 | 0.361 | 1 | -0.74 | 1.016 | 0.088 | 0.657 | 1 | -1.563 | 1.738 | 0.176 | 0.424 | 1 | -0.867 | 1.219 |
| Basophils | 10mg | 1 | 2 | 0.055 | 0.077 | 1 | -0.132 | 0.242 | 0.238 | 0.208 | 0.783 | -0.285 | 0.76 | -0.009 | 0.06 | 1 | -0.158 | 0.14 |
|  |  |  | 3 | -0.029 | 0.051 | 1 | -0.153 | 0.095 | -0.05 | 0.105 | 1 | -0.314 | 0.214 | -0.022 | 0.057 | 1 | -0.163 | 0.119 |
|  |  | 2 | 1 | -0.055 | 0.077 | 1 | -0.242 | 0.132 | -0.238 | 0.208 | 0.783 | -0.76 | 0.285 | 0.009 | 0.06 | 1 | -0.14 | 0.158 |
|  |  |  | 3 | -0.084 | 0.081 | 0.907 | -0.281 | 0.113 | -0.288 | 0.209 | 0.535 | -0.814 | 0.239 | -0.013 | 0.07 | 1 | -0.186 | 0.16 |
|  |  | 3 | 1 | 0.029 | 0.051 | 1 | -0.095 | 0.153 | 0.05 | 0.105 | 1 | -0.214 | 0.314 | 0.022 | 0.057 | 1 | -0.119 | 0.163 |
|  |  |  | 2 | 0.084 | 0.081 | 0.907 | -0.113 | 0.281 | 0.288 | 0.209 | 0.535 | -0.239 | 0.814 | 0.013 | 0.07 | 1 | -0.16 | 0.186 |
|  | Placebo | 1 | 2 | -0.041 | 0.073 | 1 | -0.22 | 0.137 | -0.129 | 0.157 | 1 | -0.524 | 0.267 | 0.02 | 0.065 | 1 | -0.14 | 0.18 |
|  |  |  | 3 | 0.059 | 0.049 | 0.693 | -0.06 | 0.178 | 0.107 | 0.079 | 0.555 | -0.092 | 0.306 | 0.025 | 0.062 | 1 | -0.126 | 0.176 |
|  |  | 2 | 1 | 0.041 | 0.073 | 1 | -0.137 | 0.22 | 0.129 | 0.157 | 1 | -0.267 | 0.524 | -0.02 | 0.065 | 1 | -0.18 | 0.14 |
|  |  |  | 3 | 0.1 | 0.077 | 0.595 | -0.088 | 0.288 | 0.236 | 0.158 | 0.436 | -0.162 | 0.634 | 0.005 | 0.075 | 1 | -0.18 | 0.19 |
|  |  | 3 | 1 | -0.059 | 0.049 | 0.693 | -0.178 | 0.06 | -0.107 | 0.079 | 0.555 | -0.306 | 0.092 | -0.025 | 0.062 | 1 | -0.176 | 0.126 |
|  |  |  | 2 | -0.1 | 0.077 | 0.595 | -0.288 | 0.088 | -0.236 | 0.158 | 0.436 | -0.634 | 0.162 | -0.005 | 0.075 | 1 | -0.19 | 0.18 |
|  | 5mg | 1 | 2 | 0.092 | 0.07 | 0.583 | -0.079 | 0.263 | 0.2 | 0.147 | 0.547 | -0.17 | 0.57 | 0.01 | 0.063 | 1 | -0.146 | 0.165 |
|  |  |  | 3 | 0.097 | 0.047 | 0.12 | -0.017 | 0.211 | .194* | 0.074 | 0.039 | 0.007 | 0.38 | 0.024 | 0.06 | 1 | -0.124 | 0.172 |
|  |  | 2 | 1 | -0.092 | 0.07 | 0.583 | -0.263 | 0.079 | -0.2 | 0.147 | 0.547 | -0.57 | 0.17 | -0.01 | 0.063 | 1 | -0.165 | 0.146 |
|  |  |  | 3 | 0.005 | 0.074 | 1 | -0.175 | 0.186 | -0.006 | 0.148 | 1 | -0.378 | 0.366 | 0.014 | 0.074 | 1 | -0.167 | 0.195 |
|  |  | 3 | 1 | -0.097 | 0.047 | 0.12 | -0.211 | 0.017 | -.194* | 0.074 | 0.039 | -0.38 | -0.007 | -0.024 | 0.06 | 1 | -0.172 | 0.124 |
|  |  |  | 2 | -0.005 | 0.074 | 1 | -0.186 | 0.175 | 0.006 | 0.148 | 1 | -0.366 | 0.378 | -0.014 | 0.074 | 1 | -0.195 | 0.167 |
| DHEA | 10mg | 1 | 2 | 4.273 | 27.711 | 1 | -65.62 | 74.165 | 7.333 | 11.542 | 1 | -24.36 | 39.026 | 3.125 | 41.786 | 1 | -107.816 | 114.066 |
|  |  |  | 3 | 7.909 | 23.002 | 1 | -50.108 | 65.926 | -4.667 | 15.119 | 1 | -46.182 | 36.849 | 12.625 | 33.085 | 1 | -75.215 | 100.465 |
|  |  | 2 | 1 | -4.273 | 27.711 | 1 | -74.165 | 65.62 | -7.333 | 11.542 | 1 | -39.026 | 24.36 | -3.125 | 41.786 | 1 | -114.066 | 107.816 |
|  |  |  | 3 | 3.636 | 13.728 | 1 | -30.988 | 38.26 | -12 | 11.102 | 0.898 | -42.485 | 18.485 | 9.5 | 21.329 | 1 | -47.128 | 66.128 |
|  |  | 3 | 1 | -7.909 | 23.002 | 1 | -65.926 | 50.108 | 4.667 | 15.119 | 1 | -36.849 | 46.182 | -12.625 | 33.085 | 1 | -100.465 | 75.215 |
|  |  |  | 2 | -3.636 | 13.728 | 1 | -38.26 | 30.988 | 12 | 11.102 | 0.898 | -18.485 | 42.485 | -9.5 | 21.329 | 1 | -66.128 | 47.128 |
|  | Placebo | 1 | 2 | 24.929 | 24.563 | 0.953 | -37.025 | 86.882 | -5.625 | 7.068 | 1 | -25.033 | 13.783 | 65.667 | 48.25 | 0.574 | -62.437 | 193.77 |
|  |  |  | 3 | 28.357 | 20.39 | 0.521 | -23.07 | 79.784 | -5.125 | 9.258 | 1 | -30.548 | 20.298 | 73 | 38.203 | 0.219 | -28.429 | 174.429 |
|  |  | 2 | 1 | -24.929 | 24.563 | 0.953 | -86.882 | 37.025 | 5.625 | 7.068 | 1 | -13.783 | 25.033 | -65.667 | 48.25 | 0.574 | -193.77 | 62.437 |
|  |  |  | 3 | 3.429 | 12.168 | 1 | -27.262 | 34.119 | 0.5 | 6.798 | 1 | -18.168 | 19.168 | 7.333 | 24.628 | 1 | -58.055 | 72.721 |
|  |  | 3 | 1 | -28.357 | 20.39 | 0.521 | -79.784 | 23.07 | 5.125 | 9.258 | 1 | -20.298 | 30.548 | -73 | 38.203 | 0.219 | -174.429 | 28.429 |
|  |  |  | 2 | -3.429 | 12.168 | 1 | -34.119 | 27.262 | -0.5 | 6.798 | 1 | -19.168 | 18.168 | -7.333 | 24.628 | 1 | -72.721 | 58.055 |
|  | 5mg | 1 | 2 | 46.273 | 27.711 | 0.313 | -23.62 | 116.165 | 1.2 | 8.94 | 1 | -23.349 | 25.749 | 83.833 | 48.25 | 0.301 | -44.27 | 211.937 |
|  |  |  | 3 | 41.636 | 23.002 | 0.238 | -16.381 | 99.654 | 4.4 | 11.711 | 1 | -27.758 | 36.558 | 72.667 | 38.203 | 0.223 | -28.762 | 174.095 |
|  |  | 2 | 1 | -46.273 | 27.711 | 0.313 | -116.165 | 23.62 | -1.2 | 8.94 | 1 | -25.749 | 23.349 | -83.833 | 48.25 | 0.301 | -211.937 | 44.27 |
|  |  |  | 3 | -4.636 | 13.728 | 1 | -39.26 | 29.988 | 3.2 | 8.599 | 1 | -20.414 | 26.814 | -11.167 | 24.628 | 1 | -76.555 | 54.221 |
|  |  | 3 | 1 | -41.636 | 23.002 | 0.238 | -99.654 | 16.381 | -4.4 | 11.711 | 1 | -36.558 | 27.758 | -72.667 | 38.203 | 0.223 | -174.095 | 28.762 |
|  |  |  | 2 | 4.636 | 13.728 | 1 | -29.988 | 39.26 | -3.2 | 8.599 | 1 | -26.814 | 20.414 | 11.167 | 24.628 | 1 | -54.221 | 76.555 |
| Vitamin D, 25-OH | 10mg | 1 | 2 | 6.091 | 3.238 | 0.206 | -2.075 | 14.257 | 9.333 | 6.277 | 0.483 | -7.904 | 26.57 | 4.875 | 3.834 | 0.662 | -5.304 | 15.054 |
|  |  |  | 3 | 0.273 | 4.032 | 1 | -9.897 | 10.443 | 10.333 | 7.544 | 0.582 | -10.383 | 31.05 | -3.5 | 4.615 | 1 | -15.752 | 8.752 |
|  |  | 2 | 1 | -6.091 | 3.238 | 0.206 | -14.257 | 2.075 | -9.333 | 6.277 | 0.483 | -26.57 | 7.904 | -4.875 | 3.834 | 0.662 | -15.054 | 5.304 |
|  |  |  | 3 | -5.818 | 3.241 | 0.245 | -13.993 | 2.357 | 1 | 6.751 | 1 | -17.538 | 19.538 | -8.375 | 3.274 | 0.061 | -17.069 | 0.319 |
|  |  | 3 | 1 | -0.273 | 4.032 | 1 | -10.443 | 9.897 | -10.333 | 7.544 | 0.582 | -31.05 | 10.383 | 3.5 | 4.615 | 1 | -8.752 | 15.752 |
|  |  |  | 2 | 5.818 | 3.241 | 0.245 | -2.357 | 13.993 | -1 | 6.751 | 1 | -19.538 | 17.538 | 8.375 | 3.274 | 0.061 | -0.319 | 17.069 |
|  | Placebo | 1 | 2 | 2.429 | 2.87 | 1 | -4.81 | 9.667 | 2.125 | 3.844 | 1 | -8.43 | 12.68 | 2.833 | 4.427 | 1 | -8.92 | 14.587 |
|  |  |  | 3 | 1.643 | 3.574 | 1 | -7.372 | 10.658 | -2.625 | 4.62 | 1 | -15.311 | 10.061 | 7.333 | 5.329 | 0.56 | -6.814 | 21.481 |
|  |  | 2 | 1 | -2.429 | 2.87 | 1 | -9.667 | 4.81 | -2.125 | 3.844 | 1 | -12.68 | 8.43 | -2.833 | 4.427 | 1 | -14.587 | 8.92 |
|  |  |  | 3 | -0.786 | 2.873 | 1 | -8.032 | 6.461 | -4.75 | 4.134 | 0.814 | -16.102 | 6.602 | 4.5 | 3.781 | 0.751 | -5.539 | 14.539 |
|  |  | 3 | 1 | -1.643 | 3.574 | 1 | -10.658 | 7.372 | 2.625 | 4.62 | 1 | -10.061 | 15.311 | -7.333 | 5.329 | 0.56 | -21.481 | 6.814 |
|  |  |  | 2 | 0.786 | 2.873 | 1 | -6.461 | 8.032 | 4.75 | 4.134 | 0.814 | -6.602 | 16.102 | -4.5 | 3.781 | 0.751 | -14.539 | 5.539 |
|  | 5mg | 1 | 2 | 4.545 | 3.238 | 0.509 | -3.621 | 12.711 | -0.4 | 4.862 | 1 | -13.752 | 12.952 | 8.667 | 4.427 | 0.201 | -3.087 | 20.42 |
|  |  |  | 3 | 3.364 | 4.032 | 1 | -6.806 | 13.534 | 1.6 | 5.844 | 1 | -14.447 | 17.647 | 4.833 | 5.329 | 1 | -9.314 | 18.981 |
|  |  | 2 | 1 | -4.545 | 3.238 | 0.509 | -12.711 | 3.621 | 0.4 | 4.862 | 1 | -12.952 | 13.752 | -8.667 | 4.427 | 0.201 | -20.42 | 3.087 |
|  |  |  | 3 | -1.182 | 3.241 | 1 | -9.357 | 6.993 | 2 | 5.229 | 1 | -12.359 | 16.359 | -3.833 | 3.781 | 0.975 | -13.872 | 6.205 |
|  |  | 3 | 1 | -3.364 | 4.032 | 1 | -13.534 | 6.806 | -1.6 | 5.844 | 1 | -17.647 | 14.447 | -4.833 | 5.329 | 1 | -18.981 | 9.314 |
|  |  |  | 2 | 1.182 | 3.241 | 1 | -6.993 | 9.357 | -2 | 5.229 | 1 | -16.359 | 12.359 | 3.833 | 3.781 | 0.975 | -6.205 | 13.872 |
| Hemoglobin A1C | 10mg | 1 | 2 | -0.027 | 0.04 | 1 | -0.127 | 0.073 | -0.067 | 0.077 | 1 | -0.279 | 0.146 | -0.013 | 0.049 | 1 | -0.143 | 0.118 |
|  |  |  | 3 | -0.118 | 0.055 | 0.115 | -0.256 | 0.02 | -0.167 | 0.087 | 0.236 | -0.406 | 0.073 | -0.1 | 0.072 | 0.545 | -0.291 | 0.091 |
|  |  | 2 | 1 | 0.027 | 0.04 | 1 | -0.073 | 0.127 | 0.067 | 0.077 | 1 | -0.146 | 0.279 | 0.013 | 0.049 | 1 | -0.118 | 0.143 |
|  |  |  | 3 | -0.091 | 0.051 | 0.255 | -0.22 | 0.038 | -0.1 | 0.111 | 1 | -0.406 | 0.206 | -0.087 | 0.055 | 0.387 | -0.233 | 0.058 |
|  |  | 3 | 1 | 0.118 | 0.055 | 0.115 | -0.02 | 0.256 | 0.167 | 0.087 | 0.236 | -0.073 | 0.406 | 0.1 | 0.072 | 0.545 | -0.091 | 0.291 |
|  |  |  | 2 | 0.091 | 0.051 | 0.255 | -0.038 | 0.22 | 0.1 | 0.111 | 1 | -0.206 | 0.406 | 0.087 | 0.055 | 0.387 | -0.058 | 0.233 |
|  | Placebo | 1 | 2 | 0.05 | 0.035 | 0.495 | -0.039 | 0.139 | 0.062 | 0.047 | 0.63 | -0.068 | 0.193 | 0.033 | 0.057 | 1 | -0.118 | 0.184 |
|  |  |  | 3 | -0.007 | 0.049 | 1 | -0.13 | 0.115 | 0.038 | 0.053 | 1 | -0.109 | 0.184 | -0.067 | 0.083 | 1 | -0.287 | 0.154 |
|  |  | 2 | 1 | -0.05 | 0.035 | 0.495 | -0.139 | 0.039 | -0.062 | 0.047 | 0.63 | -0.193 | 0.068 | -0.033 | 0.057 | 1 | -0.184 | 0.118 |
|  |  |  | 3 | -0.057 | 0.045 | 0.65 | -0.172 | 0.057 | -0.025 | 0.068 | 1 | -0.212 | 0.162 | -0.1 | 0.063 | 0.398 | -0.268 | 0.068 |
|  |  | 3 | 1 | 0.007 | 0.049 | 1 | -0.115 | 0.13 | -0.038 | 0.053 | 1 | -0.184 | 0.109 | 0.067 | 0.083 | 1 | -0.154 | 0.287 |
|  |  |  | 2 | 0.057 | 0.045 | 0.65 | -0.057 | 0.172 | 0.025 | 0.068 | 1 | -0.162 | 0.212 | 0.1 | 0.063 | 0.398 | -0.068 | 0.268 |
|  | 5mg | 1 | 2 | 0.018 | 0.04 | 1 | -0.082 | 0.118 | 0.02 | 0.06 | 1 | -0.145 | 0.185 | 0.017 | 0.057 | 1 | -0.134 | 0.168 |
|  |  |  | 3 | -0.127 | 0.055 | 0.079 | -0.265 | 0.011 | -0.06 | 0.068 | 1 | -0.246 | 0.126 | -0.183 | 0.083 | 0.123 | -0.404 | 0.037 |
|  |  | 2 | 1 | -0.018 | 0.04 | 1 | -0.118 | 0.082 | -0.02 | 0.06 | 1 | -0.185 | 0.145 | -0.017 | 0.057 | 1 | -0.168 | 0.134 |
|  |  |  | 3 | -.145* | 0.051 | 0.023 | -0.275 | -0.016 | -0.08 | 0.086 | 1 | -0.317 | 0.157 | -.200* | 0.063 | 0.017 | -0.368 | -0.032 |
|  |  | 3 | 1 | 0.127 | 0.055 | 0.079 | -0.011 | 0.265 | 0.06 | 0.068 | 1 | -0.126 | 0.246 | 0.183 | 0.083 | 0.123 | -0.037 | 0.404 |
|  |  |  | 2 | .145* | 0.051 | 0.023 | 0.016 | 0.275 | 0.08 | 0.086 | 1 | -0.157 | 0.317 | .200* | 0.063 | 0.017 | 0.032 | 0.368 |
| ApoB | 10mg | 1 | 2 | 5.636 | 4.225 | 0.574 | -5.019 | 16.292 | -1.333 | 7.163 | 1 | -21.002 | 18.335 | 8.25 | 5.334 | 0.421 | -5.912 | 22.412 |
|  |  |  | 3 | 6.909 | 4.656 | 0.442 | -4.834 | 18.652 | 3.333 | 6.83 | 1 | -15.423 | 22.089 | 8.25 | 6.634 | 0.692 | -9.362 | 25.862 |
|  |  | 2 | 1 | -5.636 | 4.225 | 0.574 | -16.292 | 5.019 | 1.333 | 7.163 | 1 | -18.335 | 21.002 | -8.25 | 5.334 | 0.421 | -22.412 | 5.912 |
|  |  |  | 3 | 1.273 | 4.696 | 1 | -10.571 | 13.116 | 4.667 | 8.835 | 1 | -19.594 | 28.927 | 1.42E-14 | 5.816 | 1 | -15.442 | 15.442 |
|  |  | 3 | 1 | -6.909 | 4.656 | 0.442 | -18.652 | 4.834 | -3.333 | 6.83 | 1 | -22.089 | 15.423 | -8.25 | 6.634 | 0.692 | -25.862 | 9.362 |
|  |  |  | 2 | -1.273 | 4.696 | 1 | -13.116 | 10.571 | -4.667 | 8.835 | 1 | -28.927 | 19.594 | -1.42E-14 | 5.816 | 1 | -15.442 | 15.442 |
|  | Placebo | 1 | 2 | 9.286 | 3.745 | 0.055 | -0.16 | 18.731 | 4.75 | 4.386 | 0.896 | -7.295 | 16.795 | 15.333 | 6.159 | 0.07 | -1.02 | 31.686 |
|  |  |  | 3 | 10.357 | 4.127 | 0.052 | -0.052 | 20.766 | 9.875 | 4.183 | 0.104 | -1.611 | 21.361 | 11 | 7.66 | 0.507 | -9.337 | 31.337 |
|  |  | 2 | 1 | -9.286 | 3.745 | 0.055 | -18.731 | 0.16 | -4.75 | 4.386 | 0.896 | -16.795 | 7.295 | -15.333 | 6.159 | 0.07 | -31.686 | 1.02 |
|  |  |  | 3 | 1.071 | 4.162 | 1 | -9.427 | 11.57 | 5.125 | 5.41 | 1 | -9.732 | 19.982 | -4.333 | 6.716 | 1 | -22.165 | 13.498 |
|  |  | 3 | 1 | -10.357 | 4.127 | 0.052 | -20.766 | 0.052 | -9.875 | 4.183 | 0.104 | -21.361 | 1.611 | -11 | 7.66 | 0.507 | -31.337 | 9.337 |
|  |  |  | 2 | -1.071 | 4.162 | 1 | -11.57 | 9.427 | -5.125 | 5.41 | 1 | -19.982 | 9.732 | 4.333 | 6.716 | 1 | -13.498 | 22.165 |
|  | 5mg | 1 | 2 | 0.182 | 4.225 | 1 | -10.474 | 10.838 | 1.8 | 5.548 | 1 | -13.435 | 17.035 | -1.167 | 6.159 | 1 | -17.52 | 15.186 |
|  |  |  | 3 | 0.091 | 4.656 | 1 | -11.652 | 11.834 | 1 | 5.291 | 1 | -13.528 | 15.528 | -0.667 | 7.66 | 1 | -21.004 | 19.67 |
|  |  | 2 | 1 | -0.182 | 4.225 | 1 | -10.838 | 10.474 | -1.8 | 5.548 | 1 | -17.035 | 13.435 | 1.167 | 6.159 | 1 | -15.186 | 17.52 |
|  |  |  | 3 | -0.091 | 4.696 | 1 | -11.934 | 11.753 | -0.8 | 6.844 | 1 | -19.592 | 17.992 | 0.5 | 6.716 | 1 | -17.331 | 18.331 |
|  |  | 3 | 1 | -0.091 | 4.656 | 1 | -11.834 | 11.652 | -1 | 5.291 | 1 | -15.528 | 13.528 | 0.667 | 7.66 | 1 | -19.67 | 21.004 |
|  |  |  | 2 | 0.091 | 4.696 | 1 | -11.753 | 11.934 | 0.8 | 6.844 | 1 | -17.992 | 19.592 | -0.5 | 6.716 | 1 | -18.331 | 17.331 |
| HOMA_IR | 10mg | 1 | 2 | -0.032 | 0.326 | 1 | -0.854 | 0.79 | -1.282 | 0.589 | 0.145 | -2.898 | 0.335 | 0.437 | 0.37 | 0.76 | -0.544 | 1.419 |
|  |  |  | 3 | 0.075 | 0.383 | 1 | -0.89 | 1.039 | -0.86 | 0.823 | 0.947 | -3.121 | 1.402 | 0.425 | 0.366 | 0.787 | -0.548 | 1.398 |
|  |  | 2 | 1 | 0.032 | 0.326 | 1 | -0.79 | 0.854 | 1.282 | 0.589 | 0.145 | -0.335 | 2.898 | -0.437 | 0.37 | 0.76 | -1.419 | 0.544 |
|  |  |  | 3 | 0.106 | 0.29 | 1 | -0.625 | 0.837 | 0.422 | 0.59 | 1 | -1.197 | 2.042 | -0.012 | 0.296 | 1 | -0.798 | 0.774 |
|  |  | 3 | 1 | -0.075 | 0.383 | 1 | -1.039 | 0.89 | 0.86 | 0.823 | 0.947 | -1.402 | 3.121 | -0.425 | 0.366 | 0.787 | -1.398 | 0.548 |
|  |  |  | 2 | -0.106 | 0.29 | 1 | -0.837 | 0.625 | -0.422 | 0.59 | 1 | -2.042 | 1.197 | 0.012 | 0.296 | 1 | -0.774 | 0.798 |
|  | Placebo | 1 | 2 | -0.171 | 0.289 | 1 | -0.9 | 0.557 | -0.147 | 0.36 | 1 | -1.137 | 0.843 | -0.204 | 0.427 | 1 | -1.337 | 0.93 |
|  |  |  | 3 | 0.063 | 0.339 | 1 | -0.792 | 0.918 | 0.32 | 0.504 | 1 | -1.065 | 1.704 | -0.28 | 0.423 | 1 | -1.403 | 0.844 |
|  |  | 2 | 1 | 0.171 | 0.289 | 1 | -0.557 | 0.9 | 0.147 | 0.36 | 1 | -0.843 | 1.137 | 0.204 | 0.427 | 1 | -0.93 | 1.337 |
|  |  |  | 3 | 0.234 | 0.257 | 1 | -0.414 | 0.882 | 0.467 | 0.361 | 0.657 | -0.525 | 1.458 | -0.076 | 0.342 | 1 | -0.983 | 0.831 |
|  |  | 3 | 1 | -0.063 | 0.339 | 1 | -0.918 | 0.792 | -0.32 | 0.504 | 1 | -1.704 | 1.065 | 0.28 | 0.423 | 1 | -0.844 | 1.403 |
|  |  |  | 2 | -0.234 | 0.257 | 1 | -0.882 | 0.414 | -0.467 | 0.361 | 0.657 | -1.458 | 0.525 | 0.076 | 0.342 | 1 | -0.831 | 0.983 |
|  | 5mg | 1 | 2 | -0.427 | 0.326 | 0.598 | -1.249 | 0.395 | -0.425 | 0.456 | 1 | -1.677 | 0.827 | -0.428 | 0.427 | 0.989 | -1.562 | 0.705 |
|  |  |  | 3 | -0.579 | 0.383 | 0.419 | -1.544 | 0.386 | -1.204 | 0.638 | 0.245 | -2.955 | 0.548 | -0.058 | 0.423 | 1 | -1.181 | 1.066 |
|  |  | 2 | 1 | 0.427 | 0.326 | 0.598 | -0.395 | 1.249 | 0.425 | 0.456 | 1 | -0.827 | 1.677 | 0.428 | 0.427 | 0.989 | -0.705 | 1.562 |
|  |  |  | 3 | -0.152 | 0.29 | 1 | -0.883 | 0.579 | -0.779 | 0.457 | 0.336 | -2.033 | 0.476 | 0.37 | 0.342 | 0.881 | -0.537 | 1.278 |
|  |  | 3 | 1 | 0.579 | 0.383 | 0.419 | -0.386 | 1.544 | 1.204 | 0.638 | 0.245 | -0.548 | 2.955 | 0.058 | 0.423 | 1 | -1.066 | 1.181 |
|  |  |  | 2 | 0.152 | 0.29 | 1 | -0.579 | 0.883 | 0.779 | 0.457 | 0.336 | -0.476 | 2.033 | -0.37 | 0.342 | 0.881 | -1.278 | 0.537 |

| **Supplementary Table 6. Analysis of blood marker which change significantly over time** | | | | | | | | | | | | | | | | | | |
| --- | --- | --- | --- | --- | --- | --- | --- | --- | --- | --- | --- | --- | --- | --- | --- | --- | --- | --- |
|  |  |  |  |  |  |  |  |  |  |  |  |  |  |  |  |  |  |  |
| **Repeated Measures ANOVA** | | | | | | |  |  |  |  |  |  |  |  |  |  |  |  |
| All Genders |  |  |  |  |  |  | Females |  |  |  |  | Males |  |  |  |  |  |  |
| Group | Measure | df 1 | df 2 | F | p-value | Partial Eta Squared | df 1 | df 2 | F | p-value | Partial Eta Squared | df 1 | df 2 | F | p-value | Partial Eta Squared |  |  |
| 10mg | Calcium | 2 | 52 | 6.909 | 0.002 | 0.21 | 2 | 10 | 3.616 | 0.066 | 0.42 | 2 | 40 | 3.827 | 0.03 | 0.161 |  |  |
|  | Uric Acid | 2 | 52 | 0.978 | 0.383 | 0.036 | 2 | 10 | 1.051 | 0.385 | 0.174 | 2 | 40 | 0.519 | 0.599 | 0.025 |  |  |
|  | WBC^ | 1.333 | 34.663 | 0.795 | 0.413 | 0.03 | 2 | 10 | 0.682 | 0.528 | 0.12 | 2 | 40 | 0.809 | 0.452 | 0.039 |  |  |
|  | Hematocrit | 2 | 52 | 0.071 | 0.931 | 0.003 | 2 | 10 | 0.8 | 0.476 | 0.138 | 2 | 40 | 0.508 | 0.605 | 0.025 |  |  |
|  | Absolute Neutrophils^ | 1.269 | 32.984 | 0.885 | 0.378 | 0.033 | 2 | 10 | 0.744 | 0.5 | 0.13 | 2 | 40 | 0.276 | 0.76 | 0.014 |  |  |
|  | Absolute Lymphocytes | 2 | 52 | 3.326 | 0.044 | 0.113 | 2 | 10 | 1.976 | 0.189 | 0.283 | 2 | 40 | 1.904 | 0.162 | 0.087 |  |  |
|  | Absoloute Monocytes^ | 1.577 | 41.012 | 0.92 | 0.386 | 0.034 | 1.086 | 5.432 | 1.27 | 0.313 | 0.202 | 2 | 40 | 0.275 | 0.761 | 0.014 |  |  |
|  | Neutrophils | 2 | 52 | 1.276 | 0.288 | 0.047 | 2 | 10 | 1.077 | 0.377 | 0.177 | 2 | 40 | 0.466 | 0.631 | 0.023 |  |  |
|  | Lymphocytes | 2 | 52 | 2.169 | 0.124 | 0.077 | 2 | 10 | 1.188 | 0.344 | 0.192 | 2 | 40 | 1.131 | 0.333 | 0.054 |  |  |
|  | CO2 | 2 | 52 | 7.492 | 0.001 | 0.224 | 2 | 10 | 2.5 | 0.132 | 0.333 | 2 | 40 | 5.77 | 0.006 | 0.224 |  |  |
|  | eGFR | 2 | 52 | 0.163 | 0.85 | 0.006 | 2 | 10 | 0.41 | 0.674 | 0.076 | 2 | 40 | 0.519 | 0.599 | 0.025 |  |  |
|  | DHEA^ | 1.175 | 30.546 | 1.668 | 0.208 | 0.06 | 2 | 10 | 1.016 | 0.397 | 0.169 | 1.171 | 23.417 | 1.919 | 0.179 | 0.088 |  |  |
|  | HA1C | 2 | 66 | 3.359 | 0.042 | 0.107 | 2 | 14 | 0.791 | 0.473 | 0.102 | 2 | 40 | 2.515 | 0.094 | 0.112 |  |  |
|  |  |  |  |  |  |  |  |  |  |  |  |  |  |  |  |  |  |  |
| Placebo | Calcium | 2 | 56 | 4.185 | 0.02 | 0.13 | 2 | 20 | 1.708 | 0.207 | 0.146 | 2 | 34 | 2.526 | 0.095 | 0.129 |  |  |
|  | Uric Acid | 2 | 56 | 3.009 | 0.057 | 0.097 | 2 | 20 | 1.475 | 0.253 | 0.129 | 2 | 34 | 2.247 | 0.121 | 0.117 |  |  |
|  | WBC^ | 1.642 | 45.964 | 0.973 | 0.371 | 0.034 | 2 | 20 | 1.37 | 0.277 | 0.121 | 2 | 34 | 0.145 | 0.865 | 0.008 |  |  |
|  | Hematocrit | 2 | 56 | 2.74 | 0.073 | 0.089 | 2 | 20 | 1.917 | 0.173 | 0.161 | 2 | 34 | 2 | 0.151 | 0.105 |  |  |
|  | Absolute Neutrophils | 2 | 56 | 0.437 | 0.648 | 0.015 | 2 | 20 | 0.556 | 0.582 | 0.053 | 2 | 34 | 0.077 | 0.926 | 0.005 |  |  |
|  | Absolute Lymphocytes^ | 1.615 | 45.212 | 2.102 | 0.143 | 0.07 | 2 | 20 | 3.021 | 0.071 | 0.232 | 2 | 34 | 0.034 | 0.967 | 0.002 |  |  |
|  | Absoloute Monocytes^ | 1.339 | 37.496 | 0.374 | 0.606 | 0.013 | 2 | 20 | 1.281 | 0.3 | 0.114 | 2 | 34 | 0.192 | 0.827 | 0.011 |  |  |
|  | Neutrophils | 2 | 56 | 1.094 | 0.342 | 0.038 | 2 | 20 | 1.068 | 0.363 | 0.096 | 2 | 34 | 0.294 | 0.747 | 0.017 |  |  |
|  | Lymphocytes | 2 | 56 | 2.67 | 0.078 | 0.087 | 2 | 20 | 3.217 | 0.061 | 0.243 | 2 | 34 | 0.757 | 0.477 | 0.043 |  |  |
|  | CO2 | 2 | 56 | 0.69 | 0.506 | 0.024 | 2 | 20 | 0.59 | 0.564 | 0.056 | 2 | 34 | 0.204 | 0.816 | 0.012 |  |  |
|  | eGFR | 2 | 56 | 2.119 | 0.13 | 0.07 | 2 | 20 | 0.68 | 0.518 | 0.064 | 2 | 34 | 4.285 | 0.022 | 0.201 |  |  |
|  | DHEA^ | 1.516 | 42.456 | 0.654 | 0.484 | 0.023 | 2 | 20 | 0.141 | 0.869 | 0.014 | 2 | 34 | 0.708 | 0.5 | 0.04 |  |  |
|  | HA1C | 2 | 58 | 0.902 | 0.411 | 0.03 | 2 | 22 | 0.957 | 0.399 | 0.08 | 2 | 34 | 0.234 | 0.793 | 0.014 |  |  |
|  |  |  |  |  |  |  |  |  |  |  |  |  |  |  |  |  |  |  |
| 5mg | Calcium | 2 | 54 | 1.258 | 0.292 | 0.045 | 2 | 16 | 1.721 | 0.21 | 0.177 | 2 | 36 | 0.687 | 0.51 | 0.037 |  |  |
|  | Uric Acid^ | 1.653 | 44.618 | 2.365 | 0.115 | 0.081 | 2 | 16 | 2.687 | 0.099 | 0.251 | 2 | 36 | 0.965 | 0.391 | 0.051 |  |  |
|  | WBC | 2 | 54 | 0.47 | 0.628 | 0.017 | 2 | 16 | 1.459 | 0.262 | 0.154 | 2 | 36 | 1.177 | 0.32 | 0.061 |  |  |
|  | Hematocrit | 2 | 54 | 3.165 | 0.05 | 0.105 | 2 | 16 | 3.719 | 0.047 | 0.317 | 2 | 36 | 1.404 | 0.259 | 0.072 |  |  |
|  | Absolute Neutrophils | 2 | 54 | 0.641 | 0.531 | 0.023 | 2 | 16 | 1.22 | 0.321 | 0.132 | 2 | 36 | 1.774 | 0.184 | 0.09 |  |  |
|  | Absolute Lymphocytes | 2 | 54 | 0.534 | 0.589 | 0.019 | 2 | 16 | 0.079 | 0.924 | 0.01 | 2 | 36 | 0.714 | 0.497 | 0.038 |  |  |
|  | Absoloute Monocytes | 2 | 54 | 0.01 | 0.99 | 0 | 2 | 16 | 0.923 | 0.417 | 0.103 | 2 | 36 | 0.524 | 0.596 | 0.028 |  |  |
|  | Neutrophils | 2 | 54 | 0.844 | 0.436 | 0.03 | 2 | 16 | 1.168 | 0.336 | 0.127 | 2 | 36 | 1.674 | 0.202 | 0.085 |  |  |
|  | Lymphocytes | 2 | 54 | 0.854 | 0.432 | 0.031 | 2 | 16 | 0.641 | 0.54 | 0.074 | 2 | 36 | 1.767 | 0.185 | 0.089 |  |  |
|  | CO2 | 2 | 54 | 3.891 | 0.026 | 0.126 | 2 | 16 | 7.921 | 0.004 | 0.498 | 2 | 36 | 0.82 | 0.449 | 0.044 |  |  |
|  | eGFR | 2 | 54 | 0.422 | 0.658 | 0.015 | 2 | 16 | 0.542 | 0.592 | 0.063 | 2 | 36 | 0.851 | 0.435 | 0.045 |  |  |
|  | DHEA | 2 | 54 | 2.982 | 0.059 | 0.099 | 2 | 16 | 3.446 | 0.057 | 0.301 | 2 | 36 | 1.974 | 0.154 | 0.099 |  |  |
|  | HA1C | 2 | 66 | 5.63 | 0.006 | 0.146 | 2 | 24 | 1.099 | 0.349 | 0.084 | 2 | 40 | 4.578 | 0.016 | 0.186 |  |  |
|  |  |  |  |  |  |  |  |  |  |  |  |  |  |  |  |  |  |  |
|  |  |  |  |  |  |  |  |  |  |  |  |  |  |  |  |  |  |  |
| **Pairwise Comparisons** | | | |  |  |  |  |  |  |  |  |  |  |  |  |  |  |  |
|  |  |  |  | All Genders | | | | | Females | | | | | Males | | | | |
| Group | Measure | Time 1 | Time 2 | Mean Difference | Std. Error | p-value | 95% Confidence Interval for Difference |  | Mean Difference | Std. Error | p value | 95% Confidence Interval for Difference |  | Mean Difference | Std. Error | p-value | 95% Confidence Interval for Difference |  |
|  |  |  |  |  |  |  | Lower Bound | Upper Bound |  |  |  | Lower Bound | Upper Bound |  |  |  | Lower Bound | Upper Bound |
| 10mg | Calcium | 1 | 2 | 0.096 | 0.061 | 0.387 | -0.061 | 0.254 | 0.2 | 0.155 | 0.76 | -0.348 | 0.748 | 0.067 | 0.066 | 0.98 | -0.107 | 0.24 |
|  |  |  | 3 | .200* | 0.052 | 0.002 | 0.068 | 0.332 | 0.317 | 0.114 | 0.116 | -0.085 | 0.719 | .167* | 0.057 | 0.027 | 0.017 | 0.317 |
|  |  | 2 | 1 | -0.096 | 0.061 | 0.387 | -0.254 | 0.061 | -0.2 | 0.155 | 0.76 | -0.748 | 0.348 | -0.067 | 0.066 | 0.98 | -0.24 | 0.107 |
|  |  |  | 3 | 0.104 | 0.047 | 0.112 | -0.017 | 0.225 | 0.117 | 0.075 | 0.54 | -0.148 | 0.381 | 0.1 | 0.058 | 0.296 | -0.051 | 0.251 |
|  |  | 3 | 1 | -.200* | 0.052 | 0.002 | -0.332 | -0.068 | -0.317 | 0.114 | 0.116 | -0.719 | 0.085 | -.167* | 0.057 | 0.027 | -0.317 | -0.017 |
|  |  |  | 2 | -0.104 | 0.047 | 0.112 | -0.225 | 0.017 | -0.117 | 0.075 | 0.54 | -0.381 | 0.148 | -0.1 | 0.058 | 0.296 | -0.251 | 0.051 |
|  | Uric Acid | 1 | 2 | 0.115 | 0.171 | 1 | -0.323 | 0.552 | 0.017 | 0.322 | 1 | -1.121 | 1.154 | 0.143 | 0.203 | 1 | -0.388 | 0.674 |
|  |  |  | 3 | 0.219 | 0.125 | 0.276 | -0.101 | 0.538 | 0.35 | 0.312 | 0.938 | -0.752 | 1.452 | 0.181 | 0.137 | 0.607 | -0.178 | 0.54 |
|  |  | 2 | 1 | -0.115 | 0.171 | 1 | -0.552 | 0.323 | -0.017 | 0.322 | 1 | -1.154 | 1.121 | -0.143 | 0.203 | 1 | -0.674 | 0.388 |
|  |  |  | 3 | 0.104 | 0.169 | 1 | -0.328 | 0.536 | 0.333 | 0.148 | 0.22 | -0.188 | 0.855 | 0.038 | 0.212 | 1 | -0.517 | 0.593 |
|  |  | 3 | 1 | -0.219 | 0.125 | 0.276 | -0.538 | 0.101 | -0.35 | 0.312 | 0.938 | -1.452 | 0.752 | -0.181 | 0.137 | 0.607 | -0.54 | 0.178 |
|  |  |  | 2 | -0.104 | 0.169 | 1 | -0.536 | 0.328 | -0.333 | 0.148 | 0.22 | -0.855 | 0.188 | -0.038 | 0.212 | 1 | -0.593 | 0.517 |
|  | WBC | 1 | 2 | -0.126 | 0.262 | 1 | -0.795 | 0.544 | -0.933 | 1.035 | 1 | -4.592 | 2.725 | 0.105 | 0.161 | 1 | -0.316 | 0.525 |
|  |  |  | 3 | 0.185 | 0.145 | 0.636 | -0.185 | 0.555 | 0.133 | 0.486 | 1 | -1.585 | 1.852 | 0.2 | 0.134 | 0.456 | -0.151 | 0.551 |
|  |  | 2 | 1 | 0.126 | 0.262 | 1 | -0.544 | 0.795 | 0.933 | 1.035 | 1 | -2.725 | 4.592 | -0.105 | 0.161 | 1 | -0.525 | 0.316 |
|  |  |  | 3 | 0.311 | 0.309 | 0.97 | -0.48 | 1.102 | 1.067 | 1.29 | 1 | -3.492 | 5.625 | 0.095 | 0.174 | 1 | -0.359 | 0.55 |
|  |  | 3 | 1 | -0.185 | 0.145 | 0.636 | -0.555 | 0.185 | -0.133 | 0.486 | 1 | -1.852 | 1.585 | -0.2 | 0.134 | 0.456 | -0.551 | 0.151 |
|  |  |  | 2 | -0.311 | 0.309 | 0.97 | -1.102 | 0.48 | -1.067 | 1.29 | 1 | -5.625 | 3.492 | -0.095 | 0.174 | 1 | -0.55 | 0.359 |
|  | Hematocrit | 1 | 2 | -0.011 | 0.386 | 1 | -1 | 0.978 | -0.167 | 0.858 | 1 | -3.198 | 2.865 | 0.033 | 0.443 | 1 | -1.125 | 1.192 |
|  |  |  | 3 | 0.137 | 0.495 | 1 | -1.131 | 1.405 | -0.983 | 0.995 | 1 | -4.499 | 2.532 | 0.457 | 0.563 | 1 | -1.013 | 1.927 |
|  |  | 2 | 1 | 0.011 | 0.386 | 1 | -0.978 | 1 | 0.167 | 0.858 | 1 | -2.865 | 3.198 | -0.033 | 0.443 | 1 | -1.192 | 1.125 |
|  |  |  | 3 | 0.148 | 0.421 | 1 | -0.929 | 1.225 | -0.817 | 0.594 | 0.683 | -2.916 | 1.283 | 0.424 | 0.504 | 1 | -0.892 | 1.74 |
|  |  | 3 | 1 | -0.137 | 0.495 | 1 | -1.405 | 1.131 | 0.983 | 0.995 | 1 | -2.532 | 4.499 | -0.457 | 0.563 | 1 | -1.927 | 1.013 |
|  |  |  | 2 | -0.148 | 0.421 | 1 | -1.225 | 0.929 | 0.817 | 0.594 | 0.683 | -1.283 | 2.916 | -0.424 | 0.504 | 1 | -1.74 | 0.892 |
|  | Absolute Neutrophils | 1 | 2 | -250.593 | 250.562 | 0.979 | -891.766 | 390.581 | -1060.167 | 991.269 | 1 | -4563.423 | 2443.089 | -19.286 | 150.697 | 1 | -412.994 | 374.423 |
|  |  |  | 3 | 39.222 | 127.056 | 1 | -285.907 | 364.352 | -115.833 | 455.021 | 1 | -1723.929 | 1492.262 | 83.524 | 108.406 | 1 | -199.696 | 366.744 |
|  |  | 2 | 1 | 250.593 | 250.562 | 0.979 | -390.581 | 891.766 | 1060.167 | 991.269 | 1 | -2443.089 | 4563.423 | 19.286 | 150.697 | 1 | -374.423 | 412.994 |
|  |  |  | 3 | 289.815 | 297.629 | 1 | -471.801 | 1051.431 | 944.333 | 1239.726 | 1 | -3436.995 | 5325.662 | 102.81 | 174.39 | 1 | -352.799 | 558.418 |
|  |  | 3 | 1 | -39.222 | 127.056 | 1 | -364.352 | 285.907 | 115.833 | 455.021 | 1 | -1492.262 | 1723.929 | -83.524 | 108.406 | 1 | -366.744 | 199.696 |
|  |  |  | 2 | -289.815 | 297.629 | 1 | -1051.431 | 471.801 | -944.333 | 1239.726 | 1 | -5325.662 | 3436.995 | -102.81 | 174.39 | 1 | -558.418 | 352.799 |
|  | Absolute Lymphocytes | 1 | 2 | 144.259 | 69.577 | 0.145 | -33.785 | 322.303 | 177 | 149.197 | 0.866 | -350.28 | 704.28 | 134.905 | 80.509 | 0.328 | -75.431 | 345.241 |
|  |  |  | 3 | 130.185 | 58.145 | 0.102 | -18.604 | 278.975 | 239.5 | 107.326 | 0.228 | -139.801 | 618.801 | 98.952 | 67.886 | 0.481 | -78.407 | 276.311 |
|  |  | 2 | 1 | -144.259 | 69.577 | 0.145 | -322.303 | 33.785 | -177 | 149.197 | 0.866 | -704.28 | 350.28 | -134.905 | 80.509 | 0.328 | -345.241 | 75.431 |
|  |  |  | 3 | -14.074 | 56.496 | 1 | -158.643 | 130.495 | 62.5 | 114.368 | 1 | -341.688 | 466.688 | -35.952 | 65.52 | 1 | -207.128 | 135.223 |
|  |  | 3 | 1 | -130.185 | 58.145 | 0.102 | -278.975 | 18.604 | -239.5 | 107.326 | 0.228 | -618.801 | 139.801 | -98.952 | 67.886 | 0.481 | -276.311 | 78.407 |
|  |  |  | 2 | 14.074 | 56.496 | 1 | -130.495 | 158.643 | -62.5 | 114.368 | 1 | -466.688 | 341.688 | 35.952 | 65.52 | 1 | -135.223 | 207.128 |
|  | Abs Monocytes | 1 | 2 | -14.074 | 25.956 | 1 | -80.493 | 52.345 | -67.333 | 64.876 | 1 | -296.613 | 161.946 | 1.143 | 27.808 | 1 | -71.509 | 73.795 |
|  |  |  | 3 | 19.556 | 17.869 | 0.851 | -26.17 | 65.281 | 24.833 | 21.829 | 0.92 | -52.312 | 101.979 | 18.048 | 22.341 | 1 | -40.321 | 76.416 |
|  |  | 2 | 1 | 14.074 | 25.956 | 1 | -52.345 | 80.493 | 67.333 | 64.876 | 1 | -161.946 | 296.613 | -1.143 | 27.808 | 1 | -73.795 | 71.509 |
|  |  |  | 3 | 33.63 | 29.453 | 0.792 | -41.74 | 108.999 | 92.167 | 77.865 | 0.869 | -183.017 | 367.35 | 16.905 | 30.886 | 1 | -63.788 | 97.597 |
|  |  | 3 | 1 | -19.556 | 17.869 | 0.851 | -65.281 | 26.17 | -24.833 | 21.829 | 0.92 | -101.979 | 52.312 | -18.048 | 22.341 | 1 | -76.416 | 40.321 |
|  |  |  | 2 | -33.63 | 29.453 | 0.792 | -108.999 | 41.74 | -92.167 | 77.865 | 0.869 | -367.35 | 183.017 | -16.905 | 30.886 | 1 | -97.597 | 63.788 |
|  | Neutrophils | 1 | 2 | -2.485 | 1.433 | 0.284 | -6.152 | 1.182 | -6.033 | 3.266 | 0.372 | -17.577 | 5.511 | -1.471 | 1.562 | 1 | -5.551 | 2.608 |
|  |  |  | 3 | -1.041 | 1.412 | 1 | -4.655 | 2.574 | -3.917 | 3.636 | 0.992 | -16.768 | 8.935 | -0.219 | 1.496 | 1 | -4.127 | 3.689 |
|  |  | 2 | 1 | 2.485 | 1.433 | 0.284 | -1.182 | 6.152 | 6.033 | 3.266 | 0.372 | -5.511 | 17.577 | 1.471 | 1.562 | 1 | -2.608 | 5.551 |
|  |  |  | 3 | 1.444 | 1.81 | 1 | -3.186 | 6.075 | 2.117 | 5.322 | 1 | -16.693 | 20.927 | 1.252 | 1.855 | 1 | -3.594 | 6.099 |
|  |  | 3 | 1 | 1.041 | 1.412 | 1 | -2.574 | 4.655 | 3.917 | 3.636 | 0.992 | -8.935 | 16.768 | 0.219 | 1.496 | 1 | -3.689 | 4.127 |
|  |  |  | 2 | -1.444 | 1.81 | 1 | -6.075 | 3.186 | -2.117 | 5.322 | 1 | -20.927 | 16.693 | -1.252 | 1.855 | 1 | -6.099 | 3.594 |
|  | Lymphocytes | 1 | 2 | 2.896 | 1.43 | 0.16 | -0.763 | 6.555 | 5.383 | 3.05 | 0.413 | -5.395 | 16.161 | 2.186 | 1.625 | 0.581 | -2.059 | 6.43 |
|  |  |  | 3 | 1.259 | 1.164 | 0.867 | -1.719 | 4.237 | 3.65 | 2.845 | 0.767 | -6.405 | 13.705 | 0.576 | 1.257 | 1 | -2.708 | 3.86 |
|  |  | 2 | 1 | -2.896 | 1.43 | 0.16 | -6.555 | 0.763 | -5.383 | 3.05 | 0.413 | -16.161 | 5.395 | -2.186 | 1.625 | 0.581 | -6.43 | 2.059 |
|  |  |  | 3 | -1.637 | 1.56 | 0.911 | -5.63 | 2.356 | -1.733 | 4.553 | 1 | -17.823 | 14.357 | -1.61 | 1.609 | 0.987 | -5.813 | 2.594 |
|  |  | 3 | 1 | -1.259 | 1.164 | 0.867 | -4.237 | 1.719 | -3.65 | 2.845 | 0.767 | -13.705 | 6.405 | -0.576 | 1.257 | 1 | -3.86 | 2.708 |
|  |  |  | 2 | 1.637 | 1.56 | 0.911 | -2.356 | 5.63 | 1.733 | 4.553 | 1 | -14.357 | 17.823 | 1.61 | 1.609 | 0.987 | -2.594 | 5.813 |
|  | CO2 | 1 | 2 | 1.185* | 0.4 | 0.019 | 0.163 | 2.208 | 0.667 | 0.422 | 0.524 | -0.823 | 2.157 | 1.333* | 0.499 | 0.044 | 0.029 | 2.638 |
|  |  |  | 3 | 1.222* | 0.347 | 0.005 | 0.334 | 2.111 | 1 | 0.447 | 0.227 | -0.581 | 2.581 | 1.286* | 0.432 | 0.022 | 0.158 | 2.414 |
|  |  | 2 | 1 | -1.185* | 0.4 | 0.019 | -2.208 | -0.163 | -0.667 | 0.422 | 0.524 | -2.157 | 0.823 | -1.333* | 0.499 | 0.044 | -2.638 | -0.029 |
|  |  |  | 3 | 0.037 | 0.327 | 1 | -0.799 | 0.873 | 0.333 | 0.494 | 1 | -1.414 | 2.081 | -0.048 | 0.399 | 1 | -1.091 | 0.995 |
|  |  | 3 | 1 | -1.222* | 0.347 | 0.005 | -2.111 | -0.334 | -1 | 0.447 | 0.227 | -2.581 | 0.581 | -1.286* | 0.432 | 0.022 | -2.414 | -0.158 |
|  |  |  | 2 | -0.037 | 0.327 | 1 | -0.873 | 0.799 | -0.333 | 0.494 | 1 | -2.081 | 1.414 | 0.048 | 0.399 | 1 | -0.995 | 1.091 |
|  | eGFR | 1 | 2 | -0.63 | 1.329 | 1 | -4.031 | 2.772 | 2.5 | 2.778 | 1 | -7.317 | 12.317 | -1.524 | 1.491 | 0.957 | -5.418 | 2.371 |
|  |  |  | 3 | -0.704 | 1.506 | 1 | -4.557 | 3.149 | 0.167 | 3.97 | 1 | -13.864 | 14.197 | -0.952 | 1.628 | 1 | -5.205 | 3.301 |
|  |  | 2 | 1 | 0.63 | 1.329 | 1 | -2.772 | 4.031 | -2.5 | 2.778 | 1 | -12.317 | 7.317 | 1.524 | 1.491 | 0.957 | -2.371 | 5.418 |
|  |  |  | 3 | -0.074 | 1.206 | 1 | -3.161 | 3.013 | -2.333 | 2.261 | 1 | -10.323 | 5.657 | 0.571 | 1.405 | 1 | -3.099 | 4.242 |
|  |  | 3 | 1 | 0.704 | 1.506 | 1 | -3.149 | 4.557 | -0.167 | 3.97 | 1 | -14.197 | 13.864 | 0.952 | 1.628 | 1 | -3.301 | 5.205 |
|  |  |  | 2 | 0.074 | 1.206 | 1 | -3.013 | 3.161 | 2.333 | 2.261 | 1 | -5.657 | 10.323 | -0.571 | 1.405 | 1 | -4.242 | 3.099 |
|  | DHEA | 1 | 2 | -10.148 | 6.598 | 0.408 | -27.033 | 6.737 | -2.167 | 6.316 | 1 | -24.489 | 20.156 | -12.429 | 8.292 | 0.449 | -34.091 | 9.234 |
|  |  |  | 3 | 16.296 | 15.81 | 0.936 | -24.16 | 56.753 | -8.333 | 7.013 | 0.864 | -33.117 | 16.45 | 23.333 | 20.078 | 0.777 | -29.122 | 75.789 |
|  |  | 2 | 1 | 10.148 | 6.598 | 0.408 | -6.737 | 27.033 | 2.167 | 6.316 | 1 | -20.156 | 24.489 | 12.429 | 8.292 | 0.449 | -9.234 | 34.091 |
|  |  |  | 3 | 26.444 | 18.624 | 0.503 | -21.213 | 74.102 | -6.167 | 4.622 | 0.719 | -22.501 | 10.167 | 35.762 | 23.636 | 0.438 | -25.99 | 97.514 |
|  |  | 3 | 1 | -16.296 | 15.81 | 0.936 | -56.753 | 24.16 | 8.333 | 7.013 | 0.864 | -16.45 | 33.117 | -23.333 | 20.078 | 0.777 | -75.789 | 29.122 |
|  |  |  | 2 | -26.444 | 18.624 | 0.503 | -74.102 | 21.213 | 6.167 | 4.622 | 0.719 | -10.167 | 22.501 | -35.762 | 23.636 | 0.438 | -97.514 | 25.99 |
|  | H1AC | 1 | 2 | -0.007 | 0.024 | 1 | -0.067 | 0.054 | -0.012 | 0.044 | 1 | -0.15 | 0.125 | -0.005 | 0.029 | 1 | -0.08 | 0.071 |
|  |  |  | 3 | -0.062 | 0.027 | 0.086 | -0.131 | 0.006 | -0.05 | 0.038 | 0.682 | -0.168 | 0.068 | -0.067 | 0.035 | 0.208 | -0.157 | 0.024 |
|  |  | 2 | 1 | 0.007 | 0.024 | 1 | -0.054 | 0.067 | 0.012 | 0.044 | 1 | -0.125 | 0.15 | 0.005 | 0.029 | 1 | -0.071 | 0.08 |
|  |  |  | 3 | -0.055 | 0.028 | 0.174 | -0.126 | 0.016 | -0.037 | 0.042 | 1 | -0.169 | 0.094 | -0.062 | 0.036 | 0.29 | -0.155 | 0.031 |
|  |  | 3 | 1 | 0.062 | 0.027 | 0.086 | -0.006 | 0.131 | 0.05 | 0.038 | 0.682 | -0.068 | 0.168 | 0.067 | 0.035 | 0.208 | -0.024 | 0.157 |
|  |  |  | 2 | 0.055 | 0.028 | 0.174 | -0.016 | 0.126 | 0.037 | 0.042 | 1 | -0.094 | 0.169 | 0.062 | 0.036 | 0.29 | -0.031 | 0.155 |
|  |  |  |  |  |  |  |  |  |  |  |  |  |  |  |  |  |  |  |
| Placebo | Calcium | 1 | 2 | .186* | 0.052 | 0.004 | 0.054 | 0.319 | 0.191 | 0.076 | 0.09 | -0.026 | 0.408 | 0.183 | 0.072 | 0.06 | -0.007 | 0.373 |
|  |  |  | 3 | 0.1 | 0.065 | 0.397 | -0.064 | 0.264 | 0.145 | 0.115 | 0.709 | -1.86E-01 | 0.477 | 0.072 | 0.078 | 1 | -0.136 | 0.28 |
|  |  | 2 | 1 | -.186* | 0.052 | 0.004 | -0.319 | -0.054 | -0.191 | 0.076 | 0.09 | -0.408 | 0.026 | -0.183 | 0.072 | 0.06 | -0.373 | 0.007 |
|  |  |  | 3 | -0.086 | 0.075 | 0.775 | -0.277 | 0.104 | -0.045 | 0.126 | 1 | -4.07E-01 | 0.316 | -0.111 | 0.095 | 0.774 | -0.363 | 0.141 |
|  |  | 3 | 1 | -0.1 | 0.065 | 0.397 | -0.264 | 0.064 | -0.145 | 0.115 | 0.709 | -0.477 | 0.186 | -0.072 | 0.078 | 1 | -0.28 | 0.136 |
|  |  |  | 2 | 0.086 | 0.075 | 0.775 | -0.104 | 0.277 | 0.045 | 0.126 | 1 | -0.316 | 0.407 | 0.111 | 0.095 | 0.774 | -0.141 | 0.363 |
|  | Uric Acid | 1 | 2 | 0.266 | 0.105 | 0.053 | -0.003 | 0.534 | 0.164 | 0.102 | 0.42 | -0.129 | 0.457 | 0.328 | 0.159 | 0.163 | -0.093 | 0.749 |
|  |  |  | 3 | 0.107 | 0.088 | 0.711 | -0.118 | 0.332 | -0.055 | 0.11 | 1 | -0.37 | 0.261 | 0.206 | 0.122 | 0.332 | -0.119 | 0.53 |
|  |  | 2 | 1 | -0.266 | 0.105 | 0.053 | -0.534 | 0.003 | -0.164 | 0.102 | 0.42 | -0.457 | 0.129 | -0.328 | 0.159 | 0.163 | -0.749 | 0.093 |
|  |  |  | 3 | -0.159 | 0.129 | 0.687 | -0.487 | 0.17 | -0.218 | 0.173 | 0.708 | -0.715 | 0.279 | -0.122 | 0.182 | 1 | -0.606 | 0.361 |
|  |  | 3 | 1 | -0.107 | 0.088 | 0.711 | -0.332 | 0.118 | 0.055 | 0.11 | 1 | -0.261 | 0.37 | -0.206 | 0.122 | 0.332 | -0.53 | 0.119 |
|  |  |  | 2 | 0.159 | 0.129 | 0.687 | -0.17 | 0.487 | 0.218 | 0.173 | 0.708 | -0.279 | 0.715 | 0.122 | 0.182 | 1 | -0.361 | 0.606 |
|  | WBC | 1 | 2 | -0.269 | 0.229 | 0.752 | -0.853 | 0.315 | -0.591 | 0.54 | 0.9 | -2.142 | 0.96 | -0.072 | 0.169 | 1 | -0.521 | 0.376 |
|  |  |  | 3 | -0.031 | 0.154 | 1 | -0.424 | 0.362 | 0.091 | 0.236 | 1 | -0.585 | 0.767 | -0.106 | 0.206 | 1 | -0.653 | 0.442 |
|  |  | 2 | 1 | 0.269 | 0.229 | 0.752 | -0.315 | 0.853 | 0.591 | 0.54 | 0.9 | -0.96 | 2.142 | 0.072 | 0.169 | 1 | -0.376 | 0.521 |
|  |  |  | 3 | 0.238 | 0.239 | 0.983 | -0.37 | 0.846 | 0.682 | 0.503 | 0.614 | -0.76 | 2.124 | -0.033 | 0.222 | 1 | -0.622 | 0.556 |
|  |  | 3 | 1 | 0.031 | 0.154 | 1 | -0.362 | 0.424 | -0.091 | 0.236 | 1 | -0.767 | 0.585 | 0.106 | 0.206 | 1 | -0.442 | 0.653 |
|  |  |  | 2 | -0.238 | 0.239 | 0.983 | -0.846 | 0.37 | -0.682 | 0.503 | 0.614 | -2.124 | 0.76 | 0.033 | 0.222 | 1 | -0.556 | 0.622 |
|  | Hematocrit | 1 | 2 | 0.848 | 0.528 | 0.357 | -0.495 | 2.192 | 0.155 | 0.613 | 1 | -1.604 | 1.913 | 1.272 | 0.759 | 0.336 | -0.743 | 3.287 |
|  |  |  | 3 | -0.283 | 0.449 | 1 | -1.426 | 0.86 | -1.155 | 0.78 | 0.509 | -3.394 | 1.085 | 0.25 | 0.521 | 1 | -1.134 | 1.634 |
|  |  | 2 | 1 | -0.848 | 0.528 | 0.357 | -2.192 | 0.495 | -0.155 | 0.613 | 1 | -1.913 | 1.604 | -1.272 | 0.759 | 0.336 | -3.287 | 0.743 |
|  |  |  | 3 | -1.131 | 0.528 | 0.123 | -2.475 | 0.213 | -1.309 | 0.786 | 0.38 | -3.564 | 0.946 | -1.022 | 0.718 | 0.518 | -2.929 | 0.884 |
|  |  | 3 | 1 | 0.283 | 0.449 | 1 | -0.86 | 1.426 | 1.155 | 0.78 | 0.509 | -1.085 | 3.394 | -0.25 | 0.521 | 1 | -1.634 | 1.134 |
|  |  |  | 2 | 1.131 | 0.528 | 0.123 | -0.213 | 2.475 | 1.309 | 0.786 | 0.38 | -0.946 | 3.564 | 1.022 | 0.718 | 0.518 | -0.884 | 2.929 |
|  | Absolute Neutrophils | 1 | 2 | -138.103 | 148.921 | 1 | -517.327 | 241.12 | -309.455 | 327.154 | 1 | -1248.411 | 629.502 | -33.389 | 136.657 | 1 | -396.212 | 329.434 |
|  |  |  | 3 | -98.103 | 129.933 | 1 | -428.973 | 232.766 | -148.636 | 219.9 | 1 | -779.766 | 482.493 | -67.222 | 165.132 | 1 | -505.648 | 371.204 |
|  |  | 2 | 1 | 138.103 | 148.921 | 1 | -241.12 | 517.327 | 309.455 | 327.154 | 1 | -629.502 | 1248.411 | 33.389 | 136.657 | 1 | -329.434 | 396.212 |
|  |  |  | 3 | 40 | 173.765 | 1 | -402.486 | 482.486 | 160.818 | 320.959 | 1 | -760.357 | 1081.994 | -33.833 | 205.539 | 1 | -579.539 | 511.872 |
|  |  | 3 | 1 | 98.103 | 129.933 | 1 | -232.766 | 428.973 | 148.636 | 219.9 | 1 | -482.493 | 779.766 | 67.222 | 165.132 | 1 | -371.204 | 505.648 |
|  |  |  | 2 | -40 | 173.765 | 1 | -482.486 | 402.486 | -160.818 | 320.959 | 1 | -1081.994 | 760.357 | 33.833 | 205.539 | 1 | -511.872 | 579.539 |
|  | Absolute Lymphocytes | 1 | 2 | -113.862 | 109.442 | 0.921 | -392.552 | 164.828 | -267.364 | 244.292 | 0.898 | -968.5 | 433.772 | -20.056 | 94.687 | 1 | -271.45 | 231.339 |
|  |  |  | 3 | 72.517 | 69.789 | 0.923 | -105.198 | 250.233 | 193.818 | 82.44 | 0.122 | -42.79 | 430.426 | -1.611 | 98.128 | 1 | -262.141 | 258.919 |
|  |  | 2 | 1 | 113.862 | 109.442 | 0.921 | -164.828 | 392.552 | 267.364 | 244.292 | 0.898 | -433.772 | 968.5 | 20.056 | 94.687 | 1 | -231.339 | 271.45 |
|  |  |  | 3 | 186.379 | 91.396 | 0.153 | -46.356 | 419.115 | 461.182 | 200.049 | 0.132 | -112.974 | 1035.338 | 18.444 | 58.362 | 1 | -136.507 | 173.396 |
|  |  | 3 | 1 | -72.517 | 69.789 | 0.923 | -250.233 | 105.198 | -193.818 | 82.44 | 0.122 | -430.426 | 42.79 | 1.611 | 98.128 | 1 | -258.919 | 262.141 |
|  |  |  | 2 | -186.379 | 91.396 | 0.153 | -419.115 | 46.356 | -461.182 | 200.049 | 0.132 | -1035.338 | 112.974 | -18.444 | 58.362 | 1 | -173.396 | 136.507 |
|  | Abs Monocytes | 1 | 2 | -7.241 | 21.965 | 1 | -63.175 | 48.692 | -35.545 | 47.27 | 1 | -171.213 | 100.122 | 10.056 | 20.753 | 1 | -45.042 | 65.154 |
|  |  |  | 3 | 12.379 | 15.179 | 1 | -26.273 | 51.032 | 38.818 | 26.523 | 0.522 | -37.306 | 114.942 | -3.778 | 17.833 | 1 | -51.123 | 43.568 |
|  |  | 2 | 1 | 7.241 | 21.965 | 1 | -48.692 | 63.175 | 35.545 | 47.27 | 1 | -100.122 | 171.213 | -10.056 | 20.753 | 1 | -65.154 | 45.042 |
|  |  |  | 3 | 19.621 | 29.434 | 1 | -55.333 | 94.574 | 74.364 | 59.505 | 0.72 | -96.419 | 245.146 | -13.833 | 29.2 | 1 | -91.358 | 63.691 |
|  |  | 3 | 1 | -12.379 | 15.179 | 1 | -51.032 | 26.273 | -38.818 | 26.523 | 0.522 | -114.942 | 37.306 | 3.778 | 17.833 | 1 | -43.568 | 51.123 |
|  |  |  | 2 | -19.621 | 29.434 | 1 | -94.574 | 55.333 | -74.364 | 59.505 | 0.72 | -245.146 | 96.419 | 13.833 | 29.2 | 1 | -63.691 | 91.358 |
|  | Neutrophils | 1 | 2 | -0.479 | 1.207 | 1 | -3.553 | 2.594 | -1.055 | 2.062 | 1 | -6.974 | 4.865 | -0.128 | 1.523 | 1 | -4.17 | 3.915 |
|  |  |  | 3 | -1.769 | 1.145 | 0.401 | -4.685 | 1.147 | -2.782 | 1.925 | 0.537 | -8.307 | 2.744 | -1.15 | 1.445 | 1 | -4.986 | 2.686 |
|  |  | 2 | 1 | 0.479 | 1.207 | 1 | -2.594 | 3.553 | 1.055 | 2.062 | 1 | -4.865 | 6.974 | 0.128 | 1.523 | 1 | -3.915 | 4.17 |
|  |  |  | 3 | -1.29 | 1.35 | 1 | -4.727 | 2.147 | -1.727 | 1.767 | 1 | -6.798 | 3.344 | -1.022 | 1.924 | 1 | -6.13 | 4.086 |
|  |  | 3 | 1 | 1.769 | 1.145 | 0.401 | -1.147 | 4.685 | 2.782 | 1.925 | 0.537 | -2.744 | 8.307 | 1.15 | 1.445 | 1 | -2.686 | 4.986 |
|  |  |  | 2 | 1.29 | 1.35 | 1 | -2.147 | 4.727 | 1.727 | 1.767 | 1 | -3.344 | 6.798 | 1.022 | 1.924 | 1 | -4.086 | 6.13 |
|  | Lymphocytes | 1 | 2 | 0.107 | 1.063 | 1 | -2.601 | 2.814 | -0.227 | 1.614 | 1 | -4.859 | 4.404 | 0.311 | 1.434 | 1 | -3.497 | 4.119 |
|  |  |  | 3 | 2.062 | 0.947 | 0.114 | -0.349 | 4.473 | 2.727 | 1.228 | 0.152 | -0.796 | 6.251 | 1.656 | 1.346 | 0.706 | -1.918 | 5.229 |
|  |  | 2 | 1 | -0.107 | 1.063 | 1 | -2.814 | 2.601 | 0.227 | 1.614 | 1 | -4.404 | 4.859 | -0.311 | 1.434 | 1 | -4.119 | 3.497 |
|  |  |  | 3 | 1.955 | 1.001 | 0.182 | -0.593 | 4.503 | 2.955* | 0.964 | 0.036 | 0.186 | 5.723 | 1.344 | 1.505 | 1 | -2.651 | 5.34 |
|  |  | 3 | 1 | -2.062 | 0.947 | 0.114 | -4.473 | 0.349 | -2.727 | 1.228 | 0.152 | -6.251 | 0.796 | -1.656 | 1.346 | 0.706 | -5.229 | 1.918 |
|  |  |  | 2 | -1.955 | 1.001 | 0.182 | -4.503 | 0.593 | -2.955* | 0.964 | 0.036 | -5.723 | -0.186 | -1.344 | 1.505 | 1 | -5.34 | 2.651 |
|  | CO2 | 1 | 2 | -0.034 | 0.417 | 1 | -1.095 | 1.027 | 0 | 0.661 | 1 | -1.896 | 1.896 | -0.056 | 0.551 | 1 | -1.519 | 1.408 |
|  |  |  | 3 | 0.414 | 0.383 | 0.867 | -0.561 | 1.389 | 0.636 | 0.527 | 0.765 | -0.876 | 2.149 | 0.278 | 0.535 | 1 | -1.143 | 1.698 |
|  |  | 2 | 1 | 0.034 | 0.417 | 1 | -1.027 | 1.095 | 0 | 0.661 | 1 | -1.896 | 1.896 | 0.056 | 0.551 | 1 | -1.408 | 1.519 |
|  |  |  | 3 | 0.448 | 0.47 | 1 | -0.748 | 1.644 | 0.636 | 0.812 | 1 | -1.694 | 2.967 | 0.333 | 0.589 | 1 | -1.229 | 1.896 |
|  |  | 3 | 1 | -0.414 | 0.383 | 0.867 | -1.389 | 0.561 | -0.636 | 0.527 | 0.765 | -2.149 | 0.876 | -0.278 | 0.535 | 1 | -1.698 | 1.143 |
|  |  |  | 2 | -0.448 | 0.47 | 1 | -1.644 | 0.748 | -0.636 | 0.812 | 1 | -2.967 | 1.694 | -0.333 | 0.589 | 1 | -1.896 | 1.229 |
|  | eGFR | 1 | 2 | -2.862 | 1.526 | 0.214 | -6.749 | 1.024 | 1.455 | 1.979 | 1 | -4.225 | 7.134 | -5.500* | 1.922 | 0.032 | -10.603 | -0.397 |
|  |  |  | 3 | -1.793 | 1.349 | 0.584 | -5.23 | 1.643 | -0.545 | 1.123 | 1 | -3.769 | 2.678 | -2.556 | 2.071 | 0.702 | -8.054 | 2.943 |
|  |  | 2 | 1 | 2.862 | 1.526 | 0.214 | -1.024 | 6.749 | -1.455 | 1.979 | 1 | -7.134 | 4.225 | 5.500* | 1.922 | 0.032 | 0.397 | 10.603 |
|  |  |  | 3 | 1.069 | 1.331 | 1 | -2.32 | 4.458 | -2 | 2.063 | 1 | -7.92 | 3.92 | 2.944 | 1.62 | 0.26 | -1.356 | 7.245 |
|  |  | 3 | 1 | 1.793 | 1.349 | 0.584 | -1.643 | 5.23 | 0.545 | 1.123 | 1 | -2.678 | 3.769 | 2.556 | 2.071 | 0.702 | -2.943 | 8.054 |
|  |  |  | 2 | -1.069 | 1.331 | 1 | -4.458 | 2.32 | 2 | 2.063 | 1 | -3.92 | 7.92 | -2.944 | 1.62 | 0.26 | -7.245 | 1.356 |
|  | DHEA | 1 | 2 | -2.31 | 20.837 | 1 | -55.37 | 50.749 | -3.182 | 6.37 | 1 | -21.465 | 15.101 | -1.778 | 33.726 | 1 | -91.321 | 87.766 |
|  |  |  | 3 | 17 | 12.162 | 0.519 | -13.97 | 47.97 | -3.364 | 8.373 | 1 | -27.396 | 20.669 | 29.444 | 18.528 | 0.391 | -19.747 | 78.636 |
|  |  | 2 | 1 | 2.31 | 20.837 | 1 | -50.749 | 55.37 | 3.182 | 6.37 | 1 | -15.101 | 21.465 | 1.778 | 33.726 | 1 | -87.766 | 91.321 |
|  |  |  | 3 | 19.31 | 20.917 | 1 | -33.954 | 72.575 | -0.182 | 6.411 | 1 | -18.583 | 18.219 | 31.222 | 33.529 | 1 | -57.798 | 120.242 |
|  |  | 3 | 1 | -17 | 12.162 | 0.519 | -47.97 | 13.97 | 3.364 | 8.373 | 1 | -20.669 | 27.396 | -29.444 | 18.528 | 0.391 | -78.636 | 19.747 |
|  |  |  | 2 | -19.31 | 20.917 | 1 | -72.575 | 33.954 | 0.182 | 6.411 | 1 | -18.219 | 18.583 | -31.222 | 33.529 | 1 | -120.242 | 57.798 |
|  | H1AC | 1 | 2 | 0.04 | 0.033 | 0.695 | -0.043 | 0.123 | 0.067 | 0.045 | 0.498 | -0.06 | 0.193 | 0.022 | 0.046 | 1 | -0.1 | 0.145 |
|  |  |  | 3 | 0.007 | 0.03 | 1 | -0.068 | 0.082 | 0.025 | 0.041 | 1 | -0.091 | 0.141 | -0.006 | 0.042 | 1 | -0.116 | 0.105 |
|  |  | 2 | 1 | -0.04 | 0.033 | 0.695 | -0.123 | 0.043 | -0.067 | 0.045 | 0.498 | -0.193 | 0.06 | -0.022 | 0.046 | 1 | -0.145 | 0.1 |
|  |  |  | 3 | -0.033 | 0.033 | 0.977 | -0.118 | 0.051 | -0.042 | 0.058 | 1 | -0.206 | 0.123 | -0.028 | 0.041 | 1 | -0.137 | 0.081 |
|  |  | 3 | 1 | -0.007 | 0.03 | 1 | -0.082 | 0.068 | -0.025 | 0.041 | 1 | -0.141 | 0.091 | 0.006 | 0.042 | 1 | -0.105 | 0.116 |
|  |  |  | 2 | 0.033 | 0.033 | 0.977 | -0.051 | 0.118 | 0.042 | 0.058 | 1 | -0.123 | 0.206 | 0.028 | 0.041 | 1 | -0.081 | 0.137 |
|  |  |  |  |  |  |  |  |  |  |  |  |  |  |  |  |  |  |  |
| 5mg | Calcium | 1 | 2 | -1.78E-15 | 0.066 | 1 | -0.169 | 0.169 | -0.122 | 0.083 | 0.537 | -0.372 | 0.128 | 0.058 | 0.088 | 1 | -0.173 | 0.289 |
|  |  |  | 3 | 0.1 | 0.08 | 0.664 | -0.104 | 0.304 | 0.078 | 0.124 | 1 | -0.298 | 0.453 | 0.111 | 0.104 | 0.906 | -0.164 | 0.385 |
|  |  | 2 | 1 | 1.78E-15 | 0.066 | 1 | -0.169 | 0.169 | 0.122 | 0.083 | 0.537 | -0.128 | 0.372 | -0.058 | 0.088 | 1 | -0.289 | 0.173 |
|  |  |  | 3 | 0.1 | 0.072 | 0.522 | -0.083 | 0.283 | 0.2 | 0.114 | 0.354 | -0.145 | 0.545 | 0.053 | 0.091 | 1 | -0.186 | 0.292 |
|  |  | 3 | 1 | -0.1 | 0.08 | 0.664 | -0.304 | 0.104 | -0.078 | 0.124 | 1 | -0.453 | 0.298 | -0.111 | 0.104 | 0.906 | -0.385 | 0.164 |
|  |  |  | 2 | -0.1 | 0.072 | 0.522 | -0.283 | 0.083 | -0.2 | 0.114 | 0.354 | -0.545 | 0.145 | -0.053 | 0.091 | 1 | -0.292 | 0.186 |
|  | Uric Acid | 1 | 2 | 0.243 | 0.15 | 0.354 | -0.141 | 0.627 | 0.211 | 0.166 | 0.719 | -0.29 | 0.712 | 0.258 | 0.21 | 0.706 | -0.296 | 0.812 |
|  |  |  | 3 | 0.379 | 0.159 | 0.075 | -0.028 | 0.786 | 0.522 | 0.25 | 0.212 | -0.233 | 1.277 | 0.311 | 0.205 | 0.444 | -0.232 | 0.853 |
|  |  | 2 | 1 | -0.243 | 0.15 | 0.354 | -0.627 | 0.141 | -0.211 | 0.166 | 0.719 | -0.712 | 0.29 | -0.258 | 0.21 | 0.706 | -0.812 | 0.296 |
|  |  |  | 3 | 0.136 | 0.213 | 1 | -0.407 | 0.679 | 0.311 | 0.252 | 0.758 | -0.45 | 1.072 | 0.053 | 0.292 | 1 | -0.719 | 0.824 |
|  |  | 3 | 1 | -0.379 | 0.159 | 0.075 | -0.786 | 0.028 | -0.522 | 0.25 | 0.212 | -1.277 | 0.233 | -0.311 | 0.205 | 0.444 | -0.853 | 0.232 |
|  |  |  | 2 | -0.136 | 0.213 | 1 | -0.679 | 0.407 | -0.311 | 0.252 | 0.758 | -1.072 | 0.45 | -0.053 | 0.292 | 1 | -0.824 | 0.719 |
|  | WBC | 1 | 2 | -0.046 | 0.229 | 1 | -0.632 | 0.539 | -0.711 | 0.452 | 0.464 | -2.075 | 0.653 | 0.268 | 0.237 | 0.815 | -0.356 | 0.893 |
|  |  |  | 3 | -0.196 | 0.193 | 0.95 | -0.688 | 0.295 | -0.478 | 0.388 | 0.758 | -1.647 | 0.691 | -0.063 | 0.217 | 1 | -0.637 | 0.511 |
|  |  | 2 | 1 | 0.046 | 0.229 | 1 | -0.539 | 0.632 | 0.711 | 0.452 | 0.464 | -0.653 | 2.075 | -0.268 | 0.237 | 0.815 | -0.893 | 0.356 |
|  |  |  | 3 | -0.15 | 0.212 | 1 | -0.692 | 0.392 | 0.233 | 0.431 | 1 | -1.066 | 1.532 | -0.332 | 0.234 | 0.52 | -0.949 | 0.286 |
|  |  | 3 | 1 | 0.196 | 0.193 | 0.95 | -0.295 | 0.688 | 0.478 | 0.388 | 0.758 | -0.691 | 1.647 | 0.063 | 0.217 | 1 | -0.511 | 0.637 |
|  |  |  | 2 | 0.15 | 0.212 | 1 | -0.392 | 0.692 | -0.233 | 0.431 | 1 | -1.532 | 1.066 | 0.332 | 0.234 | 0.52 | -0.286 | 0.949 |
|  | Hematocrit | 1 | 2 | -1.075 | 0.437 | 0.061 | -2.189 | 0.039 | -1.056 | 0.775 | 0.631 | -3.393 | 1.282 | -1.084 | 0.543 | 0.184 | -2.517 | 0.349 |
|  |  |  | 3 | -1.104 | 0.568 | 0.187 | -2.553 | 0.346 | -1.878 | 0.724 | 0.096 | -4.06 | 0.304 | -0.737 | 0.761 | 1 | -2.746 | 1.272 |
|  |  | 2 | 1 | 1.075 | 0.437 | 0.061 | -0.039 | 2.189 | 1.056 | 0.775 | 0.631 | -1.282 | 3.393 | 1.084 | 0.543 | 0.184 | -0.349 | 2.517 |
|  |  |  | 3 | -0.029 | 0.487 | 1 | -1.271 | 1.214 | -0.822 | 0.552 | 0.525 | -2.488 | 0.844 | 0.347 | 0.66 | 1 | -1.394 | 2.089 |
|  |  | 3 | 1 | 1.104 | 0.568 | 0.187 | -0.346 | 2.553 | 1.878 | 0.724 | 0.096 | -0.304 | 4.06 | 0.737 | 0.761 | 1 | -1.272 | 2.746 |
|  |  |  | 2 | 0.029 | 0.487 | 1 | -1.214 | 1.271 | 0.822 | 0.552 | 0.525 | -0.844 | 2.488 | -0.347 | 0.66 | 1 | -2.089 | 1.394 |
|  | Absolute Neutrophils | 1 | 2 | -24.036 | 220.037 | 1 | -585.672 | 537.6 | -738.778 | 432.932 | 0.379 | -2044.399 | 566.843 | 314.526 | 218.529 | 0.502 | -262.205 | 891.257 |
|  |  |  | 3 | -226.821 | 209.792 | 0.868 | -762.308 | 308.665 | -600.667 | 464.881 | 0.697 | -2002.639 | 801.305 | -49.737 | 215.345 | 1 | -618.064 | 518.59 |
|  |  | 2 | 1 | 24.036 | 220.037 | 1 | -537.6 | 585.672 | 738.778 | 432.932 | 0.379 | -566.843 | 2044.399 | -314.526 | 218.529 | 0.502 | -891.257 | 262.205 |
|  |  |  | 3 | -202.786 | 230.212 | 1 | -790.392 | 384.82 | 138.111 | 596.199 | 1 | -1659.884 | 1936.106 | -364.263 | 194.456 | 0.232 | -877.46 | 148.934 |
|  |  | 3 | 1 | 226.821 | 209.792 | 0.868 | -308.665 | 762.308 | 600.667 | 464.881 | 0.697 | -801.305 | 2002.639 | 49.737 | 215.345 | 1 | -518.59 | 618.064 |
|  |  |  | 2 | 202.786 | 230.212 | 1 | -384.82 | 790.392 | -138.111 | 596.199 | 1 | -1936.106 | 1659.884 | 364.263 | 194.456 | 0.232 | -148.934 | 877.46 |
|  | Absolute Lymphocytes | 1 | 2 | -37.143 | 52.966 | 1 | -172.337 | 98.051 | -10.111 | 107.199 | 1 | -333.397 | 313.175 | -49.947 | 61.269 | 1 | -211.644 | 111.749 |
|  |  |  | 3 | 22.821 | 64.496 | 1 | -141.802 | 187.445 | 44.667 | 170.01 | 1 | -468.042 | 557.375 | 12.474 | 55.604 | 1 | -134.274 | 159.221 |
|  |  | 2 | 1 | 37.143 | 52.966 | 1 | -98.051 | 172.337 | 10.111 | 107.199 | 1 | -313.175 | 333.397 | 49.947 | 61.269 | 1 | -111.749 | 211.644 |
|  |  |  | 3 | 59.964 | 57.689 | 0.923 | -87.285 | 207.213 | 54.778 | 154.511 | 1 | -411.191 | 520.746 | 62.421 | 48.266 | 0.637 | -64.961 | 189.803 |
|  |  | 3 | 1 | -22.821 | 64.496 | 1 | -187.445 | 141.802 | -44.667 | 170.01 | 1 | -557.375 | 468.042 | -12.474 | 55.604 | 1 | -159.221 | 134.274 |
|  |  |  | 2 | -59.964 | 57.689 | 0.923 | -207.213 | 87.285 | -54.778 | 154.511 | 1 | -520.746 | 411.191 | -62.421 | 48.266 | 0.637 | -189.803 | 64.961 |
|  | Abs Monocytes | 1 | 2 | 1.893 | 19.431 | 1 | -47.705 | 51.49 | -13.222 | 30.584 | 1 | -105.458 | 79.013 | 9.053 | 25.077 | 1 | -57.129 | 75.234 |
|  |  |  | 3 | -0.5 | 17.909 | 1 | -46.212 | 45.212 | 26.667 | 33.29 | 1 | -73.729 | 127.063 | -13.368 | 21.143 | 1 | -69.169 | 42.432 |
|  |  | 2 | 1 | -1.893 | 19.431 | 1 | -51.49 | 47.705 | 13.222 | 30.584 | 1 | -79.013 | 105.458 | -9.053 | 25.077 | 1 | -75.234 | 57.129 |
|  |  |  | 3 | -2.393 | 16.252 | 1 | -43.876 | 39.09 | 39.889 | 25.295 | 0.46 | -36.394 | 116.171 | -22.421 | 19.49 | 0.795 | -73.857 | 29.015 |
|  |  | 3 | 1 | 0.5 | 17.909 | 1 | -45.212 | 46.212 | -26.667 | 33.29 | 1 | -127.063 | 73.729 | 13.368 | 21.143 | 1 | -42.432 | 69.169 |
|  |  |  | 2 | 2.393 | 16.252 | 1 | -39.09 | 43.876 | -39.889 | 25.295 | 0.46 | -116.171 | 36.394 | 22.421 | 19.49 | 0.795 | -29.015 | 73.857 |
|  | Neutrophils | 1 | 2 | 0.046 | 1.729 | 1 | -4.366 | 4.458 | -5.111 | 2.976 | 0.373 | -14.085 | 3.863 | 2.489 | 1.926 | 0.637 | -2.593 | 7.572 |
|  |  |  | 3 | -1.921 | 1.739 | 0.837 | -6.36 | 2.517 | -4.922 | 3.484 | 0.586 | -15.428 | 5.584 | -0.5 | 1.944 | 1 | -5.631 | 4.631 |
|  |  | 2 | 1 | -0.046 | 1.729 | 1 | -4.458 | 4.366 | 5.111 | 2.976 | 0.373 | -3.863 | 14.085 | -2.489 | 1.926 | 0.637 | -7.572 | 2.593 |
|  |  |  | 3 | -1.968 | 1.72 | 0.788 | -6.358 | 2.422 | 0.189 | 4.704 | 1 | -13.999 | 14.376 | -2.989 | 1.304 | 0.102 | -6.43 | 0.451 |
|  |  | 3 | 1 | 1.921 | 1.739 | 0.837 | -2.517 | 6.36 | 4.922 | 3.484 | 0.586 | -5.584 | 15.428 | 0.5 | 1.944 | 1 | -4.631 | 5.631 |
|  |  |  | 2 | 1.968 | 1.72 | 0.788 | -2.422 | 6.358 | -0.189 | 4.704 | 1 | -14.376 | 13.999 | 2.989 | 1.304 | 0.102 | -0.451 | 6.43 |
|  | Lymphocytes | 1 | 2 | -0.368 | 1.341 | 1 | -3.791 | 3.055 | 2.967 | 1.969 | 0.511 | -2.973 | 8.906 | -1.947 | 1.652 | 0.761 | -6.306 | 2.411 |
|  |  |  | 3 | 1.318 | 1.381 | 1 | -2.208 | 4.844 | 2.722 | 2.955 | 1 | -6.191 | 11.635 | 0.653 | 1.518 | 1 | -3.353 | 4.658 |
|  |  | 2 | 1 | 0.368 | 1.341 | 1 | -3.055 | 3.791 | -2.967 | 1.969 | 0.511 | -8.906 | 2.973 | 1.947 | 1.652 | 0.761 | -2.411 | 6.306 |
|  |  |  | 3 | 1.686 | 1.347 | 0.665 | -1.753 | 5.124 | -0.244 | 3.572 | 1 | -11.017 | 10.528 | 2.6 | 1.087 | 0.084 | -0.268 | 5.468 |
|  |  | 3 | 1 | -1.318 | 1.381 | 1 | -4.844 | 2.208 | -2.722 | 2.955 | 1 | -11.635 | 6.191 | -0.653 | 1.518 | 1 | -4.658 | 3.353 |
|  |  |  | 2 | -1.686 | 1.347 | 0.665 | -5.124 | 1.753 | 0.244 | 3.572 | 1 | -10.528 | 11.017 | -2.6 | 1.087 | 0.084 | -5.468 | 0.268 |
|  | CO2 | 1 | 2 | 1.250* | 0.438 | 0.025 | 0.131 | 2.369 | 2.556* | 0.648 | 0.013 | 0.602 | 4.509 | 0.632 | 0.52 | 0.72 | -0.74 | 2.004 |
|  |  |  | 3 | 0.75 | 0.444 | 0.309 | -0.384 | 1.884 | 1 | 0.624 | 0.442 | -0.881 | 2.881 | 0.632 | 0.593 | 0.904 | -0.934 | 2.198 |
|  |  | 2 | 1 | -1.250* | 0.438 | 0.025 | -2.369 | -0.131 | -2.556* | 0.648 | 0.013 | -4.509 | -0.602 | -0.632 | 0.52 | 0.72 | -2.004 | 0.74 |
|  |  |  | 3 | -0.5 | 0.47 | 0.89 | -1.7 | 0.7 | -1.556 | 0.669 | 0.146 | -3.573 | 0.462 | 0 | 0.592 | 1 | -1.563 | 1.563 |
|  |  | 3 | 1 | -0.75 | 0.444 | 0.309 | -1.884 | 0.384 | -1 | 0.624 | 0.442 | -2.881 | 0.881 | -0.632 | 0.593 | 0.904 | -2.198 | 0.934 |
|  |  |  | 2 | 0.5 | 0.47 | 0.89 | -0.7 | 1.7 | 1.556 | 0.669 | 0.146 | -0.462 | 3.573 | 0 | 0.592 | 1 | -1.563 | 1.563 |
|  | eGFR | 1 | 2 | -0.357 | 1.709 | 1 | -4.72 | 4.005 | 0.778 | 0.662 | 0.822 | -1.219 | 2.774 | -0.895 | 2.513 | 1 | -7.527 | 5.737 |
|  |  |  | 3 | -1.464 | 1.524 | 1 | -5.353 | 2.425 | 1.667 | 1.922 | 1 | -4.13 | 7.463 | -2.947 | 1.993 | 0.47 | -8.208 | 2.313 |
|  |  | 2 | 1 | 0.357 | 1.709 | 1 | -4.005 | 4.72 | -0.778 | 0.662 | 0.822 | -2.774 | 1.219 | 0.895 | 2.513 | 1 | -5.737 | 7.527 |
|  |  |  | 3 | -1.107 | 1.744 | 1 | -5.558 | 3.344 | 0.889 | 1.889 | 1 | -4.808 | 6.585 | -2.053 | 2.412 | 1 | -8.417 | 4.312 |
|  |  | 3 | 1 | 1.464 | 1.524 | 1 | -2.425 | 5.353 | -1.667 | 1.922 | 1 | -7.463 | 4.13 | 2.947 | 1.993 | 0.47 | -2.313 | 8.208 |
|  |  |  | 2 | 1.107 | 1.744 | 1 | -3.344 | 5.558 | -0.889 | 1.889 | 1 | -6.585 | 4.808 | 2.053 | 2.412 | 1 | -4.312 | 8.417 |
|  | DHEA | 1 | 2 | 19.679 | 13.815 | 0.497 | -15.584 | 54.941 | 9.333 | 8.403 | 0.897 | -16.008 | 34.675 | 24.579 | 20.072 | 0.71 | -28.394 | 77.551 |
|  |  |  | 3 | 29.286 | 13.235 | 0.107 | -4.496 | 63.067 | 18.667 | 8.549 | 0.182 | -7.115 | 44.448 | 34.316 | 19.169 | 0.271 | -16.274 | 84.905 |
|  |  | 2 | 1 | -19.679 | 13.815 | 0.497 | -54.941 | 15.584 | -9.333 | 8.403 | 0.897 | -34.675 | 16.008 | -24.579 | 20.072 | 0.71 | -77.551 | 28.394 |
|  |  |  | 3 | 9.607 | 9.079 | 0.898 | -13.566 | 32.78 | 9.333* | 2.824 | 0.032 | 0.818 | 17.848 | 9.737 | 13.436 | 1 | -25.722 | 45.196 |
|  |  | 3 | 1 | -29.286 | 13.235 | 0.107 | -63.067 | 4.496 | -18.667 | 8.549 | 0.182 | -44.448 | 7.115 | -34.316 | 19.169 | 0.271 | -84.905 | 16.274 |
|  |  |  | 2 | -9.607 | 9.079 | 0.898 | -32.78 | 13.566 | -9.333* | 2.824 | 0.032 | -17.848 | -0.818 | -9.737 | 13.436 | 1 | -45.196 | 25.722 |
|  | H1AC | 1 | 2 | -0.015 | 0.022 | 1 | -0.071 | 0.042 | 0 | 0.028 | 1 | -0.077 | 0.077 | -0.024 | 0.032 | 1 | -0.108 | 0.061 |
|  |  |  | 3 | -.082* | 0.027 | 0.013 | -0.15 | -0.015 | -0.046 | 0.04 | 0.82 | -0.158 | 0.066 | -.105* | 0.036 | 0.024 | -0.198 | -0.012 |
|  |  | 2 | 1 | 0.015 | 0.022 | 1 | -0.042 | 0.071 | 0 | 0.028 | 1 | -0.077 | 0.077 | 0.024 | 0.032 | 1 | -0.061 | 0.108 |
|  |  |  | 3 | -0.068 | 0.029 | 0.076 | -0.14 | 0.005 | -0.046 | 0.039 | 0.764 | -0.153 | 0.061 | -0.081 | 0.041 | 0.18 | -0.187 | 0.025 |
|  |  | 3 | 1 | .082* | 0.027 | 0.013 | 0.015 | 0.15 | 0.046 | 0.04 | 0.82 | -0.066 | 0.158 | .105* | 0.036 | 0.024 | 0.012 | 0.198 |
|  |  |  | 2 | 0.068 | 0.029 | 0.076 | -0.005 | 0.14 | 0.046 | 0.039 | 0.764 | -0.061 | 0.153 | 0.081 | 0.041 | 0.18 | -0.025 | 0.187 |
|  |  |  |  |  |  |  |  |  |  |  |  |  |  |  |  |  |  |  |
| *df = degrees of freedom, provided as: between groups, within groups* | | | | | | | | | | | | | | | | | | |
| *^denotes use of Welch's ANOVA in instances that lack homogeneity of variances* | | | | | | | | | | | | | | | | | | |
| * *p* ≤ 0.05 | | | | | | | | | | | | | | | | | | |
| ***effect size provided as epsilon squared for ANOVA or omega squared for Welch's ANOVA* | | | | | | | | | | | | | | | | | | |

| **Supplementary Table 7. Analysis of gut microbiome measures** | | | | | | | | | | |
| --- | --- | --- | --- | --- | --- | --- | --- | --- | --- | --- |
|  |  |  |  |  |  |  |  |  |  |  |
| **Means and Standard Deviations** | | | | |  |  |  |  |  |  |
|  |  | All Genders | | | Females | | | Males | | |
|  | Group | Mean | Std. Deviation | N | Mean | Std. Deviation | N | Mean | Std. Deviation | N |
| Digestion 0 | 10mg | 60.459 | 10.0754 | 22 | 60.92 | 5.9424 | 5 | 60.324 | 11.15 | 17 |
|  | Placebo | 58.41 | 11.8597 | 30 | 59.255 | 11.6388 | 11 | 57.921 | 12.2745 | 19 |
|  | 5mg | 59.552 | 11.2451 | 29 | 56.673 | 10.9346 | 15 | 62.636 | 11.1262 | 14 |
|  | Total | 59.375 | 11.0714 | 81 | 58.274 | 10.4144 | 31 | 60.058 | 11.5099 | 50 |
| Digestion 48 | 10mg | 60.605 | 11.1224 | 22 | 61.78 | 10.7707 | 5 | 60.259 | 11.5241 | 17 |
|  | Placebo | 58.337 | 14.6162 | 30 | 59.827 | 14.1213 | 11 | 57.474 | 15.2072 | 19 |
|  | 5mg | 62.145 | 9.7443 | 29 | 59.273 | 7.8171 | 15 | 65.221 | 10.9085 | 14 |
|  | Total | 60.316 | 12.0769 | 81 | 59.874 | 10.5471 | 31 | 60.59 | 13.0315 | 50 |
| Inflammation 0 | 10mg | 41.423 | 13.7167 | 22 | 44.28 | 9.2546 | 5 | 40.582 | 14.9074 | 17 |
|  | Placebo | 39.257 | 9.8357 | 30 | 39.2 | 12.1249 | 11 | 39.289 | 8.6131 | 19 |
|  | 5mg | 42.431 | 15.7346 | 29 | 42.093 | 15.7762 | 15 | 42.793 | 16.2769 | 14 |
|  | Total | 40.981 | 13.1545 | 81 | 41.419 | 13.4152 | 31 | 40.71 | 13.12 | 50 |
| Inflammation 48 | 10mg | 42.773 | 11.3754 | 22 | 48.48 | 8.4141 | 5 | 41.094 | 11.7884 | 17 |
|  | Placebo | 43.523 | 13.6108 | 30 | 43.145 | 15.9294 | 11 | 43.742 | 12.5442 | 19 |
|  | 5mg | 45.814 | 16.7092 | 29 | 46.36 | 17.2844 | 15 | 45.229 | 16.7005 | 14 |
|  | Total | 44.14 | 14.1603 | 81 | 45.561 | 15.4051 | 31 | 43.258 | 13.4165 | 50 |
| Gut Dysbiosis 0 | 10mg | 73.073 | 4.7823 | 22 | 73.32 | 6.3759 | 5 | 73 | 4.4531 | 17 |
|  | Placebo | 73.187 | 8.0555 | 30 | 74.855 | 10.0916 | 11 | 72.221 | 6.7293 | 19 |
|  | 5mg | 73.51 | 7.3798 | 29 | 73.48 | 7.1793 | 15 | 73.543 | 7.8609 | 14 |
|  | Total | 73.272 | 6.973 | 81 | 73.942 | 7.9935 | 31 | 72.856 | 6.3089 | 50 |
| Gut Dysbiosis 48 | 10mg | 74.645 | 4.1743 | 22 | 72.64 | 4.8195 | 5 | 75.235 | 3.9289 | 17 |
|  | Placebo | 73.813 | 7.2346 | 30 | 75.364 | 8.8072 | 11 | 72.916 | 6.238 | 19 |
|  | 5mg | 73.814 | 7.1839 | 29 | 73.747 | 7.2216 | 15 | 73.886 | 7.415 | 14 |
|  | Total | 74.04 | 6.4613 | 81 | 74.142 | 7.3683 | 31 | 73.976 | 5.9085 | 50 |
| Intestinal Permeability 0 | 10mg | 42.6 | 14.9818 | 22 | 49.2 | 8.6432 | 5 | 40.659 | 16.0718 | 17 |
|  | Placebo | 40.51 | 11.3282 | 30 | 44.355 | 13.5367 | 11 | 38.284 | 9.523 | 19 |
|  | 5mg | 44.241 | 17.9196 | 29 | 43.093 | 18.484 | 15 | 45.471 | 17.9035 | 14 |
|  | Total | 42.414 | 14.8461 | 81 | 44.526 | 15.3349 | 31 | 41.104 | 14.5362 | 50 |
| Intestinal Permeability 48 | 10mg | 43.091 | 12.0229 | 22 | 52.22 | 7.664 | 5 | 40.406 | 11.8884 | 17 |
|  | Placebo | 44.737 | 14.8242 | 30 | 47.718 | 17.9468 | 11 | 43.011 | 12.9049 | 19 |
|  | 5mg | 47.528 | 17.738 | 29 | 46.687 | 19.4035 | 15 | 48.429 | 16.4477 | 14 |
|  | Total | 45.289 | 15.1983 | 81 | 47.945 | 17.1682 | 31 | 43.642 | 13.7635 | 50 |
| Nervous System 0 | 10mg | 42.864 | 11.9295 | 22 | 45.2 | 6.6483 | 5 | 42.176 | 13.1731 | 17 |
|  | Placebo | 44.033 | 13.7652 | 30 | 43.182 | 14.2114 | 11 | 44.526 | 13.8699 | 19 |
|  | 5mg | 44.241 | 12.1938 | 29 | 43.467 | 14.7739 | 15 | 45.071 | 9.1523 | 14 |
|  | Total | 43.79 | 12.5864 | 81 | 43.645 | 13.2503 | 31 | 43.88 | 12.2934 | 50 |
| Nervous System 48 | 10mg | 42.955 | 11.8983 | 22 | 45.4 | 6.3482 | 5 | 42.235 | 13.165 | 17 |
|  | Placebo | 41.1 | 12.1778 | 30 | 41.636 | 13.6622 | 11 | 40.789 | 11.6169 | 19 |
|  | 5mg | 42.724 | 10.8097 | 29 | 43.067 | 12.6799 | 15 | 42.357 | 8.8457 | 14 |
|  | Total | 42.185 | 11.5121 | 81 | 42.935 | 12.0109 | 31 | 41.72 | 11.2903 | 50 |
| Diversity Score 0 | 10mg | 54.818 | 25.8081 | 22 | 68.8 | 32.8512 | 5 | 50.706 | 22.9204 | 17 |
|  | Placebo | 56.167 | 30.2986 | 30 | 77.909 | 15.5271 | 11 | 43.579 | 29.8148 | 19 |
|  | 5mg | 65.172 | 28.5445 | 29 | 69.133 | 25.6065 | 15 | 60.929 | 31.801 | 14 |
|  | Total | 59.025 | 28.5381 | 81 | 72.194 | 23.4271 | 31 | 50.86 | 28.5643 | 50 |
| Diversity Score 48 | 10mg | 54.318 | 23.7354 | 22 | 48.6 | 20.7196 | 5 | 56 | 24.8772 | 17 |
|  | Placebo | 64.633 | 27.5562 | 30 | 77.727 | 20.3573 | 11 | 57.053 | 28.7701 | 19 |
|  | 5mg | 63.034 | 29.0805 | 29 | 70.667 | 26.5402 | 15 | 54.857 | 30.3919 | 14 |
|  | Total | 61.259 | 27.1624 | 81 | 69.613 | 24.9475 | 31 | 56.08 | 27.4225 | 50 |
| Immune Readiness 0 | 10mg | 45.182 | 5.7952 | 22 | 46.4 | 8.2037 | 5 | 44.824 | 5.1628 | 17 |
|  | Placebo | 45 | 10.8881 | 30 | 40.364 | 12.2741 | 11 | 47.684 | 9.3038 | 19 |
|  | 5mg | 44.552 | 11.2523 | 29 | 43.467 | 8.9512 | 15 | 45.714 | 13.5501 | 14 |
|  | Total | 44.889 | 9.8069 | 81 | 42.839 | 10.0568 | 31 | 46.16 | 9.5284 | 50 |
| Immune ReadinessScore 48 | 10mg | 43.909 | 6.8376 | 22 | 45.8 | 7.5299 | 5 | 43.353 | 6.7633 | 17 |
|  | Placebo | 43.333 | 10.0149 | 30 | 38.364 | 11.5176 | 11 | 46.211 | 8.004 | 19 |
|  | 5mg | 44.345 | 9.8571 | 29 | 44.867 | 9.8116 | 15 | 43.786 | 10.2445 | 14 |
|  | Total | 43.852 | 9.101 | 81 | 42.71 | 10.3705 | 31 | 44.56 | 8.2491 | 50 |

| **Repeated Measures Mixed ANOVA** | | | | | | | | | | | | | | | |
| --- | --- | --- | --- | --- | --- | --- | --- | --- | --- | --- | --- | --- | --- | --- | --- |
| All Genders |  |  |  |  |  | Females |  |  |  |  | Males |  |  |  |  |
| Measure | df 1 | df 2 | F | p-value | Partial Eta Squared | df 1 | df 2 | F | p-value | Partial Eta Squared | df 1 | df 2 | F | p-value | Partial Eta Squared |
| Digestion | 2 | 78 | 0.634 | 0.533 | 0.016 | 2 | 28 | 0.169 | 0.845 | 0.012 | 2 | 47 | 0.378 | 0.687 | 0.016 |
| Inflammation | 2 | 78 | 0.378 | 0.686 | 0.01 | 2 | 28 | 0.002 | 0.998 | 0 | 2 | 47 | 0.636 | 0.534 | 0.026 |
| Gut Dysbiosis | 2 | 78 | 0.169 | 0.845 | 0.004 | 2 | 28 | 0.026 | 0.974 | 0.002 | 2 | 47 | 0.36 | 0.699 | 0.015 |
| Intestinal Permeability | 2 | 78 | 0.371 | 0.691 | 0.009 | 2 | 28 | 0.002 | 0.998 | 0 | 2 | 47 | 0.658 | 0.523 | 0.027 |
| Nervous System | 2 | 78 | 0.784 | 0.46 | 0.02 | 2 | 28 | 0.109 | 0.897 | 0.008 | 2 | 47 | 0.793 | 0.459 | 0.033 |
| Diversity | 2 | 78 | 0.91 | 0.407 | 0.023 | 2 | 28 | 1.119 | 0.341 | 0.074 | 2 | 47 | 1.362 | 0.266 | 0.055 |
| Immune Readiness | 2 | 78 | 0.13 | 0.878 | 0.003 | 2 | 28 | 0.267 | 0.768 | 0.019 | 2 | 47 | 0.008 | 0.992 | 0 |

| **Pairwise Comparisons** | | | | |  |  |  |  |  |  |  |  |  |  |  |  |  |  |
| --- | --- | --- | --- | --- | --- | --- | --- | --- | --- | --- | --- | --- | --- | --- | --- | --- | --- | --- |
|  |  |  |  | All Genders | | | | | Females | | | | | Males | | | | |
| Measure | Group | Time 1 | Time 2 | Mean Difference | Std. Error | p-value | 95% Confidence Interval for Differencea |  | Mean Difference | Std. Error | p-value | 95% Confidence Interval for Differencea |  | Mean Difference | Std. Error | p-value | 95% Confidence Interval for Differencea |  |
|  |  |  |  |  |  |  | Lower Bound | Upper Bound |  |  |  | Lower Bound | Upper Bound |  |  |  | Lower Bound | Upper Bound |
| Digestion | 10mg | 1 | 2 | -0.145 | 2.108 | 0.945 | -4.341 | 4.05 | -0.86 | 4.165 | 0.838 | -9.392 | 7.672 | 0.065 | 2.547 | 0.98 | -5.059 | 5.188 |
|  |  | 2 | 1 | 0.145 | 2.108 | 0.945 | -4.05 | 4.341 | 0.86 | 4.165 | 0.838 | -7.672 | 9.392 | -0.065 | 2.547 | 0.98 | -5.188 | 5.059 |
|  | Placebo | 1 | 2 | 0.073 | 1.805 | 0.968 | -3.52 | 3.666 | -0.573 | 2.808 | 0.84 | -6.325 | 5.18 | 0.447 | 2.409 | 0.853 | -4.399 | 5.294 |
|  |  | 2 | 1 | -0.073 | 1.805 | 0.968 | -3.666 | 3.52 | 0.573 | 2.808 | 0.84 | -5.18 | 6.325 | -0.447 | 2.409 | 0.853 | -5.294 | 4.399 |
|  | 5mg | 1 | 2 | -2.593 | 1.836 | 0.162 | -6.248 | 1.061 | -2.6 | 2.405 | 0.289 | -7.526 | 2.326 | -2.586 | 2.806 | 0.362 | -8.232 | 3.06 |
|  |  | 2 | 1 | 2.593 | 1.836 | 0.162 | -1.061 | 6.248 | 2.6 | 2.405 | 0.289 | -2.326 | 7.526 | 2.586 | 2.806 | 0.362 | -3.06 | 8.232 |
| Inflammation | 10mg | 1 | 2 | -1.35 | 2.574 | 0.601 | -6.474 | 3.774 | -4.2 | 6.618 | 0.531 | -17.757 | 9.357 | -0.512 | 2.54 | 0.841 | -5.622 | 4.599 |
|  |  | 2 | 1 | 1.35 | 2.574 | 0.601 | -3.774 | 6.474 | 4.2 | 6.618 | 0.531 | -9.357 | 17.757 | 0.512 | 2.54 | 0.841 | -4.599 | 5.622 |
|  | Placebo | 1 | 2 | -4.267 | 2.204 | 0.057 | -8.654 | 0.121 | -3.945 | 4.462 | 0.384 | -13.085 | 5.194 | -4.453 | 2.403 | 0.07 | -9.287 | 0.381 |
|  |  | 2 | 1 | 4.267 | 2.204 | 0.057 | -0.121 | 8.654 | 3.945 | 4.462 | 0.384 | -5.194 | 13.085 | 4.453 | 2.403 | 0.07 | -0.381 | 9.287 |
|  | 5mg | 1 | 2 | -3.383 | 2.242 | 0.135 | -7.846 | 1.08 | -4.267 | 3.821 | 0.274 | -12.094 | 3.56 | -2.436 | 2.799 | 0.389 | -8.067 | 3.196 |
|  |  | 2 | 1 | 3.383 | 2.242 | 0.135 | -1.08 | 7.846 | 4.267 | 3.821 | 0.274 | -3.56 | 12.094 | 2.436 | 2.799 | 0.389 | -3.196 | 8.067 |
| Gut Dysbiosis | 10mg | 1 | 2 | -1.573 | 1.685 | 0.353 | -4.927 | 1.781 | 0.68 | 4.378 | 0.878 | -8.288 | 9.648 | -2.235 | 1.642 | 0.18 | -5.538 | 1.068 |
|  |  | 2 | 1 | 1.573 | 1.685 | 0.353 | -1.781 | 4.927 | -0.68 | 4.378 | 0.878 | -9.648 | 8.288 | 2.235 | 1.642 | 0.18 | -1.068 | 5.538 |
|  | Placebo | 1 | 2 | -0.627 | 1.443 | 0.665 | -3.499 | 2.246 | -0.509 | 2.952 | 0.864 | -6.555 | 5.537 | -0.695 | 1.553 | 0.657 | -3.819 | 2.43 |
|  |  | 2 | 1 | 0.627 | 1.443 | 0.665 | -2.246 | 3.499 | 0.509 | 2.952 | 0.864 | -5.537 | 6.555 | 0.695 | 1.553 | 0.657 | -2.43 | 3.819 |
|  | 5mg | 1 | 2 | -0.303 | 1.467 | 0.837 | -3.225 | 2.618 | -0.267 | 2.528 | 0.917 | -5.444 | 4.911 | -0.343 | 1.809 | 0.851 | -3.983 | 3.297 |
|  |  | 2 | 1 | 0.303 | 1.467 | 0.837 | -2.618 | 3.225 | 0.267 | 2.528 | 0.917 | -4.911 | 5.444 | 0.343 | 1.809 | 0.851 | -3.297 | 3.983 |
| Intestinal Permeability | 10mg | 1 | 2 | -0.491 | 3.362 | 0.884 | -7.185 | 6.203 | -3.02 | 8.973 | 0.739 | -21.4 | 15.36 | 0.253 | 3.178 | 0.937 | -6.141 | 6.647 |
|  |  | 2 | 1 | 0.491 | 3.362 | 0.884 | -6.203 | 7.185 | 3.02 | 8.973 | 0.739 | -15.36 | 21.4 | -0.253 | 3.178 | 0.937 | -6.647 | 6.141 |
|  | Placebo | 1 | 2 | -4.227 | 2.879 | 0.146 | -9.959 | 1.506 | -3.364 | 6.049 | 0.583 | -15.755 | 9.028 | -4.726 | 3.006 | 0.123 | -10.774 | 1.321 |
|  |  | 2 | 1 | 4.227 | 2.879 | 0.146 | -1.506 | 9.959 | 3.364 | 6.049 | 0.583 | -9.028 | 15.755 | 4.726 | 3.006 | 0.123 | -1.321 | 10.774 |
|  | 5mg | 1 | 2 | -3.286 | 2.929 | 0.265 | -9.116 | 2.544 | -3.593 | 5.18 | 0.494 | -14.205 | 7.018 | -2.957 | 3.502 | 0.403 | -10.003 | 4.088 |
|  |  | 2 | 1 | 3.286 | 2.929 | 0.265 | -2.544 | 9.116 | 3.593 | 5.18 | 0.494 | -7.018 | 14.205 | 2.957 | 3.502 | 0.403 | -4.088 | 10.003 |
| Nervous System | 10mg | 1 | 2 | -0.091 | 1.837 | 0.961 | -3.748 | 3.566 | -0.2 | 3.492 | 0.955 | -7.353 | 6.953 | -0.059 | 2.24 | 0.979 | -4.565 | 4.448 |
|  |  | 2 | 1 | 0.091 | 1.837 | 0.961 | -3.566 | 3.748 | 0.2 | 3.492 | 0.955 | -6.953 | 7.353 | 0.059 | 2.24 | 0.979 | -4.448 | 4.565 |
|  | Placebo | 1 | 2 | 2.933 | 1.573 | 0.066 | -0.198 | 6.065 | 1.545 | 2.354 | 0.517 | -3.277 | 6.368 | 3.737 | 2.119 | 0.084 | -0.526 | 8 |
|  |  | 2 | 1 | -2.933 | 1.573 | 0.066 | -6.065 | 0.198 | -1.545 | 2.354 | 0.517 | -6.368 | 3.277 | -3.737 | 2.119 | 0.084 | -8 | 0.526 |
|  | 5mg | 1 | 2 | 1.517 | 1.6 | 0.346 | -1.668 | 4.702 | 0.4 | 2.016 | 0.844 | -3.73 | 4.53 | 2.714 | 2.468 | 0.277 | -2.252 | 7.68 |
|  |  | 2 | 1 | -1.517 | 1.6 | 0.346 | -4.702 | 1.668 | -0.4 | 2.016 | 0.844 | -4.53 | 3.73 | -2.714 | 2.468 | 0.277 | -7.68 | 2.252 |
| Diversity | 10mg | 1 | 2 | 0.5 | 6.86 | 0.942 | -13.158 | 14.158 | 20.2 | 12.928 | 0.129 | -6.282 | 46.682 | -5.294 | 8.155 | 0.519 | -21.7 | 11.111 |
|  |  | 2 | 1 | -0.5 | 6.86 | 0.942 | -14.158 | 13.158 | -20.2 | 12.928 | 0.129 | -46.682 | 6.282 | 5.294 | 8.155 | 0.519 | -11.111 | 21.7 |
|  | Placebo | 1 | 2 | -8.467 | 5.875 | 0.154 | -20.163 | 3.229 | 0.182 | 8.716 | 0.984 | -17.672 | 18.036 | -13.474 | 7.714 | 0.087 | -28.992 | 2.044 |
|  |  | 2 | 1 | 8.467 | 5.875 | 0.154 | -3.229 | 20.163 | -0.182 | 8.716 | 0.984 | -18.036 | 17.672 | 13.474 | 7.714 | 0.087 | -2.044 | 28.992 |
|  | 5mg | 1 | 2 | 2.138 | 5.975 | 0.721 | -9.758 | 14.034 | -1.533 | 7.464 | 0.839 | -16.822 | 13.756 | 6.071 | 8.986 | 0.503 | -12.007 | 24.149 |
|  |  | 2 | 1 | -2.138 | 5.975 | 0.721 | -14.034 | 9.758 | 1.533 | 7.464 | 0.839 | -13.756 | 16.822 | -6.071 | 8.986 | 0.503 | -24.149 | 12.007 |
| Immune Readiness | 10mg | 1 | 2 | 1.273 | 2.402 | 0.598 | -3.51 | 6.056 | 0.6 | 5.289 | 0.91 | -10.235 | 11.435 | 1.471 | 2.718 | 0.591 | -3.997 | 6.938 |
|  |  | 2 | 1 | -1.273 | 2.402 | 0.598 | -6.056 | 3.51 | -0.6 | 5.289 | 0.91 | -11.435 | 10.235 | -1.471 | 2.718 | 0.591 | -6.938 | 3.997 |
|  | Placebo | 1 | 2 | 1.667 | 2.057 | 0.42 | -2.429 | 5.762 | 2 | 3.566 | 0.579 | -5.305 | 9.305 | 1.474 | 2.571 | 0.569 | -3.698 | 6.645 |
|  |  | 2 | 1 | -1.667 | 2.057 | 0.42 | -5.762 | 2.429 | -2 | 3.566 | 0.579 | -9.305 | 5.305 | -1.474 | 2.571 | 0.569 | -6.645 | 3.698 |
|  | 5mg | 1 | 2 | 0.207 | 2.092 | 0.921 | -3.959 | 4.373 | -1.4 | 3.054 | 0.65 | -7.655 | 4.855 | 1.929 | 2.995 | 0.523 | -4.096 | 7.954 |
|  |  | 2 | 1 | -0.207 | 2.092 | 0.921 | -4.373 | 3.959 | 1.4 | 3.054 | 0.65 | -4.855 | 7.655 | -1.929 | 2.995 | 0.523 | -7.954 | 4.096 |

| **Repeated Measures ANOVA** | | | | | | |  |  |  |  |  |  |  |  |  |  |
| --- | --- | --- | --- | --- | --- | --- | --- | --- | --- | --- | --- | --- | --- | --- | --- | --- |
| All Genders |  |  |  |  |  |  | Females |  |  |  |  | Males |  |  |  |  |
| Group | Measure | df 1 | df 2 | F | p-value | Partial Eta Squared | df 1 | df 2 | F | p-value | Partial Eta Squared | df 1 | df 2 | F | p-value | Partial Eta Squared |
| 10mg | Digestion | 1 | 21 | 0.005 | 0.945 | 0 | 1 | 4 | 0.02 | 0.894 | 0.005 | 1 | 18 | 0.001 | 0.977 | 0 |
|  | Inflammation | 1 | 21 | 1.104 | 0.305 | 0.05 | 1 | 4 | 3.201 | 0.148 | 0.445 | 1 | 18 | 0.119 | 0.735 | 0.007 |
|  | Gut Dysbiosis | 1 | 21 | 2.514 | 0.128 | 0.107 | 1 | 4 | 0.069 | 0.806 | 0.017 | 1 | 18 | 4.729 | 0.045 | 0.228 |
|  | Intestinal Permeability | 1 | 21 | 0.12 | 0.733 | 0.006 | 1 | 4 | 6.641 | 0.062 | 0.624 | 1 | 18 | 0.02 | 0.889 | 0.001 |
|  | Nervous System | 1 | 21 | 0.022 | 0.884 | 0.001 | 1 | 4 | 1 | 0.374 | 0.2 | 1 | 18 | 0.005 | 0.942 | 0 |
|  | Diversity | 1 | 21 | 0.006 | 0.94 | 0 | 1 | 4 | 2.125 | 0.219 | 0.347 | 1 | 18 | 0.558 | 0.466 | 0.034 |
|  | Immune Readiness | 1 | 21 | 0.933 | 0.345 | 0.043 | 1 | 4 | 0.096 | 0.772 | 0.023 | 1 | 18 | 0.812 | 0.381 | 0.048 |
| Placebo | Digestion | 1 | 29 | 0.001 | 0.973 | 0 | 1 | 10 | 0.033 | 0.859 | 0.003 | 1 | 18 | 0.024 | 0.878 | 0.001 |
|  | Inflammation | 1 | 29 | 3.911 | 0.058 | 0.119 | 1 | 10 | 0.723 | 0.415 | 0.067 | 1 | 18 | 4.042 | 0.06 | 0.183 |
|  | Gut Dysbiosis | 1 | 29 | 0.144 | 0.707 | 0.005 | 1 | 10 | 0.024 | 0.879 | 0.002 | 1 | 18 | 0.138 | 0.714 | 0.008 |
|  | Intestinal Permeability | 1 | 29 | 2.215 | 0.147 | 0.071 | 1 | 10 | 0.3 | 0.596 | 0.029 | 1 | 18 | 2.676 | 0.119 | 0.129 |
|  | Nervous System | 1 | 29 | 1.908 | 0.178 | 0.062 | 1 | 10 | 0.333 | 0.577 | 0.032 | 1 | 18 | 1.54 | 0.231 | 0.079 |
|  | Diversity | 1 | 29 | 2.115 | 0.157 | 0.068 | 1 | 10 | 0.001 | 0.981 | 0 | 1 | 18 | 2.824 | 0.11 | 0.136 |
|  | Immune Readiness | 1 | 29 | 0.501 | 0.485 | 0.017 | 1 | 10 | 0.234 | 0.639 | 0.023 | 1 | 18 | 0.253 | 0.621 | 0.014 |
| 5mg | Digestion | 1 | 28 | 3.227 | 0.083 | 0.103 | 1 | 14 | 2.339 | 0.148 | 0.143 | 1 | 13 | 1.121 | 0.309 | 0.079 |
|  | Inflammation | 1 | 28 | 1.418 | 0.244 | 0.048 | 1 | 14 | 1.047 | 0.324 | 0.07 | 1 | 13 | 0.375 | 0.551 | 0.028 |
|  | Gut Dysbiosis | 1 | 28 | 0.037 | 0.85 | 0.001 | 1 | 14 | 0.011 | 0.918 | 0.001 | 1 | 13 | 0.032 | 0.861 | 0.002 |
|  | Intestinal Permeability | 1 | 28 | 0.766 | 0.389 | 0.027 | 1 | 14 | 0.382 | 0.546 | 0.027 | 1 | 13 | 0.365 | 0.556 | 0.027 |
|  | Nervous System | 1 | 28 | 1.105 | 0.302 | 0.038 | 1 | 14 | 0.037 | 0.851 | 0.003 | 1 | 13 | 1.825 | 0.2 | 0.123 |
|  | Diversity | 1 | 28 | 0.119 | 0.733 | 0.004 | 1 | 14 | 0.037 | 0.85 | 0.003 | 1 | 13 | 0.384 | 0.546 | 0.029 |
|  | Immune Readiness | 1 | 28 | 0.008 | 0.929 | 0 | 1 | 14 | 0.21 | 0.654 | 0.015 | 1 | 13 | 0.302 | 0.592 | 0.023 |

| **Pairwise Comparisons** | | | | | |  |  |  |  |  |  |  |  |  |  |  |  |  |
| --- | --- | --- | --- | --- | --- | --- | --- | --- | --- | --- | --- | --- | --- | --- | --- | --- | --- | --- |
|  |  |  |  | All Genders | | | | | Females | | | | | Males | | | | |
| Group | Measure | Time 1 | Time 2 | Mean Difference | Std. Error | p-value | 95% Confidence Interval |  | Mean Difference | Std. Error | p-value | 95% Confidence Interval |  | Mean Difference | Std. Error | p-value | 95% Confidence Interval |  |
|  |  |  |  |  |  |  | Lower Bound | Upper Bound |  |  |  | Lower Bound | Upper Bound |  |  |  | Lower Bound | Upper Bound |
| 10mg | Digestion | 1 | 2 | -0.145 | 2.095 | 0.945 | -4.502 | 4.211 | -0.86 | 6.055 | 0.894 | -17.672 | 15.952 | 0.065 | 2.179 | 0.977 | -4.554 | 4.683 |
|  |  | 2 | 1 | 0.145 | 2.095 | 0.945 | -4.211 | 4.502 | 0.86 | 6.055 | 0.894 | -15.952 | 17.672 | -0.065 | 2.179 | 0.977 | -4.683 | 4.554 |
|  | Inflammation | 1 | 2 | -1.35 | 1.285 | 0.305 | -4.022 | 1.322 | -4.2 | 2.347 | 0.148 | -10.717 | 2.317 | -0.512 | 1.485 | 0.735 | -3.661 | 2.637 |
|  |  | 2 | 1 | 1.35 | 1.285 | 0.305 | -1.322 | 4.022 | 4.2 | 2.347 | 0.148 | -2.317 | 10.717 | 0.512 | 1.485 | 0.735 | -2.637 | 3.661 |
|  | Gut Dysbiosis | 1 | 2 | -1.573 | 0.992 | 0.128 | -3.636 | 0.49 | 0.68 | 2.592 | 0.806 | -6.517 | 7.877 | -2.235* | 1.028 | 0.045 | -4.414 | -0.056 |
|  |  | 2 | 1 | 1.573 | 0.992 | 0.128 | -0.49 | 3.636 | -0.68 | 2.592 | 0.806 | -7.877 | 6.517 | 2.235* | 1.028 | 0.045 | 0.056 | 4.414 |
|  | Intestinal Permeability | 1 | 2 | -0.491 | 1.418 | 0.733 | -3.439 | 2.457 | -3.02 | 1.172 | 0.062 | -6.274 | 0.234 | 0.253 | 1.778 | 0.889 | -3.516 | 4.022 |
|  |  | 2 | 1 | 0.491 | 1.418 | 0.733 | -2.457 | 3.439 | 3.02 | 1.172 | 0.062 | -0.234 | 6.274 | -0.253 | 1.778 | 0.889 | -4.022 | 3.516 |
|  | Nervous System | 1 | 2 | -0.091 | 0.613 | 0.884 | -1.366 | 1.185 | -0.2 | 0.2 | 0.374 | -0.755 | 0.355 | -0.059 | 0.797 | 0.942 | -1.749 | 1.632 |
|  |  | 2 | 1 | 0.091 | 0.613 | 0.884 | -1.185 | 1.366 | 0.2 | 0.2 | 0.374 | -0.355 | 0.755 | 0.059 | 0.797 | 0.942 | -1.632 | 1.749 |
|  | Diversity | 1 | 2 | 0.5 | 6.581 | 0.94 | -13.187 | 14.187 | 20.2 | 13.858 | 0.219 | -18.276 | 58.676 | -5.294 | 7.087 | 0.466 | -20.317 | 9.729 |
|  |  | 2 | 1 | -0.5 | 6.581 | 0.94 | -14.187 | 13.187 | -20.2 | 13.858 | 0.219 | -58.676 | 18.276 | 5.294 | 7.087 | 0.466 | -9.729 | 20.317 |
|  | Immune Readiness | 1 | 2 | 1.273 | 1.318 | 0.345 | -1.468 | 4.013 | 0.6 | 1.939 | 0.772 | -4.784 | 5.984 | 1.471 | 1.632 | 0.381 | -1.989 | 4.93 |
|  |  | 2 | 1 | -1.273 | 1.318 | 0.345 | -4.013 | 1.468 | -0.6 | 1.939 | 0.772 | -5.984 | 4.784 | -1.471 | 1.632 | 0.381 | -4.93 | 1.989 |
| Placebo | Digestion | 1 | 2 | 0.073 | 2.118 | 0.973 | -4.258 | 4.405 | -0.573 | 3.146 | 0.859 | -7.582 | 6.437 | 0.447 | 2.864 | 0.878 | -5.571 | 6.465 |
|  |  | 2 | 1 | -0.073 | 2.118 | 0.973 | -4.405 | 4.258 | 0.573 | 3.146 | 0.859 | -6.437 | 7.582 | -0.447 | 2.864 | 0.878 | -6.465 | 5.571 |
|  | Inflammation | 1 | 2 | -4.267 | 2.157 | 0.058 | -8.679 | 0.146 | -3.945 | 4.642 | 0.415 | -14.287 | 6.397 | -4.453 | 2.215 | 0.06 | -9.106 | 0.2 |
|  |  | 2 | 1 | 4.267 | 2.157 | 0.058 | -0.146 | 8.679 | 3.945 | 4.642 | 0.415 | -6.397 | 14.287 | 4.453 | 2.215 | 0.06 | -0.2 | 9.106 |
|  | Gut Dysbiosis | 1 | 2 | -0.627 | 1.65 | 0.707 | -4.001 | 2.748 | -0.509 | 3.267 | 0.879 | -7.788 | 6.77 | -0.695 | 1.869 | 0.714 | -4.621 | 3.231 |
|  |  | 2 | 1 | 0.627 | 1.65 | 0.707 | -2.748 | 4.001 | 0.509 | 3.267 | 0.879 | -6.77 | 7.788 | 0.695 | 1.869 | 0.714 | -3.231 | 4.621 |
|  | Intestinal Permeability | 1 | 2 | -4.227 | 2.84 | 0.147 | -10.035 | 1.581 | -3.364 | 6.141 | 0.596 | -17.046 | 10.319 | -4.726 | 2.889 | 0.119 | -10.796 | 1.344 |
|  |  | 2 | 1 | 4.227 | 2.84 | 0.147 | -1.581 | 10.035 | 3.364 | 6.141 | 0.596 | -10.319 | 17.046 | 4.726 | 2.889 | 0.119 | -1.344 | 10.796 |
|  | Nervous System | 1 | 2 | 2.933 | 2.124 | 0.178 | -1.41 | 7.277 | 1.545 | 2.678 | 0.577 | -4.421 | 7.512 | 3.737 | 3.012 | 0.231 | -2.59 | 10.064 |
|  |  | 2 | 1 | -2.933 | 2.124 | 0.178 | -7.277 | 1.41 | -1.545 | 2.678 | 0.577 | -7.512 | 4.421 | -3.737 | 3.012 | 0.231 | -10.064 | 2.59 |
|  | Diversity | 1 | 2 | -8.467 | 5.822 | 0.157 | -20.375 | 3.442 | 0.182 | 7.515 | 0.981 | -16.562 | 16.925 | -13.474 | 8.017 | 0.11 | -30.317 | 3.37 |
|  |  | 2 | 1 | 8.467 | 5.822 | 0.157 | -3.442 | 20.375 | -0.182 | 7.515 | 0.981 | -16.925 | 16.562 | 13.474 | 8.017 | 0.11 | -3.37 | 30.317 |
|  | Immune Readiness | 1 | 2 | 1.667 | 2.355 | 0.485 | -3.15 | 6.483 | 2 | 4.139 | 0.639 | -7.221 | 11.221 | 1.474 | 2.931 | 0.621 | -4.685 | 7.632 |
|  |  | 2 | 1 | -1.667 | 2.355 | 0.485 | -6.483 | 3.15 | -2 | 4.139 | 0.639 | -11.221 | 7.221 | -1.474 | 2.931 | 0.621 | -7.632 | 4.685 |
| 5mg | Digestion | 1 | 2 | -2.593 | 1.444 | 0.083 | -5.55 | 0.364 | -2.6 | 1.7 | 0.148 | -6.246 | 1.046 | -2.586 | 2.442 | 0.309 | -7.861 | 2.69 |
|  |  | 2 | 1 | 2.593 | 1.444 | 0.083 | -0.364 | 5.55 | 2.6 | 1.7 | 0.148 | -1.046 | 6.246 | 2.586 | 2.442 | 0.309 | -2.69 | 7.861 |
|  | Inflammation | 1 | 2 | -3.383 | 2.841 | 0.244 | -9.203 | 2.437 | -4.267 | 4.17 | 0.324 | -13.211 | 4.677 | -2.436 | 3.977 | 0.551 | -11.027 | 6.156 |
|  |  | 2 | 1 | 3.383 | 2.841 | 0.244 | -2.437 | 9.203 | 4.267 | 4.17 | 0.324 | -4.677 | 13.211 | 2.436 | 3.977 | 0.551 | -6.156 | 11.027 |
|  | Gut Dysbiosis | 1 | 2 | -0.303 | 1.588 | 0.85 | -3.556 | 2.949 | -0.267 | 2.559 | 0.918 | -5.755 | 5.222 | -0.343 | 1.922 | 0.861 | -4.495 | 3.809 |
|  |  | 2 | 1 | 0.303 | 1.588 | 0.85 | -2.949 | 3.556 | 0.267 | 2.559 | 0.918 | -5.222 | 5.755 | 0.343 | 1.922 | 0.861 | -3.809 | 4.495 |
|  | Intestinal Permeability | 1 | 2 | -3.286 | 3.756 | 0.389 | -10.98 | 4.408 | -3.593 | 5.813 | 0.546 | -16.061 | 8.874 | -2.957 | 4.892 | 0.556 | -13.526 | 7.612 |
|  |  | 2 | 1 | 3.286 | 3.756 | 0.389 | -4.408 | 10.98 | 3.593 | 5.813 | 0.546 | -8.874 | 16.061 | 2.957 | 4.892 | 0.556 | -7.612 | 13.526 |
|  | Nervous System | 1 | 2 | 1.517 | 1.443 | 0.302 | -1.439 | 4.473 | 0.4 | 2.09 | 0.851 | -4.083 | 4.883 | 2.714 | 2.009 | 0.2 | -1.627 | 7.055 |
|  |  | 2 | 1 | -1.517 | 1.443 | 0.302 | -4.473 | 1.439 | -0.4 | 2.09 | 0.851 | -4.883 | 4.083 | -2.714 | 2.009 | 0.2 | -7.055 | 1.627 |
|  | Diversity | 1 | 2 | 2.138 | 6.204 | 0.733 | -10.571 | 14.847 | -1.533 | 7.972 | 0.85 | -18.631 | 15.565 | 6.071 | 9.804 | 0.546 | -15.108 | 27.251 |
|  |  | 2 | 1 | -2.138 | 6.204 | 0.733 | -14.847 | 10.571 | 1.533 | 7.972 | 0.85 | -15.565 | 18.631 | -6.071 | 9.804 | 0.546 | -27.251 | 15.108 |
|  | Immune Readiness | 1 | 2 | 0.207 | 2.295 | 0.929 | -4.494 | 4.908 | -1.4 | 3.053 | 0.654 | -7.948 | 5.148 | 1.929 | 3.507 | 0.592 | -5.649 | 9.506 |
|  |  | 2 | 1 | -0.207 | 2.295 | 0.929 | -4.908 | 4.494 | 1.4 | 3.053 | 0.654 | -5.148 | 7.948 | -1.929 | 3.507 | 0.592 | -9.506 | 5.649 |

| **Supplementary Table 9. Analysis of SF-36 self-reported measures of well-being** | | | | | | | | | | |
| --- | --- | --- | --- | --- | --- | --- | --- | --- | --- | --- |
|  |  |  |  |  |  |  |  |  |  |  |
| **Means and Standard Deviations** |  | All Genders |  |  | Females |  |  | Males |  |  |
|  | Group | Mean | Std. Deviation | N | Mean | Std. Deviation | N | Mean | Std. Deviation | N |
| Physical Functioning week 0 | 10mg | 97.097 | 3.3601 | 31 | 95.625 | 4.1726 | 8 | 97.609 | 2.9655 | 23 |
|  | Placebo | 95 | 8.0943 | 30 | 95 | 5.7735 | 13 | 95 | 9.6825 | 17 |
|  | 5mg | 94.559 | 7.8207 | 34 | 95 | 6.5465 | 15 | 94.211 | 8.8605 | 19 |
|  | Total | 95.526 | 6.8212 | 95 | 95.139 | 5.6677 | 36 | 95.763 | 7.4749 | 59 |
| Physical Functioning week 24 | 10mg | 95.968 | 8.002 | 31 | 95.625 | 5.63 | 8 | 96.087 | 8.7849 | 23 |
|  | Placebo | 94 | 8.8474 | 30 | 93.462 | 9.4394 | 13 | 94.412 | 8.639 | 17 |
|  | 5mg | 95 | 7.9772 | 34 | 95.667 | 5.6273 | 15 | 94.474 | 9.559 | 19 |
|  | Total | 95 | 8.2191 | 95 | 94.861 | 7.12 | 36 | 95.085 | 8.881 | 59 |
| Physical Functioning week 48 | 10mg | 95.806 | 6.8431 | 31 | 95.625 | 5.63 | 8 | 95.87 | 7.3318 | 23 |
|  | Placebo | 94.667 | 8.8992 | 30 | 95.385 | 5.5758 | 13 | 94.118 | 10.9309 | 17 |
|  | 5mg | 95 | 8.9612 | 34 | 96 | 6.3246 | 15 | 94.211 | 10.7061 | 19 |
|  | Total | 95.158 | 8.2337 | 95 | 95.694 | 5.7511 | 36 | 94.831 | 9.4671 | 59 |
| Role limitations due to physical health week 0 | 10mg | 95.968 | 9.347 | 31 | 93.75 | 11.5728 | 8 | 96.739 | 8.6088 | 23 |
|  | Placebo | 88.333 | 27.6472 | 30 | 86.538 | 29.9572 | 13 | 89.706 | 26.603 | 17 |
|  | 5mg | 92.647 | 19.971 | 34 | 91.667 | 15.4303 | 15 | 93.421 | 23.3365 | 19 |
|  | Total | 92.368 | 20.3277 | 95 | 90.278 | 20.9402 | 36 | 93.644 | 20.0179 | 59 |
| Role limitations due to physical health week 24 | 10mg | 92.742 | 22.5403 | 31 | 100 | 0 | 8 | 90.217 | 25.8263 | 23 |
|  | Placebo | 94.167 | 20.43 | 30 | 100 | 0 | 13 | 89.706 | 26.603 | 17 |
|  | 5mg | 94.853 | 13.455 | 34 | 91.667 | 18.0937 | 15 | 97.368 | 7.8825 | 19 |
|  | Total | 93.947 | 18.8484 | 95 | 96.528 | 12.1784 | 36 | 92.373 | 21.9002 | 59 |
| Role limitations due to physical health week 48 | 10mg | 93.548 | 20.3795 | 31 | 96.875 | 8.8388 | 8 | 92.391 | 23.1531 | 23 |
|  | Placebo | 90.833 | 23.1964 | 30 | 92.308 | 21.3713 | 13 | 89.706 | 25.0917 | 17 |
|  | 5mg | 92.647 | 15.7266 | 34 | 95 | 10.351 | 15 | 90.789 | 19.0221 | 19 |
|  | Total | 92.368 | 19.6626 | 95 | 94.444 | 14.7734 | 36 | 91.102 | 22.1489 | 59 |
| Role limitations due to emotional problems week 0 | 10mg | 97.852 | 8.316 | 31 | 100 | 0 | 8 | 97.104 | 9.5939 | 23 |
|  | Placebo | 93.337 | 20.3374 | 30 | 97.438 | 9.2358 | 13 | 90.2 | 25.721 | 17 |
|  | 5mg | 89.212 | 24.2438 | 34 | 91.107 | 23.4694 | 15 | 87.716 | 25.3732 | 19 |
|  | Total | 93.334 | 19.2066 | 95 | 95.369 | 16.2435 | 36 | 92.092 | 20.8447 | 59 |
| Role limitations due to emotional problems week 24 | 10mg | 95.7 | 18.7401 | 31 | 100 | 0 | 8 | 94.204 | 21.6756 | 23 |
|  | Placebo | 96.67 | 10.1608 | 30 | 97.438 | 9.2358 | 13 | 96.082 | 11.0591 | 17 |
|  | 5mg | 94.121 | 19.1892 | 34 | 95.56 | 11.7171 | 15 | 92.984 | 23.774 | 19 |
|  | Total | 95.441 | 16.5631 | 95 | 97.225 | 9.3342 | 36 | 94.353 | 19.7195 | 59 |
| Role limitations due to emotional problems week 48 | 10mg | 96.777 | 10.0079 | 31 | 95.838 | 11.7733 | 8 | 97.104 | 9.5939 | 23 |
|  | Placebo | 100 | 0 | 30 | 100 | 0 | 13 | 100 | 0 | 17 |
|  | 5mg | 96.076 | 15.9301 | 34 | 95.553 | 17.2219 | 15 | 96.489 | 15.302 | 19 |
|  | Total | 97.544 | 11.1334 | 95 | 97.222 | 12.2824 | 36 | 97.741 | 10.4762 | 59 |
| Energy/Fatigue week 0 | 10mg | 72.903 | 13.7704 | 31 | 65 | 15.353 | 8 | 75.652 | 12.3679 | 23 |
|  | Placebo | 68.333 | 17.3371 | 30 | 69.615 | 21.2585 | 13 | 67.353 | 14.2651 | 17 |
|  | 5mg | 65 | 19.2275 | 34 | 60 | 21.63 | 15 | 68.947 | 16.6315 | 19 |
|  | Total | 68.632 | 17.1421 | 95 | 64.583 | 20.1911 | 36 | 71.102 | 14.6237 | 59 |
| Energy/Fatigue week 24 | 10mg | 75.161 | 15.1923 | 31 | 70.625 | 11.4759 | 8 | 76.739 | 16.2095 | 23 |
|  | Placebo | 70.667 | 15.9597 | 30 | 76.538 | 15.4629 | 13 | 66.176 | 15.2612 | 17 |
|  | 5mg | 67.206 | 14.6262 | 34 | 65.333 | 13.2916 | 15 | 68.684 | 15.7975 | 19 |
|  | Total | 70.895 | 15.4365 | 95 | 70.556 | 14.2817 | 36 | 71.102 | 16.217 | 59 |
| Energy/Fatigue week 48 | 10mg | 77.419 | 11.3189 | 31 | 74.375 | 11.4759 | 8 | 78.478 | 11.3252 | 23 |
|  | Placebo | 70.5 | 15.1629 | 30 | 73.462 | 15.9928 | 13 | 68.235 | 14.5711 | 17 |
|  | 5mg | 70.294 | 12.9651 | 34 | 69.333 | 13.7408 | 15 | 71.053 | 12.6468 | 19 |
|  | Total | 72.684 | 13.4838 | 95 | 71.944 | 13.9529 | 36 | 73.136 | 13.2905 | 59 |
| Emotional Wellbeing week 0 | 10mg | 84.645 | 10.333 | 31 | 77.5 | 12.6378 | 8 | 87.13 | 8.3519 | 23 |
|  | Placebo | 81.067 | 11.3105 | 30 | 82.769 | 9.7139 | 13 | 79.765 | 12.5276 | 17 |
|  | 5mg | 76.706 | 14.4883 | 34 | 76.267 | 14.0584 | 15 | 77.053 | 15.1931 | 19 |
|  | Total | 80.674 | 12.5769 | 95 | 78.889 | 12.3422 | 36 | 81.763 | 12.6985 | 59 |
| Emotional Wellbeing week 24 | 10mg | 86.581 | 8.9769 | 31 | 80 | 11.9044 | 8 | 88.87 | 6.601 | 23 |
|  | Placebo | 83.6 | 9.1787 | 30 | 85.846 | 7.0456 | 13 | 81.882 | 10.4036 | 17 |
|  | 5mg | 81.176 | 12.5926 | 34 | 83.2 | 9.4657 | 15 | 79.579 | 14.6603 | 19 |
|  | Total | 83.705 | 10.603 | 95 | 83.444 | 9.2781 | 36 | 83.864 | 11.41 | 59 |
| Emotional Wellbeing week 48 | 10mg | 86.581 | 8.4253 | 31 | 82.5 | 9.5469 | 8 | 88 | 7.7225 | 23 |
|  | Placebo | 85.333 | 9.9839 | 30 | 88 | 8 | 13 | 83.294 | 11.0666 | 17 |
|  | 5mg | 81.882 | 11.6196 | 34 | 80.8 | 12.1137 | 15 | 82.737 | 11.4738 | 19 |
|  | Total | 84.505 | 10.2437 | 95 | 83.778 | 10.472 | 36 | 84.949 | 10.1666 | 59 |
| Social Functioning week 0 | 10mg | 93.145 | 16.4059 | 31 | 82.813 | 28.2981 | 8 | 96.739 | 7.7399 | 23 |
|  | Placebo | 94.167 | 10.7546 | 30 | 93.269 | 10.9632 | 13 | 94.853 | 10.8783 | 17 |
|  | 5mg | 92.279 | 14.1068 | 34 | 91.667 | 12.1988 | 15 | 92.763 | 15.7651 | 19 |
|  | Total | 93.158 | 13.8582 | 95 | 90.278 | 16.6667 | 36 | 94.915 | 11.6335 | 59 |
| Social Functioning week 24 | 10mg | 94.355 | 14.0084 | 31 | 90.625 | 14.5621 | 8 | 95.652 | 13.9034 | 23 |
|  | Placebo | 96.667 | 7.996 | 30 | 98.077 | 4.6942 | 13 | 95.588 | 9.8238 | 17 |
|  | 5mg | 95.221 | 13.4188 | 34 | 97.5 | 7.0076 | 15 | 93.421 | 16.8575 | 19 |
|  | Total | 95.395 | 12.1017 | 95 | 96.181 | 8.8738 | 36 | 94.915 | 13.7556 | 59 |
| Social Functioning week 48 | 10mg | 97.177 | 8.3602 | 31 | 95.313 | 9.3003 | 8 | 97.826 | 8.1291 | 23 |
|  | Placebo | 96.25 | 7.4495 | 30 | 95.192 | 8.1305 | 13 | 97.059 | 7.0287 | 17 |
|  | 5mg | 95.588 | 8.6396 | 34 | 95.833 | 9.0468 | 15 | 95.395 | 8.5498 | 19 |
|  | Total | 96.316 | 8.1283 | 95 | 95.486 | 8.5319 | 36 | 96.822 | 7.9034 | 59 |
| Pain week 0 | 10mg | 84.597 | 15.112 | 31 | 72.5 | 20.831 | 8 | 88.804 | 10.0812 | 23 |
|  | Placebo | 81.75 | 16.9196 | 30 | 84.615 | 16.4838 | 13 | 79.559 | 17.4158 | 17 |
|  | 5mg | 84.853 | 12.9091 | 34 | 84.167 | 9.8501 | 15 | 85.395 | 15.1443 | 19 |
|  | Total | 83.789 | 14.8882 | 95 | 81.736 | 15.6161 | 36 | 85.042 | 14.4175 | 59 |
| Pain week 24 | 10mg | 86.613 | 12.8379 | 31 | 90 | 7.5593 | 8 | 85.435 | 14.1753 | 23 |
|  | Placebo | 82.833 | 16.2514 | 30 | 85.577 | 14.6541 | 13 | 80.735 | 17.5171 | 17 |
|  | 5mg | 85.515 | 14.7553 | 34 | 86 | 15.2304 | 15 | 85.132 | 14.7778 | 19 |
|  | Total | 85.026 | 14.5933 | 95 | 86.736 | 13.4539 | 36 | 83.983 | 15.2643 | 59 |
| Pain week 48 | 10mg | 87.661 | 12.075 | 31 | 87.188 | 12.4955 | 8 | 87.826 | 12.2081 | 23 |
|  | Placebo | 83.833 | 16.4229 | 30 | 87.308 | 12.4775 | 13 | 81.176 | 18.8356 | 17 |
|  | 5mg | 88.456 | 12.9541 | 34 | 91 | 9.8107 | 15 | 86.447 | 14.9377 | 19 |
|  | Total | 86.737 | 13.8815 | 95 | 88.819 | 11.2518 | 36 | 85.466 | 15.2174 | 59 |
| General Health week 0 | 10mg | 83.548 | 16.4415 | 31 | 75 | 19.0863 | 8 | 86.522 | 14.7274 | 23 |
|  | Placebo | 75.333 | 20.4658 | 30 | 76.923 | 21.6543 | 13 | 74.118 | 20.0963 | 17 |
|  | 5mg | 75.588 | 17.9547 | 34 | 76.333 | 20.5693 | 15 | 75 | 16.1589 | 19 |
|  | Total | 78.105 | 18.5251 | 95 | 76.25 | 20.0846 | 36 | 79.237 | 17.5876 | 59 |
| General Health week 24 | 10mg | 85 | 11.8322 | 31 | 80 | 12.5357 | 8 | 86.739 | 11.3427 | 23 |
|  | Placebo | 78.333 | 18.0198 | 30 | 81.923 | 18.4321 | 13 | 75.588 | 17.7555 | 17 |
|  | 5mg | 81.471 | 12.6449 | 34 | 84.333 | 13.0749 | 15 | 79.211 | 12.1636 | 19 |
|  | Total | 81.632 | 14.4296 | 95 | 82.5 | 14.8083 | 36 | 81.102 | 14.2958 | 59 |
| General Health week 48 | 10mg | 86.613 | 10.9839 | 31 | 80.625 | 10.8356 | 8 | 88.696 | 10.4683 | 23 |
|  | Placebo | 76.167 | 16.7478 | 30 | 81.154 | 14.7414 | 13 | 72.353 | 17.5995 | 17 |
|  | 5mg | 81.471 | 14.0092 | 34 | 81.667 | 15.9985 | 15 | 81.316 | 12.6757 | 19 |
|  | Total | 81.474 | 14.5477 | 95 | 81.25 | 14.1611 | 36 | 81.61 | 14.8974 | 59 |
| *df = degrees of freedom, provided as: between groups, within groups* | | | | | | | | | | |
| *^denotes use of Welch's ANOVA in instances that lack homogeneity of variances* | | | | | | | | | | |
| * *p* ≤ 0.05 |  |  |  |  |  |  |  |  |  |  |
| ***effect size provided as epsilon squared for ANOVA or omega squared for Welch's ANOVA* | | | | | | | | | | |

| **Repeated Measures Mixed ANOVA** | | | |  |  |  |  |
| --- | --- | --- | --- | --- | --- | --- | --- |
| All Genders |  |  |  |  |  |  |  |
|  | df 1 | df 2 | F | p value | Partial Eta Squared |  |  |
| Physical Function^ | 3.582 | 164.794 | 0.554 | 0.677 | 0.012 |  |  |
| Role limitations due to physical health^ | 3.743 | 172.194 | 0.584 | 0.663 | 0.013 |  |  |
| Role limitations due to emotional problems^ | 3.328 | 153.103 | 0.948 | 0.426 | 0.02 |  |  |
| Energy/Fatigue | 4 | 184 | 0.393 | 0.814 | 0.008 |  |  |
| Emotional Wellbeing | 4 | 184 | 0.685 | 0.603 | 0.015 |  |  |
| Social Functioning^ | 3.688 | 169.626 | 0.259 | 0.891 | 0.006 |  |  |
| Pain | 4 | 184 | 0.143 | 0.966 | 0.003 |  |  |
| General Health^ | 3.42 | 157.342 | 1.511 | 0.209 | 0.032 |  |  |
|  |  |  |  |  |  |  |  |
|  |  |  |  |  |  |  |  |
| **Pairwise Comparisons** |  |  |  |  |  |  |  |
|  |  |  | All Genders | | | | |
| Group | Time 1 | Time 2 | Mean Difference | Std. Error | p value | 95% Confidence Interval for Differencea |  |
|  |  |  |  |  |  | Lower Bound | Upper Bound |
| Physical Function | Baseline | 24 weeks | 0.563 | 0.589 | 1 | -0.874 | 1.999 |
|  |  | 48 weeks | 0.394 | 0.674 | 1 | -1.25 | 2.039 |
|  | 24 weeks | Baseline | -0.563 | 0.589 | 1 | -1.999 | 0.874 |
|  |  | 48 weeks | -0.168 | 0.498 | 1 | -1.383 | 1.046 |
|  | 48 weeks | Baseline | -0.394 | 0.674 | 1 | -2.039 | 1.25 |
|  |  | 24 weeks | 0.168 | 0.498 | 1 | -1.046 | 1.383 |
| Role limitations due to physical health | Baseline | 24 weeks | -1.604 | 2.067 | 1 | -6.645 | 3.436 |
|  |  | 48 weeks | -0.027 | 2.56 | 1 | -6.27 | 6.217 |
|  | 24 weeks | Baseline | 1.604 | 2.067 | 1 | -3.436 | 6.645 |
|  |  | 48 weeks | 1.578 | 2.557 | 1 | -4.658 | 7.813 |
|  | 48 weeks | Baseline | 0.027 | 2.56 | 1 | -6.217 | 6.27 |
|  |  | 24 weeks | -1.578 | 2.557 | 1 | -7.813 | 4.658 |
| Role limitations due to emotional problems | Baseline | 24 weeks | -2.03 | 2.391 | 1 | -7.861 | 3.8 |
|  |  | 48 weeks | -4.151 | 2.061 | 0.141 | -9.178 | 0.876 |
|  | 24 weeks | Baseline | 2.03 | 2.391 | 1 | -3.8 | 7.861 |
|  |  | 48 weeks | -2.121 | 1.578 | 0.547 | -5.969 | 1.727 |
|  | 48 weeks | Baseline | 4.151 | 2.061 | 0.141 | -0.876 | 9.178 |
|  |  | 24 weeks | 2.121 | 1.578 | 0.547 | -1.727 | 5.969 |
| Energy/Fatigue | Baseline | 24 weeks | -2.266 | 1.309 | 0.261 | -5.458 | 0.927 |
|  |  | 48 weeks | -3.992* | 1.288 | 0.008 | -7.132 | -0.853 |
|  | 24 weeks | Baseline | 2.266 | 1.309 | 0.261 | -0.927 | 5.458 |
|  |  | 48 weeks | -1.727 | 1.124 | 0.384 | -4.468 | 1.015 |
|  | 48 weeks | Baseline | 3.992* | 1.288 | 0.008 | 0.853 | 7.132 |
|  |  | 24 weeks | 1.727 | 1.124 | 0.384 | -1.015 | 4.468 |
| Emotional Wellbeing | Baseline | 24 weeks | -2.980* | 0.979 | 0.009 | -5.367 | -0.592 |
|  |  | 48 weeks | -3.793* | 0.964 | <.001 | -6.145 | -1.441 |
|  | 24 weeks | Baseline | 2.980* | 0.979 | 0.009 | 0.592 | 5.367 |
|  |  | 48 weeks | -0.813 | 0.855 | 1 | -2.898 | 1.272 |
|  | 48 weeks | Baseline | 3.793* | 0.964 | <.001 | 1.441 | 6.145 |
|  |  | 24 weeks | 0.813 | 0.855 | 1 | -1.272 | 2.898 |
| Social Functioning | Baseline | 24 weeks | -2.217 | 1.548 | 0.466 | -5.991 | 1.557 |
|  |  | 48 weeks | -3.141 | 1.427 | 0.091 | -6.621 | 0.338 |
|  | 24 weeks | Baseline | 2.217 | 1.548 | 0.466 | -1.557 | 5.991 |
|  |  | 48 weeks | -0.925 | 1.193 | 1 | -3.833 | 1.984 |
|  | 48 weeks | Baseline | 3.141 | 1.427 | 0.091 | -0.338 | 6.621 |
|  |  | 24 weeks | 0.925 | 1.193 | 1 | -1.984 | 3.833 |
| Pain | Baseline | 24 weeks | -1.254 | 1.387 | 1 | -4.636 | 2.129 |
|  |  | 48 weeks | -2.917 | 1.352 | 0.101 | -6.214 | 0.381 |
|  | 24 weeks | Baseline | 1.254 | 1.387 | 1 | -2.129 | 4.636 |
|  |  | 48 weeks | -1.663 | 1.313 | 0.626 | -4.866 | 1.54 |
|  | 48 weeks | Baseline | 2.917 | 1.352 | 0.101 | -0.381 | 6.214 |
|  |  | 24 weeks | 1.663 | 1.313 | 0.626 | -1.54 | 4.866 |
| General Health | Baseline | 24 weeks | -3.445* | 1.191 | 0.014 | -6.349 | -0.54 |
|  |  | 48 weeks | -3.260* | 1.127 | 0.014 | -6.007 | -0.513 |
|  | 24 weeks | Baseline | 3.445* | 1.191 | 0.014 | 0.54 | 6.349 |
|  |  | 48 weeks | 0.185 | 0.816 | 1 | -1.805 | 2.174 |
|  | 48 weeks | Baseline | 3.260* | 1.127 | 0.014 | 0.513 | 6.007 |
|  |  | 24 weeks | -0.185 | 0.816 | 1 | -2.174 | 1.805 |

| **Repeated Measures ANOVA by gender for Emotional Wellbeing and General Health** | | | | | | | | | | | | | |
| --- | --- | --- | --- | --- | --- | --- | --- | --- | --- | --- | --- | --- | --- |
| Females |  |  |  |  |  |  | Time 1 | Time 2 | Mean Difference | Std. Error | p value | 95% Confidence Interval |  |
|  |  | df 1 | df 2 | F | p value | Partial Eta Squared |  |  |  |  |  | Lower Bound | Upper Bound |
| Emotional Wellbeing | 10mg | 2 | 14 | 1.322 | 0.298 | 0.159 | Baseline | 24 weeks | -2.5 | 3.202 | 1 | -12.513 | 7.513 |
|  |  |  |  |  |  |  |  | 48 weeks | -5 | 3.359 | 0.541 | -15.507 | 5.507 |
|  |  |  |  |  |  |  | 24 weeks | Baseline | 2.5 | 3.202 | 1 | -7.513 | 12.513 |
|  |  |  |  |  |  |  |  | 48 weeks | -2.5 | 2.612 | 1 | -10.668 | 5.668 |
|  |  |  |  |  |  |  | 48 weeks | Baseline | 5 | 3.359 | 0.541 | -5.507 | 15.507 |
|  |  |  |  |  |  |  |  | 24 weeks | 2.5 | 2.612 | 1 | -5.668 | 10.668 |
|  | Placebo | 2 | 24 | 2.491 | 0.104 | 0.172 | Baseline | 24 weeks | -3.077 | 2.179 | 0.55 | -9.134 | 2.98 |
|  |  |  |  |  |  |  |  | 48 weeks | -5.231 | 2.537 | 0.185 | -12.283 | 1.822 |
|  |  |  |  |  |  |  | 24 weeks | Baseline | 3.077 | 2.179 | 0.55 | -2.98 | 9.134 |
|  |  |  |  |  |  |  |  | 48 weeks | -2.154 | 2.337 | 1 | -8.648 | 4.341 |
|  |  |  |  |  |  |  | 48 weeks | Baseline | 5.231 | 2.537 | 0.185 | -1.822 | 12.283 |
|  |  |  |  |  |  |  |  | 24 weeks | 2.154 | 2.337 | 1 | -4.341 | 8.648 |
|  | 5mg | 2 | 28 | 2.756 | 0.081 | 0.164 | Baseline | 24 weeks | -6.933 | 2.985 | 0.107 | -15.045 | 1.179 |
|  |  |  |  |  |  |  |  | 48 weeks | -4.533 | 3.553 | 0.668 | -14.191 | 5.124 |
|  |  |  |  |  |  |  | 24 weeks | Baseline | 6.933 | 2.985 | 0.107 | -1.179 | 15.045 |
|  |  |  |  |  |  |  |  | 48 weeks | 2.4 | 2.336 | 0.965 | -3.948 | 8.748 |
|  |  |  |  |  |  |  | 48 weeks | Baseline | 4.533 | 3.553 | 0.668 | -5.124 | 14.191 |
|  |  |  |  |  |  |  |  | 24 weeks | -2.4 | 2.336 | 0.965 | -8.748 | 3.948 |
| General Health | 10mg | 1.225^ | 8.577 | 1.334 | 0.291 | 0.16 | Baseline | 24 weeks | -5 | 4.629 | 0.948 | -19.478 | 9.478 |
|  |  |  |  |  |  |  |  | 48 weeks | -5.625 | 4.272 | 0.688 | -18.985 | 7.735 |
|  |  |  |  |  |  |  | 24 weeks | Baseline | 5 | 4.629 | 0.948 | -9.478 | 19.478 |
|  |  |  |  |  |  |  |  | 48 weeks | -0.625 | 1.752 | 1 | -6.104 | 4.854 |
|  |  |  |  |  |  |  | 48 weeks | Baseline | 5.625 | 4.272 | 0.688 | -7.735 | 18.985 |
|  |  |  |  |  |  |  |  | 24 weeks | 0.625 | 1.752 | 1 | -4.854 | 6.104 |
|  | Placebo | 2 | 24 | 1.703 | 0.203 | 0.124 | Baseline | 24 weeks | -5 | 3.049 | 0.381 | -13.474 | 3.474 |
|  |  |  |  |  |  |  |  | 48 weeks | -4.231 | 2.933 | 0.524 | -12.384 | 3.922 |
|  |  |  |  |  |  |  | 24 weeks | Baseline | 5 | 3.049 | 0.381 | -3.474 | 13.474 |
|  |  |  |  |  |  |  |  | 48 weeks | 0.769 | 2.765 | 1 | -6.915 | 8.453 |
|  |  |  |  |  |  |  | 48 weeks | Baseline | 4.231 | 2.933 | 0.524 | -3.922 | 12.384 |
|  |  |  |  |  |  |  |  | 24 weeks | -0.769 | 2.765 | 1 | -8.453 | 6.915 |
|  | 5mg | 2 | 28 | 3.729 | 0.037 | 0.21 | Baseline | 24 weeks | -8 | 3.155 | 0.071 | -16.574 | 0.574 |
|  |  |  |  |  |  |  |  | 48 weeks | -5.333 | 3.29 | 0.382 | -14.275 | 3.609 |
|  |  |  |  |  |  |  | 24 weeks | Baseline | 8 | 3.155 | 0.071 | -0.574 | 16.574 |
|  |  |  |  |  |  |  |  | 48 weeks | 2.667 | 2.433 | 0.875 | -3.946 | 9.28 |
|  |  |  |  |  |  |  | 48 weeks | Baseline | 5.333 | 3.29 | 0.382 | -3.609 | 14.275 |
|  |  |  |  |  |  |  |  | 24 weeks | -2.667 | 2.433 | 0.875 | -9.28 | 3.946 |
| Males |  |  |  |  |  |  |  |  |  |  |  |  |  |
| Emotional Wellbeing | 10mg | 2 | 44 | 1.075 | 0.35 | 0.047 | Baseline | 24 weeks | -1.739 | 1.201 | 0.486 | -4.852 | 1.374 |
|  |  |  |  |  |  |  |  | 48 weeks | -0.87 | 1.231 | 1 | -4.059 | 2.32 |
|  |  |  |  |  |  |  | 24 weeks | Baseline | 1.739 | 1.201 | 0.486 | -1.374 | 4.852 |
|  |  |  |  |  |  |  |  | 48 weeks | 0.87 | 1.123 | 1 | -2.041 | 3.781 |
|  |  |  |  |  |  |  | 48 weeks | Baseline | 0.87 | 1.231 | 1 | -2.32 | 4.059 |
|  |  |  |  |  |  |  |  | 24 weeks | -0.87 | 1.123 | 1 | -3.781 | 2.041 |
|  | Placebo | 2 | 32 | 1.713 | 0.196 | 0.097 | Baseline | 24 weeks | -2.118 | 2.145 | 1 | -7.852 | 3.617 |
|  |  |  |  |  |  |  |  | 48 weeks | -3.529 | 1.875 | 0.234 | -8.541 | 1.482 |
|  |  |  |  |  |  |  | 24 weeks | Baseline | 2.118 | 2.145 | 1 | -3.617 | 7.852 |
|  |  |  |  |  |  |  |  | 48 weeks | -1.412 | 1.713 | 1 | -5.991 | 3.167 |
|  |  |  |  |  |  |  | 48 weeks | Baseline | 3.529 | 1.875 | 0.234 | -1.482 | 8.541 |
|  |  |  |  |  |  |  |  | 24 weeks | 1.412 | 1.713 | 1 | -3.167 | 5.991 |
|  | 5mg | 2 | 36 | 2.31 | 0.114 | 0.114 | Baseline | 24 weeks | -2.526 | 2.969 | 1 | -10.362 | 5.309 |
|  |  |  |  |  |  |  |  | 48 weeks | -5.684 | 2.395 | 0.087 | -12.006 | 0.637 |
|  |  |  |  |  |  |  | 24 weeks | Baseline | 2.526 | 2.969 | 1 | -5.309 | 10.362 |
|  |  |  |  |  |  |  |  | 48 weeks | -3.158 | 2.552 | 0.695 | -9.892 | 3.576 |
|  |  |  |  |  |  |  | 48 weeks | Baseline | 5.684 | 2.395 | 0.087 | -0.637 | 12.006 |
|  |  |  |  |  |  |  |  | 24 weeks | 3.158 | 2.552 | 0.695 | -3.576 | 9.892 |
| General Health | 10mg | 1.431^ | 31.489 | 0.936 | 0.374 | 0.041 | Baseline | 24 weeks | -0.217 | 2.073 | 1 | -5.588 | 5.154 |
|  |  |  |  |  |  |  |  | 48 weeks | -2.174 | 1.933 | 0.819 | -7.184 | 2.836 |
|  |  |  |  |  |  |  | 24 weeks | Baseline | 0.217 | 2.073 | 1 | -5.154 | 5.588 |
|  |  |  |  |  |  |  |  | 48 weeks | -1.957 | 1.077 | 0.249 | -4.747 | 0.834 |
|  |  |  |  |  |  |  | 48 weeks | Baseline | 2.174 | 1.933 | 0.819 | -2.836 | 7.184 |
|  |  |  |  |  |  |  |  | 24 weeks | 1.957 | 1.077 | 0.249 | -0.834 | 4.747 |
|  | Placebo | 1.51^ | 24.153 | 0.741 | 0.451 | 0.044 | Baseline | 24 weeks | -1.471 | 2.704 | 1 | -8.698 | 5.756 |
|  |  |  |  |  |  |  |  | 48 weeks | 1.765 | 3.235 | 1 | -6.883 | 10.413 |
|  |  |  |  |  |  |  | 24 weeks | Baseline | 1.471 | 2.704 | 1 | -5.756 | 8.698 |
|  |  |  |  |  |  |  |  | 48 weeks | 3.235 | 1.866 | 0.307 | -1.752 | 8.223 |
|  |  |  |  |  |  |  | 48 weeks | Baseline | -1.765 | 3.235 | 1 | -10.413 | 6.883 |
|  |  |  |  |  |  |  |  | 24 weeks | -3.235 | 1.866 | 0.307 | -8.223 | 1.752 |
|  | 5mg | 1.482^ | 26.677 | 3.615 | 0.053 | 0.167 | Baseline | 24 weeks | -4.211 | 3.017 | 0.539 | -12.173 | 3.752 |
|  |  |  |  |  |  |  |  | 48 weeks | -6.316* | 1.981 | 0.015 | -11.544 | -1.088 |
|  |  |  |  |  |  |  | 24 weeks | Baseline | 4.211 | 3.017 | 0.539 | -3.752 | 12.173 |
|  |  |  |  |  |  |  |  | 48 weeks | -2.105 | 2.035 | 0.944 | -7.475 | 3.264 |
|  |  |  |  |  |  |  | 48 weeks | Baseline | 6.316* | 1.981 | 0.015 | 1.088 | 11.544 |
|  |  |  |  |  |  |  |  | 24 weeks | 2.105 | 2.035 | 0.944 | -3.264 | 7.475 |
